# 19th European Burns Association Congress

**DOI:** 10.3390/ebj3040046

**Published:** 2022-11-25

**Authors:** Nadia Depetris, Maurizio Stella

**Affiliations:** Burn Center, CTO Hospital, Città Della Salute e Della Scienza, 10126 Turin, Italy

**Keywords:** burns, burn care, burn centres

## Abstract

Abstracts of the workshops, plenary sessions, oral and poster presentations of the 19th EBA Congress in Turin, Italy, 7–10 September 2022.

## 1. Introduction

Dear friends and colleagues,

It is an honor to present the abstracts of the 19th EBA Congress, held in Turin in September 2022. Some of you were here in person, and some were online; some will read the abstracts of the presentations for the first time in this special issue of the European Burns Journal. during the last two years, the period defined as the Covid Era, we faced a very complex situation. Healthcare professionals dedicated their energies to caring for patients during the spread of a pandemic. In many cases, health professionals were also victims of the COVID-19 infection. Therefore, it was an absolute pleasure to meet again in Turin. The 19th EBA Congress was comprehensive and articulated and provided us the opportunity to exchange experiences, ideas, and expectations in the field of burn care. The topic of “Identity” was developed during the Congress in Turin through a holistic and inclusive approach. Identity defines us as burn care professionals and guides us in treating burns and deciding the specific therapies needed, from lab examinations to wound healing to psychological support. It is Identity that our patients are looking for, after their traumas, for social reintegration purposes and to recover as individuals. Burn care professionals from different fields joined the Congress and looked at this crucial concept from different points of view. Experienced colleagues shared their knowledge in the various sessions, and the youngest professionals proposed new contributions. Technical and scientific advancements were presented, and humanistic disciplines, such as narrative medicine, arts, and social sciences, were included to provide a broader understanding of burn care. We are sure that, reading and studying this special issue of the EBJ, all of us will find a deeper conception of our everyday practice and a fresher motivation to develop further and research burn care.

## 2. Workshops

*W.001*

**My Softest Scar: Tension Reducing Taping to Improve Scarring**

**Peter Moortgat ^1^ and Danila Toscano ^2^**
  ^1^  OSCARE, Antwerp, Belgium
  ^2^  Physical Medicine and Rehabilitation University, Orthopedic and Trauma, Center (CTO), Città della Salute e della Scienza, Turin, Italy

Burn scars require a multimodal and multidisciplinary approach to improving aesthetic and functional results. Tension-reducing taping showed beneficial effects in the post-acute rehabilitation phase of burns. This workshop allows you to meet physical therapists of two different European centers who integrate the use of taping into their multimodal approach to post burns care. They will share with you the theory behind their methods, their practical approaches toward physical scar management, and the demonstration of their specific tension-reducing taping techniques.

*W.002*

**Visual Arts in Burn Surgery**

**Mark Fisher ^1^, Laura Pompermaier ^2^, Dacia Mastrantuono ^3^, Anna Marchisio ^3^ and Elisa Curestiuc ^4^**
  ^1^  The University of Iowa, USA
  ^2^  Linköping University, Sweden
  ^3^  Arts High School, Turin, Italy
  ^4^  Language High Scool, Turin, Italy

Visual arts introduce different ways of thinking and perceiving the identity and beauty of people.
The practice of visual arts, such as drawing, sculpture and photography, may be integrated into the burn reconstructive process, allowing professionals and patients to see, perceive and feel the self after injury.
The Workshop will include two parts: a didactic session reviewing the use of visual art tools in the planning of reconstructive burn surgery, and a practical charcoal studio drawing session with the guidance of art students and experienced instructors. Participants will draw a series of ‘virtual models’ with burn contractures and deformities. Through this experience we will develop our ability to burn reconstructive process.

*W.003*

**Optimal Nutritional Therapy in Patients with Burns: From Theory to Practice**

**Yvonne Verweij-Tilleman ^1^, Frederiek G. Bosch ^2^ and Daren K. Heyland ^3^**
  ^1^  Burn Centre Maasstad Hospital, Rotterdam, The Netherlands
  ^2^  Association of Dutch Burn Centres and Burn Centre Groningen, Martini Hospital, The Netherlands
  ^3^  Department of Medicine, Queen’s University, Kingston, Canada

Nutritional therapy is an essential part of the multidisciplinary burn treatment.
In fact, burns induce a hypermetabolic, inflammatory and catabolic state, leading to increased nutritional demands. Suboptimal nutritional therapy, and immobilisation or lack of exercise further worsen nutritional status. Malnutrition in hospitalised patients is associated with higher morbidity and mortality, decreased wound healing, daily functioning, substantial longer in hospital stay, and increased costs.
However, feeding patients with severe burns according to current guidelines can be a challenge.
During this workshop, both theory and practice of delivering optimal nutritional therapy in patients with burns will be covered. Current state of the evidence, including current scientific knowledge gaps, practical experiences and opportunities for the optimisation of nutritional therapy will be shared and discussed with participants.

*W.004*

**Cosmetic Camouflage Make-Up for Restoring Burn Survivors’ Identity**

**Franck Duteille ^1^, Stefania Anna Simone ^2^, Silvia Magi ^3^ and Antonella Lanzoni ^4^**
  ^1^  CHU de Nantes, Service de Chirurgie Plastique Reconstructrice et Esthétique, Centre des Brûlés Adultes et Enfants, Faculté de Médecine de Nantes
  ^2^  University Hospital, Graz, Austria University Hospital, Zurich, Switzerland
  ^3^  Orthopedic and Trauma Center (CTO), Cittá Della Salute e Della Scienza, Turin, Italy
  ^4^  Municipality of Turin, Italy

Visible scars and skin irregularities, after burn injuries, alter a person’s body image, self-esteem and result in significant distress. Medical camouflage offers a way to color-match skin irregularities and reduces the visual attention to the scars from people around those affected. Camouflage is an important tool that gives those affected a choice over their appearance and a sense of control over how they want to look every day. during this workshop, you will have the opportunity to learn more about the application of camouflage techniques: starting with theoretical basics, turning over to practical examples and discussing tips based on personal experiences. Camouflage’s role in rebuilding confidence after burn injuries will be discussed in the panel afterwards.

## 3. Plenary Sessions

*PS.001*

**Interactive Educational Course: Clinical Challenges**
**Clemens Schiestl, Zurich, Switzerland**
**Nadia Depetris Turin, Italy**
**Anna Schildt, Helsinki, Finland**
**Jill Meirte, Antwerp, Belgium**
**Renate Pfann, Zurich, Switzerland**
**Livia Emma, Turin, Italy**
**Simona Cagnazzo, Turin, Italy**
**Athina Lavrentieva, Thessaloniki, Greece**
**Francesco de Rosa, Turin, Italy**
**Filippo Mariano, Turin, Italy**
**Paolo Costa, Turin, Italy**

A 40-year-old woman is admitted to the Burn Centre. She has III degree burn injury to her face and hands due to flame. We will follow the clinical evolution of the patient till her discharge from the Burn Centre and discuss the clinical challenges she posed with an international multidisciplinary team of burn care experts.

*PS.002*

**The Journey of the Cultured Keratinocyte: Where Do We Come from—What Lies Ahead**
**Naiem Moiemen, Birmingham, UK**
**Maurizio Stella, Turin, Italy**
**Clemens Schiestl, Zurich, Switzerland**
**Anthony de Buys Roessingh, Losanne, Switzerland**
**Miguel Alaminos Mingorance, Granada, Spain**
**Sophie Böttcher, Zurich, Switzerland**

Experienced professionals will present in this session the historical development and ongoing research of human keratinocyte cultures, focusing on the European regulatory framework in the field and the possible clinical applications for burn care.

*PS.003*

**Identity in Burn Care through Bioethics, Social Sciences, and Arts**
**Folke Sjioberg, Linkoping and Sweden**
**Sophia Papadopoulou, Thessaloniki, Greece**
**Marta Allué, Tarragona, Spain**
**Mark Fisher, Iowa, USA**
**Paola Chesi, Milan, Italy**

Different perspectives from humanistic disciplines for understanding burn care.

*PS.004*

**Identity after Fire Disasters**
**Stian Almeland, Bergen, Norway**
**Koen Maertens, Brussel, Belgium**
**Eleonora Balzani, Turin, Italy**

The session develops the themes of prevention and mass-casualties management starting from a cinema discussion. Il sole sulla pelle (Italy, 2018, 62 mins) is an Italian documentary film, directed and written by Massimo Bondielli e Gino Martella, produced by “Caravanserraglio Film Factory” and “Il mondo che vorrei onlus”. Il sole sulla pelle is based on the 2009 Viareggio railway station explosion, which caused 32 deads and several victims. The movie is especially centered on survivors and victims’ families, who have been dedicated their lives in search of truth, justice and disasters’ prevention.

*PS.005*

**Burn Surgery: A Multidisciplinary Team Approach to Surgical Management**
**Tina Palmieri, Sacramento, California, US**

The medical management of burn injury requires integrating multiple different specialties, particularly in the operating room. The goal of this presentation is to describe how the multidisciplinary team can be used in the operating room to optimize surgical outcomes.

*PS.006*

**Can Nutrients Be Used to Improve Outcomes of Severe Burns?**
**Daren Heyland, Toronto, Canada**

Do you think that nutrients can impact the clinical outcomes of severe burn patients?
In this session will be presented the results of the largest randomized trial of severe burn patients ever conducted, The RE-ENERGIZE trial, which randomized 1200 patients from 54 burn units from 14 countries to receive enteral glutamine or placebo. In the same presentation, the background rationale for the VICToRY will be presented. The VICToRY trial aims to enroll 666 patients with severe burns to study the effect of high dose intravenous vitamin C.

*PS.007*

**The European Burn Journal: Reporting Science Research to Progress in Burn Care**
**Naiem Moiemen, Birmingham, UK**
**Anthony de Buys Roessingh, Losanne, Switzerland**

Aims and scopes of the official journal of the European Burns Association will be presented, together with a special insight in the process of reading and writing scientific papers.

*PS.008*

**Should Prognostic Scores Guide End of Life Decisions in Burn Care? (Pro-Con Debate)**
**Athina Lavrentieva, Thessaloniki, Greece**
**Jyrki Vuola, Helsinki, Finland**
**Valentina Genco, Turin, Italy**
**Folke Sjioberg, Linkoping, Sweden**
**Peter Vogt, Hannover, Germany**
**Marco Vergano, Turin, Italy**

A review of the main prognostic scores from burn care literature, discussing their application and possible integration in clinical decisions.

*PS.009*

**Special Insights in Infections in Burn Care**
**Francesco De Rosa, Turin, Italy**
**Luis Cabral, Coimbra, Portugal**
**Thomas Leclerc, Clamart, France**
**Silvia Scabini, Turin, Italy**
**Marisa Caetano, Coimbra, Portugal**

Specific topics about infections in burns will be presented and discussed, such as the possible application of bacteriophages, the occurrence of fungal infections, and the pharmacokinetics and pharmacodynamics of antibiotics in burn patients.

*PS.010*

**Who Is the Burn Care Professional? (Round Table)**
**Clemens Schiestl, Zurich, Switzerland**
**Sandra Cristina Marola Sanchez, Turin, Italy**
**Tina Palmieri, Sacramento, USA**
**Alette De Jong, Beverwijk, The Netherlands**
**Julia Elrod Zurich, Switzerland**
**Teresa Tredoux, London, UK**
**Stian Almeland, Bergen, Norway**
**Thomas Leclerc, Clamart, France**

The multidisciplinary team approach is essential for the success of burn care. How should the different health professionals integrate? Knowledge, clinical experience, and non-technical skills are the keys.

*PS.011*

**To Cut or Not to Cut?**
**Peter Moortgat, Antwerp, Belgium**

After introducing the general principles of scar management, clinical cases will be presented discussing with the participants different options of treatment.
When will we choose non-invasive, semi-invasive, or invasive burn scar management?

*PS.012*

**Tube or Not Tube?**
**Tina Palmieri, Sacramento, USA**

After introducing the general principles of airway management and ventilation in burns, clinical cases will be presented discussing with the participants different options of treatment. When will we choose to intubate or ventilate our patient? Maybe will we perform a tracheostomy?

## 4. Keynote Lectures

*K.001*

**Acute Surgical Management in Austere Settings**
**Habib Ur Rahman Qasim, Kabul, Afghanistan**

The Burn Unit at Indira Gandhi Hospital (Kabul, Afghanistan) is the country’s sole children’s burn unit.
The Indira Gandhi Hospital is a 400 beds hospital located in Kabul, Afghanistan. The Burn Unit was established in 2013 and, thanks to the support of several international organizations (Kinderspistal Zurich, SDC, Interburns UK, and ECPB), nowadays have 36 beds for burns, serving around 1000 burn patients per year (mean TBSA 25%, mean age 6 years). The leading cause of burns is scalded (around 67%). The help of international organizations played a critical role in the development of staff capacity through coaching, mentoring, training, and exposure visits. These learning opportunities played a vital role in building capacities and upgrading the knowledge of the Indira Gandhi staff in burn care.

*K.002*

**The Use of Procalcitonin to Guide Antibiotic Therapy in Burns**
**Amen Messadi, Intensive Burn Care Department, Trauma and Burn Center, Tunis, Tunisia**

Introduction: The use of biomarkers, necessarily coupled with close clinical examination, may predict outcomes, and monitor the efficacy of antimicrobial therapy, allowing faster de-escalation or stop. reducing the development of resistance and the financial cost. The objective of our study was to evaluate the interest of plasma procalcitonin (PCT) biomarker to guide the initiation, modification and discontinuation of antibiotics in septic burn patients. Methods: a prospective, observational study was conducted in a 20-bed Burn Intensive Care Unit in Tunisia, for 13 months (August 2018–September 2019). Burn patients admitted in ICU developed sepsis were included. Sepsis was retained according to the French Burn Association Criteria for the presence of infection. Patients who died or were discharged before day 3 of the protocol were excluded. Serum PCT was measured over the entire septic episode every 48 h until resolution of infection based on clinical signs and decrease of PCT about 80% compared to its of initial value. Patients were assigned to two groups depending on the clinical course and outcome: Group A = patients with favorable evolution; Group B = patients with unfavorable evolution. Results: A total of 120 septic patients admitted to the Burn ICU were included in our study. The mean age was 32 ± 17 years. TBSA was 32 ± 14%. UBS was 40 ± 25 and ABSI was 6 ± 2. Secondary transfer was reported in 55% of cases. They were mainly thermal burns in 79% of cases and electrical burns in 21%. Sepsis occurred at 3 days after burns. Initial PCT was positive (>0.69 ng/mL) in all patients with a median value of 12 ng/mL (maximum 200 ng/mL). Conclusion: monitoring PCT in septic burn patients allows to Predict effectiveness of sepsis treatment at day 3 when decreased by 30% or above compared to its initial value and lead to decrease the duration of antibiotic treatment with an average duration of treatment of 5 days reducing antibiotic exposure and selective pressure of multidrug resistance bacteria.

*K.003*

**The Importance of Data in Burn Care**
**Prof. Naiem Moiemen, Birmingham, United Kingdom**

No abstract available.

*K.004*

**Enzymatic Debridement: Indications, Technique and Complications**
**Maurizio Governa, Verona, Italy**

Enzymatic debridement is a powerful tool for selective burn eschar removal as an alternative of early surgical debridement. We retrospectively analyze our experience with Nexobrid from 2017 to 2022: 136 patients were treated (mean age 54 y.o.), with deep second and third-degree burns involving a mean 27%TBSA. We revised the indications and the technique already approved by the European consensus and SIUST Italian consensus; clinical outcome is presented focusing and burns involving the hands and the face, subpopulations of pediatric and elderly patients, severe (<40%TBSA) burns. The procedure, if correctly performed in the first 72 h from trauma, proved to be a powerful tool to remove eschar, reducing blood loss and the need for autologous skin.

*K.005*

**Keynote Lecture: From Basic Research to Clinical Practice**
**Tiziana Musso, Turin, Italy**

Bacterial infections are a main factor in determining burn wound progression. The evolution of resistance to last-resort antibiotics, such as colistin (Col), in multidrug–resistant (MDR) Gram-negative bacteria (GNB), including Pseudomonas aeruginosa, Acinetobacter baumannii and Klebsiella pneumoniae, causes a relevant problem in the treatment of burn wound infections.
Progress in nanomedicine has provided a chance to develop nanotherapeutics that efficiently prevent infections and facilitate healing of burn wounds. Currently nanostructures include liposomes and lipid nanoparticles (NPs), polymer nanodrugs, protein and metal-based NPs. These nanocarriers have been proposed for antibiotic delivery to increase drug at the infection sites and manage antimicrobial resistance. New delivery systems for Col, to be used both for topic and parental administration may improve bioavailability and toxicity. We developed a for mulation of chitosan-coated human albumin nanoparticles for the Col delivery (Col/haNPs) and evaluated the antimicrobial and antibiofilm activity as well as the biocompatibility. We next optimized a topical for mulation named “NANOFILM”, based on a film for ming spray (glycerol, etilacetate and the copolymer Plastoid^®^ B) and Col/haNPs. The Col/haNPs showed size lower than 200 nm, high encapsulation efficiency and a prolonged in vitro release of Col. The safety of the nano for mulation was demonstrated by the absence of cytotoxicity on epithelial cells and human skin and hemolytic activity. Both Col/haNPs and the NANOFILM can reduce the MIC values respect to free Col, in both MDR Col resistant and Col susceptible strains. Moreover, the Col/haNPs displayed a potent biofilm inhibition and significantly reduced the biofilm at 1/2 MIC. By contrast, Col free is able to reduce biofilm only at higher concentrations. The antimicrobial effect of NANOFILM was also demonstrated in an ex vivo skin infection model using A. baumannii. Our findings suggest that Col/haNPs represent a promising nanocarrier for Col topical delivery with high antimicrobial activity on MDR GNB.

*K.006*

**Burn Care Outcomes: An International Perspective**
**Laura Pompermaier, Sweden**

Measurable changes in the health status of an individual attributed to provided interventions are defined as health care outcomes, and they can be used to evaluate the quality of care, highlighting effective strategies. The ISBI seeks to a global standard of burn care but had not yet indicated burn care specific outcomes of global interest, which might contribute to increase transparency, guide quality improvement, and harmonize the standard of care. for this reason, in 2021 the ISBI-Burn Care Committee developed a survey to identify quality indicators for the specific need of burn care, based on scientific evidence, and distributed it among international burn care providers. Most of 124 responders were physicians from Americas and Europe, although responders included individuals from all WHO-regions and from all burn care professionals. More than 80% of responders indicated in-hospital mortality and incidence of severe infections as significant outcome-quality indicators. If on the one hand establishing mortality is indisputable, on the other hand assess severe infections is not as easy, since there is no professional consensus on a universal definition of infection. Development of pressure sores during the hospitalization was considered important by only 1% of responders, and this finding might mirror the low response rate of nursing staff (5%). Agreement on feasible outcome-indicators is a first step to achieve a global standard of care. However, since burn care is resource-intensive and dependent on resources availability, quality indicators of the structural and processual characteristics of settings in which burn care is provided should also be evaluated to contextualize and interpret the findings on outcomes.

*K.007*

**Toxicology in Burn Patients**
**Romolo Villani, Naple, Italy**

The number of intoxications among severe burns is underestimated in hospital cases since most of them die at the scene of the accident. Carbon monoxide and cyanide, generated by the combustion of organic and plastic products, are the main agents that through the inhalation route lead to intoxication of the burn victim. These substances interfere with blood transport and mitochondrial use of oxygen. We have tools that allow us to quickly and effectively identify monoxide in the blood of our patients, while unfortunately a specific and appropriate diagnosis for cyanide intoxication is not yet available. The latter should be suspected whenever carbon monoxide poisoning occurs, with high lactate values, especially if higher than 10 mmoL/L. Only immediate treatment, started already in the pre-hospital phase, can reduce the number of inhalation victims. Therefore, rescuers must have specific diagnostic equipment and everything needed for treatment: high concentration oxygen, high doses of Vit. B12, hyperbaric therapy. Burn patients who have inhaled even when they arrive at the hospital have a worse prognosis. With the same age and extent of burns, the risk of death is almost doubled. But monoxide and cyanide are not the only poisons with which the burned person can come into contact. Methemoglobinizing substances and many other chemicals can be involved in the poisoning of burn victims. Also in these cases, the speed of diagnosis and treatment is essential, through the use of all available antidotes.

*K.008*

**Keynote Lecture: Principles of Burn Wound Closure**
**Juan P. Barret, Spain**

No abstract available.

*K009*

**Principles of Scar Treatment in Rehabilitation**
**Peter Moortgat, Oscare, Antwerp, Belgium**

Rehabilitation is only one part of the multi-disciplinary approach of burn scar management, although it might be considered as the most time-intensive part. To obtain an optimal outcome 6 key principles should always be kept in mind. Know your patient. It is indispensable to know as much as possible about your patient by means of an intake for m. Examples of intake variables are age, gender, genetic predisposition, blood pressure, hormonal activity, occupation, etc… Listen to your patient. Nowadays patient empowerment has become increasingly important. PROMs are an important tool to know what is important to your patient. POSAS, EQ-5D, BSHS-B, SF-36 and DLQI are the most widely used PROMs in (burn) scar management. Measure. In scar assessment objective and subjective measurement tools are equally important and should be used both. Colour, thickness, pliability, texture and hydration are the most frequently measured scar characteristics. Choose the right treatment. The objective and subjective assessments for m a base to choose the right treatment. To date there exists a wide variety of treatment algorithms depending on the type of scar (e.g., hypertrophic, linear, burn, keloid). Know what you are doing inside that scar. Knowledge of the working mechanisms of different scar treatments is inevitable to obtain the best outcomes. Scientific literature can help us to increase our understanding of the mechanisms leading to scar remodelling. Re-evaluate and adjust treatment. Re-evaluation should be performed on a regular basis. The results of these assessments help us to evaluate the effectiveness of our treatment plan. According to the results adjustment of the treatment plan can be necessary. To optimize our outcomes, we have to raise awareness about treatment options, improve health literacy of the patient and educate specialized caregivers. Digital care pathways, informative websites and specialist search engines can be helpful tools to reach our goals.

*K.010*

**Basic Principles of Burn Care in Children**
**Martin Meuli, Switzerland**

No abstract available.

*K.011*

**The EBA Verification Program**
**Clemens Schiestl, Switzerland**

No abstract available.

*K.012*

**Reconstruction in Austere Settings: The Role of International Organizations**
**Enrique Steiger, Switzerland**

No abstract available.

*K.013*

**How COVID-19 Has Been Affecting Burn Care**
**Thomas Leclerc, Clamart, France**

Since 2020, the pandemic of COVID-19 has caused an unprecedented worldwide healthcare crisis. It has impacted almost all human activities including burn care. Burns epidemiology changed. Hospital admissions for burns decreased or remained stable, with differences among age groups. They were related to more severe burns, suggesting appropriate triage. Scalds increased in children and so did psychiatric disorders in adults. Many fire disasters occurred in hospitals. The organization of burn care also changed. Thanks to quick adaptation, despite shortages, burn care successfully went on. Burn treatment capacity often decreased. Many burn professionals helped manage COVID-19 patients. Before vaccines, COVID-19 positive burn patients had to be cohorted in dedicated isolation units, hence a need for complex triage. Infection control and prevention involved strictest zoning, hygiene and personal protective equipment procedures. Regional strategic planning, astute management of limited resources, preservation of burn care workforce, strict cohorting, and adaptation of treatment strategies were fundamental to tackle the crisis. Under pressure, telemedicine received a boost that will last. after vaccines, less severe outbreaks occurred. COVID-19 positive burn patients needed no more cohorting but still highest hygiene and safety precautions. Saturated ICUs had to triage not only COVID-19 patients but also other critical conditions including severe burns. Surgical procedures had to be delayed according to prioritization rules. Critically ill patients including those with severe burns also had to undergo prioritization for critical care. COVID-19 associated with burns posed specific medical challenges, but whether it worsened outcomes is unclear. ARDS was managed according to general recommendations with adjusted isolation precautions. Eligible patients received dexamethasone without burn specificity. On the whole, burn care providers hardly yet successfully met major organizational challenges during the crisis, but COVID-19 is not over. Further research is still highly needed.

*K.014*

**May We Prevent Burn Injuries? The EBA Activities**
**Mamta Shah, Manchester, United Kingdom**

Burn injuries are a global problem acknowledged by WHO (A WHO plan for burn prevention and care 2018). Apart from deaths, non-fatal burns are the leading cause of morbidity, disabilities and disfigurement with many man-h of work lost. Burn care is a huge burden to the health systems across the globe. Hence prevention has been the focus of health professionals in burn care for decades. Despite some inroads into prevention by legislative changes to themoregulation valves for hot water, building regulations, nightwear legislation as a few examples of success stories, we are far from achieving our goals particularly for children. It is time to revisit the history of prevention and understand the need to engage with a wide range of professionals, patients and carers to make an impact in preventing burn injuries. Prevention programmes should be tailored to the needs of the local populations in the areas and this requires an understanding of the key causes of burn injuries. Data collection is fundamental to measuring the impact of any prevention programme but awareness of how burns occur in the population, goes a long way in preventing the problem. Public engagement is key to success. The EBA is best placed to share best practises (education), support and promote data collection (burns awareness, share real life stories, public engagement), collaborate (research and development of products causing burn injury, engineering, geo-mapping of burn injuries) and advocacy (legislation, governments) and work with other non-governmental organisations to support the common cause of prevention of burns.

*K.015*

**Small Adults? Insights in Pediatric Burn Care**
**Naiem Moiemen, United Kingdom**

No abstract available.

*K.016*

**International Humanitarian Organizations and Burn Care**
**Tom Potokar, Switzerland**

The aim of any humanitarian organization working in burn care should be to reduce the incidence of burn injuries and to improve the outcomes. A simple enough aim in theory, but very difficult to put into practice in the complex environment of low resources, insecurity and competing demands. There is no one size fits all approach and individuals and organisations should be aware of their strengths and their limitations. Different organisations have different skill sets and modes of operation and emergency, conflict, and development work require different responses (for example NGO’s experienced in emergency response and adhering to WHO EMT minimum standards are appropriate to respond to a mass casualty burn event—professional associations are not—although they may have members who can deploy with a suitable NGO) There is a gradient from complete substitution to embedded support to distance based support, and different contexts will determine which of these is the most appropriate. However, whatever the approach there should be no ‘us ‘ and ‘them’ only ‘we’. Assessment must be comprehensive and look at assets as well as needs and the goal must be to achieve at least minimum standards of care. Education and training resources must be contextualized and tailored to the specific requirements and resources should be screened for quality and relevance. The final pathway to improvement is behavior change and motivation as well as an understanding of the knowledge to action pipeline, implementation science and health systems are as critical as purely clinical skills for creating sustainable impact. However, stainable change is ultimately a political, educational and socio-economic issue.

*K.017*

**Antibiotic Stewardship in Burn Patients**

**Athina Lavrentieva**
  A-ICU, Burn ICU, Papanikolaou Hospital, Thessaloniki, Greece

Antibiotic stewardship is a central component of approach to management of infectious complications in burn patients. Antibiotic stewardship has been defined as the coordinated interventions designed to improve and measure the appropriate use of antimicrobials by promoting the selection of the optimal antimicrobial drug regimen, dose, duration of therapy, and route of administration. The key elements of antibiotic stewardship include: leadership, prospective audit and feedback, antibiotic reevaluation (time-out), rapid diagnostics and laboratory testing, clinical pathways, computerized decision support, infection control (preventive strategies that reduce the incidence of nosocomial infection). Implementing ASP in the burn unit environment is challenging, as no single measure alone will be successful. The three pivotal moments during an antibiotic therapy are: the initiation of the antibiotic treatment, the moments of re-evaluation during treatment also known as the antibiotic time-out, the de-escalation and discontinuation of an antibiotic treatment. Source control is an essential component in the management of burn would infection. Antibiotic use should be considered a core competency of burn practitioners; as one of the largest consumers of antibiotics in the hospital, Burn Unit is well situated to reap benefit from an implementation of effective practices of ASP: (1) Collect microbiological samples before starting antimicrobial therapy (2) De-escalate antibiotics (3) Reduce the duration of antibiotic therapy as much as possible (4) Do not administer antibiotics for burn wound infection without clinical indications of infection (5) Address source control as rapidly as possible (e.g., catheter removal, abscess drainage, wound excision-grafting) (6) Optimize antibiotic pharmacokinetics and pharmacodynamics (PK/PD) parameters (7) Monitor therapeutic drug concentration.

*K.018*

**Analyzing the Root Cause of Burn Scars: The Key to Designing Effective Prevention and Treatment**

**Mark Fisher, Iowa, USA**

Every time we have a clinic encounter with a burn patient with scars and contractures, we are faced with the challenge of understanding the root cause of the problem before us. Principles of plastic surgery were perhaps more emphasized in previous generations and our approach to analysis and pedagogy would benefit by a return to principle-based practice. And yet there is also room for considerable elaboration of this concept. Our analysis of the burn scar and contracture needs to be informed by more than a physiologic analysis: we also need to understand the contracture in terms of the patients’ perspective and in the larger psychosocial context. during the present session we review multiple illustrative cases to explore root-cause analysis of the burn scar contracture in this light.

## 5. Oral Presentations

*O.001*

**A Prospective Multi-Centre Randomised Study for the Treatment of Burns with Dermis Grafts or Split-Thickness Skin Grafts**

**Andrew Lindford ^1^, Sinan Dogan ^2^, Moustafa Elmasry ^2^, Folke Sjöberg ^2^, Esko Kankuri ^3^, Jyrki Vuola ^1^, Jussi Valtonen ^1^, Ahmed T. El-Serafi ^2^, Marina Perdiki Grigoriadi ^4^, Islam Abdelrahman ^2^, Ingrid Steinvall ^2^, Matilda Karlsson ^2^ and Pia Olofsson ^2^**
  ^1^  Helsinki Burn Centre, Helsinki University Hospital
  ^2^  Department of Hand Surgery, Plastic Surgery and Burns in Linköping and Department of Biomedical and Clinical Sciences, Linköping University
  ^3^  Department of Pharmacology, Faculty of Medicine, University of Helsinki
  ^4^  Department of Clinical Pathology, and Department of Biomedical and Clinical Sciences, Linköping University

**Objectives**: The standard split-thickness grafting (STSG) technique has been used for 150 years with only limited improvements. Its limitations are well known and relate to donor-site morbidity and recipient-site aesthetic issues (mesh patterns, wound contracture, and scarring). The aim of this study was to investigate whether donor-site morbidity (healing time and cosmesis) could be reduced by a novel, modified STSG technique using only the dermal component. The secondary objective was to examine the effects of this pure dermis graft on the recipient site in comparison to a standard STSG.
**Methods**: We report a prospective, randomised controlled, multi-centre, intra-individual comparison study. Twenty-one patients received a dermis graft and a regular STSG. Aesthetic and scar assessments were performed using The Patient and Observer Scar Assessment Scale (POSAS) and a Cutometer Dual MPA 580 on both donor and recipient sites. These were also examined histologically for remodelling and scar for mation.
**Results**: Dermis and the STSG donor sites healed in 8 and 14 days, *p* < 0.005, respectively. Patient-reported POSAS showed better values for colour at all three time measurements (3, 6, and 12 months) and the observers rated both vascularity and pigmentation to be improved at these time points (*p* < 0.01). Scar for mation was seen at the dermis donor and recipient sites after 6 months as in the standard scar healing process.
**Conclusions**: The dermis graft reduced donor site morbidity, as it healed faster and with better cosmesis than the standard STSG. The overall long-term outcome favoured the dermis grafting technique. However, this needs to be further explored.
Trial registration: ClinicalTrials.gov Identifier (NCT05189743).

*O.002*

**Meek Micrografting for Burns; Review on its Outcomes. Searching for the Superior Skin Grafting Technique**

**Danielle Rijpma ^1,2^, Karel Claes ^3,4^, Henk Hoeksema ^3,4^, Ignace de Decker ^3,4^, Jozef Verbelen ^3,4^, Stan Monstrey ^3,4^, Anouk Pijpe ^1,2,5,6^, Paul van Zuijlen ^1,2,6,7,8^ and Annebeth de Vries ^1,8,9^**
  ^1^  Burn Centre of Red Cross Hospital
  ^2^  Amsterdam UMC Location Vrije Universiteit Amsterdam, Plastic, Reconstructive and Hand Surgery
  ^3^  Department of Plastic Surgery, Ghent University Hospital
  ^4^  Ghent Burn Center, Ghent University Hospital
  ^5^  Association of Dutch Burn Centres, Burn Centre, Red Cross Hospital Beverwijk
  ^6^  Amsterdam Movement Sciences Institute, Amsterdam UMC
  ^7^  Department of Plastic, Reconstructive and Hand Surgery, Red Cross Hospital
  ^8^  Department of Pediatric Surgery, Amsterdam UMC, location AMC
  ^9^  Department Surgery, Red Cross Hospital

**Objectives**: Autologous split-thickness skin grafting is the standard of care for most deep dermal and full-thickness burns. Meshed grafting is most commonly used. Patients with extensive burn injuries have limited donor-site availability. Meek micrografting is a well-known technique to enable larger expansions. We conducted a review on the outcomes of micrografting.
**Methods**: A literature search in PubMed, Web of Science, Google Scholar and the Cochrane Library databases was conducted from the first report of micrografting in 1958 until February 2021, including the terms ‘burns’, ‘micrografting’ and/or ‘Meek’. Original papers reporting the outcomes of Meek micrografting were included.
**Results**: In total, 1529 papers were identified, and 15 articles were included. Most studies were rated as poor study quality. Weighted averages could be calculated for three outcome parameters: 82 ± 7% for ‘graft take’, 51 ± 18 days for ‘time to wound healing’ and 53 ± 23 days for ‘length of hospital stay’. Scar quality was minimally described and often poorly assessed. Limited data were available on the outcomes’ donor-site size, bacterial load and rate of wound infection, number of operations and cost effectiveness.
**Conclusions**: Multiple outcomes of Meek micrografting from the 15 included studies were evaluated. The overall study quality was poor, and there is a specific lack of data on scar quality. Therefore, it is not possible to draw conclusions on the outcomes of Meek micrografting. To further investigate the performance of Meek micrografting, a randomized controlled trial is required.

*O.003*

**Hydrosurgical and Conventional Debridement of Burns: Randomized Clinical Trial**

**Nine Legemate ^1,2^, Kelly Kwa ^3^, Harold Goei ^1^, Anouk Pijpe ^3^, Esther Middelkoop ^2,3^, Paul van Zuijlen ^2,3^, Gerard Beerthuizen ^4^, Marianne Nieuwenhuis ^4^, Margriet van Baar ^1^, Kees van der Vlies ^1^ and the HyCon Study Group ^1,2,3,4^**
  ^1^  Burn Centre, Maasstad Hospital
  ^2^  Department of Plastic Surgery, AmsterdamUMC
  ^3^  Burn Centre, Red Cross Hospital
  ^4^  Burns Centre, Martini Hospital

**Objectives**: Tangential excision of burned tissue followed by skin grafting is the cornerstone of burn surgery. Hydrosurgery has become popular for tangential excision, with the hypothesis that enhanced the preservation of vital dermal tissue reduces scarring. The aim of this trial was to compare scar quality after hydrosurgical versus conventional debridement before split-skin grafting.
**Methods**: A double-blind randomized within-patient multicentre controlled trial was conducted in patients with burns that required split-skin grafting. One wound area was randomized to hydrosurgical debridement and the other to Weck knife debridement. The primary outcome was scar quality at 12 months, assessed with the observer part of the Patient and Observer Scar Assessment Scale (POSAS). Secondary outcomes included complications, scar quality, colour, pliability, and histological dermal preservation.
**Results**: Overall, 137 patients were randomized. At 12 months, scars of the hydrosurgically debrided wounds had a lower POSAS observer total item score (mean 2.42 (95 per cent c.i. 2.26 to 2.59) versus 2.54 (95 per cent c.i. 2.36 to 2.72; *p*  =  0.023)) and overall opinion score (mean 3.08 (95 per cent c.i. 2.88 to 3.28) versus 3.30 (95 per cent c.i. 3.09–3.51); *p*  =  0.006). Patient-reported scar quality and pliability measurements were significantly better for the hydrosurgically debrided wounds. Complication rates did not differ between both treatments. Histologically, significantly more dermis was preserved with hydrosurgery (*p*  <  0.001).
**Conclusions**: One year after surgery, scar quality and pliability were better for hydrosurgically debrided burns, probably owing to enhanced histological preservation of the dermis.

*O.004*

**The Timing of Surgery of Intermediate Depth Burns**

**Denise Van Uden ^1^, Inge Spronk ^1,2,3^, Margriet E. van Baar ^1,2^, Anouk Pijpe ^4,5,6,7^, Annebeth Meij-de Vries ^4,9^, Marianne K. Nieuwenhuis ^10,11,12^, Sonja M. H. J. Scholten-Jaegers ^10^, Esther Middelkoop ^5,13^, Eelke Bosma ^10,14^, Paul P. M. van Zuijlen ^6,7,8,9^ and Cornelis H. van der Vlies ^1,15^**
  ^1^  Association of Dutch Burn Centres, Maasstad Hospital
  ^2^  Department of Public Health, Erasmus MC, University Medicical Center Rotterdam
  ^3^  Dutch Burns Foundation
  ^4^  Burn Centre, Red Cross Hospital
  ^5^  Association of Dutch Burn Centres (ADBC)
  ^6^  Amsterdam UMC location Vrije Universiteit Amsterdam, Department of Plastic Reconstructive and Hand Surgery
  ^7^  Amsterdam Movement Sciences (AMS) Institute, Amsterdam UMC
  ^8^  Burn Center and Department of Plastic and Reconstructive Surgery
  ^9^  Amsterdam UMC Location University of Amsterdam, Paediatric Surgical Centre, Emma Children’s Hospital
  ^10^  Association of Dutch Burn Centers, Burn Center Martini Hospital Groningen
  ^11^  Hanze University of Applied Sciences Groningen, Research Group Healthy Ageing, Allied Health Care and Nursing
  ^12^  Department of Human Movement Sciences, University Medical Center Groningen, University of Groningen
  ^13^  Department of Surgery, Red Cross Hospital
  ^14^  Department of Surgery, Martini Hospital
  ^15^  Trauma Research Unit Department of Surgery, University Medical Center Rotterdam

**Objectives**: The optimal care of intermediate burn wounds remains a topic of debate. Generally, there are two treatment strategies (1) early skin grafting (within 7 days postburn) or (2) conservative antimicrobial topical treatment with grafting after 14–21 days for the remaining defects, if needed. These strategies have pros and cons with little known about patient-relevant outcomes. It is important to take the patients’ preferences into consideration while making the treatment decision in addition to clinical necessity. Therefore, we aimed to gain insight into patient-relevant outcomes and (cost)effectiveness of both treatment strategies for patients with intermediate burn wounds.
**Methods**: A core set of process indicators, outcomes and cost components (value-based healthcare (VBHC)-burns core set) will be developed. This will be conducted by identifying current process indicators and outcomes in a metadata catalogue, identifying important patient outcomes by questionnaires, and reaching a consensus by a modified Delphi Method finalizing the core set. Next, our current registries will be updated according to the VBHC-burns core set. Subsequently, the core set will be used in the cluster cross-over study, conducted in the three Dutch burn centres. during a period of nine months, patients with intermediate burns (i.e., wounds with an expected healing potential of 14–21 days, according to a laser Doppler image) receive conservative treatment with surgery if needed. during the following nine months, the treatment strategy in patients will be early skin grafting within 7 days. Outcomes of both strategies will be analysed and incorporated into a—to be developed—decision aid. An iterative process with patients and healthcare providers will finalize the decisions aid that aims at supporting shared decision-making in clinical practice.
**Results**: We expect to include approximately 300 patients with intermediate burn wounds in each cluster of nine months. These patients will be followed for one year. The first cluster starts at 1 June 2022. The VBHC-burns core set will then be available to measure process indicators, outcomes and societal cost of both strategies. Primarily, short-term outcomes from cluster one will be presented.
**Conclusions**: This unique cluster cross-over study will provide insights into patient-relevant outcomes in the treatment decision of intermediate depth burns. The findings will inform patients and healthcare providers, serve as a practical guide to understand and prioritize the preferences and goals of patients, and support the involvement of patients in the treatment decision. Ultimately, this will lead to optimized person-centred acute burn care and improved patient-relevant outcomes while maintaining or lowering costs.

*O.005*

**Functional and Aesthetic Outcome of Acute III° Burn Injuries to the Dorsum of the Hand after Free Flap Transfer**

**Simon Kuepper, Bernd Hartmann and Jenny E. Dornberger**
  BG Unfallkrankenhaus

**Objective**: Complex burns to the hand are challenging in terms of reconstructive coverage, because of the sophisticated anatomy of the upper extremity and the need to preserve as much function as possible. Special caution is required regarding sufficient debridement of devitalised tissue and a potentially increased risk of infection in the acute setting. We evaluate the outcome of seven patients over the last three years that needed acute free flap coverage. The research objectives were indication, timing, principles for flap selection, complications, flap survival rate and outcome.
**Methods**: From January 2019 to January 2022, seven patients with deep burn wounds to the dorsum of the hand were admitted to the Department of Burns and Plastic Surgery of BG Trauma Center of Berlin, Germany. The affected patients included four males and three females, aged 2–58 years. Five of them had a work-related injury involving their dominant hand. Two patients sustained a high-voltage injury. after debridement, the defect size ranged between 3.0 cm × 2.5 cm and 9.0 cm × 6.0 cm. In three patients, thermal damage not only affected the skin, but at least one of the extensor tendons.
**Results**: Reconstruction with cutaneous or fascia flaps within 3 weeks post-trauma was the preferred method. Defects were covered with fascia flaps in four cases, either serratus or temporal fascia. The remaining three received a free ALT flap combined with a tendon reconstruction and, if necessary, also reconstruction of the paratenon. All flaps survived; we observed one partial flap failure in a serratus fascia flap. after 6–12 weeks of focused occupational hand therapy during outpatient care, five out of seven patients showed a good-to-very-good hand function. The functionality of the hand was assessed before and after the 8 weeks of occupational therapy using the DASH questionnaire. Patients with tendon repair showed a lesser functional result. One patient had to be revised with arthrodesis in two PIP joints. All but one patient expressed satisfaction with the aesthetic result.
**Conclusions**: Our data suggest that early coverage of hand burns and even high-voltage injuries to the hand require custom-tailored reconstructive solutions for limb salvage und function preservation. Cases with required tendon repair are regarded as demanding extensive early reconstruction to preserve the best possible hand function. Fascia flaps and thin ALT-Flaps prove to have a good functional and aesthetic outcome and are reliable and suitable for the coverage of the dorsum of the hand.

*O.006*

**The Versatility of Perifascial Loose Areolar Tissue Grafting in Extremity Burns**

**Burak Ozkan ^1^, Cagri A. Uysal ^1^ and Mehmet Haberal ^2^**
  ^1^  Department of Plastic, Reconstructive and Aesthetic Surgery, Baskent University Faculty of Medicine
  ^2^  Department of General Surgery and Burn Center, Baskent University Faculty of Medicine

**Introduction**: Perifascial areolar tissue (PAT) is an areolar layer over muscle fascia. It has been shown that PAT is resistant to ischemia and prone to survive even in ischemic conditions. PAT grafts provide a vascular tissue layer on necrotic bone and tendons where skin grafting cannot be possible. The effect of PAT grafting in burn reconstruction has not been reported yet. We aim to present our experience and discuss the role of PAT grafting in extremity burns.
**Materials and Methods**: Between January 2019 and December 2020, 16 PAT graftings were performed in 11 patients. All patients had deep full thickness burns in their upper and lower extremities with exposed bones or tendons. PAT grafts were harvested from the abdominal region. PAT grafts were utilized to the upper extremity in 7 patients and the lower extremity in 4 patients. Immediate skin grafting was performed during the same session.
**Results**: The mean age of the patients was 50.7. The mean defect size was 3.3 × 3 cm^2^. The mean follow-up time was 11.8 months. The survival rates of PAT and skin grafts were 93.8 and 68.6%, respectively. Partial skin graft losses were encountered in 4 patients and total skin graft loss was seen in 1 patient.
  **Conclusions**: PAT grafting is an alternative method to dermal substitutes and flap surgery in small-to-medium-sized defects with bone and tendon exposure in burn patients.

*O.007*

**How to Implement Aromatherapy on a Burn Centre**

**Anneke Melissant and Helma Hofland**
  Maasstadziekenhuis, Rotterdam, The Netherlands

**Objectives**: Burn patients experience stress and pain during wound care despite pharmacological interventions. Non-pharmacological interventions such as breathing techniques, music, social talk, virtual reality, massage and aromatherapy are described to help the patient to relax and distract from pain during procedures, as well as for relaxation afterwards. These interventions can be used in combination or alone. The choice of intervention is determined by individual preferences and the situation. Many non-pharmacological interventions are used in burn care, but aromatherapy is not widely used. The aim is to identify the obstacles nurses might have when implementing this intervention.
**Method**: Aromatherapy is a treatment that uses scents and oils to enhance health and wellbeing. By stimulating the limbic system with soothing or uplifting scents, it initiates all kinds of processes to calm the body and mind. Essential elements in the implementation are training, obtaining material and willingness to use the intervention by the team members.
  1. Training: A qualified course is necessary to implement and instruct colleagues how and when to start with aromatherapy on the ward. after the training the nurse can experiment, with patients’ consent, on the use of oils as inhalation or with massage.
  2. Material: Money from sponsors can be used to buy diffusers and oils to begin with. Once the organization realizes that aromatherapy is essential, money is budgeted to continue with this intervention. Blends of several oils can be made by the nurse depending on the needs of the patients (sleep, focus, gain control, relaxation, etc.).
  3. Implementation: In 2017 a dedicated nurse started a one-year training program. Actions to implement aromatherapy followed during this training, including instruction lessons on the ward to create awareness, folder with non-pharmacological interventions including demonstrations how to use aromatherapy. after the course in 2019 a small core team of interested nurses was initiated and trained by this nurse.

**Results**: Since 2019, this intervention can be offered to patients on a regular base. All nurses on the ward are able to use diffusers. The enthusiastic core team is essential to guarantee continuity.
Two cases showing how aromatherapy was initiated into the daily routine in the burn centre will be discussed.
**Conclusions**: Aromatherapy gives a personal holistic care and can be used individually or in combination with other non-pharmacological interventions. Nurses need additional training and enthusiasm to implement aromatherapy into the daily routine of the burn centre.

*O.008*

**Which Were the Effects of Restrictive Visits during COVID Pandemic to Severely Burned Patients, Their Relatives and Medic Staff?**

**Maluisa Pérez Del Prado, Pol Miguel Puigbarraca, Laura Garcia Gómez and Mònica Soler Rovira**
  Vall d’Hebron, Barcelona, Spain

**Objective**: To create a research project to determine the effects of restrictive visits due to the COVID pandemic on severely burned patients, their relatives and medic staff to show off the drastic contingency plans applied during this pandemic. The imposition of restrictive visits represented a setback to the paternalistic paradigm, focused on physical and technification care, relegating dangerously psycho-emotional well-being to second place. These measures had direct consequences on the advances in quality care made by humanization in recent years. Severely burned patients had to face one of the greatest traumas that can occur, with significant physical and psychological suffering, while away from their relatives. In turn, their relatives had to cope with such an overwhelming situation, apart from their loved ones, and with increasing emotional desolation and delegation of the responsibility of the caregiving role to the health professionals.
**Methods**: To develop a research project with quantitative and retrospective methodology at Vall d’Hebron’s Hospital Burns Unit in Barcelona that analyses how these restrictive measures affected severely burned patients, their relatives and medical staff, as well to consider the convenience and proportionality of these measures and determinate the risks and benefits that this caused in the short and medium term.
**Results**: There are large publications that show off the reverse effects of the restrictive measures applied especially in intensive care units, but there is little information related to the burned patients. The publications recommend expanding research to determine the true scope of these measures, as well as to create visit protocols that integrate the specific needs of burned patients.
**Conclusions**: Being able to execute this research project will allow us to know how burned patients, their relatives and medical staff experienced such devastating events deprived of their loved ones, and it will help us spotlight the proportionality and convenience of the measures.

*O.009*

**Evaluation of a Brief Burn-Specific Screening Instrument**

**Helma Hofland ^1,2^, Anita Boekelaar ^3^, Anneke van de Steenoven ^1^ and Nancy van Loey ^1,2^**
  ^1^  Maasstadhospital
  ^2^  Association of Dutch Burn Centres
  ^3^  Rode Kruis Ziekenhuis

**Objectives**: Burn survivors may face a variety of physical and psychological problems after their burn injury. As a consequence, supportive needs may differ across burn survivors. However, in outpatient settings, time constraints may hamper in-depth conversations to identify physical and particularly psychosocial concerns. Therefore, the aim of this study was to develop and evaluate a screening instrument that may inform clinicians about supportive needs.
**Methods**: Adult patients attending the outpatient clinic were asked to complete the instrument which comprises a distress thermometer (DT) and a burn-specific problem list (BS-PL). The BS-PL has 44 items divided into 9 domains: physical concerns, functional concerns, skin-therapy-related concerns, body-related concerns, stigmatization, intimacy and sexuality, psychological problems, social concerns and positive coping. Answers are scored yes/no. Additionally, burn survivors were asked whether they wanted to discuss their concerns. Correlational analyses were performed to test the adequacy of the items in relation to the level of distress. Acceptability to completers was qualitatively tested.
**Results**: One hundred two adult patients visiting the outpatient clinic completed the DT and BS-PL. All items were used. The mean distress score was 3.92. Thirty-three burn survivors wanted to talk about their concerns with a professional; they reported higher distress scores (M = 5.97 versus M = 2.95 for those who did not want to) and they reported more frequent problems with pain, itch, fatigue and a range of psychological problems. Correlations between the DT and the BS-PL domains varied. No significant correlations between items of positive coping, skin-therapy-related concerns and social problems were observed. A cutoff point of ≥6 was suggested to indicate higher distress. Acceptability to burn survivors was high.
**Conclusions**: These preliminary results show that the DT and BS-PL are acceptable to burn survivors, contains relevant items and can be used as a screening instrument to detect possible supportive needs.

*O.010*

**Completing the Toolbox of Pain Measures: A Multicenter Clinimetric Study on Pain Measurement in Critically Ill Adults with Burns**

**Alette De Jong ^1^, Wim Tuinebreijer ^2^, Helma Hofland ^3^, Jetty Meyer ^4^ and Nancy van Loey ^3^**
  ^1^  Red Cross Hospital
  ^2^  Erasmus MC, Trauma Research Unit
  ^3^  Association of Dutch Burn Centres, Maasstad Hospital
  ^4^  Martini Hospital

**Objectives**: Pain measurement enables the evaluation of pain interventions and leads to the improvement of individualized pain treatment. There is a need for appropriate pain measurement instruments to assess pain in critically ill adults with burns who are unable to provide self-reports. The aim of this study is to investigate whether the Behaviour Pain Scale (BPS) and the Critical Pain Observation Tool (CPOT) are valid, reliable and clinical useful instruments to measure pain behaviour in critically ill adults with burns.
**Methods**: We performed a multicentre observational cohort study with repeated measurements. Pain was assessed by BPS and CPOT, three times a day, once during wound care and twice during periods of rest, by two trained burns and/or ICU nurses who observed patients simultaneously. A questionnaire was used to survey clinical usefulness of the scales. Classical test theory according to the COSMIN guideline was applied to assess the clinimetric properties.
**Results**: In total, 132 nurses rated 75 patients, which yielded a total of 1040 paired observations. The majority (63–87%) of nurses indicated that BPS and CPOT reflect background and procedural pain-specific features. Principal component factor analysis showed that all BPS and CPOT items were loaded on one latent factor (≥0.81). Spearman’s rho correlation between BPS and CPOT was 0.79 for background and 0.83 for procedural pain, meeting the criterion of ≥0.70. Internal consistency by Cronbach’s alpha met the criterion of ≥0.70 ≤0.95 for both types of pain, ventilated and non-ventilated patients, for both scales, except for non-ventilated patients observed by BPS (0.67). Cronbach’s alphas for CPOT were higher than for BPS. Intraclass correlation coefficients (ICC) of the total scores were sufficient (≥0.70) for both scales, but ICCs were insufficient in patients with facial burns (0.63–0.66). A Wilcoxon signed ranks test showed no statistically significant difference between background and procedural pain behaviour scores for both scales. The majority of the nurses indicated that the scales are short and easy to administer and are clear and easy to understand. Due to low median total scores, and thus a lack of data variation, but suggesting that pain seems well controlled, we were unable to calculate cut-off scores.
**Conclusions**: Both BPS and CPOT are valid and reliable scales for the measurement of pain in ICU patients with burns. They seem to measure one construct and show sufficient inter-rater agreement. The scales are, however, not able to distinguish background from procedural pain.

*O.011*

**The Role of Nurses in a Non-Profit Organization: Cute Project Experience**

**Enzo Amelio, Daniele Bollero, Dorina Caldarescu, Martina Canata, Paola Curto, Alessia Desantis, Vincenzo Lo Vermi, Samantha Marocco, Eva Mesturino and Loredana Silvestro**
  Cute Project

**Objectives**: Nurses are one of the key figures in the accomplishment of the Cute Project’s mission. The Cute Project is a non-profit organization that works at improving the quality of burn treatment and preventing burn injuries. Thanks to this program, nurses can express their potential both in Italy and through humanitarian aid.
**Methods**: Nurses from the Cute Project teach about burn injuries and how to prevent them in Italian schools and in rural Congo, Uganda and Benin. In these countries, they offer high-quality assistance in the operating room, and they train members of the local staff in the care of adult and young patients. Furthermore, they collaborate with the doctors and surgeons of Cute Project, with the help of Sermig of Turin, in outpatient service, to ensure care to people who have difficulty accessing to the Italian Health Service.
**Results**: Since 2016, 4042 children have taken part in that preventive program through interactive workshops in primary schools and kindergartens in Italy. Nurses have also led educational and preventive activities aimed at children in Congo, Uganda, and Benin. In these countries, the Cute Project’s scrub nurses have worked in the operating room, trained, and supervised the local scrub nurses and health workers and operated on up to 1000 patients from 2013 to 2019. They have also given theoretical lessons about nursing care to the local staff.
When it comes to plastic surgery, in Turin, nurses and surgeons of the Cute Project examined 168 outpatients between 2014 and 2020.
**Conclusions**: Inside a non-profit organization, nurses can play a decisive role. The nature of their job (educational, technical, relational) makes them essential to the fulfillment of the mission. The Cute Project gives the opportunity to nurses to explore and develop their skills in every field possible.

*O.012*

**Setting the Scene: Simulation Training for Burn Nurses**

**Susan Molloy, Maria Wormmeester, Leo Bolhuis, Hans Eshuis, Tineke Kingma, Helma Kramer, Gerbrig Bijker, Marianne Nieuwenhuis and Aagtje Mekkering**
  Martini Ziekenhuis

**Objectives**: Admissions of patients with burns are acute, mostly infrequent, varying from small to life-threatening burns. This has ramifications for the expertise and proficiency of nursing staff. Long intervals can elapse between admission experiences, which in turn can cause stress. All our nurses have completed the Emergency Management of Severe Burns course; however, without follow-up, competency stagnates. To ensure their optimal admission response, we have, since 2013, introduced Simulation training. This training is an on-going process and one which demands our continuous attention. Retrospective evaluation of recorded simulations and instructors feedback revealed room for improvement. Can we improve it and if so, how? Additionally, how can we measure this improvement?
**Methods**: Simulation training is held annually in the center’s admission room, involving all burn intensive care and general burn nurses. As per the training session, three to four nurses re-enact an acute admission according to the ABCDE method with a professional simulation actor and under the guidance of trained Crisis Resource Management (CRM) burn nurses. An extensive debriefing follows according to the CRM structure. Currently, preparation for the candidates consists of written and oral instruction. In aid thereof, we have created an instruction film of an exemplary simulation where both technical and non-technical aspects are clearly demonstrated, as proposed by Esher et al., [1]. Furthermore, the introduction of CRM to the training demanded a better scoring system than we were already using. A literature search did not provide a scoring system which fulfilled all our wishes. The closest was the TEAM scoring system Cooper et al., [2], which we adapted (TEAM Adapted to Burns (TATB)). Nurses will be shown the instruction film at the beginning of the simulation training day. All simulations are filmed and will consequently be assessed using the TATB for m.
**Results**: Around 50 nurses participate annually. They exhibit a high competency in technical skills, but we see room for improvement, especially in: Leadership, Communication and Methodology. The effect of the simulation and impact of the addition of the instruction film will be evaluated.
**Conclusions**: We have learnt through literature studies, feedback and observation of the participants, to create an optimal learning environment and have adapted our training accordingly. Developing a Simulation training is a learning curve for all involved. We have taken many steps for ward since commencement nine years ago, and wish to share our experiences with more burn professionals.

**References**
  1. Escher, C.; Rystedt, H.; Creutzfeldt, J.; Meurling, L.; Nyström, S.; Dahlberg, J.; Edelbring, S.; Amorøe, T.N.; Hult, H.; Felländer-Tsai, L.; et al. Method matters: Impact of in-scenario instruction on simulation-based teamwork training. *Adv. Simul.* **2017**, *2*, 25. PMID: 29450026.
  2. Cooper, S.; Cant, R.; Porter, J.; Sellick, K.; Somers, G.; Kinsman, L.; Nestel, D. Rating medical emergency teamwork performance: Development of the Team Emergency Assessment Measure (TEAM). *Resuscitation* **2010**, *81*, 446–452. PMID: 20117874.

*O.013*

**Nutritional Impact Symptoms in Patients 6 Months After Minor Burn Injury—A Questionnaire Survey**
**Josefin Dimander ^1^, Fredrik Huss ^1^, Adriana Miclescu ^1^, Agneta Andersson ^2^ and Catarina Lindqvist ^3^**
  ^1^  Uppsala University Hospital
  ^2^  Uppsala University
  ^3^  Karolinska Institute

**Objectives**: To examine the prevalence of nutritional impact symptoms (NIS) 6 months after a minor burn injury.
**Methods**: Patients admitted to the Burn Centre, and ≥18 years old, were asked to participate in the study during their regular follow-up at 6 months post burn injury. Nutritional impact symptoms were examined with two modified and content validated questionnaires, the eating symptom questionnaire (ESQ-burn), and disease-related appetite questionnaire (DRAQ-burn). Information on the burn injury and medical treatment including nutritional treatment and patients´ demographics were collected.
**Results**: A total of 18 patients were recruited for the study. The mean age was 58.0 (SD 18.7) years, 10 male (56%) and 8 women (44%). The mean total burn surface area was 8.6% (SD 9.6) Six months after injury, the mean body weight change was +0.8 kg (SD 4.2). during the first two weeks post burn injury, nutritional treatment with parenteral nutrition (*n* = 2, 11%), enteral nutrition (*n* = 2, 11%), oral nutritional supplements (*n* = 9, 50%), and protein enrichment (*n* = 3, 17%) had been provided to participants. In total, 9 of the participants (50%) had contact with a dietitian after injury.
In ESQ, the majority of patients (*n* = 11, 61%) reported 1–4 mild to moderate symptoms of NIS, where “tiredness/fatigue affecting appetite” were the most commonly reported NIS (*n* = 7, 39%), followed by “pain or ache affecting my appetite” (*n* = 6, 33%), and thereafter “nausea” (*n* = 5, 28%) and “tiredness/fatigue preventing me from eating” (*n* = 5, 28%).
In DRAQ, some patients reported that the injury had affected their appetite (*n* = 6, 33%), and that they never or rarely felt hungry (*n* = 5, 28%). Some participants depended on someone else to prepare their meals because of the burn injury (*n* = 5, 28%).
**Conclusions**: We need to pay attention to NIS during follow-ups post burn injury. Considering that participants in this study have minor burns, surprisingly, many have reported nutritional impact symptoms 6 months after injury. If and how this effects nutritional intake and status and frequency of NIS after major burns warrants further investigation.

*O.014*

**Do We Apply the Appropriate Measures to Prevent and Treat Delirium in Severely Burned Patients? Review of Actions Carried out by Nursing Staff to the Management of Delirium**

**Pol Miguel Puigbarraca, Marisa Pérez del Prado, Laura Garcia Gómez and Mònica Soler Rovira**
  Vall d’Hebron Hospital

**Objective**: To carry out a bibliographic review on nursing measures applied in critical units to prevent and treat delirium and check if these are applied at Vall d’Hebron’s Burns Unit in Barcelona.
**Method**: Through a bibliographic review of quantitative and qualitative articles and systematic reviews to the last 5 years where strategies developed by nursing staff to the optimal management of delirium in patients older than 18 years in burn units and critical patient units are described.
**Results**: The consulted bibliography identifies that the following strategies are the most accepted to prevent and treat delirium: assessment and stimulation of cognitive status, promotion of early mobilization, adaptation of audition and vision, promotion of night rest, maintenance of hydration and review of medication.
**Conclusions**: Generally, all the measures to prevent and treat delirium found in the bibliography consulted are applied by the nursing staff at Vall d’Hebron’s Burns Unit. Even so, there is currently no protocol at this unit to the standardization and register of these actions, a fact that would facilitate the early detection and treatment of this syndrome.

*O.015*

**High-Voltage Electrical Injury: A 4-Year Retrospective Analysis of 145 Cases at a Burn Center in Tunisia**

**Amel Mokline, Hana Fredj, Houdeifa Hermassi, Wala Brahmi, Manel Ben Saad, Bahija Gasri, Imen Jemi and Amen Allah Messadi**
  Intensive Burn Care Department, Tunis, Tunisia

**Objective**: High-voltage electrical (HVE) injuries cause significant morbidity and mortality despite relatively small burn sizes.
The objective of our study was to evaluate epidemiological profile, clinical data, and outcomes of electrical injuries at a major burn center in Tunisia.
**Methods**: Retrospective descriptive study enrolled consecutive High-voltage electrical burn patients admitted to the burn center in Tunisia between March 2017 to December 2021.
**Results**: during the study period, 1918 patients were admitted, among which 182 were victims of HVE injuries (9%). In total, 145 were included. The age of patients was 35 ± 12 years with a masculine predominance (97%). In our series, 15% were children (*n* = 21). Most burns occurred in industrial workers and electricians at work (67% of cases (*n* = 96), followed by or householders at home (25.4%). TBSA was 18 ± 1%. Rhabdomyolysis was noted in 87% of cases, with a level of CPK at 18243 U/L. Acute renal failure occurred in 13% of cases. Myocardial injury occurred in 100 patients (68%). Electrocardiogram was performed in half of the patients. Abnormalities were noted in 68 of cases: Sinus tachycardia (36%, *n* = 28) and atrial fibrillation de novo occurred in 2 cases. Compartment syndrome was noted in 35 patients (24%) requiring escharotomy. Excision of necrotic tissues was done in 40% of cases. Amputations were performed in 25% of cases. Venous thromboembolic complication was observed in 15% of cases. Sepsis occurred in 68 patients (46%). The average ICU stay of patients was 22 ± 3 days. Mortality was 17% (*n* = 25) due, essentially, to refractory septic shock.
**Conclusions**: High-voltage injuries were associated with more extensive burns, longer ICU stays, and more complications and amputations.

*O.016*

**Does Optimizing Prophylactic Anticoagulation in Burns Reduce Venous Thromboembolism Complications?**

**Amel Mokline, Racha Ghabara, Hana Fredj, Manel Ben Saad, Imen Jemi, Bahija Gasri, Hana Ben Ali and Amen Allah Messadi**
  Intensive Burn Care Department, Tunis, Tunisia

**Objective**: Assess the impact of optimizing prophylactic anticoagulation with enoxaparin in burn patients on the incidence of venous thromboembolism (VTE).
**Methods**: Prospective, evaluative, case–control study conducted in intensive burn care department in Tunisia for 24 months, (February 2018–February 2020). Patients were divided into two groups according to the prophylactic anticoagulation modalities:
  − G1 (Equation) receiving enoxaparin en mg/12H = 22.8 + (3.3 ×% SCB/10) + (1.89 × (weight in kg)/10));
  − G2 (No equation) receiving enoxaparin at a single dose of 1 mg/weight.
The goal of prophylactic antifactor Xa level was 0.2–0.4 IU/mL.
**Results**: during the study period, 216 patients were included, divided into two groups: G1 (*n* = 108) et G2 (*n* = 108). The groups were comparable in terms of sex, age, weight, burned skin surface and VTE risk. Additionally, severity of the 2 groups was comparable regarding: smoke inhalation (*p* = 0.46), use of mechanical ventilation (*p* = 0.22), use of catecholamines within 48 h (*p* = 0.56) and rescue incision (*p* = 0.77). In the equation group, initial dose of enoxaparin was 0.42 ± 0.12 mg. Target anti Xa was reached at the 1st dosage in 55 patients 55 (50.9%). The median final dose of enoxaparin required to reach the anti Xa target was 52 mg every 12 h (range, 35–69 mg). No episodes of bleeding, thrombocytopenia, or heparin allergy were documented in either group.
The incidence of VTE complications was higher in group 2 than in group 1 (9% versus 4%; *p* = 0.001 with an OR = 1.6 and CI [0.47–1.03]). The length of ICU stay was longer for G2 with a significant difference (30 days vs. 22 days; *p* = 0.001). Mortality was the same for the two groups.
**Conclusions**: Optimizing thromboprophylaxis in severely burned patients with enoxaparin, using the enoxaparin dosing equation, allows to achieve prophylactic anti-Xa level and to reduce risk of VTE complications.

*O.017*

**Vasoactive and/or Inotropic Drugs in Initial Resuscitation of Burn Injuries: A Systematic Review**

**Kristine Knappskog ^1,2^, Nina Gjerde Andersen ^3^, Anne Berit Guttormsen ^1,4^, Henning Onarheim ^1,4^, Stian Kreken Almeland ^1,2^ and Sigrid Beitland ^5^**
  ^1^  Department of Clinical Medicine, Faculty of Medicine, University of Bergen
  ^2^  Department of Plastic, Hand and Reconstructive Surgery, Norwegian National Burn Center, Haukeland University Hospital
  ^3^  Department of Anaesthesiology, Division of Emergencies and Critical Care, Oslo University Hospital
  ^4^  Department of Anaesthesia and Intensive Care, Haukeland University Hospital
  ^5^  Specialised Health Care services, Quality and Clinical Pathways, Norwegian Directorate of Health

**Objectives**: According to current guidelines, initial burn resuscitation should be performed with fluids alone. The aims of the study were to review the frequency of use of vasoactive and/or inotropic drugs in initial burn resuscitation and to assess the benefits and harms of adding such drugs to fluids.
**Methods**: A systematic literature search was conducted in PubMed, Embase, Cochrane Database of Systematic Reviews, Cochrane Central Register of Controlled Trials, UpToDate, and SveMed+ through 3 December 2021. The search included studies on critically ill burn patients receiving vasoactive and/or inotropic drugs in addition to fluids within 48 h after burn injury.
**Results**: The literature search identified 1058 unique publications that were screened for inclusion. after assessing 115 publications in full text, only two retrospective cohort studies were included. One study found that 16 out of 52 (31%) patients received vasopressor(s). Factors associated with vasopressor use were increasing age, burn depth and% total body surface area (TBSA) burned. Another study observed that 20 out of 111 (18%) patients received vasopressor(s). Vasopressor use was associated with increasing age, Baux score and%TBSA burned in addition to more frequent dialysis treatment and increased mortality. Study quality assessed by the Newcastle-Ottawa quality assessment scale was considered good in one study, but uncertain due to the limited description of methods in the other.
**Conclusions**: This systematic review revealed that there is a lack of evidence regarding the benefits and harms of using vasoactive and/or inotropic drugs in addition to fluids during early resuscitation of patients with major burns. There is a need for more data to decide whether vasoactive and/or inotropic drugs should be added to fluids or not. There seems to be a discrepancy between treatment guidelines suggesting use of fluid alone, and clinical practice studied in surveys indicating that vasoactive and/or inotropic drugs are frequently used.

*O.018*

**Utility of Partial Pressure of Venous to Arterial Carbon Dioxide Gradient: Guide and Prognostic Value in Critically Burn Patients**

**Javier Vejo Gutiérrez, Manuel Sánchez Sánchez, Irene Seises García, Alba López Fernández, Jesús Soto Gómez-Cambronero, Raquel Yébenes Calvo, Javier Rábano Alonso, Eva María Flores Cabeza, Pablo Millán Estañ and Carola Gutiérrez Melón**
  Hospital Universitario La Paz, Madrid, Spain

**Objectives**: The aim of this study is to analyse the relation between hemodynamic parameters and analytic indicators of hypoperfusion such as Pv-aCO_2_ gap and lactate during early resuscitation in severely burn patients and to analyse its correlation with severity scores in order to evaluate its prognostic value according to standardized severity scores (APACHE II and SOFA score).
**Methods**: We did a prospective observation in patients admitted to the Burn Intensive Care Unit from June 2018 to May 2021 with more than 30% of total body surface area (TBSA) burned. Pv-aCO_2_, lactate levels and hemodynamic measurements were performed during the period of admission and at least for 72 h during the initial resuscitation. APACHE II score was registered as well as SOFA score at the moment of admission and at h 24, 48 and 72 since burning time. We considered statistical significance with *p* < 0.05 and used Spearman’s test, simple regression, and Kruskal–Wallis test.
**Results**: In the study period, a number of 27 patients were recruited, 23 of whom (83.2%) were men and 4 (14.8%) women. Mean age was 51 ± 16 years and TBSA 41.81 ± 18%. The main burning mechanism was flame (85%), and the mean Baux index was 92 ± 20. Mean Pv-aCO_2_ at h 12 and 16 were 12 ± 10 and 9 ± 5, respectively, and those were statistically different with mean Pv-aCO_2_ at hour 36 (7.6 ± 12). The correlation between cardiac index and lactate levels was r = −0.42 (*p* = <0.001) and between cardiac index and Pv-aCO_2_ was r = −0.32 (*p* = <0.001). Regarding severity scores, the correlation between Pv-aCO_2_ with Baux index, APACHE II and SOFA scores at h 24, 48 and 72 was not significant, whereas there is a tendency in correlation between Pv-aCO_2_ and SOFA scores at admission (r = 0.4.4, *p* = 0.1).
**Conclusions**: The Pv-aCO_2_ gap is modified in the early resuscitation of severely burn patients. Hemodynamic situation of this group of patients may be evaluated indistinctly with lactate levels or Pv-aCO_2_ gap because of their good correlation with the cardiac index in early resuscitation. The Pv-aCO_2_ gap shows scarce correlation with the SOFA score at admission. However, it is not a good severity indicator at admission, and it cannot be used as a prognostic value because of the lack of correlation with standardised severity scores.

*O.019*

**Initial Albuminemia and Albumin Administration in Severely Burned Patients: Do They Matter?**

**Christoph Ils ^1^, Mette M. Berger ^2^, Anthony De Buys Roessingh ^1,3^, Christelle Jung ^3^, Dan Carel ^2^ and Olivier Pantet ^1,2^**
  ^1^  University of Lausanne
  ^2^  Service of Intensive Care Medicine, Lausanne University Hospital
  ^3^  Children and Adolescent Surgery Service, Lausanne University Hospital

**Objectives**: during burn shock, the early use of albumin has always been debated. We hypothesized that early hypoalbuminemia was an independent risk factor for mortality and that early albumin administration was associated with a decrease in mortality, acute kidney injury (AKI), dialysis and fluid requirements.
**Methods**: This was a retrospective, single-center study conducted in the burn intensive care unit of Lausanne University Hospital between 1 January 2006 and 31 December 2018. The inclusion criteria were age ≥14 years and burns >20% total body surface area (TBSA). The exclusion criteria were admission >8h after burn accident, transfer in the first week to another burn unit or withdrawal of therapy during the first 72h. Data were presented as median (IQR) or number (%).
**Results**: In total, 141 patients were included, with burns 35% (24% to 50%) TBSA, age 39 (26–56) years, 68.1% were male and 56.7% had inhalation injuries. The ABSI score was 8 (7–10). In total, 17 (12%) patients died. Minimal albuminemia in the first 24h was lower in non-survivors (15 (14–20) vs. 24 (19–31) g/L; *p* < 0.001) and was found to be an independent risk factor for mortality when adjusted for ABSI (*p* < 0.001) with a best cut-off of 22 g/L to predict mortality (sensitivity 63.6%; specificity 87.5%). In univariate analysis, albumin 20% was administered more often to non-survivors (12 (71%) vs. 30 (24%); *p* < 0.001), in increased quantities (47 (25–58) vs. 30 (10–40) g; *p* = 0.030) and earlier (9 (5–13) vs. 18 (14–20) h; *p* < 0.001). AKI was more frequent in albumin recipients (29 (69%) vs. 33 (33%); *p* < 0.001). The logistic regression model adjusted for ABSI found that albumin administration increased the risk of developing AKI in the first 7 days (OR 1.03 (95% CI 1.00–1.05); *p* = 0.035), without any significant effect on mortality or fluid requirements. Increased quantities of albumin administered in the first 7 days were associated with higher cumulated fluid balance, even after adjustment for TBSA.
**Conclusions**: Hypoalbuminemia <22 g/L in the first 24h was found to be an independent risk factor for mortality. Albumin administration was associated with an increase in AKI, without significant reduction in fluid requirement at 24h, but with an increase in fluid balance on day 7. Our study was underpowered to draw conclusions on mortality and dialysis. In the absence of a prospective study clearly demonstrating a benefit of albumin, its use should remain confined to extreme hypoalbuminemia

*O.020*

**Burns Mortality Prediction Models Comparative Study in a Portuguese Burn Centre: 5-Year Study**

**Gonçalo Ferreira, José Miguel Azevedo, Dr. Dmitry Shelepenko, Inês Catalão and Luís Cabral**
  Plastic, Reconstructive Surgery and Burns Unit Department, Coimbra Hospital and University Centre

**Objectives**: This study aims to evaluate and compare different validated mortality prediction models performance in our burns unit (BU) and compare survivors’ and non-survivors’ associated characteristics.
**Methods**: We have conducted a retrospective study of adult burn patients admitted to our BU between 2017 and 2021 (5-year period). Polytraumatized and toxic epidermal necrolysis patients were excluded. Mortality was assessed. Survivors’ and non-survivors’ clinical and demographic characteristics were analyzed. The four models included were the Abbreviated Burn Severity Index (ABSI), Belgian Outcome in Burn Injury (BOBI), revised Baux and Ryan model. Predicted and observed mortality were compared with the Hosmer-Lemeshow test for the models’ goodness-of-fit, receiver operating curves (ROC) and area under curve (AUC) for discriminative performance evaluation.
**Results**: A total of 641 patients were included and 58,2% were male. Patients’ median age was 62 years and the median total burned surface area (TBSA) was 8%. Third-degree burns were present in 71% and inhalation injury in 12.3%. The observed mortality rate was 9.4% (*n* = 60). Non-survivors were significantly older (73 vs. 60 years; *p* < 0.001), had a larger TBSA (27.75 vs. 7%; *p* < 0.001), higher frequency of third-degree burns (96.7 vs. 68.3%; *p* < 0.001) and inhalation injury (31.7% vs. 10.3%; *p* < 0.001), but no significant difference in gender. All models showed adequate goodness of fit with *p*-values >0.05 in the Hosmer–Lemeshow test assessment. Revised Baux (AUC 0.870 ± 0.025), ABSI (AUC 0.850 ± 0.026) and BOBI (AUC 0.831 ± 0.026) have demonstrated good discriminative power, and the Ryan model (0.774 ± 0.030) was only moderate.
**Conclusions**: The four scoring models revealed proper predictive performance, with revised Baux presenting as the most accurate model for mortality prediction. Their use in the BU represents a practical and valuable tool for risk stratification, treatment appropriateness and improve the quality control of burn care.

*O.021*

**Acute Kidney Injury in Septic Burn Patients: Incidence and Outcome**

**Eirini Nikolaidou ^1^, Theodora Ligomenou ^1^, Argyro Pipinia ^1^, Zoi Tzimorota ^1^, Sophia Papadopoulou ^1^, Athina Lavrentieva ^2^ and Militsa Bitzani ^2^**
  ^1^  Plastic Surgery Department and Burn ICU, Papanikolaou Hospital
  ^2^  A-ICU, Burn ICU, Papanikolaou Hospital

**Introduction**: Acute kidney injury (AKI) is a common complication in burn patients admitted to the intensive care unit (ICU) associated with increased morbidity and mortality. Sepsis-Associated AKI (SA-AKI) account for the majority of cases of AKI in the ICU with poor prognosis. Our primary aim was to review the incidence and outcome of AKI in septic burn patients admitted to the Burn ICU.
**Materials and Methods**: Design, setting, and patients: Observational study of burn patients who were admitted to a Burn ICU and fulfilled the criteria for sepsis and AKI. Main outcome measures: Occurrence of acute kidney injury, factors contributing to etiology, illness severity, treatment, need for renal support after hospital discharge, and hospital mortality.
**Results**: of the critically ill burn patients admitted during the study period, 33 (5.7%) had septic complications during their ICU stay, including 18 who were diagnosed with acute kidney injury according to the KDIGO criteria (stage 1—5 patients, stage 2—8 patients, stage 3–5 patients). Patients with AKI had higher severity of burn injury and higher incidence of inhalation injury (Abbreviated Burn Severity Index—9.2 ± 1.9 vs. 7.8 ± 2, and Total Burned Surface Area—39.5 ± 16 vs. 27.6 ± 13, inhalation injury—38.9% vs. 14.3%), *p* < 0.05. The most common contributing factor to AKI was septic shock (72.2%; 95% CI, 69.9–76.5%). Dialysis dependence at hospital discharge was 3% (1 patient) for survivors. Overall hospital mortality was 42%. Septic burn patients with AKI diagnosis had a higher mortality rate in comparison to non-AKI septic patients (65.6%vs. 21.4%). Independent risk factors for hospital mortality included the use of two or more vasopressors (odds ratio [OR], 1.97; 95% CI, 1.50–2.55; *p* < 0.001), presence of AKI (OR, 1.97; 95% CI, 1.73–2.14; *p* < 0.001) and septic shock (OR, 1.42; 95% CI, 1.02–1.81; *p* < 0.01).
**Conclusions**: High incidence of AKI was observed in Burn ICU patients with septic complications. The diagnosis of acute kidney injury in septic burn patients was associated with a high hospital mortality rate.

*O.022*

**Quality of Burn Care from the Patients’ Perspective: Relevance of Outcomes and Quality Indicators**

**C. Lansdorp ^1^, I. Spronk ^2,3,4^, L. van Damme ^4^, D. van Uden ^2^, A. Pijpe ^1,5,6^, M. van Baar ^2,3^, M. Nieuwenhuizen ^7,8,9^, E. Bosma ^7,10^, C. van der Vlies ^1,11^ and P. van Zuijlen^1,6,12,13^**
  ^1^  Department of Plastic Reconstructive and Hand Surgery, Amsterdam UMC Location Vrije Universiteit Amsterdam
  ^2^  Association of Dutch Burn Centres, Maasstad Hospital
  ^3^  Department of Public Health, Erasmus MC, University Medical Center Rotterdam
  ^4^  Dutch Burns Foundation
  ^5^  Burn Centre, Red Cross Hospital
  ^6^  Amsterdam Movement Sciences (AMS) Institute
  ^7^  Association of Dutch Burn Centers, Burn Center Martini Hospital Groningen
  ^8^  Research Group Healthy Ageing, Allied Health Care and Nursing, Hanze University of Applied Sciences Groningen
  ^9^  Department of Human Movement Sciences, University Medical Center Groningen, University of Groningen
  ^10^  Department of Surgery, Martini Ziekenhuis
  ^11^  Trauma Research Unit Department of Surgery, University Medical Center Rotterdam
  ^12^  Burn Center and Department of Plastic and Reconstructive Surgery
  ^13^  Paediatric Surgical Centre, Emma Children’s Hospital, Amsterdam UMC Location University of Amsterdam

**Objectives**: Recently, a project called ‘Highly Specialized Burn Care & Research Programme, The Netherlands’ was started to establish a value-based health care (VBHC) framework with a shared decision-making approach within the three Dutch Burn Centres. Part of this project is the re-design of already existing data registries on patient and injury characteristics and clinical processes (Dutch Burn Repository R3) and patient-reported outcomes (Burn Centre Outcomes Registry, The Netherlands). The objective of the study presented here was to obtain the perspective from a representative group of patients on the relevance and importance of outcomes and quality indicators. This information will ultimately be used to aid a Delphi consensus procedure in which a ‘core set’ of items to be included in the VBHC framework will be established.
**Methods**: This study consisted of a patient survey and two patient focus groups. The (online) survey, available in Dutch and English, contains open-ended questions about outcomes after a burn injury, as well as questions on the importance of specific items based on the International Classification of Functioning. It has been sent out, both digitally and on paper, to 500 burn patients that were treated in a burn centre in The Netherlands in the past 3 to 36 months. for patients with a low level of health literacy or whom are of non-Western culture with a language barrier, interviews using the same survey were conducted. In the two focus groups, each including 3–7 patients, all relevant aspects regarding outcomes and quality indicators during the complete patient journey were discussed and documented.
**Results**: Preliminary results of 136 patients that completed the questionnaire show that ‘wound healing’ (together with infection and wound care), ‘independence’ and ‘sleep quality’ were the most important outcomes: these were rated as ‘very important’ by >60% of participants. Full results are scheduled to be obtained by the end of May, and ready to be presented at the Congress in September.
**Conclusions**: This study provides insight into the relevance and importance of outcomes and quality indicators from a patient perspective. This information will be used in the consensus procedure to establish a VBHC ‘core set’, which will be used to improve quality of burn care and to inform patients on expected outcomes. The VBHC framework will support ongoing innovation and improvements in line with our mission to achieve the best possible autonomy and reintegration into daily life for every burn patient.

*O.023*

**Burn Centres Outcomes Registry in The Netherlands (BORN): First Results of Implementation**

**Margriet Van Baar ^1,2^, D. N. Hulleman ^3,4^, A. S. Niemeijer ^3,5^, A. A. Boekelaar ^6^, N. P. G. Bijker ^4^, J. van de Steenoven ^7^, C. van Schie ^8^, A. Pijpe ^6,9^, M. K. Nieuwenhuis ^3,10^, N. E. E. van Loey ^1^, L. van Dammen ^8^, I. Spronk ^1,2^, E. Middelkoop ^9,11^, S. M. H. J. Scholten-Jaegers ^5^, E. Bosma ^5^, C. H. van der Vlies ^7,12^, P. P. M. van Zuijlen ^6,9^ and T. M. Haanstra ^8^**
  ^1^  Association of Dutch Burn Centres, Maasstad Hospital
  ^2^  Department of Public Health, Erasmus MC, University Medical Centre Rotterdam
  ^3^  Association of Dutch Burn Centres, Martini Hospital
  ^4^  Department for Human Movement Sciences, University Medical Center Groningen
  ^5^  Burn Centre, Martini Hospital
  ^6^  Burn Centre, Red Cross Hospital
  ^7^  Burn Centre, Maasstad Hospital
  ^8^  Dutch Burns Foundation
  ^9^  Department of Plastic, Reconstructive and Hand Surgery, Amsterdam UMC, Amsterdam Movement Sciences, Vrije Universiteit Amsterdam
  ^10^  Research Group Healthy Ageing, Allied Health Care and Nursing, Hanze University of Applied Sciences Groningen
  ^11^  Association of Dutch Burn Centres, Red Cross Hospital
  ^12^  Trauma Research Unit, Department of Surgery, Erasmus MC, University Medical Centre Rotterdam

**Objectives**: The aim of Dutch burn centres to provide optimal person-centred care matching a patient’s preferences and goals by providing the right care, at the right time, in the right place for the right price for every patient. To this end, insight into patients’ values is of utmost importance. A first step is to assess patients’ values, using patient-reported outcomes. The three Dutch burn centres introduced patient reported outcome measures (PROMS) in specialized burn care for adults in 2018. The Burns Centres’ Outcomes Registry in The Netherlands (BORN) routinely collects outcomes of care relevant to the patient, using a state-of-the-art electronic measurement system including real-time feedback for both patient and practitioner.
In this study, we evaluated the response, predictors of response, and outcome levels.
**Methods**: A prospective cohort study was conducted. All adults after an admission to specialized burn care in The Netherlands in 2018–2019 were eligible. We collected and analysed data on response (i.e., reasons for non-inclusion, percentage of participating patients), predictors of response (e.g., patient and burn characteristics), and one of the five routinely collected PROMS; general health-related quality of life (HRQOL) was measured with the EuroQol-5D.
**Results**: Preliminary data show that approximately half of the adults discharged from specialized burn care was registered in the BORN system. Important reasons to refrain from participation were mental health issues (21%), language problems (19%), and refusal (19%). Response rates of the first questionnaire, at discharge, ranged between 40 and 80%. Participation dropped slowly to one-year post-discharge. Participants who survived major burns more often completed the one-year follow-up. Age and length of stay were not associated with this response.
Quality of life data showed that the majority of the patients return to pre-injury levels in most HRQOL domains, except on activities of daily living and anxiety and depression.
**Conclusions**: PROMs are an essential part of person-centred care. BORN response rates dropped slowly over time, and could only partly be predicted by patient or injury characteristics. Response rate seems to be strongly associated with the use in clinical practice. We hypothesize that the use of the PROMS in the consultation room is of major importance in response rates to PROMS. Feedback from the start onwards, also by means of an optimal dashboard, can support the use of individual results in clinical processes for goal setting, shared decision-making, and treatment evaluation.

*O.024*

**Analysis of Factors Affecting Burns Mortality: A National Burn Centre Experience from Pakistan 2007–2021**

**Muhammad Rehan, Tariq Iqbal, Mehwish Sarwar, Muhammad Shais Khan, Muhammad Hassaan Khan, Usman Waheed**
  Burn Care Centre, Pakistan Institute of Medical Sciences, Shaheed Zulfiqar Ali Bhutto Medical University

**Objectives**: Mortality rates are significant outcome parameters after a burn injury. The objective of the current study was to analyze the characteristics of the patients admitted to our burn care centre and identify the factors associated with mortality in the burn.
**Methodology**: This was a cross-sectional single-centre study involving a retrospective analysis of mortality rates in burn patients over a period of 15 years from January 2007 to December 2021. during the study period, 7866 burn patients were admitted to the ICU of the burn care centre. Data were acquired at the time of admission and were logged prospectively during the course of the hospital stay. The patients were followed until discharge or death.
**Results**: during the reporting period of 2007–2021, 7866 patients were registered in the current study with the majority of them being males (64.09%). The mortality rate was calculated as 23.16% (1822/7866). The patients who died were mostly males (64.33%) and were considerably younger (mean death age 14.34 years). The majority of the patients (both groups) had a total body surface area (TBSA) of > 60%, mostly with inhalation injury. The TBSA was statistically associated with burn mortality. The average duration of stay at the burn care centre was 15.5 days for the survivors’ group (Group 2), whereas it was 11.4 days for the patients who died (Group 1) during the course of their treatment. The degree of burn had a positive correlation with mortality (*p* = 0.001). A rise in the burn degree from second- to third-degree significantly increased the risk of death.
**Conclusions**: About 23.16% of all admitted patients died mostly from flame burns and sepsis as the most common cause of death. Increased burned TBSA, younger age, and inhalation injury worsened the prognosis.
Applicability of clinical practice: The causes of mortality after burn injury change over time and data need to be updated for informed decision-making.

*O.025*

**Multinational Analysis of the Treatment of Burn Patients in Africa—Infrastructure, Challenges and Solutions**

**Julia Elrod, Judith Lindert, Dorothy Bbaale, Shobha Chamania and Christoph Mohr**
  University Medical Center Mannheim, Heidelberg University

**Objectives**: Burns remain a serious public health problem, especially in low- and middle-income countries. Socioeconomic status and health systems with limited resources impede high-quality surgical care. The objective of this study was to systematically collect data on the health care of burned patients on the African continent as a basis for improving burn care.
**Methods**: An online survey covering the topics of the current health care situation, available infrastructure, human resources, perceived challenges and solutions for improvement of the treatment of thermal injuries was created. It was distributed in English and French with snowball methods via online platforms, social media, distribution lists and by email. Descriptive statistics were performed; in addition, the AI-based random for est was applied to identify determinants for a reduction in the reported mortality rate.
**Results**: In total, 271 evaluable questionnaires from 237 cities in 40 African countries were analysed. Overall, 81.9% originated from countries with a very low Human Development Index (HDI) (4th quartile). The majority of all responses were from tertiary health care facilities. In only 18.8% of respondents, therapy was free of charge for the patients. A separate burn ward was available in 34.7% of all institutions and an intensive care unit for burn patients was available in 21.0%. Regular skin grafts were performed in a minority of all centers only. Mortality varied significantly between centers. Random-forest-based analysis revealed a close association between HDI (feature importance: 0.38) and mortality. The most important reason for poor outcome was perceived to be late presentation due to prior treatment by a traditional healer. The greatest perceived potential for improvement was introduction of an intensive care unit, followed by improved prevention or education of the population.
**Conclusions**: This study provides an ample overview of the current state of care for burned patients on the African continent. A variety of factors, including a low HDI, delayed hospital presentation, e.g., due to prior care by non-physicians and lack of equipment seem to worsen the outcome. Introduction of an intensive care unit and communal education are perceived to be important steps in improving health care in burns.

*O.026*

**Epidemiology of Burns in Latin America: Do We Need a Regional Burn Registry?**

**Orlando Flores, Jorge Rojas and Rodrigo Fuentes**
  COANIQUEM

**Objectives**: Burn injuries cause important loss to health status globally. Despite being considered clinically relevant in Latin America, there is not a systematic registry for epidemiological and clinical data in the region. The aim of this study is to (a) analyse the quantity and quality of reported epidemiological and clinical data on burn injuries across Latin America and (b) to discuss the need for a regional burn registry for the region.
**Methods**: A scoping review was performed following the PRISMA extension for scoping reviews. The search strategy was used in PubMed and Scielo databases, using “epidemiology”, “burns”, “Latin America” and related terms as search words. Data were tabulated and selected articles were critically appraised to inform the quality of the information reported.
**Results**: Epidemiological and clinical data from 28 studies were retrieved. The majority of them reported data from single-centre studies, with a limited number of cases included. No studies referred to a national or regional registry of data for burn injuries in Latin America.
**Conclusions**: Latin America has a scarcity of epidemiological and clinical data that can help to decrease the burden of burns injuries on morbidity and mortality. A well-designed registry will provide information that allows benchmarking between Latin America and other regions, determines levels of coverage, and sets up the quality-of-care indicators and compliance.

*O.027*

**Epidemiological Trends of Burns, 2016–2020 in Lithuania**

**Viljamas Sipavičius ^1^, Monika Rimdeikaitė ^2^ and Rytis Rimdeika ^3^**
  ^1^  Faculty of Medicine, Lithuanian University of Health Sciences
  ^2^  Faculty of Medicine, Vilnius University
  ^3^  Hospital of Lithuanian University of Health Sciences Kaunas Clinics, Plastic and Reconstructive Surgery Clinic

**Objective**: To evaluate the epidemiological characteristics of burns in Lithuania over five years.
**Methods**: Data were used from the Health Information Center of the Institute of Hygiene. Statistical analysis of the data was performed using IBM SPSS statistics 23.0 software. A comparison was made between the percentage distributions of burns during the study period using the Mann–Whitney U criteria.
**Results**: during a 5-year period (2016–2020), adults were affected by burns. Men accounted for 55.51% burns and women for 44.49%. Men experienced burns more often. There was a statistically significant difference between the genders (*p* = 0.009). A decreasing trend in burns was observed in the adult group. Burns decreased by 5.56% in 5 years. In the group of men, burns reduced from 12.10% to 8.51% (decrease of 3.59%) and in the group of women it reduced from 9.93% to 7.97% (decrease of 1.96%).
Distribution of burns localization: head and neck, 7.31%; trunk, 7.60%; shoulder and arm, 10.69%; wrist and hand, 13.36%; hip and foot, 14.20%; eye and its accessory organs, 23.27%; airways, 0.69%.
Over 5 years, according to the localization of the burns, the percentage of burns decreased: head and neck by, 4.77% (more men, *p* = 0.009); trunk, 11.16%; shoulder and arm, 8.37% (more men, *p* = 0.036); wrist and hand, 2.3% (more men, *p* = 0.047); hip and leg, 0.94% (more men, *p* = 0.009); ankle and foot, 8.94%; eye and its accessory organs, 16.31% (more men, *p* = 0.009); respiratory tract, 5.26% (more men, *p* = 0.009).
**Conclusions**: Our epidemiological analysis showed that men experienced burns more often than women. The most common burn localizations were the wrist and hand and/or eye with its accessory organs. Evaluating the analysis of burns in Lithuania in 2016–2020, we can state that there is a positive change, as the number of burned patients is decreasing. Burns decreased by 5.56% in 5 years.

*O.028*

**Elderly Patients in a Major Burn Center: A Five-Year Review**

**Dmitry Shelepenko, José Miguel Azevedo, Gonçalo Tomé, Inês Catalão and Luís Cabral**
  Coimbra Hospital and University Centre (CHUC)

**Objectives**: In recent decades, there has been a growing increase in the aging of western populations. Elderly patients represent an important part of hospitalizations in most Burn Units, due to their greater predisposition to accidents, their comorbidities and/or greater need for health care. The aim of this study was primarily to identify typical characteristics of the elderly population and factors that may alter the course and outcomes of these patients, based on a statistical analysis of patients admitted to a European Burn Unit over a five-year period.
**Methods**: The Authors analyzed medical records of all patients admitted to Coimbra Burns Unit, in Portugal, between 1st January 2017 and 31st December 2021. Patients were divided into two main groups: “non-elderly” (<65 years) and “elderly” (≥65 years). The groups were compared in terms of time, cause and place of accident, total burn surface area (TBSA) and affected anatomical structures, airway injury, number of visits to the operating room (OR), mean length of stay and mortality.
**Results**: during the sampling period, the number of admissions reached 683 patients (284 women, and 399 men). 374 were under 65 years (112 women and 262 men) and 309 (172 women and 137 men) were older than 65 years. The source of injury was mainly fire (52%) and hot liquid (27%). The most frequent place of accidents was at home (75%) followed by work (16%). In the population aged over 65 years, only 1% had an accident in the workplace, and the percentage of electrical and chemical burns was quite small.
Approximately 80% of all patients had between 0 and 20% of TBSA, and the mortality of these patients was 1.6% in <65 group and 8.5% in those aged ≥65. Even greater differences were observed with more extensive burns, and the affected anatomical regions also varied. In the elderly group, there was also an increased number of visits to the OR (79% vs. 56%), greater number of days of hospitalization (23.6 vs. 19.8) and a higher global mortality rate (16% vs. 5%).
**Conclusions**: With the advancement of medical and surgical care, we have been able to save the lives of many elderly patients with extensive burns. However, morbidity remains high and greater investments must be made in preventing these accidents. The elderly population and their families must be aware of risks from fireplaces, hot water bags and pots, especially when potential victims are left at home alone.

*O.029*

**The Use of Enzymatic Debridement Therapy for Deep Burns Treatment According to Functional Regions—A Clinical Study**

**Angela Tecuceanu ^1^, Teodor Stamate ^2^, Dan Cristian Moraru ^2^, Clara Larisa Ibanescu ^3^, Irina-Mihaela Jemnoschi Hreniuc ^1^, Ioana Munteanu ^2^, Caterina Lorenzi ^2^, Teodor Popa ^2^, Audrey Clebant ^2^, Camelia Tamas ^2^ and Cristian Moraru ^2^**
  ^1^  University of Medicine and Pharmacy “Gr. T. Popa”
  ^2^  Plastic, Reconstructive and Burns Surgery Clinic “Sf. Spiridon” Emergency County Hospital
  ^3^  Emergency Hospital “Sfantul Ioan cel Nou”

**Objective**: To evaluate the particularities and efficiency of the enzymatic debridement using bromelain powder in the treatment of second- and third-degree burns, located on the limbs and trunk regions.
**Methods**: Enzymatic debridement is an effective minimal invasive method in deep burns treatment, proven good results in eschar removal. In our retrospective study, we included 16 patients (10 men and 6 women), hospitalized in our Burn department from the “Sf. Spiridon” Emergency County Hospital Iasi, during a 4-year period (2019–2022), which met the application criteria for enzymatic debridement (using bromelain powder). All the patients included in our study suffered thermal injuries, with a total burn area ranging from 5 to 40% tbsa, IIA, IIB and III degree. We applied the enzymatic therapy within the first 72 h after the injury (12 cases in the first 24 h, 2 cases within the first 48 h, 2 cases in the first 72 h).
For our selected patients, the use of enzymatic debridement was performed at the level of the upper and trunk. The distribution of the lesions according to the anatomical region was as follows: upper limb (3 cases with IIA and IIB burns localized on the arm, 6 cases with thermal injuries IIB-III degree of the for earm and hand, including a circumferential burn lesion), lower limb (2 cases on the anterior thigh- IIB degree,2 cases of the dorsal skin foot-IIA degree and 1 on the anterior-external face of the calf-IIA and IIB injuries), 2 cases on the trunk-IIB and III degree.
We combined the enzymatic debridement with local applications of ointments based on the low molecular weight hyaluronic acid (LMW-HA) in IIA burns-6 cases, or with skin graft reconstruction and LMW-HA topics in IIB-III burns (10 cases).
**Results**: The survival rate was 75% (12 cases). In 11 cases, the wounds had negative bacteriological secretions. The patients were satisfied due to the fast healing, good esthetic and functional result.
**Conclusions**: The enzymatic debridement of the burned tissue is an important tool in wound management, reducing the hospitalization period and saving the skin graft donor sites.

*O.030*

**Mortality Reduction in Major Burn Patients over 70 Years Old Treated with Enzymatic Debridement**

**Rocio Terrados, Hernan López-Tello, Jose Ramón Martínez Méndez**
  La Paz University Hospital

**Objectives**: The elderly burn population can be regarded as a vulnerable and often challenging group for specialized burn units. The aim of this research was to study the effect of surgical treatment with enzymatic bromelain debridement in mortality rates on major burn patients aged 70 or older when compared with tangential debridement.
**Methods**: All patients admitted in the Burns Unit of the La Paz Hospital of Madrid (Spain) between 2010 and 2021, aged 70 or older, who had burns affecting more than 10% total body surface area (TBSA) were collected. Patients who did not undergo at least one debridement surgery during their stay were excluded from the analysis. Differences in patient mortality between those who received enzymatic versus tangential debridement were assessed using the chi squared test for independence, establishing 0.05 as the maximum tolerable probability of type I error.
**Results**: In total, 38 patients were admitted. Their mean age was 80.3 ± 6.2 years (55.3% male). Regarding% TBSA, the mean value was 16.4 ± 5. There was no significant difference in distribution of burn severity, reflected by the ABSI score, Charlson comorbidity score, inhalation rate and number of interventions between treatment groups. The mortality rate in the group that underwent tangential excision was 44%, whereas it reached 9% in the group treated with enzymatic debridement. The comparison showed a statistically significant difference (*p* = 0.037). Further mortality analysis within subgroups showed statistically significant differences when using enzymatic debridement on patients who had a TBSA greater than 15% (*p* = 0.045).
**Conclusions**: Enzymatic debridement has shown a significant reduction in mortality rates in this study when applied to elderly patients who sustained major burn injuries as opposed to tangential excision. Moreover, further subgroup analysis suggests that enzymatic debridement could be superior when treating patients with a% TBSA greater than 15%. The data show that enzymatic debridement is safe when applied to elderly patients and should be considered as a treatment option.

*O.031*

**Helpful Hints in Deciding What and When to Operate after Enzymatic Debridement**

**Karel Claes ^1^, Ignace De Decker ^1^, Stan Monstrey ^1^, Yaron Shoham ^2^, Tom Vyncke ^1^ and Henk Hoeksema ^1^**
  ^1^  Ghent University Hospital
  ^2^  Soroka University Medical Center, Ben-Gurion University of the Negev

**Objectives**: In recent years, it has become clear that the burn eschar in deep burns can be selectively removed using the enzymatically debriding agent NexoBrid^®^ (EDNX). In deep partial-thickness burns, such selective debridement preserves the non-injured dermis, which is sometimes sufficient for spontaneous re-epithelization. Nevertheless, it can be extremely challenging to determine exactly what and when to operate after an EDNX procedure.
**Methods**: Mainly LDI-blue areas, determined between 48 h and 5d post-burn, which were afterwards treated with EDNX were selected. Six practical and three expert EDNX users evaluated the high-quality digital images of the wound beds immediately post NexoBrid^®^ removal and after a 2 h wet-to-dry (WTD) dressing period.
**Results**: Overall, 102 mainly LDI-blue areas in 32 patients were analyzed. Wound bed evaluations post EDNX versus post WTD of all 9 EDNX users were not significantly different. Moreover, there was a good to excellent consensus between the practical and expert EDNX users in the wound bed evaluations. Although there was an 84% consensus on the decision whether or not to operate, there was only partial consensus (65%) in the clinical evaluation of the exact healing time, which might be of less importance after enzymatic debridement.
All mainly LDI-blue areas with incomplete enzymatic debridement, determined during clinical investigation by expert EDNX users, required surgery. Additionally, the investigators demonstrated that the following wound bed characteristics were independent predictors of the need for surgical treatment: visible fat lobules (*p* = 0.028), translucent fat lobules (*p* < 0.001), dermal step-off in the wound bed (*p* < 0.001), visible blood vessels (*p* < 0.001) and coagulated blood vessels (*p* = 0.023). Additionally, higher color code ranges on our own developed wound bed classification were significantly related to a surgical intervention (*p* = 0.006).
**Conclusions**: This study is the first to address the clinical wound bed evaluation of LDI-confirmed deep burns immediately after removing NexoBrid^®^ as well as after a 2 h WTD dressing. during this evaluation, wound bed characteristics such as incomplete debridement, visible and/or translucent fat lobules, visible and/or coagulated blood vessels and a dermal step-off in the wound bed should lead to an early and reliable decision for skin grafting. These characteristics, combined with a higher range (4–5) in the newly developed wound bed color code, suggest low spontaneous healing potential post EDNX (Figure 1). for burn centers using LDI, mean flux values below 119.5PU—in addition to the above-mentioned wound bed evaluation—are a clear indicator for surgical therapy.

*O.032*

**One Year Follow up Results of the DETECT Enzymatic Debridement Multicenter RCT**

**Yaron Shoham ^1^, William Hickerson ^2^, Jeremy Goverman ^3^, Sigrid Blome-Eberwein ^4^, Adam Singer ^5^, Lucy Wibbenmeyer ^6^, Nicholas Meyer ^7^, Joshua Carson ^8^, James Gallagher ^9^, Steven Kahn ^10^, Dhaval Bhavsar ^11^, Tam Pham ^12^, Jeffrey Shupp ^13^, Eliana Pagnozzi ^14^, Ilaria Mataro ^14^, Tudor Andrei Stancioiu ^15^, Bretislav Lipovy ^16^, Guga Kashibadze ^17^, Stan Monstrey ^18^, Henk Hoeksema ^18^, Silviu Marinescu ^19^, Ana Maria Boiangiu ^19^, Bernd Hartmann ^20^, Frank Sander ^20^, Yehuda Ullman ^21^, Antonio Di Lonardo ^22^ and Kevin Foster ^23^**
  ^1^  Soroka University Medical Center
  ^2^  Firefighter’s Regional Burn Center
  ^3^  Mass General Hospital
  ^4^  Lehigh Valley Hospital Network
  ^5^  Stony Brook University
  ^6^  University of Iowa Hospitals
  ^7^  Columbia St Mary’s Hospital
  ^8^  University of Florida Health System
  ^9^  New York Presbyterian Hospital
  ^10^  Medical University of South Carolina
  ^11^  University of Kansas Health System
  ^12^  University of Washington Regional Burn Center
  ^13^  Medstar Washington Hospital Center
  ^14^  Cardarelli Hospital
  ^15^  Emergency Hospital of Plastic, Reconstructive and Burns Surgery
  ^16^  University Hospital
  ^17^  Khechinashvili University Hospital
  ^18^  University Hospital
  ^19^  Bagdasar-Arseni Clinical Emergency Hospital
  ^20^  BG Klinikum Unfallkrankenhaus
  ^21^  Rambam Medical Center
  ^22^  Ospedale di Cisanello
  ^23^  Maricopa Medical Center

**Objectives**: NexoBrid^®^ (NXB) enzymatic debridement of deep thermal burns was approved for use in the EU a decade ago, and since then has gradually been introduced into common burn care. A second phase 3 multicenter RCT, the DETECT study, was conducted as part of post approval commitments in the EU and the approval process in the US. Topline acute stage results of this study were reported previously. The objective of this abstract is to present the 12-month follow up results of predefined endpoints of scar quality, function and quality of life (QoL).
**Methods**: Overall, 175 adult patients with deep burns were randomized in a phase 3 RCT to one of three treatment arms—NXB, Standard of Care (SOC), or Gel vehicle (placebo control) in a 3:3:1 ratio (75 NXB, 75 SOC, and 25 Gel). Scar quality (cosmesis) and function data were analyzed for long-term data collected at 3, 6, 12 and 24 months. Cosmesis and function were measured using Modified Vancouver Scar Scale (MVSS) and Patient and Observer Scar Assessment Scale (POSAS) to demonstrate that NXB treatment was non-inferior to SOC treatment, as measured at 12 months from wound closure date, evaluated by assessors blinded to the treatment arm. QoL was measured by the EQ-5D (EuroQol 5 Dimensions), VAS (Visual Analog Scale) and BSHS-B (Burn Specific Health Scale—Brief) scales. Missing values were imputed in the analysis using multiple imputation, with best-case and worst-case imputations as sensitivity analyses.
**Results**: The 12-month follow-up mean MVSS scores were significantly lower (better) for the NXB group (3.70 ± 2.10) compared with the SOC (5.08 ± 3.11) and Gel groups (5.63 ± 2.99). A regression analysis showed that NXB had a 1.36 MVSS point advantage over SOC after adjustment for all other variables in the model (*p*-value = 0.0027). The 95% CI for this treatment effect was −2.24 to −0.48, excluding 0, indicating superiority of NXB over SOC. The 3- and 6-month follow-up MVSS scores were also lower for the NXB group (5.51 ± 3.09, 4.43 ± 2.59) compared with the SOC (6.63 ± 3.44, 5.43 ± 3.75) and Gel (7.56 ± 2.67, 8.89 ± 3.37) groups. POSAS scores followed similar trends to MVSS scores but did not reach statistical significance. QoL was similar among treatment arms.
**Conclusions**: In addition to the significant acute stage results presented previously, the long-term results of this RCT further demonstrate the safety of NXB treatment, including significantly better 12-month follow-up MVSS scores.
**Funding**: Funded with US Federal funds, BARDA, under Contract No. HHSO100201500035C.

*O.033*

**Laser Speckle Contrast Analysis (LASCA) Technology for the Evaluation of Burn Wound Enzymatic Debridement**

**Karolina Ziółkowska, Marcin Gierek, Przemysław Strzelec, Wojciech Łabuś and Diana Kitala**
  Dr Stanislaw Sakiel Centre for Burn Treatment, Siemianowice Ślaskie, Poland

Enzymatic demarcation becomes one of the surgeon’s primary tools in the treatment of burns. In the method using Nexobrid containing proteolytic enzymes, it is assumed that only the necrotic tissue would be removed. The living tissue would remain intact and be prepared directly for the next stage of treatment, i.e., covering the wound with a graft or other special dressing. Surgeon’s assessment of the degree of burn and tissue viability may require support from medical devices. The PeriCam PSI System uses an invisible near-infrared (NIR) (785 nm) laser for the blood perfusion measure. Laser speckle contrast analysis (LASCA) technology is a non-invasive method that enables the immediate visualization of microcirculation perfusion in the examined tissue.
**The Aim of Study**: The goal of the study was to verify the effectiveness of the enzymatic method of demarcation of burn wounds by means of LASCA technology.
**Material and Methods**: From October 2021 to April 2022, at the Dr Stanislaw Sakiel Centre for Burns Treatment in Siemianowice Śląskie, enzymatic demarcation along with the LASCA study was carried out among 21 patients. The procedure was started with the LASCA test before demarcation, then immediately after removing Nexobrid and 24 h after enzymatic demarcation.
**Conclusions**: The LASCA is a non-invasive method that enables immediate visualization of microcirculation perfusion in the examined tissue. The combination of two methods: enzymatic demarcation and LASCA allows us to use the least invasive demarcation method and to effectively, non-invasively and quickly assess the degree of wound cleansing, and choose the best method of further treatment. The use of LASCA technology may be useful tool for the prospect decision concerning the further therapeutic scheme.

**Keywords**: enzymatic demarcation; Nexobrid; necrosis removal; burns; LASCA; laser analysis

*O.034*

**Elderly Patients and Burn Injury: Enzymatic Debridement, Did We Gain Anything? Preliminary Data**

**Jasminka Minic, Enrico Vigato, Edoardo Dalla Pozza and Maurizio Governa**
  Plastic Surgery and Burn Unit, A.O.U.I. of Verona

**Objective**: Constant aging of the world population increases the incidence of burn injuries in the elderly population. The complexity of elderly patients in due to many physiological changes and comorbidities plays an important role in the outcome of burn injuries. Successful burn treatment of elderly patients is a challenging task not only in the acute phase but also in their long-term care. Selective enzymatic debridement (ED) as an alternative to standard of care (SOC) in burned adults has proven efficacy in the literature, but its use in the elderly has limited data.
**Methods**: We performed a retrospective cohort study of 89 elderly patients consecutively admitted at our Burn Unit for partially deep and deep burns in the last five years. Patients were divided into two groups according to the acquired treatment, with ED (36%) or SOC (64%). Demographic data, surgical approach, TBSA percentage, burn depth, number of surgical interventions, transfusions, comorbidities and outcomes were collected by medical registers and elaborated through statistical analysis. All two-sided *p*-values less than 0.05 were considered significant.
**Results**: The groups were comparable for demographic parameters (mean age 75.8 ± 9.8 years old), presence of comorbidities (61.8%) and burn features (mean TBSA 18.5 ± 7.5%). Complete selective debridement was achieved earlier (*p* < 0.0001) and with fewer transfusions (*p* < 0.0001) in the ED cohort compared with the SOC cohort; the total number of surgical procedures needed for healing was lower for the ED group, but still not significant (*p* = 0.051). The total number for transfusion at the discharge was similar between groups. Overall, 10 out of 89 patients died (11.2%) without statistical significance between the two groups. The mortality rate per 1000 person/days was 2.7 (95%CI = 1.4–5.2): 3.3 (95%CI = 1.6–6.9) in the SOF group and 1.7 (95%CI = 0.4–6.7) in the NXB group. Age (HR = 1.09; 95%CI = 1.01–1.18) and TBSA (HR = 1.1; 95%CI = 1.01–1.2) represented at the univariate analysis as the only significant factors for overall survival (OS).
**Conclusions**: Regardless of the limited number of patients, these preliminary results show that selective enzymatic debridement might represent a new weapon in the early treatment of elderly burned patients, a high-risk subpopulation whose mortality is particularly high both in the acute phase and in the catabolic phase. Additional investigation will certainly clarify the importance and usefulness of this new approach in terms of quality of life and prognostic outcomes in this fragile category.

*O.035*

**The Benefit of Nexobrid on Patients with Partial to Full-Thickness Burns Compared with Those Undergoing a Surgical Escharectomy**

**Antonella Marisa Citterio, Bianca Jade Mare and Franz Baruffaldi Preis**
  Niguarda Hospital

The main goals to be pursued in the treatment of burn injuries is the debridement and the promotion of a rapid re-epithelization in partial-thickness injuries, the preparation of an ideal bed for graft coverage in full-thickness burns. The treatment is based on surgical escharectomy or the autolytic process.
The study sought to compare the outcomes of Nexobrid vs. surgical escharectomy in adult patients with termal burns on between 15% and 45% of the TBSA. The depth of the burn was determined on clinical evaluation performed by the specialist. Calculation of the extent of the affected surface was carried out with a Lund and Browder map.
A case–control observational study was performed, examining a total of 40 patients from 2020 to 2021 in the Milan Burn Unit. Overall, 20 patients were treated with Nexobrid and the other 20 underwent a standard escharectomy.
The study shows that blood transfusions (*p* ≤ 0.005), infection rate (35% vs. 85%), time of debridement (2 days vs. 5 days), need of skin grafting (*p* = 0.0019), surgical excision (*p* = 0.0032) and hospital stay (*p* = 0.0059) decreased significantly.
The efficacy, the quickness, selectivity, and safety of enzime debridement with Nexobrid in burn therapy have been proven, and it can be used as an alternative to standard care burn treatment.

*O.036*

**Fatigue after Burn—How Does Fatigue Affect Daily Activities, Health-Related Quality of Life, and Return to Work: Preliminary Results**

**Sara Enblom ^1^ and Fredrik Huss ^1,2^**
  ^1^  Burn Center, Department of Plastic and Maxillofacial Surgery, Uppsala University Hospital
  ^2^  Department of Surgical Sciences, Plastic Surgery, Uppsala University

**Objectives**: Physical function and daily activities are often affected after a burn injury. Previous research shows complications that significantly affect recovery after a burn injury, such as pain, muscle weakness, scar contractures, and hypertrophic scarring. Many also report fatigue as a complication.
The aim with this ongoing study is to investigate the correlation between fatigue and activity performance, health-related quality of life, and return to work following a burn injury. In what way do these correlations change the first year post-burn? Is there a correlation between these variables and TBSA%, length of stay, and sex? Is there a correlation between fatigue and anxiety/depression? Can written information about fatigue improve the patient’s ability to handle daily life?
**Methods**: Seventy patients scheduled for follow-up at the Burn center’s outpatient clinic in Uppsala, Sweden, 6 months post-burn, who fulfill all inclusion criteria and no exclusion criteria, are asked to participate in the study. The study started in May 2020 and is estimated to take 4 years to complete.
Apart from ordinary assessments the patients that are included fill in three additional questionnaires. These assessments are also filled in at the follow-up visit 6 months later.
The following assessments are used in the study:
Fatigue Severity Scale (FSS), a self-assessment scale translated to Swedish but not yet validated for burns; Brief Fatigue Inventory (BFI), a self-assessment scale, valid and reliable for burns but not validated in a Swedish population; EQ-5D, a standardized assessment for measuring self-assessed health-related quality of life and current state of health; PS-ADL, is a valid and reliable self-assessment instrument evaluating activity performance; Hospital Anxiety and Depression Scale (HADS), is a valid self-assessment scale evaluating anxiety and depression. Return to work is assessed through a couple of questions regarding current employment/studies.
To answer the question if simple written information can affect the patients experience of fatigue, patients with pronounced fatigue are randomized to a control group or an intervention group. The intervention group receives information about fatigue, the control group receives none. Patients without fatigue for m a reference group whose data are evaluated regarding the correlation between fatigue and anxiety/depression.
**Anticipated Results**: The study will hopefully give a broader understanding about how fatigue affects daily living and if there are correlations that can predict the development of fatigue.
The study has been approved by the Swedish Ethical Review Authority (Dnr 2020-00387). The study is ongoing and preliminary results will be presented.

*O.037*

**Exoskeleton Robot Using 3-Dimensional Modeling in Hand Burn Injury Patient**

**Cheong Hoon Seo**
  Hallym University

**Objectives**: Hands are the part of the body that are most commonly involved in burns, and the main complications are finger joint contractures and nerve injuries. Hypertrophic scarring cannot be avoided despite early management of acute hand burn injuries, and some patients may need application of an exoskeleton robot to restore hand function. To do this, it is essential to individualize the customization of the robot for each patient. Three-dimensional (3D) technology, which is widely used in the field of implants, anatomical models, and tissue fabrication, makes this goal achievable.
**Methods**: This report is a study on the usefulness of an exoskeleton robot using 3D technology for patients who lost bilateral hand function due to burn injury. Five burn patients with upper limb dysfunction after a flame and chemical burn injury, with resultant impairment of manual physical abilities, were examined.
**Results**: after wearing an exoskeleton robot made using 3D printing technology, the patients could handle objects effectively and satisfactorily
**Conclusions**: This innovative approach provided considerable advantages in terms of customization of size and reduction in manufacturing time and costs, thereby showing great potential for use in patients with hand dysfunction after burn injury.

*O.038*

**Health-Related Quality of Life and Social Reintegration after Burns**

**Maria Fernanda Hutter, Christian Smolle and Lars-Peter Kamolz**
  Department of Plastic, Aesthetic and Reconstructive Surgery, LKH University Klinikum Graz

**Objective**: Burn injuries are common severe and devastating injuries, which can have a lasting impact on the patients’ life on a physical, psychological and social level. In recent decades, mortality has been greatly reduced due to many advances in intensive care medicine, skin replacement procedures and surgical management. Consequently, not only survival but also health-related quality of life (HRQoL) and social reintegration (SR) are increasingly important outcome parameters. The present study aims to take a first step towards the assessment of HRQoL and SR after burn injuries in the Austrian population.
**Material and methods**: In this single-center follow-up study, self-reported HRQoL and SR of 128 of 388 (33.0%) adult in-patients overall with for mer burn injuries, treated between 2012 and 2019 at the Division of Plastic, Aesthetic and Reconstructive Surgery at the Department of Surgery at the University Hospital of Graz, were assessed using the SF-36 V1.0 in German and further questions evaluating SR. The questionnaire outcomes were set into relation with clinical data obtained from the medical records. Statistical analysis was performed with SPSS 27.0 for Windows.
**Results**: of the 128 participants, 72.7% were male and 27.3% were female. Mean age at the time of injury was 40.0 years (±15.7) and mean%TBSA was 9.2% (±11.0%). Male patients had sustained significantly more extensive injuries (*p* = 0.005). The study revealed that female patients scored significantly (*p* < 0.05) and consistently lower in all domains of the SF-36, except for “bodily pain” (*p* 0.061). Additionally, female patients scored lower in all domains of SR. However, significant differences were only found in the domain fulfillment (*p* = 0.050) and mental wellbeing (*p* = 0.015). of the pre-burn employed male participants, 86% were employed upon interview, as were 62.9% of females, whereas in total, only 3 participants had lost their jobs. Overall, unemployment had declined (6.3% vs. 10.2% at the time of interview). Consumption of alcohol, as well as tobacco and illegal drugs, decreased for all patients. Psychiatric disorders were more common in women than in men (17.1% vs. 2.2%, *p* = 0.002).
**Discussion**: SR after burn injury in this study cohort seems to be good. Return to work, as well as substance consumption, has shown a promising trend. Strikingly, HRQoL was lower in women after burn injury, and psychiatric comorbidities were also more common in women. Further research on reasons for this gender discrepancy and possible targets to improve rehabilitation these patients.

*O.039*

**Development and Validation of an Algorithm for the Identification of Activities Typical for Burn Intensive Care Patients Based on Two Accelerometers**

**Yvonne Dikkema ^1,2,3^, L. J. Mouton ^3^, K. W. Gerrits ^3^, T. A. Valk ^3^, M. van der Steen-Diepenrink ^4^, J. E. Eshuis ^5^, H. Houdijk ^3^, C. P. van der Schans ^2,6,7^, A. S. Niemeijer ^1^ and M. K. Nieuwenhuis ^1,2,3^**
  ^1^  Association of Dutch Burn Centers, Burn Center Martini Hospital
  ^2^  Research group Healthy Ageing, Allied Health Care and Nursing, Hanze University of Applied Sciences Groningen
  ^3^  Department of Human Movement Sciences, University Medical Center Groningen
  ^4^  Department of Intensive Care Martini Hospital
  ^5^  Burn Center, Martini Hospital
  ^6^  Department of Rehabilitation Medicine, University Medical Center Groningen, University of Groningen
  ^7^  Department of Health Psychology, University Medical Center Groningen, University of Groningen

**Objective**: Detailed information on the amount of activity performed is essential to tailor rehabilitation. However, intensive care (IC) patients, including people with burns, are relatively inactive, making it difficult to gain insight into the actual physical activity performed, including nurse-initiated activities such as positioning or transfers to the chair. Accelerometer data combined with an algorithm that converts these data into activity information can potentially measure such activities. To date, however, there is no valid and reliable algorithm to analyze activities specific to (burn) IC patients. Therefore, our aim was to develop and validate an algorithm for the identification of activities typical for burn intensive care patients based on two accelerometers.
**Methods**: A cross-sectional study was performed with 10 healthy participants in January–February 2021. The participants wore two accelerometers, one on their chest and one on their contra lateral leg while performing 14 different static and dynamic activities typical for burn IC patients (e.g., prone-supine lying, lying on the left or right side, semi sit, sitting on edge of bed or chair, standing, walking, bed cycling and transfers/transitions in and around an ICU bed). Video recordings served as a reference standard. Activities were classified from the videos by two different raters and interrater reliability was determined. Development of the accelerometer algorithm was based on body angles and bodily movement of four participants maximizing the percentage agreement (%agr) relative to video observations. Validity of the algorithm was assessed with%agr against the video using the data of the remaining six participants.
**Results**: Reliability between the two video-raters was high, with ICCs all above 0.76, except for walking (0.30). The algorithm had high%agr on lying activities (all above 95%), and on sitting activities (all above 74%). Transfers performed by the participants themselves scored very high (80–84%agr) but transfers with the aid of a health care provider scored low (14–18%agr). Bed cycling scored as moderate to high (57–75%agr).
**Conclusions**: This study shows that we have developed a valid algorithm for the identification of activities typical for burn IC patients based on two accelerometers. This method can be used for future studies, to gain insight in what specific activities are performed, as well as their frequency and duration, during the day and night, and even throughout the whole hospital stay. With this information, a better understanding on the amount of activity performed by (burn) IC patients can be obtained.

*O.040*

**Implementation of a Cross-Cultural Knowledge Translation Model in Paediatric Burns in Chile: A Pilot Initiative**

**Jenniffer Garcia and Orlando Flores**
  Coaniquem

**Objectives**: To develop a cross-cultural knowledge translation initiative in a pediatric burns centre in Chile. Current literature describes that health professionals in Chile face several drawbacks that limit their abilities to access scientific evidence and inform practice with the best available information. This project aims to build a culture of research and develop the capacity for knowledge translation and implementation among members of a rehabilitation team in Chile, in partnership with Australian implementation scientists’ experts in the field of burns. A cross-cultural mentoring model has been utilized to guide the process.
**Methods**: Community-based participatory research is being used as a methodological approach to collaboratively work with ten Chilean members of an interdisciplinary rehabilitation team, to implement evidence-based approaches in their practice with children with burns.
**Results**: The findings will discuss the results of a pioneering initiative in Chile, identifying barriers and facilitators to the cross-cultural adaptation and implementation of evidence-based approaches in the field of pediatric burns in Chile.
**Conclusions**: The lack of knowledge locally produced and restricted abilities to adapt and implement research knowledge into clinical practice deters the capacity of Chilean health professionals to deliver high-quality services for children with burns. Intercultural and interlingual partnerships are needed to bridge the gap and support the development of knowledge translation skills of clinicians from non-English and/or developing countries that remain isolated from the global progress made in the field of burns.

*O.041*

**Self-Management…Right at the Start**

**Karin Bouwmeester**
  Martini Ziekenhuis

**Objectives**: Self-management aims to empower people with chronic conditions to take control of their treatment. It is a set of approaches which helps a patient manage their own health. This could be physical health, mental health or both. Important items of self-management are patient-centered care, shared decision-making, patient empowerment and education. Patients can only take control over their lives (or treatment), if they are involved in setting their own rehabilitation goals and if they have the confidence that they are sufficiently equipped to carry out the required activities.
**Methods**: In burn centers, people are admitted who, in a split second, are transformed from a normal functioning person into a patient with burns. Treatment in burn centers can take a long time. It can take years even to recover from severe burns. It can turn into a ‘chronical’ situation, a situation where self-management applies. Supporting self-management of patients is important from the beginning, meaning right from the start of hospitalization. All practitioners, doctors as well as therapists, need to involve the patient in their treatment. They need to explain, to educate, to train, so patients can take control of their treatment.
**Results**: At the burn center of the Martini Hospital in Groningen, The Netherlands, we involve patients in their treatments and rehabilitation goals. We educate them in their exercise and training program and explain the goals of exercises. We offer different training opportunities. Furthermore, we discuss post clinical rehabilitation settings. Patients are thus more aware about their current situation, learning about their limitations and about their possibilities. Furthermore, we give them freedom of choice, as far as possible, and motivate them. We need to involve all the patient in the burn centre to take control over their life, from all different ages, levels and nationalities. The care professional needs to give or take more responsibility for the treatment, depending on the situation and patient’s wishes, possibilities. Patient as well as practitioners, need to make responsible choices.
**Conclusions**: To give patients control over their treatment and to take control over their lives, they need to participate in their care and take responsibility for their own health. Practitioners must create the necessary conditions to do so. This has to start right at the start of the hospitalization.

*O.042*

**Aquatic Exercise Therapy after Burn Injury—An Implementation Project**

**Saskia J. M. Sizoo ^1^, Karin Bouwmeester ^2^, Ymke Lucas ^1^, Sonja M. H. J. Scholten-Jaegers ^3^, Marianne K. Nieuwenhuis ^4,5,6^ and Moniek Akkerman ^3,4^**
  ^1^  Burn Centre Maasstad Hospital
  ^2^  Department for Physical Therapy, Martini Hospital
  ^3^  Burn Centre Groningen, Martini Hospital
  ^4^  Association of Dutch Burn Centres, Burn Centre Groningen, Martini Hospital
  ^5^  University Medical Center Groningen, Department of Human Movement Sciences, University of Groningen
  ^6^  Research Group Healthy Ageing, Allied Health Care and Nursing, Hanze University of Applied Sciences

**Objectives**: In a recent study in the Dutch burn center Rotterdam, aquatic exercise therapy proved to be a feasible rehabilitation mode for patients with burns, which is more comfortable for patients than regular exercise therapy on land [1]. It allowed patients to move more freely during therapy. This is favorable, as patients may achieve greater ranges of motion and more intensive cardiovascular training in water compared with exercise therapy on land. Based on the very positive experiences in Rotterdam, we would like to enable aquatic exercise therapy after discharge for all Dutch burn patients who could benefit.
**Methods**: Aquatic exercise therapy is indicated for patients with healed burn wounds, who show fear of movement, obvious movement limitations/contractures, and/or a substantial loss of physical fitness after hospital discharge. As none of the three Dutch burn centers has an in-hospital swimming pool, two alternative options are being explored. Patients admitted to a burn center in Rotterdam attend 1–4 introduction sessions of aquatic exercise therapy, provided by the physical therapist from the burn center, in a swimming pool close to the hospital. after these sessions, patients are encouraged to continue aquatic exercise on their own. Patients admitted to a burn center in Groningen are referred to a physical therapist that offers aquatic exercise therapy in their own home environment. The feasibility of both options is explored, including facilitators and barriers. In a later phase of the project, the effectiveness of aquatic exercise therapy on patient-relevant outcome parameters will be structurally evaluated. Possibilities for reimbursement will be explored as well.
**Results (preliminary)**: In Rotterdam, two patients attended the introduction sessions. Both were very enthusiastic regarding aquatic exercise therapy and continued aquatic exercise on their own. Their fear of movement disappeared, both patients regained confidence in their own body, and their mobility improved. In Groningen, no patients were able to attend aquatic exercise sessions yet. Experienced barriers were long-lasting rest defects (*n* = 2), costs (*n* = 1), and aquatic exercise sessions not offered at a convenient time (*n* = 1). Inclusion is on-going.
**Conclusions**: With this implementation project, we hope to gain insight in the feasibility of both options for aquatic exercise therapy, to solve most barriers, and to demonstrate the effectiveness of aquatic exercise for patients after burn injury. This will hopefully lead to a more prominent place for aquatic exercise therapy in burn rehabilitation, including opportunities for (partial) reimbursement.

**Reference**
  1. Sizoo, S.J.M.; Akkerman, M.; Trommel, N.; Esser, J.; Veen-van der Velden, M.; Oen, I.; van der Vlies, C.H.; van Baar, M.E.; Nieuwenhuis, M.K. Feasibility and acceptability of aquatic exercise therapy in burn patients—A pilot study. *Burns Open* **2021**, *5*, 10–20. https://doi.org/10.1016/j.burnso.2020.10.001.

*O.043*

**A Field Training Course for the Rehabilitation of Severe Burn Victims, from the Acute Phase to the Scarring Phase: A Response for the Use of Rehabilitation Management in the Area**

**Daniela Arena ^1^, Danila Toscano ^1^, Lucia Troilo ^1^, Lorena Sarzi ^1^, Maurizio Stella ^2^, Loredana Cozzolino ^3^, Antonio Saldi ^4^, Giuliana Centini ^5^, Laura Clarici ^6^, Nadia Depetris ^2^ and Giuseppe Massazza ^1^**
  ^1^  Orthopedic and Trauma Center (CTO), Città Della Salute e Della Scienza, Physical Medicine and Rehabilitation University (MFRU)
  ^2^  Burn Center, Orthopedic and Trauma Center (CTO), Città Della Salute e Della Scienza
  ^3^  Human Resources Management, Training Department Organization, Secretary Office, Città della Salute e della Scienza
  ^4^  Human Resources Management, Training Department Organization, Training Course Designer, Città della Salute e della Scienza
  ^5^  MSN, RN, Past Director of Continuing Medical Education Center, Città della Salute e della Scienza
  ^6^  Human Resources Management, Training Department Organization -Training Sector Coordinator -Città della Salute e della Scienza

**Targets**: Upon discharge for m the Burn Centre, the patient undergoes an intense and prolonged process of rehabilitation. The patient often does not find an adequate and uniformed response from the service provider. As a result, since 2015, a training course in the field (FSC- for mazione sul campo) has been provided, intended for physiotherapists in burn centres, plastic surgery departments, residential and outpatient rehabilitation facilities, in order to:
  - Promote the dissemination of knowledge in relation to the rehabilitation of the burn victim.
  - Respond to the growing need for a revision and updating of practise, uniformed and specialised, throughout the sector.
  - Create a widespread network of health care professionals, experienced in the evaluation and rehabilitative treatment of burn pathology and scarring.

**Methods**: The ‘FSC’ is an individualised style of training, lasting for four days for a total of 32 h, which consist of 4 theoretical lessons.

The activities undertaken throughout each day of training are described in the detailed time schedule. The project includes the input from various professions.
  - A scientific manager (physiotherapist coordinator).
  - A training planner.
  - An administrative contact.
  - A total of 4 teachers (plastic surgeon, physiatrist, tutor/physiotherapists experienced in the rehabilitation treatment of patients with severe burns and extensive scars).
  - The teacher trainer ratio is 1/3.

**Results**: Overall, 15 editions of the above-described training took place between November 2015 and June 2021 with 30 Physiotherapists trained. At the end of the course, the students were evaluated by a practical test, and all the learners developed the expected skills. A satisfaction questionnaire reported that the participants responded well, with high levels of satisfaction. Furthermore, the questionnaire will provide an evaluation of the relapse in the rehabilitation treatment of extensive scars.
In addition, the ‘FSC’ has allowed:
  - The implementation of a local network of physiotherapists who are expert in the evaluation and rehabilitation treatment of burn pathology and scarring.
  - The continuous discussion, with a dedicated ‘chat’, between experiences and trained physiotherapists on numerous clinical cases and collaboration in identifying the best avenues of care for patients being discharged from different burn centres in various regions in the country.

**Conclusions**: This training model can be replicated in other burn centres to train health care professionals locally, spreading knowledge on the treatment of burn patients and patients with extensive scars as recommended following international guidelines.

*O.044*

**Toxic Epidermal Necrolysis Specific Modified SCORTEN Used for Accurate Prediction of Mortality: A Ten-Year Single-Center Study**

**Zahra Haghani Dogahe ^1^, Shahin Hallaj ^2^ and Mohammadreza Mobayen ^1^**
  ^1^  Burn and Regenerative Medicine Research Center, Guilan University of Medical Sciences, Guilan, Iran
  ^2^  Glaucoma Research Center, Wills Eye Hospital, Philadelphia, PA 19107, USA

**Objectives**: SCORTEN has been introduced as a reliable scale for predicting poor prognosis among TEN-spectrum-afflicted patients. However, lack of a unique scoring scale for TEN patients represents the need for modification in SCORTEN, to enhance its efficacy mentioned in a couple of studies. The aim of this study was to determine a modified scoring scale for TEN patients.
**Methods**: This is a retrospective cross-sectional study, collecting data about TEN patients from 1 March 2010 to 1 March 2020. Here, we present a single center study of patients referring to burn referral center of Guilan province. All patients diagnosed with TEN syndrome and admitted to Velayat burn center, were retrospectively extracted from the Hospital Information System (HIS). Primary outcome measurement was to identify the efficacy of SCORTEN in TEN patients.
**Results**: Overall, 51 TEN patients were selected with the mean age of 39.16 ± 22.67 and a 2:3.1 male to female ratio. Blood glucose levels, and tachycardia did not show a significant difference between the two groups of expired and discharged from TEN syndrome (>30% TBSA). ESR, BE, platelet counts, and creatinine levels showed significant differences between the two groups. Moreover, SCORTEN demonstrated 71.16% efficacy (*p*-value < 0.05) in our study, and therefore, we have modified it to achieve 91.67% accuracy (*p*-value < 0.0001) with specific modified SCORTEN for TEN.
**Conclusions**: Proposed TEN-specific modified SCORTEN can be used as an accurate and optimized indicator of poor prognosis among TEN patients.

*O.045*

**Necrotizing Fasciitis: 10 Years of Experience at Coimbra’s Burn Centre**

**Inês Catalão, José Miguel Azevedo, Gonçalo Tomé, Dmitry Shelepenko, Sara Ramos and Luís Cabral**
  Department of Plastic Surgery and Burns Unit, Coimbra University Hospital Centre

**Objectives**: Necrotizing fasciitis is a rapidly progressive soft tissue infection, that, although uncommon, it is a true medical emergency. The aim of this study was to describe the experience of a Burn Centre in treating patients with necrotizing fasciitis, during a consecutive ten-year period.
**Methods**: The sample is composed of all patients with necrotizing fasciitis admitted to the Burn Centre of Coimbra’s University Hospital, from January 1, 2011, to December 31, 2021. Demographic and clinical data, including gender, age, location of infection, etiology, length of stay, predisposing factors, microbiological results, surgical reconstructive techniques, complications and outcomes, were retrospectively analysed.
**Results**: In total, 10 cases were admitted: 5 (50%) males and 5 (50%) females; mean age was 74.6 ± 9.4 years; mean length of stay was 32 ± 20.7 days and mortality was 10%. Arterial hypertension and diabetes mellitus were the most frequent comorbidities, and the most common etiologies were traumatic (30%) and idiopathic (30%). In total, 50% of patients had lower limb infection. Streptococcus pyogenes was the most frequently identified microorganism in cultures. All patients underwent surgical debridement, with a mean number of interventions of 2.6 ± 1.0. 90% of the patients underwent skin autografts and 20% underwent reconstruction with flaps. A total of 4 (40%) patients had postoperative complications, but they had an adequate outcome.
**Conclusions**: Necrotizing fasciitis is a potentially fatal infection that requires early diagnosis and appropriate treatment; therefore, a high index of suspicion is essential. Radical and early debridement, as well as antimicrobial and supportive therapy are the mainstay of necrotizing fasciitis management. It is desirable that these patients are treated in multidisciplinary units, capable of supporting critically ill patients, such as Burn Centres, in order to reduce mortality rate and complications associated with this infection.

*O.046*

**The Impact of Necrotizing Soft Tissue Infections: A Qualitative Study**

**Jaco Suijker ^1,2^, Annebeth Meij-de Vries ^2,3^, Matthea Stoop ^3^, Anouk Pijpe ^2,3^, Anita Boekelaar ^3^, Marthe Egberts ^1^ and Nancy Van Loey ^1^**
  ^1^  Association of Dutch Burn Centres
  ^2^  Amsterdam UMC
  ^3^  Red Cross Hospital

**Objectives**: Necrotizing Soft Tissue Infections (NSTI) are severe infections marked by local tissue destruction and systemic sepsis, which require aggressive treatment and often prolonged hospitalization. after discharge, a long recovery trajectory awaits most patients. To improve long-term quality of life, it is important to understand the challenges patients face in the acute phase, as well as in the long term.
**Methods**: Thematic analysis was applied to semi-structured interviews with 25 NSTI survivors, of which 14 participated in two focus groups and 11 in interviews.
**Results**: The mean age of the participants was 49 years, mostly (56%) female. The mean time since diagnosis was 5 years, with a range from 6 months to 17 years. Participants reported that NSTI was often not recognized, which resulted in misdiagnosis. Once in hospital, many were severely ill. Treatment was perceived as heavy due to pain, wound care procedures and the life-threatening situation. The disease also had a major impact on family members. Many experienced long-term physical consequences such as severe scarring, cognitive impairment, fatigue and recurrent infections. Psychological consequences related to adjusting to an altered appearance, coming to terms with a life-threatening disease, and fear for relapse. Changes in social circles, lack of understanding and staring were among social consequences. for some, the disease affected vocational activities, causing financial problems. Factors facilitating recovery were social support and putting into perspective. Aftercare may be improved regarding care transition, taking the patient’s perspective and information provision.
**Conclusions**: This study reveals that NSTI is a disease with a large impact on the physical and psychosocial wellbeing of the survivors and their relatives. In order to improve outcomes, a multi-disciplinary approach is required, and clinicians should actively involve patients, use open communication, and provide clear and reliable information. Additionally, this study reveals which domains should be included in future quantitative, patient-reported outcome-measure-based quality-of-life studies.

*O.047*

**Approaches to Surgical Debridement in Necrotizing Soft Tissue Infections: Outcomes of an Animated, Interactive Survey**

**Jaco Suijker ^1,2,3^, Fabienne Hofmans ^3^, Paul van Zuijlen ^2,3^, Huib Cense ^3^, Jaap Bonjer ^2^ and Annebeth Meij-de Vries ^2,3^**
  ^1^  Association of Dutch Burn Centres
  ^2^  Amsterdam UMC
  ^3^  Red Cross Hospital

**Objectives**: Necrotizing soft tissue infections (NSTI) affect long-term quality of life in survivors. Different approaches to debridement may influence quality of life. The aim of this study was to assess the current practice of the debridement of NSTI in The Netherlands.
**Methods**: An animated, interactive online survey was distributed among general surgeons and plastic surgeons in The Netherlands. Two NSTI cases were presented, followed by questions regarding the preferred surgical approach. Case one described a woman with a swollen, red leg, with signs of sepsis and without visible necrosis. Case two described an immunocompromised man with septic shock syndrome and extensive necrosis.
**Results**: In total, 232 responses were included (143 general surgeons, 89 plastic surgeons). In case one, 32% chose to preserve all skin, whereas 17% chose to resect all skin above the affected fascia, including normal-looking skin. In case two, all participants resected necrotic skin, and most (88%) also resected blue discolored skin. Although 32% did not resect more than blue discolored and necrotic skin, 35% also resected red-colored skin, and 21% resected all skin overlying the affected fascia, including normal-colored skin. Respondents working in a hospital with a burn center tended to preserve more skin, whereas plastic surgeons chose skin resection compared with general surgeons.
**Conclusions**: Through a novel approach to a survey, the authors demonstrate the existence of extensive practice variety regarding the approach to debridement of NSTI among Dutch general and plastic surgeons. Consensus is needed, followed by the targeted education of surgeons.

*O.048*

**Clinically Important HLA Alleles in Severe Systemic Hypersensitivity Reactions to Drugs**

**Roberta Verrua ^1^, Silvia Deaglio ^2^, Paola Magistroni ^2^, Gina Adriana Mazzola ^2^, Monica Berrino ^2^, Angelo Faini ^2^, Giulia Brach del Prever ^2^, Maurizio Stella ^1^ and Antonio Amoroso ^2^**
  ^1^  AOU Città della Salute e della Scienza di Torino, SC Centro Grandi Ustionati
  ^2^  AOU Città della Salute e della Scienza di Torino, SC Immunogenetica e Biologia dei Trapianti

**Objectives**: Genetic polymorphisms of HLA vary widely at a population level and are responsible for developing severe cutaneous adverse drug reactions (SCARs) such as Stevens–Johnson syndrome (SJS), toxic epidermal necrolysis (TEN), drug reaction with eosinophilia and systemic symptoms (DRESS) and acute generalized exanthematous pustulosis (AGEP). Specific human leukocyte antigen (HLA) alleles are well-established biomarkers for abacavir, carbamazepine, and allopurinol hypersensitivity. However, the cost-effectiveness of genotype-guided prescribing of these medications has only been evaluated in the United States and few countries across Europe and Southeast Asia. The aim of this study is typing patients with these ADRs and produce a report and related genetic counseling in order to provide useful information on the harmful and unwanted effect resulting from the use of drugs.
**Methods**: The burn centre, the reference unit of the GRESIF project (severe systemic hypersensitivity reactions to a drug), in collaboration with the Department of Immunogenetics of the University of Turin, started a study for the HLA typing of patients with severe skin ADRs. To activate the procedure, our team has prepared consent and information on to the processing of genetic data and the method of sending the sample. Territorial health facilities have received the documentation with the support of pharmacovigilance managers. An EDTA blood sample was collected from each subject, analyzed, and then reports and related genetic counseling were provided on the base of ADRs and culprit drug.
**Results**: From 2020 to 2022, we enrolled 24 patients consequently tested in the lab: 13 TEN, 8 SJS, 2 DRESS and 1 AGEP. In total, 12 patients were males and 12 were females. The mean age was 65.1 ± 18.6. Overall, 20 patients had Caucasian ethnicity, 3 had African and 1 had Asian ethnicity.
There is no statistically significant difference between the mean age of male (61.5 ± 17.4 years) and females (68.6 ± 19.7 years) and no frequency difference between males (50%) and females (50%).
The culprit drugs were identified based on the guidelines: allopurinol, antibiotics and carbamazepine were drugs most frequently involved. The results showed that 7 patients who received allopurinol were all positive to HLA B 58:01.
**Conclusions**: HLA allele screening represents an important means to prevent drug-induced SCARs. Further research should explore ethnic variability in genetic testing and different drugs and classes of drugs; studies should report on incidence and long-term sequelae in drug-hypersensitivity survivors.

*O.049*

**A Combined Protocol of Plasmapheresis and IVIGs Reduces Mortality in TEN**

**Agnieszka Surowiecka, Tomasz Korzeniowski, Jerzy Struzyna**
  East Center of Burns Treatment and Reconstructive Surgery

**Objectives**: There are no unequivocal treatment guidelines in TEN. Immunosuppressive treatment may increase the wound infection risk and mortality, thus immunomodulatory treatment should be applied. Dermoepidermal cytotoxic T-lymphocytes are responsible for dermal infiltration in a non-immediate type-IVc hypersensitivity reaction. Plasmapheresis within the first days in TEN purges the causative residual drug and inflammatory cytokines, including the Fas ligand. IVIGs inhibit cell lysis by binding the Fas ligand.
**Methods**: A retrospective analysis of 35 patients with diagnosed in histopathology TEN was performed. Our protocol included a cycle of plasmapheresis with frozen fresh plasma twice daily for the first 2 days following admission, and once daily for the subsequent 5 to 7 days. IVIGs were administered after the first two sessions of plasmapheresis, for 4 to 7 days. The dosage was calculated according to body weight, at 0.4 to 0.5 g/kg.
**Results**: The mean age of all the patients was 50.7 [range 14–82, SD 19.54]. There were 7 (20%) patients with a history of neoplasm in the cohort. The mean extent of lesions was 73.8% [range 24–100, SD 24.26]. 67.74% of the patients requires catecholamines and 70.97% who underwent mechanical ventilation during hospitalisation. Administration of the catecholamines increased the mortality risk (*p* = 0.015). The estimated at the admission mortality was 41.9%, whereas the actual mortality rate observed was 12.5%. Our protocol improved the survival, OR = 26.57, RR = 6.4, *p* = 0.022.
**Conclusions**: Our study proved that simultaneous plasmaphereses with IVIGs administration is a safe method and improves patients’ outcome in TEN. Immunomodulatory treatment aim is to remove the causative agents and to gain immune tranquility. Early aggressive plasma purification is necessary to eliminate proinflammatory agents, FAS ligand, drug or drug metabolites and to prevent serious multiorgan complications. IVIGs are responsible for binding the FAS ligand and blocking progressive epidermal necrosis.

*O.050*

**The Experience of Burn Center Units into the GRESIF Study in the Management of Stevens–Johnson Syndrome/TEN**

**Roberta Verrua ^1^, Chiara Gamba ^2^, Maria Teresa Fierro ^3^, Elena Marra ^3^, Eleonora Marrazzo ^4^, Elisabetta Geninatti ^4^, Maria Concetta Bilancio ^5^, Olivia Leone ^6^, Maurizio Stella ^1^ and Jan Schroeder ^2^**
  ^1^  AOU Città Della Salute e Della Scienza di Torino, SC Centro Grandi Ustionati
  ^2^  ASST Grande Ospedale Metropolitano Niguarda Allergologia e Immunologia
  ^3^  AOU Città Della Salute e Della Scienza, SC Dermatologia Universitaria
  ^4^  ASL Città di Torino
  ^5^  Farmacovigilanza Direzione Generale Welfare, Regione Lombardia
  ^6^  Struttura Epidemiologia e Valutazione Delle Performance, Direzione Generale Welfare, Regione Lombardia

**Objectives**: Stevens–Johnson syndrome (SJS), toxic epidermal necrolysis (TEN) and SJS–TEN overlap are severe mucocutaneous conditions, associated with significant morbidity and mortality. Treatment is based on the suspension of the culprit drug and on beginning of an optimal supportive treatment in a specialized centre as burn centre. However, the use of which specific treatment and what dose for which patient with SJS/TEN are issues that are the subject of ongoing debate among clinicians. The objective of the study is the harmonization of diagnostic–therapeutic procedures to improve the clinical management of patients in order to develop a prognostic index applicable in clinical practice.
This pharmacovigilance project, based on AIFA funding, is named GRESIF (severe systemic hypersensitivity reactions to a drug).
**Methods**: To obtain an early diagnosis of the disease and prompt therapy, we have organized a surveillance network starting from the coordinating burn centres, involving the territorial health facilities in Northwest Italy. To ensure an optimal diagnosis and prognosis process, as well as the treatment of sequelae, we have drawn up guidelines.
**Results**: From 2019 to 2021, 50 reports were collected. Patients were treated in burn centres (16 patients) or in an outpatients’ clinic under a coordinated management of their clinical course.
Unlike burn guidelines, which recommend the prompt debridement of detached epidermis, our clinicians favor a conservative approach to preserve detached epidermis as a biologic dressing, reflecting the different underlying mechanisms involved with SJS/TEN and burn injury. Antishear strategies, such as limiting dressing changes, using an air-fluidized bed, and selecting nonadherent dressings, are recommended. General management focuses on supportive care: fluid balance, prevention of infective complications, maintenance of airway and renal function, pain control and nutritional support.
Although supportive care represents the most important treatment for SJS and TEN management, other therapies are often prescribed as treatment by means of IVIG 0.75 g/kg/day for 4 days, based on the inhibition of cell lysis via binding of FAS–ligand interaction.
**Conclusions**: The implementation of shared diagnostic and therapeutic protocols to be applied promptly to reduce both mortality and long-term sequelae is mandatory to manage these extremely rare but serious reactions.

*O.051*

**Nonburn Patients in Burn Units: An 11-Year Burn Centre Experience**

**José Miguel Azevedo ^1^, Arnaldo Figueiredo ^2^, Gonçalo Tomé ^1^, Dmitry Shelepenko ^1^, Inês Catalão ^1^, Susana Pinheiro ^1^ and Luís Cabral ^1^**
  ^1^  Department of Plastic Surgery and Burns Unit, Coimbra University Hospital Centre
  ^2^  Department of General Surgery, Leiria Hospital Centre

**Objectives**: Burn units’ main purpose is to treat severe burn injuries. However, due to the characteristics of these specialized units, there are other diseases with loss of cutaneous tissue that benefit from hospitalization and treatment directed at burn units. The aim of this study is to characterize these patients and try to better understand the role of burn units in their treatment and outcomes.
**Methods**: We carried out a descriptive series of patients admitted to our burns unit with injuries not related to burns admitted from January 2011 to December 2021.
**Results**: during the analysis period, 74 unburned patients were admitted, aproximatedly corresponding to 1 in each 20 patients. The male/female ratio was 1,06 and the average age was 64.95 years. The average length of stay was 26.89 days, with a mortality rate of 31.1%.
Of these patients, 37 (50%) were admitted for Lyell’s Syndrome, 14 cases (18.9%) for trauma injuries from different body segments, 9 cases (12.2%) for necrotizing fasciitis (including a Fournier’s gangrene case), 6 cases (8.1%) for SSTJ/TEM overlap syndrome, 4 cases (5.4%) for Stevens-Johnson Syndrome and 4 isolated cases for other rare diseases (1 pityriasis lichenoides et varioliformis acute (PLEVA), ulceronecrotic variant of Mucha-Haberman, 1 subacute cutaneous lupus, 1 epidermolysis bullosa and 1 acute generalized exanthematous pustulosis).
The treatment included balneotherapy sessions, dressing care and supportive medical therapy for all patients. In addition, orotracheal intubation and invasive mechanical ventilation was required in 27 patients (36.5%) and aminergic support in 24 cases (32.4%), most of these had Lyell’s Syndrome. The mortality rate of Lyell’s Syndrome was 48.64%, accounting for 18 of the 23 deaths registered in nonburn patients. Surgical treatment was required in 28 cases (37.8%), mainly on trauma injuries and necrotizing fasciitis.
**Conclusions**: Burn units combine specialized wound care and critical care. Equipped with their own operating room, anesthesia and surgical team, patients can be submitted to several surgical interventions as needed in the appropriate timings. The aseptic environment, experience in infection control and rational use of antibiotics are essential, attending to the break of the skin barrier. for all these reasons, burn units are the best option in selected non-burn severe cases of cutaneous loss of substance. It is important for health professionals to be aware of this resource in order to optimize care and improve the prognosis of these critically ill patients, as well as optimizing the criteria for referring non-burned patients to burn units.

*O.052*

**Monocyte- and Neutrophil Extracellular Traps Are Present in the Dermal Microvasculature of Burns Wounds and Coincide with a Procoagulant Phenotype**

**Britt van der Leeden ^1,2^, H. Ibrahim Korkmaz ^3,4,5,6^, Bouke Boekema ^3,6^, Chopie Hassan ^7^, Paul van Zuijlen ^3,5,6^, Hans Niessen ^1,8,9^, Susan Gibbs ^4,10^ and Paul Krijnen ^1,9^**
  ^1^  Department of Pathology, Amsterdam University Medical Centers (AUMC)
  ^2^  Amsterdam Infection & Immunity, AUMC
  ^3^  Association of Dutch Burn Centers (ADBC)
  ^4^  Department of Molecular Cell Biology and Immunology, Amsterdam UMC, Vrije Universiteit
  ^5^  Burn Center, Red Cross Hospital
  ^6^  Department of Plastic, Reconstructive and Hand Surgery, AUMC
  ^7^  Pharming Technologies B.V.
  ^8^  Department of Cardiac Surgery, AUMC
  ^9^  Amsterdam Cardiovascular Sciences, AUMC
  ^10^  Department of Oral Cell Biology, Academic Centre for Dentistry Amsterdam (ACTA), University of Amsterdam and Vrije Universiteit

**Background**: Burn wound conversion is a post-burn phenomenon where vital tissue is lost to due to the expansion of necrosis. Loss of perfusion in the burn wound may be an important underlying cause of burn wound conversion and is induced by microvascular damage, persisting inflammation and post-burn coagulopathy. Previously we showed that neutrophils extracellular traps (NETs) are present in microcirculatory thrombi of burn wounds and a switch in the microcirculatory endothelium toward a procoagulant phenotype.
**Objectives**: The aim of this study was to investigate the presence of monocyte extracellular traps (METs) and NETs in the burn wound as well as the local and systemic pro-coagulant and inflammatory phenotype in the microcirculatory endothelium after burn injury.
**Methods**: Eschar was operatively obtained from burn wound patients (*n* = 21) with a mean total burned body surface area (TBSA) of 29%. Herein, the coagulation factors, i.e., tissue factor (TF) and factor XII (FXII) together with the endothelial cell marker CD31 were studied using immunohistochemistry. The presence of NETs and METs was analyzed via immunofluorescence, combining specific immune cell markers myeloperoxidase (MPO: NETs) and CD14 (METs) together with endothelial cell marker CD31 and the extracellular trap (ET) marker histone 3 citrullin. To assess inflammatory and procoagulatory systemic effects, plasma was obtained from burn patients (*n* = 7) with a mean TBSA of 35%. Herein, the levels of neutrophil activation marker (HNE-a1ATC), DNA cleaving protein (DNAse1), nucleosome complex for extracellular DNA, and the coagulatory marker prothrombin factor 1.2 were determined.
**Results**: Increased expression of TF and FXII was found intravascular in all eschar samples compared with uninjured skin from abdominoplastic surgery. Neutrophils were the most predominant cell type of the immune cell infiltrate in eschar. Both NETs and METs were found in the lumen of the dermal microvasculature in the eschar tissue 7 up to 40 days post-burn. Significantly increased plasma levels of HNE-a1ATC, DNAse1 and nucleosome complex were found from day 1 up to 15 days post-burn compared with plasma obtained from healthy donors.
**Conclusions**: This study shows that although neutrophils are the prominent source of extracellular traps in eschar, monocytes also contribute to post-burn ETosis and may thereby contribute to the hyper-coagulatory state after burns and burn wound conversion. This coincides with a systemic increase in nucleosomes, DNAse1 and neutrophil activation after burn injury, thereby indicating the systemic presence of neutrophil activation and possible ETosis after burn injury.

*O.053*

**Investigating the Molecular and Cellular Pathophysiology of Heterotopic Ossification after Burn Injury**

**Nichola Foster ^1,2^, Lucy Barrett ^2,3^, Mark Fear ^2^, Andrew Stevenson ^2^, Nathan Pavlos ^4^, Dale Edgar ^1,2,5^, Fiona Wood ^2,5^, Edward Raby ^2,5^, Frank Li ^6^, Jason Brown ^7^, Leila Cuttle ^8^ and Aleksandra Edmundson ^7^**
  ^1^  Burn Injury Research Node, Institute for Health Research/School of Physiotherapy, The University of Notre Dame
  ^2^  Burn injury Research Unit and Fiona Wood Foundation, University of Western Australia
  ^3^  Telethon Kids Institute
  ^4^  School of Biomedical Sciences, University of Western Australia
  ^5^  State Adult Burns Unit, Fiona Stanley Hospital
  ^6^  Physiotherapy Department, Concord Repatriation General Hospital
  ^7^  Professor Stuart Pegg Adult Burns Centre, Royal Brisbane and Women’s Hospital
  ^8^  Queensland University of Technology, at Centre for Children’s Health Research

Heterotopic ossification (HO) is a debilitating complication of burn and trauma. However, the cellular and molecular mechanisms underlying HO remain unclear; therefore, treatment of HO is limited. Interstitial fibroblasts, not cardiomyocytes, undergo differentiation towards an osteogenic phenotype and drive myocardial calcification in a variety of heart disease conditions. We hypothesised that, similarly, burn patients that develop HO after injury have a dermal fibroblast phenotype that is more susceptible to osteogenic differentiation.
**Objectives**:
  1. Identify the key genes and signalling pathways that are different in fibroblasts from patients that develop HO after a burn injury compared with those that do not develop HO after a burn.
  2. Determine whether fibroblasts from burn injured patients that develop HO are more susceptible to differentiating to an osteoblastic phenotype compared with control fibroblasts.

**Methods**: The study group (HO+, *n* = 9) consisted of burns patients diagnosed with HO, while the control group (HO−, *n* = 4) consisted of burns patients without a confirmed diagnosis of HO and matched by age, gender and total burn surface area. Fibroblasts from 3mm skin punch biopsies were cultured for RNA extraction. RNA-sequencing and analysis was performed to compare the global gene expression profiles of fibroblasts between HO+ and HO− subjects. To confirm the RNAseq data, differentially expressed genes (DEGs) were selected and tested using Flow Cytometry and quantitative real-time polymerase chain reaction (qRT-PCR). Osteogenic differentiation assays were also conducted using fibroblasts from HO+, HO− and uninjured humans, with alkaline phosphatase activity and mineralisation quantified.
**Results**: A total of 136 significant DEGs were identified between HO+ and HO− subjects, of which 29 were upregulated, and 107 genes downregulated. Gene Ontology analysis revealed that upregulated genes were significantly enriched in biological processes including cell morphogenesis involved in neuronal differentiation and the Wnt signalling pathway. Pathway analysis revealed that upregulated genes were primarily associated with pathways of neurodegeneration. Preliminary data from osteogenic differentiation assays indicated that fibroblasts from HO+ patients exhibit more alkaline phosphatase activity and mineralisation compared with controls.
**Conclusions**: Fibroblasts from HO+ patients appear different in their transcriptome compared with controls. Preliminary functional analysis suggests they are more susceptible to osteogenic differentiation. Further validation of these gene targets using functional studies is necessary and continuing. However, the data suggest a predisposition in some patients to HO after burns that may be identified and ultimately prevented in the future.

*O.054*

**Burn Injury Causes Long-Lasting Influx of Neutrophils, Release of Pro-Inflammatory Cytokines and Shifts in T Cell Composition in Blood and Burn Tissue from Patients**

**Patrick Mulder ^1,2^, Marcel Vlig ^1^, Esther Fasse ^2^, Bram van Cranenbroek ^2^, Matthea Stoop ^3^, Anouk Pijpe ^3^, Paul van Zuijlen ^3,4,5,6^, Evelien de Jong ^3,7^, Irma Joosten ^2^, Hans Koenen ^2^ and Bouke Boekema ^1,4^**
  ^1^  Preclinical Research, Association of Dutch Burn Centres
  ^2^  Laboratory of Medical Immunology, Radboudumc
  ^3^  Burn Center, Red Cross Hospital
  ^4^  Amsterdam UMC, Department of Plastic Reconstructive and Hand Surgery
  ^5^  Amsterdam UMC, Paediatric Surgical Center, Emma’s Children’s Hospital
  ^6^  Amsterdam Movement Sciences (AMS) Institute, Amsterdam UMC
  ^7^  Department of Intensive Care, Red Cross Hospital

Burn injury is often followed by an extensive, derailed immune response which is present in burned tissue, as well as in peripheral blood. Regardless of infection, burn patients often suffer from systemic inflammatory response syndrome. Leukocytes in the blood and wound area produce cytokines and growth factors that enforce the inflammation. An aggressive, persistent immune response can delay wound healing, damage the surrounding tissues and ultimately contribute to severe scarring. To prevent such secondary complications and improve wound healing, a better understanding of the underlying mechanisms and interplay of immune cells is essential.
In this longitudinal study, we analyzed the immune profile in blood from patients at various time intervals after injury using flow cytometry and cytokine assays. Similarly, we analyzed the immune cells and cytokines in burn tissue obtained from patients who underwent eschar debridement. Results were compared with blood and skin samples from healthy subjects.
The patient cohort showed signs of systemic inflammation and persistently high levels of c-reactive protein and pro-inflammatory cytokines IL-6, IL-8, MCP-1, MIP-1β, and MIP-3α were measured. We observed continuous high numbers of neutrophils and monocytes in blood for at least 39 days. Increased numbers of CD10- (immature) neutrophils were present in peripheral blood in the first three weeks after injury. Total blood lymphocyte numbers did not increase, but the number of effector T cells as well as regulatory T cells increased from the second week onward. Within the CD4+ T cell population, elevated numbers of T cells with a pro-inflammatory phenotype were found. In burn tissue, we found an immediate increase in mainly CD10+ (mature) neutrophils and macrophages. The number of neutrophils and macrophages was still increased in burn tissue debrided 3 or 4 weeks after injury. Increased lymphocyte numbers were found in burn tissue from two and three weeks after burn injury.
Altogether, these data reveal that burn injury induced a persistent inflammatory response that originated mainly from innate immune cells. The long-lasting release of immature neutrophils and infiltration of neutrophils into the wound upholds the inflammatory state, thereby increasing the risk of secondary complications.

*O.055*

**Impact of Severe Burns on Pancreatic Islets: An Experimental Model in Rats**

**Santiago Santelis ^1,2^, Ebru Abali ^1,3^, Gonca Ozgun ^4^, Handan Ozdemir ^4^, Neslihan Basci Tutuncu ^5^ and Mehmet Haberal ^1,3^**
  ^1^  Burn Center and Burn and Fire Disasters Institute, Baskent University
  ^2^  Research Department, Benaim Burn Foundation
  ^3^  Department of General Surgery, Faculty of Medicine, Baskent University
  ^4^  Department of Pathology, Faculty of Medicine, Baskent University
  ^5^  Department of Endocrinology and Metabolism, Faculty of Medicine, Baskent University

**Introduction**: Severe burn victims experience a systemic inflammatory response and a hypermetabolic response that can generate adverse effects on many distant organs and systems. The underlying mechanisms of beta cell failure in burn patients with acquired insulin resistance after a burn trauma is not well understood. Our aim in this study was to describe the histopathological changes in the pancreatic islets secondary to severe burns in an experimental animal model.
**Materials and Methods**: Fourteen Wistar albino rats were randomly divided into two groups: the sham group and the burn only group. A full thickness burn model was designed to induce a burn of 25% of the total body surface area. Seven days after burn induction, a pancreatectomy was performed. Pancreatic tissues were examined under light microscopy, and islet size and cellularity were calculated by digital imaging analysis.
**Results**: The histopathologic examination was unremarkable, but the mean number of islets per pancreatic tissue was lower in the burn group than that in the sham group. We observed a significant difference in the mean number of cells per one islet between the two groups, with the cell count higher in the burn group (*p* < 0.05).
**Conclusions**: during the acute phase of burn injury in rats, we observed a decrease in the number of pancreatic islets with remarkable hypercellularity. It is important to devote efforts to understand, prevent, attenuate, and control the transient and permanent impact of severe burns to pancreas not only to improve patients’ survival rate, but to provide a good quality of life for severe burn survivors.

*O.056*

**An Assessment of Pressure Delivery Beneath Negative Pressure Wound Therapy Utilising a Cadaveric Porcine Model**

**Emma Lumsden ^1,2,3^, Roy Kimble ^1,2,3^, Bronwyn Griffin ^1,2,3^ and Robert Ware ^1,2,3^**
  ^1^  Queensland Children’s Hospital
  ^2^  Centre for Children’s Health Research
  ^3^  Griffith University

**Objectives**: Negative pressure wound therapy (NPWT) is broadly used in surgical wound management; however, there remains a debate around the mechanism of action and application within burn care. This study was done to help understand the pressure delivered to tissue beneath NPWT when varying pressures, layers of kerlix and distribution of dressings are applied to a burn.
**Methods**: Utilising a cadaveric porcine model, a Codman intracranial pressure (ICP) ExpressTM monitor and MicrosensorTM transducer was used to assess pressure. The transducer was inserted under ultrasound guidance via cannulation to the skin, dermis, subcutaneous or muscular layer. Smith and Nephew’s MepitelTM, ActicoatTM, varying layers of KerlixTM (10, 20 or 30 layers) and NPWT (Smith and Nephew Renays TouchTM) were then applied either circumferentially or non-circumferentially. Each set of results is indicative of the ICP probe reading when NPWT was delivered at −40, −60, −80, −100 and −120 mmHg.
**Results**: The median pressure recordings were skin: −42, −61, −80, −98 mmHg; dermis: 1, 2, 3, 4, 6mmHg (this increase in pressure was less significant when circumferential dressings or 30 layers of Kerlix was applied) and muscular: 0, 0, 0, 0, 0 mmHg. The subcutaneous layer had marked variation with no observed trend.
**Conclusions**: These data suggest negative pressure paradoxically exerts a positive pressure on the dermis. However, circumferential dressings and increased layers of kerlix reduced this positive pressure. This knowledge has impacted our burn NPWT dressing selection at Queensland Children’s Hospital. The limitation of this study is the cadaveric model; a live model is required for future studies.

*O.057*

**Detailed Characterization of a Novel Human Ex Vivo Model for Burn Injuries**

**Elisabeth Hofmann ^1,2^, Ines Foessl ^3^, Anita Eberl ^4^, Eva-Maria Prugger ^4^, Dagmar Kolb ^5^, Hanna Luze ^2^, Simon Schwingenschuh ^4^, Thomas Birngruber ^4^, Martin Funk ^5^, Lars-Peter Kamolz ^1,2^ and Petra Kotzbeck ^1,2^**
  ^1^  Joanneum Research for schungsgesellschaft mbH, Coremed
  ^2^  Medical University of Graz, Division of Plastic Aesthetic and Reconstructive Surgery, Research Unit for Tissue Regeneration, Repair and Reconstruction
  ^3^  Medical University of Graz, Division of Endocrinology and Diabetology
  ^4^  Joanneum Research for schungsgesellschaft mbH, Health
  ^5^  Medical University of Graz, Core Facility Ultrastructure Analysis
  ^6^  Evomedis GmbH

**Objectives**: Burn injuries belong to the most common soft tissue injuries and can not only result in extensive trauma but are also associated with cases of major emergency such as sepsis or systemic inflammatory response syndrome. The early phase after a burn injury is critical and determines later potential complications in wound healing. Burn injuries activate numerous processes, including heat shock, inflammation and tissue regeneration responses and thereby promote the release of cytokines and other signalling molecules such as miRNAs. Nevertheless, skin tissue reactions in the early phases after burn injuries still need to be investigated in more detail. Therefore, reliable burn models are needed to elucidate the exact sequence of events during the healing process and to monitor potential biomarkers that could give information about treatment success such as miRNAs.
**Methods**: We induced contact burns on fresh human abdominal skin explants that were resected during abdominoplasty. Gene and miRNA expression patterns, cytokine production profiles of key mediators such as IL8 and IL6 and skin ultrastructure were analysed for 24 h after the burn injury. To mimic purely inflammation-mediated reactions in comparison to a complex burn injury, we applied the TLR4-agonist lipopolysaccharide (LPS) intradermally. Additionally, we used open-flow microperfusion (OFM), a sampling technique that allows time and location dependent collection of dermal interstitial fluid (dISF), to also monitor the release of specific miRNAs and cytokines.
**Results**: In burn injuries, we found significant changes in gene and miRNA expression as soon as one hour after burn injury. Inflammatory genes such as IL8 were significantly up-regulated, whereas miRNAs were systematically down-regulated. Lipopolysaccharide stimulation initiated an inflammatory response while leaving the expression patterns of heat shock and tissue repair genes unaffected for the duration of the experiment. miRNAs were actively released and mobilized into the dISF, whereas miR-497-5p could be identified stably downregulated in tissue and dISF in the early phase after a burn injury.
**Conclusions**: Using this novel ex vivo human skin model we were able to study the immediate early responses to skin injuries including burns for up to 24 h. We found that miR-497-5p could serve as potential biomarkers to assess burn severity since it is not only down-regulated in skin but also mobilized into the dISF after burn injury.

*O.058*

**Long-Term Mortality and Predictors in Elderly Burn Patients, a 10-Year National Longitudinal Cohort Study**

**C. I. Cords ^1,2,3^, M. E. van Baar ^1,4^, A. Pijpe ^5,6^, M. K. Nieuwenhuis ^7,8,9^, M. H. J. Verhofstad ^3^ and C. H. van der Vlies ^2,3^**
  ^1^  Association of Dutch Burn Centres, Maasstad Hospital
  ^2^  Department of Trauma and Burn Surgery, Maasstad Ziekenhuis
  ^3^  Trauma Research Unit Department of Surgery, Erasmus MC
  ^4^  Department of Public Health, Erasmus MC
  ^5^  Association of Dutch Burn Centres, Red Cross Hospital
  ^6^  Department of Plastic Reconstructive and Hand Surgery, Amsterdam Movement Sciences (AMS) Institute, Amsterdam UMC, Location VUmc
  ^7^  Association of Dutch Burn Centres, Martini Hospital
  ^8^  Research Group Healthy Ageing, Allied Health Care and Nursing, Hanze University of Applied Sciences
  ^9^  University of Groningen, University Medical Centre Groningen, Department of Human Movement Sciences

**Objectives**: Due to an aging population worldwide, the elderly burn patient population is growing. Insight into long-term mortality rates of the elderly patients after burn injury and predictors affecting the outcome is limited. This study aimed to provide such information for this patient population. In addition, standardised mortality rates were calculated for one-year and five-year mortality.
**Methods**: A multicentre observational retrospective cohort study was conducted in all three dedicated Dutch burn centres. Patients aged ≥65 years who had been admitted with burn injuries from 2009 to 2018 were included. Data were retrieved from electronic patient records and the Dutch Burn Repository R3, a registration for Dutch specialised burn care. Mortality rates and standardised mortality rates (SMR) were calculated. Survival analysis (multivariable Cox proportional hazards regression) was used to assess predictors of time to mortality/survival during five-year follow-up.
**Results**: In total, 682 out of 771 patients were discharged alive, and the median age was 74 years (25th–75th percentiles 69.0–81.0). The one-year and five-year mortality rates were 8.1% and 23.4%, respectively. The SMRs were 1.9 (95% CI 1.5–2.5) and 1.4 (95% CI 1.2–1.6), respectively. The SMRs were highest in patients aged 65–74 years (SMR at 5 year 10.1, 95% CI 7.7–13.0). The relative risk of dying up to five years afterwards was predicted by age (HR 1.1, 95% CI 1.0–1.1), multimorbidity (HR 2.3, 95% CI 1.6–3.5) and non-home discharge location (HR 2.1, 95% CI 1.4–3.2). Sex, socio-economic status, mechanical ventilation, ICU admission, length of hospital stay, extent of burn(s), surgical treatment or Revised Baux score were found not to predict risk of dying up to five years after discharge.
**Conclusions**: Long-term mortality after burn injury was higher compared with the age- and sex-matched general Dutch population, especially in the young and the elderly. Predictors for long-term mortality risk were age, multimorbidity and discharge destination. Burn injury or treatment characteristics were not directly associated with an increased risk of dying early. The reason for an increased mortality rate after burn injuries is probably associated with other factors. It could be hypothesized that either pre-injury characteristics as multimorbidity at time of burn injury, psychosocial status and potential long-lasting systemic impacts on the heart and circulation play a role in the increased mortality or that a decreased health status makes patients more prone to burn injuries, leading to early death.

*O.059*

**Patients’ Experience of Care in Dutch Burn Centers and Aftercare: Results from Focus Groups**

**Lotte Van Dammen ^1^, Hendriët Wanders ^2^, Irma Visser ^2^, Inge Spronk ^1,3^, Nina Lansdorp ^4^, Marscha Heijblom ^5^, Matthea Stoop ^6^, Carine van Schie ^1^, Marianne Nieuwenhuis ^7,8,9^ and Nancy van Loey ^3,10^**
  ^1^  Dutch Burns Foundation
  ^2^  Dutch Association of Burn Survivors
  ^3^  Association of Dutch Burn Centers
  ^4^  Department of Plastic Reconstructive and Hand Surgery, Amsterdam UMC Location Vrije Universiteit Amsterdam
  ^5^  Association of Dutch Burn Centers, Maasstad Hospital
  ^6^  Association of Dutch Burn Centers, Red Cross Hospital
  ^7^  Association of Dutch Burn Centers, Burn Center Martini Hospital Groningen
  ^8^  Research Group Healthy Ageing, Allied Health Care and Nursing, Hanze University of Applied Sciences Groningen
  ^9^  Department of Human Movement Sciences, University Medical Center Groningen, University of Groningen
  ^10^  Department of Clinical Psychology, Utrecht University

**Objectives**: An increasing body of knowledge documents the quality of care and aftercare for patients with (severe) burn injuries from the professionals’ perspective. However, there is limited evidence regarding quality of care and aftercare from the patients’ perspective. The aim of this study was to identify factors that were important for Dutch patients with burns at the time of arrival, during hospital admission, and throughout the aftercare process.
**Methods**: Patients with burns and parents of pediatric burn patients were asked to participate in a focus group, to talk about their experiences with burn care. Within the group that was invited to participate (*n* = 24), a total of nine patients, and one parent of a pediatric burn patient, were willing to participate. The focus groups were part of the Highly Specialized Burn Care, Education and Research Program within Dutch burn centers, which is aimed at the development of a sustainable value-based healthcare framework. Topics discussed during the first (*n* = 7) and second (*n* = 3) focus group were summarized.
**Results**: Participants indicated that it was important to have healthcare professionals set clear expectations as soon as possible after arrival in the burn center about the necessity of hospital admission. It is essential for patients and their families to receive information about wound care and side effects such as fever and pain, even if admission is not necessary. These clear expectations, and the need for information, are also important when a decision is made about surgery. Participants also mentioned that it is helpful to receive information about the wound and scar development over time. Patients would like to be involved in pain management and treatment, together with the healthcare professionals; this also helps patients to regain control. Another topic that was raised concerned the first time a patient sees their own wounds, and how it can help to respect the patients’ personal wishes in this process. Finally, participants agreed that contact with other burn survivors and parents of pediatric patients plays a crucial role. Exchanging information and experiences with other survivors is very insightful and helps them cope with the lifelong sequela of burns.
**Conclusions**: Burn patients would like to receive understandable and accurate information about treatment, pain management, wound healing, scar maturation processes and aftercare, and be more actively involved in decision-making. This information fits with the value-based healthcare framework and is important to improve care for burn patients.

*O.060*

**Analysis of Rehabilitation Goals in the Rehabilitation of Burn Victims According to the International Classification of Functioning, Disability and Health—Development of a ICF Core Set**

**Hubert Neubauer, Leila Harhaus, Hans Ziegenthaler, Annette Stolle and Ulrich Kneser**
  BG Klinik

**Objectives**: Physical limitations and complaints remain after a burn injury. Burn-specific rehabilitation is recommended after severe burns. Contemporary rehabilitation concepts are patient-centred and based on the International Classification of Functioning, Disability and Health (ICF). An important element of patient-centred rehabilitation are the rehabilitation goals, which are jointly agreed by the patient and the treatment team in a participative decision-making process at the beginning of the rehabilitation.
**Methods**: In a prospective multicentre trial to investigate the effectiveness of ICF-oriented rehabilitation concepts for burn patients, the rehabilitation goals were recorded. At the beginning of the rehabilitation, a maximum of three goals and a minimum of one goal were agreed upon with the patients. The rehabilitation goals mentioned were assigned to the corresponding ICF categories, and the frequency of the ICF categories were analysed.
**Results**: Between November 2018 and April 2021, 103 burn victims (99 males, 4 females) with a mean age of 44.5 years (SD = 13.5 years) were enrolled in the study with 19.6% total body surface area (SD = 19.2% TBSA) and after an average of 36.5 days of intensive care. A total of 285 rehabilitation goals were recorded.
Rehabilitation goals from 23 different categories were stated. Skin-related rehabilitation goals, such as reducing scar tension, were stated most frequently (21%). The second most frequently stated goal was the improvement the mobility of one or multiple joints, such as improving hand function (20%). The third most frequently mentioned rehabilitation goal was improvement in walking ability (12%). The fourth most frequently mentioned rehabilitation goal was improvement of the strength of muscle function (11%). The fifth most frequently mentioned rehabilitation goal was the reduction in pain (8%).
**Conclusions**: The analysis of the rehabilitation goals according to ICF components shows a concentration on a few frequently mentioned ICF components. The five most frequently mentioned categories account for three quarters of the stated rehabilitation goals. A core dataset of ICF categories (disease-specific selection of frequent ICF categories) will be developed from these data. The rehabilitation goals mentioned demonstrate the specific problems of burn victims, which are expressed in the rehabilitation goals.

*O.061*

**Severe Burns: A Nationwide Study on Management and Outcomes**

**Anouk Pijpe ^1,2,3^, Tara Hartsuiker ^1,2,3^, Margriet Van Baar ^4,5^, Esther Middelkoop ^1,2,3^ and A Dutch Burn Repository Group ^1,4,6^**
  ^1^  Burn Center, Red Cross Hospital, and Association of Dutch Burn Centres
  ^2^  Amsterdam UMC location Vrije Universiteit Amsterdam, Plastic, Reconstructive and Hand Surgery
  ^3^  Amsterdam Movement Sciences, Tissue Function and Regeneration
  ^4^  Association of Dutch Burn Centres, Maasstad Hospital
  ^5^  Erasmus MC, University Medical Centre Rotterdam, Department of Public Health
  ^6^  Association of Dutch Burn Centres, Martini Hospital

**Objectives**: In current research there is limited knowledge on the general healthcare utilization and outcomes of burn patients with wounds exceeding 20% of their total body surface area (TBSA). The purpose of this study was to assess treatment characteristics and outcomes of severely burned patients in the acute phase, to analyze the prevalence, indications, and techniques of consecutive reconstructive surgery, and to evaluate outcomes in terms of scar quality.
**Methods**: A nationwide cohort study was conducted on all severely burned (>20% TBSA) patients admitted to one of the three Dutch burn centres between 2009 and 2019. Details on patient characteristics and procedures performed were collected over a 10-year follow-up period from the Dutch Burn Repository, a uniform national registration for Dutch specialized burn care. Scar quality was evaluated using data from a scar registration database from a single centre concerning patients admitted between 2009 and 2018.
**Results**: A total of 527 patients were admitted between 2009 and 2019 with a mean% TBSA burned of 43.7 ± 21.8. Data from 388 patients, who survived the initial care period, were analysed. of non-survivors, 3.2% became deceased during treatment, and other non-survivors either received comfort care or treatment was discontinued due to severe complications in an early treatment phase. Surgical treatment of acute burns took place in 90% of the patients (*n* = 350), and patients were most often (76.3% of procedures) treated with meshed split-thickness skin grafts. Consecutive reconstructive surgery was required in 24.8% of burn patients. The mean number of reconstructive procedures performed per patient was 4.4 ± 4.3. Arms and head/neck area were the locations most frequently operated on and the most frequent indication for surgery was scar contracture. Scar quality in patients with severe burns (>20% TBSA) was worse when compared with patients with less extensive burns <20% TBSA). General scar improvement occurred after the 3-month evaluation.
**Conclusions**: This study provides an overview of treatment and outcome characteristics in severely burned patients in the acute phase of burn care and evaluated the reconstructive needs up to 10 years after the initial burn incident. These results can be used as a baseline for studies that aim to study novel burn care modalities and their outcomes.
* The ‘Dutch Burn Repository group’ is composed of colleagues of the Burn Centre, Red Cross Hospital Beverwijk; Burn Centre, Maasstad Hospital Rotterdam; Burn Centre, Martini Hospital Groningen; Association of Dutch Burn Centres.

*O.062*

**Course of Perceived Fatigue after Pediatric Burns—A Prospective Cohort Study**

**Moniek Akkerman ^1,2^, Jessica J. J. Cramer-Kruit ^3^, Leonora J. Mouton ^2^, Bea Spek ^4^, Anuschka S. Niemeijer ^1,3^, Sonja M. H. J. Scholten-Jaegers ^5^, Lucas H. V. van der Woude ^2,6^ and Marianne K. Nieuwenhuis ^1,6,7^**
  ^1^  Association of Dutch Burn Centres, Burn Centre Groningen, Martini Hospital
  ^2^  Department of Human Movement Sciences, University of Groningen, University Medical Center Groningen
  ^3^  Scientific Institute, Martini Hospital
  ^4^  Department of Clinical Epidemiology, Biostatistics and Bioinformatics, University of Amsterdam, Amsterdam University Medical Center (Location Academic Medical Center)
  ^5^  Burn Centre Groningen, Martini Hospital
  ^6^  Center for Rehabilitation, University of Groningen, University Medical Center Groningen
  ^7^  Research Group Healthy Ageing, Allied Health Care and Nursing, Hanze University of Applied Sciences

**Objectives**: First, to describe the course of perceived fatigue in children and adolescents with burns during the initial six months after hospital discharge, and secondly, identify potential risk factors of burn-related fatigue, and examine the association between perceived fatigue and exercise capacity.
**Methods**: Eligible for this longitudinal prospective cohort study were children aged 6–18 years, admitted to one of the three Dutch burn centers with burns covering ≥5% of total body surface area (TBSA), or a length of stay of ≥2 weeks, or both. Perceived fatigue was assessed using both child and parent proxy reports of the Dutch version of the Pediatric Quality of Life Inventory Multidimensional Fatigue Scale (PedsQL MFS) at discharge, and at six weeks, three months, and six months after discharge. PedsQL MFS scores ≥1 SD below the age-group specific non-burned reference mean were considered to signify fatigue. Exercise capacity was assessed with the Steep Ramp Test.
**Results**: In total, 22 children and adolescents (13 boys/9 girls, age 6–18 years, and burns covering 2–34% of total body surface area) were included. The prevalence of perceived fatigue decreased from 65% at discharge to 28% six months after discharge. On group level, the severity of perceived fatigue decreased over time, reaching healthy reference values from six weeks after discharge. On individual level, the course of perceived fatigue over time varied widely. Age, sex, and burn severity could not predict the severity of perceived fatigue. Assessment of perceived fatigue at three months after discharge explained up to 69% of the variance in PedsQL MFS scores at six months after discharge. A weak association (r = 0.062–0.538) was found between perceived fatigue and exercise capacity.
**Conclusions**: Although the severity of perceived fatigue decreased over time in the majority of participants, 28% still experienced fatigue six months after discharge. Further research is required to explore the impact of burn-related fatigue on daily functioning and quality of life.

*O.063*

**Prevalence and Levels of Perceived Fatigue following Burns: Preliminary Results of a Systematic Review**

**Julia Prent ^1,2^, Maaike Wildekamp ^2^, Sonja Scholten-Jaegers ^1^, Han Houdijk ^2^, Marianne Nieuwenhuis ^1,2,3^ and Noor Mouton ^2^**
  ^1^  Association of Dutch Burn Centers, Burn Center Martini Hospital
  ^2^  University of Groningen, University Medical Center Groningen, Department for Human Movement Sciences
  ^3^  Hanze University of Applied Sciences Groningen, Research group Healthy Ageing, Allied Health Care and Nursing

**Objectives**: Perceived fatigue following burns has been described as a major problem, affecting adjustment to post-injury daily life. However, an overview of the extent of perceived fatigue following burns is lacking. Therefore, we aimed to describe (1) the prevalence of perceived fatigue following burns and (2) the difference in level of perceived fatigue between patients with burns and healthy reference values.
**Methods**: In April 2022, a literature search in PubMed was performed. Additionally, references from retrieved publications were screened. Studies were eligible for inclusion if the extent of perceived fatigue after burns was assessed by either reporting the prevalence of perceived fatigue, or by comparing levels of perceived fatigue between patients with burns and healthy references. Data regarding study design, participants characteristics, methods, and primary results were extracted.
**Results**: Thirteen publications were included. Most studies (seven) used the 36-Item Short for m Survey Vitality Subscale to measure perceived fatigue. In the remaining (five) studies, the Pediatric Quality of Life Inventory Multidimensional Fatigue Scale, the Brief Fatigue Inventory, or the Multidimensional Fatigue Inventory were used. Seven studies reported the prevalence of perceived fatigue after burns. There was a large variation in how these studies determined whether an individual experienced fatigue. In some studies, participants were asked whether they experienced fatigue (yes/no), whereas in other studies they had to score one or two standard deviations above the mean value of healthy references to be classified as experiencing fatigue. The prevalence of perceived fatigue was 70–75.2% in the acute phase. At twelve months after discharge, the prevalence ranged from 5.0–52.1%. Eight studies compared levels of perceived fatigue between patients with burns and healthy references. In none of these studies were significant differences found.
**Conclusions**: Perceived fatigue was, though highly variable between studies, fairly prevalent in patients with burns. In contrast, comparisons of levels of perceived fatigue between patients with burns and healthy references revealed no significant differences. These inconsistent results, in combination with the high variability in prevalence between studies, preclude definitive conclusions about the extent of perceived fatigue after burns. The results of this systematic review, highlight the importance of developing standardized methods to determine the prevalence and levels of perceived fatigue.

*O.064*

**Impacts of Patient Administered Analgesia in an Acute Burns Clinic**

**Isabella Stevens-Harris, Tom Challoner and Wareth Maamoun**
  University Hospital North Midlands

**Objectives**: A recurring problem noted during the assessment of new burn presentations referred to the out-patient clinic was that adequate pain relief had not been advised or taken prior to attendance. This resulted in both a poor patient experience and inadequate wound debridement due to excessive pain, compounded by the inability of analgesia administration in the out-patient department. A proposed information leaflet advising self-administration of simple analgesia prior to attendance would likely improve patient experience and facilitate comprehensive assessment of injury with adequate wound debridement in the out-patient setting.
**Methods**: A prospective audit was undertaken between November 2021 and January 2022. This included paediatric and adult patients referred to the University Hospital North Midlands burns dressing clinic. The data collected included patient demographics, Total Body Surface Area of the burn, burn location and whether analgesia had been both recommended and taken prior to the appointment. following the burn assessment and management, patients were asked to complete a pain score. In the paediatric cohort, the Wong–Baker Faces pain rating scale was used, or if they were too young to participate, the care giver completed this on their behalf.
**Results**: Within the adult population, 27% of patients took analgesia prior to their appointment with an average pain score of 2/10. Alternatively, the 73% without analgesia, had on average a higher pain score of 3/10. Within the paediatric cohort, results showed fewer than 15% of children had taken analgesia prior to assessment and an average pain score of 3.8/10.
**Conclusions**: It was noted that within both cohorts there was a significantly low proportion of patients having had any for m of analgesia prior to their first outpatient burns assessment. It was noted, particularly within the paediatric population, that patients were being influenced by care givers when completing the pain scores, or alternatively, parents were underrepresenting the pain of the child. A consequence of the inadequate analgesia was incomplete assessment and debridement of the burns, which may have resulted in a lower documented pain score due to under-debridement. As a result, an information booklet was created to be given to all patients seen in the emergency department with new burns, which contained advise on analgesia administration prior to the out-patient burns dressings clinic. A repeat loop of the audit cycle is being undertaken to re-evaluate the effectiveness of this change.

*O.065*

**Burn Patients and Delirium Risks**
**Aoife O’Brien ^1,2^, Jane Hopkins ^3^, Glenn Boardman ^4^, Guy Stanley ^1,2^, Patrick Daly ^1^, Lisa Martin ^2^ and Fiona Wood ^1,2^**
  ^1^  Burns Service of Western Australia, Fiona Stanley Hospital, SMHS
  ^2^  Burn Injury Research Unit, University of Western Australia, Crawley, WA, USA
  ^3^  Service 4, Fiona Stanley Hospital
  ^4^  Library & Information Service, Fiona Stanley Hospital, SMHS

**Objectives**: There were three aims of the study: to assess the compliance with the 4AT delirium risk screening tool, to assess delirium prevalence in a cohort of patients across four different surgical specialties, and to compare these four groups according to clinical outcome—length of stay, h in ICU, discharge destination, number of operations, mortality.
**Methods**: A snapshot retrospective audit was conducted to review the compliance with completion of the 4AT for m for patients who were later coded as delirious in the hospital system over 12-month period in burns, general surgery, and orthopaedic surgery. Subsequently, a retrospective service evaluation of delirious and non-delirious patients under the burns, acute general surgery, orthopaedic surgery, and plastic surgery teams was completed. This audit compared delirious and non-delirious patients’ length of stay (LOS), intensive care unit (ICU) h, numbers of operations, and age with univariate analysis and subsequent multivariant analysis.
**Results**: The screening tool was completed in 38% of cases later diagnosed with delirium. Burn patients with delirium are younger (mean 58 years), have fewer surgeries and a longer LOS (27 d) than patients in the general surgery, orthopaedics, and plastic reconstructive surgery specialties. across all specialties, delirium was more prevalent in females than males.
**Conclusions**: Burn patients more likely to have delirium at a younger age and after fewer surgical interventions compared with patients from other surgical specialties. Burn patients may have a lower age threshold for delirium than other specialties. Screening for delirium risk is particularly important for all burn patients, regardless of age, admitted to hospital for their acute injury, particularly if ICU admission is required.

*O.066*

**NMDA Receptor Encephalitis and Others Neurological Disorders in Burn Victims**

**Thorben Dieck, Nicco Krezdorn and Peter M. Vogt**
  Hannover Medical School

**Objectives**: Severe burn injuries can exacerbate pre-existing neurological diseases like epilepsy or can mask the occurrence of neurological diseases. We present the diagnostic and management of a 23-year-old patient with severe scald injuries based on a supposed psychiatric disorder with masked NMDA-receptor encephalitis in a 23-year-old patient.
**Methods**: Case report of NMDA-receptor encephalitis in a scald injured patient an overview of the management of neurological disorders in burn victims.
**Results and Conclusions**: Neurological disorders can be the cause of severe burn or scald injuries and can exacerbate during the treatment. Therefore, medical history, monitoring of neurological disorders and the awareness to relevant comorbidities are essential in the treatment of burn victims.

*O.067*

**Haemoglobin Transfusion Threshold in Adult Burns Intensive Care: “How Low Do We Go?”**

**Christopher Van Wyk, Russel Emamdee, Emed Chohan, Wajihah Saghir and Salih Salim**
  Saint Andrews Burns Unit

**Introduction**: Critically unwell patients were transfused to maintain Haemoglobin (Hb) of 10 g/L or above until Hebert et al. in 1999 showed lower mortality rates in young critically ill patients with an APCHE score of ≤20 who were set a transfusion trigger of 70 g/L. Current practise in burns management employs the same transfusion trigger. Patients with severe burn injury are more likely to spend longer periods in a critical care environment and undergo multiple surgeries as part of their healing and recovery process than the average patient in general critical care unit.
**Objectives**: The objective of the study was to analyse the Hb levels in adult patients admitted to our burns intensive treatment unit over the period of their stay.
**Methods**: after getting approval by the hospital’s audit department, a retrospective analysis of adult patients admitted to BITU during the years 2017 to 2019 who were considered survivable, and hence were managed in the unit for more than 2 weeks, was performed. Data were collected on pertinent demographics and all Hb levels done by the pathology laboratory during their stay. The final outcomes (survived/died) were also noted.
**Results**: Overall, 56 patients fulfilled the study criteria and were included in the study. In total, 48 patients (86%) were admitted following thermal injury and 8 (14%) were admitted following medical skin loss. The age range of patients was between 20 and 87 years. The injured skin area ranged between 5% and 92% TBSA. The length of stay was 15 to 120 days. On average, Hb levels in these patients were tested daily. A total of 2534 Hb samples were collected and analysed, of which 428 samples (17%) fell below the transfusion threshold of 70 g/L. In total, 651 samples (25.7%) showed a Hb level in the range of 70–75 g/L. Unfortunately, 9 of the study patients passed away after the initial two weeks.
**Conclusions**: The study showed a large proportion of Hb samples falling below the transfusion threshold. A quarter of the samples analysed had the Hb just above the transfusion trigger. The impact of waiting for Hb levels to fall below the conventional transfusion trigger in patients admitted with significant burns injury needs further evaluation.

**Reference**
  1. Hébert, P.C.; Wells, G.; Blajchman, M.A.; Marshall, J.; Martin, C.; Pagliarello, G.; Tweeddale, M.; Schweitzer, I.; Yetisir, E.; Transfusion Requirements in Critical Care Investigators for the Canadian Critical Care Trials Group. A multicenter, randomized, controlled clinical trial of transfusion requirements in critical care. Transfusion Requirements in Critical Care Investigators, Canadian Critical Care Trials Group. *N. Engl. J. Med.* **1999**, *340*, 409–417.

*O.068*

**Cutaneous Steam Burns and Steam Inhalation Injuries. A Case Report and a Literature Review**

**Sebastian Holm ^1^, Olof Engström ^1^, Marielle Melander ^2^, Monika C. S. Horvath ^2,3^, Filip Fredén ^4,5^, Miklós Lipcsey ^4,5,6^ and Fredrik Huss ^1,7^**
  ^1^  Burn Centre, Department of Plastic Additionally, Maxillofacial Surgery, Uppsala University Hospital
  ^2^  Division for for ensic Medicine in Uppsala, National Board of for ensic Medicine
  ^3^  Department of Surgical Sciences, Uppsala University
  ^4^  Department of Anesthesia and Intensive Care, Uppsala University Hospital
  ^5^  Department of of Surgical Sciences, Anesthesia and Intensive Care, Uppsala University
  ^6^  Hedenstierna Laboratory
  ^7^  Department of Surgical Sciences, Plastic Surgery, Uppsala University

**Objectives**: Scalds are one type of burn that are often mentioned alone and occur mostly in the paediatric population. Inhaled steam is mostly cooled off in the airways, which is why thermal damage is rarely seen. A sudden exposure to hot steam/inhalation can cause a thermal inhalation injury. We present a clinical case of a 26-year-old man with extensive cutaneous steam burns and a steam inhalation injury due to an explosion-like release of steam from the central heating in his house. The patient passed away after 11 days of treatment. Autopsy revealed extensive mucosal damage to the respiratory tract down to the alveoli.
**Methods**: A scoping review was performed, with the aim to summarize all published papers in English, about steam-related injuries.
**Results**: The search was conducted using the PubMed^®^ and Cochrane libraries on 19 May 2021, without a set time period. out of a total of 1186 identified records, 31 were chosen for review. The papers were screened for relevance and eligibility. The inclusion criteria used were all papers describing a case, or cases, involving a steam inhalation injury or burn. A total of 31 articles were included and analysed based on year of publication, type of paper, number of patients involved, age, gender, type of injury/event, treatment, and outcome.
**Conclusions**: Burns related to the contact with steam are generally rare and can be both minor and severe. The more severe cases related to steam exposure are mostly workplace accidents and the minor injuries reported in the literature are often related to steam inhalation therapy, especially in the paediatric population. Our literature review found eight case reports, thirteen case series, eight reviews, and two letters to the Editor. This review examines the challenges that can be found dealing with patients suffering from cutaneous steam burns and/or steam inhalation injuries. A steam injury to the airways or the skin can be directly life-threatening and should be treated with caution. Many studies highlight the danger of steam to the respiratory tract and its vulnerability to this type of injury that can lead to acute respiratory insufficiency and sometimes death. This is also demonstrated in the case report described above.

*O.069*

**Association between T-REGULARY Cells and Outcome in Patients with Severe Burn Injuries**

**Crescenzo Sala, Anna Lanza, Francesco Coletta, Maria Notaro, Mariella Loreto, Antonio Tomasello and Romolo Villani**
  Aorn Cardarelli

**Objectives**: A major burn leads to an inflammatory response and catabolism that, when compounded by burn wound nutrient losses, can lead to severe nutrition losses and deficiencies. These losses can impair immune function and wound healing and place burn patients at high risk for organ injury and mortality. Our aim is to demonstrate the correlation between T-regulatory cells and mortality in patients with severe burn injuries in order to find a new therapeutic approach.
**Methods**: This is a retrospective observational study; overall, 131 patients with severe burn injuries have been admitted in our Intensive Care Unit from June 2019 to March 2021. Patients were aged between 11 and 97 years (mean age 55 years). TBSA was between 8% and90% (mean 30%), 76 males and 49 females. for each patient at acceptance blood samples were taken including blood count, electrophoresis, electrolytes, liver and kidney function indices, PCR, Pct and coagulation. The day after admission, a sample was carried out for the study of lymphocyte subpopulations on a peripheral smear. Evaluation of the patients’ lymphocyte subsets was obtained by flow cytometry. Blood samples were performed daily, while PCR and PcT were performed at the entrance and after 7 days.
**Results**: Overall, 41 patients died (32% mortality among patients with TBSA > 40%). 45 patients (36%) required IOT, of these 32 (25% of the sample) required vasopressors. TBSA, ABSI > 8, IOT and the use of vasopressors increases mortality and morbidity. Our study shows that severe burns induce a marked decrease in HLA-DR expression, in the ratio of CD4 to CD8 and in the number of activated T lymphocytes, whereas B lymphocytes are generally increased. All this is associated with a worse outcome. According to what was observed in our cohort, an alteration of the expression of HLA-DR, of the activated T lymphocytes and of the CD4/CD8 ratio was observed in the first days after the event, especially in deceased patients and with higher TBSA and ABSI. The reductive alterations of these parameters were particularly related to the extent of the burn (TBSA), and also showed a significant correlation with the length of stay (LoS) and mortality. It was not possible to establish a correlation between alteration of the lymphocyte structure and the risk of developing complications of an infectious nature. In order to clearly delineate a relationship between the onset of sepsis and the different treatment strategies, a multivariate survey of large cohorts of patients is necessary.

*O.070*

**Mental Health and Burn Patients**

**Raimo Palmu, Timo Partonen, Kirsi Suominen, Jyrki Vuola and Erkki Isometsä**
  Helsinki University Hospital

**Objectives**: We have assessed mental disorders among acute burn patients pre-burn and post-burn. Here, we review all 10 publications of ours.
**Methods**: Medical records of 811 patients treated in Helsinki Burn Center during 1989–1997 were assessed for suicidality. Next, all consecutive acute burn patients (*n* = 107) admitted to the Helsinki Burn Center during 2006–2007 were assessed with the structured clinical interview for mental disorders (SCID), prospectively followed and examined again at 6 months post burn. Information on their suicidality, quality of life, functioning and return to work as well as influence of alcohol, smoking and impulsiveness was collected with questionnaires and from medical records.
**Results**: Suicidal patients (5.7% of all) had greater TBSA (24% vs. 6%), flame injury and psychiatric morbidity, were unemployed or on disability pension more often than the remaining burn patients. In total, 60% had at least one lifetime mental disorder before burn injury, their frequencies of lifetime substance-related disorders (47%), psychotic disorders (10%) and personality disorders (23%) were clearly higher than in the general population. Although the frequencies of depressive disorders and PTSD were relatively high after the burn, the total picture of post-burn mental disorders is much more variable. We found a robust relationship between burn severity and certain mental disorders post burn. Psychiatric consultation (20%) and actual psychiatric care (21%) were rare during the 6 months after burn. Previous psychiatric history strongly influenced care decisions. The quality of life was unexpectedly good among acute burn patients and predicted by mental disorders. Difficulties in social and occupational functioning remained at 6 months after burn, being predicted by both TBSA and post-burn mental disorders. Two-thirds of those employed at baseline returned to work during the 6 months post burn. The capacity to work was predicted by both smaller TBSA and lack of diagnosable mental disorders after burn. In total, 52% were under the influence of alcohol, and 19% had been both drinking and smoking at the moment of the burn. They had lifetime psychiatric disorders (73% vs. 45%) significantly more often. Impulsiveness did not correlate with burn severity or any burn-related variable but was associated with major depression, alcohol dependence and personality (antisocial) disorders.
**Conclusions**: Having at least one mental disorder is important to the outcome pre burn, at burn and post burn even if compared with severity of the burn. Attention to alcohol-use disorders and risk behaviors as well as their screening in clinical contact is needed for the prevention of burns.

*O.071*

**Post-Traumatic Stress Symptoms and Interpersonal Processes in Burn Survivors and their Partner**

**Elise Boersma-van Dam ^1,2^, Rens van de Schoot ^2^, Iris Engelhard ^2^ and Nancy Van Loey ^2,3^**
  ^1^  Association of Dutch Burn Centres
  ^2^  Utrecht University
  ^3^  Association of Dutch Burn Centres

**Objectives**: Burn survivors and their partner can develop post-traumatic stress disorder (PTSD) symptoms, and a burn event may also impact the way couples interact with each other. Survivors and partners may try to protect each other from further emotional distress by avoiding talking about the burn event, but they may also encourage each other to talk about the burn event. The aim of the study was to investigate bidirectional relations between survivor’s and partner’s PTSD symptoms and avoidance- and approach-oriented interpersonal processes.
**Methods**: In a longitudinal multi-center study 118 couples of burn survivors and their partner participated. Measures of PTSD symptoms and partner-oriented avoidance and approach were assessed in the acute phase following the burns, and follow-ups took place up to 18 months post-injury. Intra- and interpersonal effects were examined in a random intercept cross-lagged panel model.
**Results**: Results showed that the survivor’s approach positively predicted subsequent levels of the survivor’s PTSD symptoms, and partner’s PTSD symptoms positively predicted subsequent levels of their partner’s approach. Additionally, partner’s avoidance and partner’s PTSD symptoms reinforced each other over time. Between the two couple members, partner’s approach predicted subsequent lower levels of survivor’s PTSD symptoms. Furthermore, exploratory regression analyses showed that burn severity moderated the effect of survivor’s avoidance on survivor’s PTSD symptoms, showing that avoidance was related to higher levels of PTSD symptoms within more severely burned survivors, but not in less severely burned survivors.
**Conclusions**: In conclusion, the partner’s approach was related to lower levels of the survivor’s PTSD symptoms, whereas the survivor’s approach was related to higher levels of the survivor’s PTSD symptoms. PTSD symptoms and partner-oriented avoidance reinforce each other over time in partners and possibly also in more severely burned survivors. These findings emphasize the importance to screen for and monitor PTSD symptoms in burn survivors and their partner and encourage couples’ mutual self-disclosure.

*O.072*

**Body Image Dissatisfaction and Sexual Problems after Burns**

**Nancy Van Loey ^1^, Anne-Sofie Goemanne ^2^, Els Vandermeulen ^3^, Inge Bastiaenssen ^4^, Anita Boekelaar ^5^, Jetty Meijer ^6^ and Helma Hofland ^7^**
  ^1^  ADBC, Maasstad Hospital
  ^2^  University Hospital
  ^3^  Queen Astrid Military Hospital
  ^4^  ZNA
  ^5^  Red Cross Hospital
  ^6^  Martini Hospital
  ^7^  Maasstad Hospital

**Objective**: Burn injuries can impact diverse domains of functioning, including intimacy and sexuality. Burns can cause extensive scarring which may affect appearance and sensitivity of the skin. Adjusting to an altered appearance may impact body image satisfaction, which has been shown to be an important factor in sexual life. The aim of this study was to investigate the possible impact of burn severity and body image dissatisfaction on sexual problems in the aftermath of a burn injury.
**Methods**: Adult burn survivors admitted to a burn center in The Netherlands or Belgium were invited to complete the Satisfaction with Appearance scale (SWAP) 3 months postburn and the Burns sexuality scale (BuSS) 6 months postburn. Linear regression analysis was used to investigate the impact of TBSA burned, body image, age and gender, on sexual problems.
**Results**: A total of 117 adult burn survivors completed the measures. Body image dissatisfaction and age predicted more sexual problems. Gender and TBSA burned did not predict sexual problems, but showed to be significantly related to body image dissatisfaction, indicating an indirect relationship through body image with sexual problems.
**Conclusions**: This study provides evidence for a relationship between body image problems and sexual problems and highlights the need for health care professionals to screen for and address sexuality and body image issues. Guiding partners to be involved in care before discharge from the hospital may pave the way to an open communication between partners to discuss their concerns and doubts related to body image issues.

*O.073*

**A Preliminary Study to Build a Diversity Sexual Guideline in a Burn Unit**

**Sara Guila Fidel-Kinori, Eudald Castells-Panisello, María Sonsoles Cepeda-Diez, Marisa Perez del Prado, Angel Arevalo-Bernabe, Maria Lluïsa Torrent-Bertran, María José Sanchez-Garcia, Laura Grossi-Garriga, Pol Miguel-Puigbarraca, Mónica Soler-Rovira, Antonio Bulla, Jordi Serracanta-Domenech and Joan Pere Barret-Nerin**
  Hospital Universitario Vall d’Hebron

**Objectives**:
  − Carry out a study on the criteria and opinions of the burns care team, on Sexual Diversity.
  − Use the results to create a guide of good practices for admission and health care for people with sexual diversity.
  − Methods.
  − Design a digital survey with the main items for inclusive clinical practices, based on International LGTBQ+ guidelines.
  − Six types of questions, based on the International Guidelines on Assistance to LGTBQ+ Collective, were designed to be assessed with a likert scale, from 1 = totally disagree to 5 = maximum agreement.
  − Sent to all health and non-health personnel who work in the Burn Unit of the Vall d’hebron Hospital. (*n* = 71).

**Results**: A total of 34 responses were received out of 71 (48%). Regarding gender, 18 were women (52.9%); 16 are doctors (47.1%), 12 nurses (35.3%), 3 are clinical assistants (8.8%) and 3 have other occupations (8.8%). The average job seniority is 12 years (M = 12.26, SD = 16.855), with a range between 1–25 years. 23.7% work between 37.5 and 40 h/week, 8.8% work between 20 and 30 h/week, between 10 and 20 h/week (5.9%) and 14.3% have other h. Regarding the results of the survey, in Question 1 (Sexual orientation and gender identity can affect the health and well-being of patients.) an average of 4.65 (SD = 0.812) stands out. In Question 2 (With an understanding of gender and sexual identity, we can provide more appropriate care) the mean is 3.44 (SD = 1.211), showing a more central response tendency, and in Question 3 (Health care that responds to the unique and specific needs of LGTBQ+ patients, such as having a single room, can improve the patient-unit relationship and adherence to treatment.) the mean is 4.24 (SD = 1.103), whereas in Question 4 (Waiting rooms and other common spaces must reflect and be inclusive for LGTBQ+ patients and family members …) the mean is 4.26 (SD = 0.898). In Question 5 (It would be necessary to create specific work groups, health personnel and people from the LGTBQ+ community, to design an environment of respectful and inclusive treatment) the mean is 4.41 (SD = 0.957), and in Question 6 (I am aware of my misconceptions, prejudices and stereotypes and other communication barriers …), the mean is 3.91 (SD = 1.138).
**Conclusions**: The responses received, with a tendency towards the affirmation of the items, in terms of integrating and giving sensitive and specific treatment to people who self-identify within the LGTBQ+ collective, led to recommendations for building a consensual guideline.

*O.074*

**Self-Inflicted Versus Accidental Burn Injuries: A Systematic Review of Characteristics and Outcomes**

**Ezekwe Amirize ^1^, Oluwasemilore Adebayo ^2^, Kwaku Duah-Asante ^3^, Hadyn Kankam ^1^, Abdulrazak Abdulsalam ^1^ and Naiem Moiemen ^1,2^**
  ^1^  Scar Free Foundation Centre for Burns Research, University Hospitals Birmingham NHS Foundation Trust, Birmingham, UK
  ^2^  University of Birmingham
  ^3^  Imperial College School of Medicine

**Objectives**: Due to their peculiar characteristics, self-inflicted burn injuries constitute a significant burden to affected patients, their families and the healthcare system compared with accidental burns. They occur worldwide with varying incidence amongst cultures, regions, social and individual health backgrounds. However, irrespective of location and personal circumstances, these injuries are preventable. This review aims to highlight the characteristics and outcome of this patient population in comparison to accidental burn injuries from available literature. The available evidence will help identify possible instigating factors and mechanisms to mitigate these injuries.
**Methods**: A comprehensive literature search was performed using Medline and Embase databases. Variations of the terms “self-inflicted”, “suicide” and “burns” were combined in a search strategy, with MeSH and Emtree terms. Titles were screened in parallel by independent investigators. Strict predefined eligibility criteria were employed to identify studies comparing the characteristics and/or outcomes of self-inflicted burns with accidental burns patients. Qualitative analyses and risk of bias assessments were undertaken for each included study.
**Results**: The literature search returned 3241 hits. following removal of duplicates, title/abstract and full text screening was conducted with 26 studies identified for inclusion in this review. All studies compared the characteristics of patients with self-inflicted burns to those with accidental burns; in total, 17 studies also assessed the outcomes. Studies were retrospective and covered most continents, with the exception of Africa. Relative to accidental burns, self-inflicted burn cohorts were predominantly female, younger and unemployed with pre-existing psychiatric disorders. Marital and/or family problems were frequently cited triggers for the incident and recreational drug and alcohol abuse was also more common in this group. With regard to intentional burns, flames were the most common source of injury, and they were more likely to be severe in nature (as quantified by the percentage total body surface area affected). In hospital length of stay and mortality were also significantly greater, relative to the accidental burns population.
**Conclusions**: Intentional self-inflicted burns are common in certain cultures as a means of protest and among people with impulsive behavior. The development of best practices for prevention, treatment, rehabilitation and social support is necessary. Targeted and community-based prevention strategies would be worthwhile. Increased awareness of these injuries and the complications of burns should be in the spotlight.

*O.075*

**Good Quality of Life 30 Years after a Mass Burn Accident: A Case Report of 85 Years Old Man**

**Janneke Damen-Thissen ^1,2^, Kiran Baran ^1^ and Paul van Zuijlen ^1,3,4,5^**
  ^1^  Burn Center Red Cross Hospital
  ^2^  Department of Geriatrics, Red Cross Hospital
  ^3^  Department of Plastic and Reconstructive Surgery, Red Cross Hospital
  ^4^  Paediatric Surgical Centre, Emma Children’s Hospital, Amsterdam UMC, location AMC
  ^5^  Amsterdam Movement Sciences (AMS) Institute, Amsterdam UMC

**Objectives**: In 1992, a man aged 55 years old was admitted at the Burn Center Beverwijk after being a victim in a mass burn accident. Besides being an employer at a chemical factory, he was also a volunteer firefighter for this company. He was one of the men trying to stop a massive burn accident. Due to an explosion at this chemical factory, our patient sustained flame injury with a TBSA of 68%. Together with this burn victim, we evaluated his quality of life 30 years after this massive burn injury.
**Methods**: We requested informed consent from this patient and his wife. Together with the plastic surgeon, who has treated this 55-year-old burn victim, we performed a status investigation. We interviewed the man and his wife. We discussed topics such as: rehabilitation trajectory, frailty (G8) and quality of life (EQ-5D-5L; Burn Specific Health Scale—Brief Questionnaire) at the age of 85.
**Results**: We obtained informed consent from our patient and his wife. The plastic surgeon reviewed his work from 30 years ago: the patient was admitted for 99 days at the Burn Center Beverwijk, underwent 35 operations and underwent the rehabilitation process for 2.5 years. The patient had no relevant medical history before the accident. At the age of 83, a Transcatheter Aortic Valve Implantation was performed because of aortic stenosis. Before the surgery the geriatric patient did not showed frailty, except based on his age and a daily medication amount of 5 (G8 = 15/17). This 85-year-old man and his wife are living independently, and he spends every day working in his allotment garden. The quality-of-life questionnaires showed high scores: EQ-5D-5L shows no disabilities and limitations in daily living (level 1/5). Additionally, the Burns Specific Health Scale—Brief (domains describing function with respect to heat sensitivity, affect, hand function, treatment regimens, work, sexuality, interpersonal relationships, simple abilities, and body image) was taken: in addition to problems picking up coins (2/4 = moderate), there were no limitations at all (4/4 = none).
**Conclusions**: This case report shows us that despite his extensive burn injury, this man has a good quality of life, even at the age of 85. He has come a long way after 99 days of admission at the Burn Center Beverwijk and a rehabilitation trajectory of 2.5 years. He is living independently without limitations in daily living. Every day, he enjoys working in his allotment garden!

*O.076*

**The Vital Role of the Social Worker in the Burn Centre**

**Klaudia Kokkola**
  HUS Helsinki University Hospital

**Objectives**: The aim of this presentation is to give an overview of the role of the social worker with burn patients in specialized health care and address the issue of social determinants of health and co-occurrence of social problems and health issues. Social work as a profession is integrated into the health care system in many countries. Medical social work is specialized in understanding the relationship between health issues and social factors. Burn patients often have severe social problems and as a patient group represent an example of how social problems and health issues are closely connected.
**Methods**: The presentation focuses on addressing how social problems and health issues are related and demonstrating the work of social workers with burn patients in a Burn Centre. A literature review and personal experiences are used as the basis for this study.
**Results**: Social work plays a major role in the rehabilitation process of burn patients. It is essential that social work as a profession is integrated into the health care system because social problems and health issues often are associated.
**Conclusions**: As burn patients often have severe social problems, it is important to understand how non-medical factors also affect health issues and why the role of social work in health care is fundamental.

*O.077*

**Pain, Depression, Anxiety, Alexithymia and Poor Sleep in Patients with Post-Burn Hypertrophic Scars**

**Sara Gavinelli ^1^, Maurizio Stella ^1^, Gianluca Isoardo ^2^, Alessandro Cicolin ^2^ and Alessandra Giordano ^2^**
  ^1^  Centro Grandi Ustionati C.T.O
  ^2^  Centro Multidisciplinare per La Diagnosi e La Terapia Dei Disturbi Del Sonno

**Background**: Post-burn hypertrophic scars (PBHSs) are frequently complicated by intense pain and itch. Previously, we demonstrated that pain in PBHS is neuropathic in nature. Poor sleep quality, depression, anxiety, alexithymia, and cognitive impairment are frequently associated with pain and the mechanisms underlying pain chronicization and emergence of sleep, mood, emotion regulation and cognitive impairment may be common. This association can be detrimental for quality of life, more than pain in itself.
**Methods**: In this cross-sectional study, we investigated psychological features with the Beck Depression Inventory II (BDI-II), the State-Trait Anxiety Inventory (STAI-Y), the 20-item Toronto Alexithymia Scale (TAS 20), the 12-item General Health Questionnaire (GHQ-12), the Multidimensional Scale of Perceived Social Support (MSPSS) and the Body Consciousness Questionnaire (BCQ). Sleep quality was evaluated by the Pittsburgh Sleep Quality Index (PSQI). Pain was graded by Numeric Rating Scale (NRS) and its neuropathic origin screened by Douleur Neuropathique 4-question (DN4) score. Executive function was evaluated by frontal assessment during outpatient visit. Results of comparison with age and sex matched healthy controls and of correlations among psychological feature, executive function, sleep quality and pain will be presented.
**Results**: Patient recruitment for the study will end in August.

*O.078*

**Monitoring of Biomarkers during Wound Healing and the Impact of Exacerbated Inflammation**

**Anna-Lisa Pignet ^1,2^, Elisabeth Hofmann ^1^, Anna Schwarz ^1^, Julia Fink ^1^, Marlies Schellnegger ^1,2^, Johanna Einsiedler ^1^, Judith Holzer-Geissler ^2^, David Hahn ^1^ Anita Eberl ^3^, Eva-Maria Prugger ^3^, Thomas Birngruber ^3^, Lars-Peter Kamolz ^1,2^ and Petra Kotzbeck ^1,2^**
  ^1^  Joanneum Research for schungsgesellschaft mbH, Coremed—Cooperative Centre for Regenerative Medicine
  ^2^  Medical University Graz, Division of Plastic Aesthetic and Reconstructive Surgery, Research Unit for Tissue Regeneration, Repair and Reconstruction
  ^3^  Joanneum Research for schungsgesellschaft mbH, Health

**Objectives**: Skin injuries initiate myriad biological processes, including inflammatory responses and the release of cytokines and other signaling molecules. While inflammatory responses are an essential component of wound healing, excessive inflammation is a common complication in burn injuries and may lead to hypertrophic scar for mation and promotes chronic wound development in acute wounds. The exact sequences of events initiated by exacerbated inflammation still needs extensive investigations. Therefore, reliable models are needed to elucidate the mechanisms during physiologic and disrupted healing processes. Here, we present detailed data on events during the early phase of healing in physiologic, as well as in highly inflamed acute wounds and burns.
**Methods**: We established porcine models for superficial, full-thickness and burn wounds to analyze healing and the impact of prolonged inflammation. Resiquimod, an immunomodulator that causes inflammation through TLR7/8 signaling, was topically applied to extend the inflammatory phase for up to 6 days after wounding. Wounds without resiquimod served as respective controls. Wound healing progression was observed for ten days using hyperspectral imaging, thermography and wound scores. On the final day of the experiment, biopsies were harvested to characterize histological and immunohistological changes. Furthermore, the gene expression and production of various cytokines (e.g., IL-6, CXL8, IL-10, TNF, TGFb1), adipokines (e.g., adiponectin, leptin, dermatopontin, RBP-4) and growth factors (e.g., ACTA2, VEGFA, HIF1a) in wounds of different healing states were analyzed. To characterize the dynamics of the secreted cytokines, growth factors and eicosanoids (e.g., prostaglandins, thromboxanes) dermal interstitial fluid was collected via open-flow-microperfusion.
**Results**: In physiologic wound healing, the release of signaling molecules, such as IL6, CXCL8, PGE2 and TNF peaked on the second day and declined on the sixth day after wounding. Application of the immunomodulator resiquimod to split-skin and full-thickness wounds induced severe inflammation, resulting in significantly poorer healing compared with the corresponding control wounds. Pro-inflammatory biomarkers were increased at the level of gene expression, protein production, as well as secretion. Interestingly, we found that increased inflammation in resiquimod-treated wounds resulted in the for mation of a significant adipocyte layer in the dermis and epidermis. Gene expression analysis of adipokines showed that adiponectin, leptin, dermatopontin and RBP-4 were altered, at least in resiquimod-treated wounds.
**Conclusions**: We believe the precise knowledge of the processes during physiologic and delayed wound healing is crucial for the development of new therapeutic strategies to support healing after burns and to prevent hypertrophic scarring or wound chronicity.

*O.079*

**A Retrospective Study on Rapid Removal of Biofilm and Necrosis with a Hygroscopic Chemical Debriding Compound**

**Michel Hermans**
  Hermans Medical Consulting

**Objectives**: Debridement is an essential step in the treatment of all lesions. A new material (TDA) uses a hygroscopic mechanism to provide debridement in an innovative way: when in contact with biofilm and necrosis, rapid desiccation occurs, with subsequent dissolution of these compounds. TDA is primarily aimed at treating chronic lesions (i.e., venous and diabetic ulcers): in these indications, excisional surgical debridement typically is not performed, and TDA was developed as a fast-acting and effective alternative. In a retrospective study, clinical performance was conducted, with the objective of assessing possible side effects of the compound, as well as the speed and percentage of the development of granulation tissue, which is a crucial condition for healing by secondary intention.
**Methods**: Data of patients of >18 years, with lesions of the lower leg or foot older than 4 weeks and without a clear healing trend, were assessed. TDA was applied once, followed by treatment with Vaseline gauze. Time to, and percentage, of complete granulation were assessed.
**Results**: Diabetic (*n* = 20) and venous (*n* = 22) ulcers in 54 patients (39 males, (72%), average age: 72.0 years) participated in the study, as well as post-trauma (*n* = 9), vasculitic (*n* = 2), and an ischemic lesion. of the 20 diabetic ulcers, 12 (37%) were diagnosed as Wagner III-IV. The average lesion size was 57.4 cm2, and lesion age ranged from 1 to 12 months. Most patients suffered from serious comorbidities.
No study-compound-related side effects occurred. At study end, 50 out of 54 lesions (92.5%) showed complete granulation in, on average, 36.2 days. The four lesions that did not granulate completely were Wagner grade III–IV ulcers, with osteomyelitis and/or severe microcirculatory ischemia as underlying reasons.
**Conclusions**: The aetiology and pathology of ulcers is significantly different from those of burns. Therefore, extrapolation of our results into burn treatment is inappropriate. There is a category of burns, however, that could benefit from a treatment with TDA. These are the deep lesions that are typically too small to be excised, such as a full-thickness burn that results from contact with a hot motorcycle exhaust pipe or cannot be excised for other reasons. We hypothesize that the use of TDA would shorten time to healing significantly since a crucial first step in wound healing by secondary intention, (waiting for) the development of granulation tissue, could be skipped completely.

*O.080*

**Platelet-Rich Plasma in Burns: A Scoping Review**

**Gonçalo Ferreira, José Miguel Azevedo, Dmitry Shelepenko, Inês Catalão and Luís Cabral**
  Plastic and Reconstructive Surgery and Burns Unit Department, Coimbra Hospital and University Centre

**Objectives**: The propose of this study was to analyze the impact, identify knowledge gaps, clarify concepts, and examine how research has been conducted regarding the role and safety of PRP in burn patients.
**Methods**: A scoping review was performed. PubMed, Scopus and Embase were used for the literature search. Articles were subjected to a methodical evaluation following the Preferred Reporting Items for Systematic Reviews and Meta-Analyses (PRISMA) standards for Scoping Reviews and those of Joanna Briggs Institute and the Systematic Review Center for Laboratory animal Experimentation (SYRCLE) for quality assessment. Only those focusing on the evaluation of the effect of PRP in burn patients were included.
**Results**: In total, 48 articles were included. They involved skin burns (*n* = 31), ocular burns (*n* = 8), esophageal burns (*n* = 2) and general considerations (*n* = 7). Considerable study type variability, differences in methods for PRP effect analysis, and its production protocol disparity were encountered. Overall main findings included the PRP positive effect on wound healing, pain reduction, scar regression, in esophageal function the recovery and positive recovery after alkaline eye burn. Nevertheless, the results specifically associated with re-epithelization are not fully consensual. Regarding PRP safety, no relevant complications or side effects were reported.
**Conclusions**: PRP stands out for the vast potential and positive impact on burn healing. However, relevant PRP production heterogeneity and inconsistency on the results analysis methods, may limit its study and effect evaluation. Methodological standardization and further randomized clinical trials are essential to better understand PRP role on burn patients and to ensure its application safety.

*O.081*

**Randomized Controlled Trial of Second-Degree Burns Treatment: Silver Sulfadiazine versus Natural Silk**

**Eric Dantzer**
  Instruction Military Hospital Sainte Anne

**Introduction**: Silver sulfadiazine (SSD) is currently the standard treatment for superficial second-degree burns. These burns are common and should not necessarily require hospital treatment. However, caretaking may become an issue: the dressing technique is not always mastered by home health care nurses, and their practical realization may be more difficult considering the surface to be treated; the duration of care can be too long and painful. We have evaluated the interest of a dressing based on natural silk with this indication.
**Material and Methods**: In a preliminary study, we have compared SSD with silk dressing (S) in the treatment of second-degree burns. following diagnosis, the topical to be used was determined by drawing of lots. The SSD associated with fatty gauze was applied and then covered with dry gauzes and bandages. Silk was applied directly on the lesion and then covered with dry gauzes and bandages. Dressings were changed every 48 h. The SSD was changed with each dressing after cleansing the lesion with saline; the silk one was left in place until healing, and only the dry gauzes and bandages were changed. Pain, duration of care and healing time were assessed.
**Results**: Overall, 60 patients were included: 19 men and 11 women (7 to 83 years) were treated with SSD; 16 men and 14 women (17 to 84 years) were treated with silk. The average surface area treated was 5% in both groups (1% to 20% SSD and 1% to 23% S). Pain (EVA) was evaluated between 3 and 4/10 for the SSD group and between 0 and 2 for the S group. The dressing duration was 20–30 min for the SSD group and 10–15 min for the S group. The average healing time was 13 days for both groups. Two complications, such as infection, were observed in each group.
**Conclusions**: The flexibility of silk facilitates application on all surfaces and localisations. Silk and SSD treatment has the same healing time as that obtained with SSD. Silk has allowed shorter treatments, because it is technically simple, and less painful as the lesion was never exposed to air. This enabled a reduction in analgesics. A larger study should confirm that second-degree burns can be managed with silk dressing as outpatients, by home health care nurses, inexperienced in burns and even for larger surfaces and reduce the cost effective of the treatment and the need for painkillers.

*O.082*

**Developing a Burn-Specific Perioperative Checklist: Version Zero, a Starting Point**

**Laura Cappuyns ^1,2^, Andrew Holme ^1^, Susan McCrossan ^3^, Nicole Lee ^4^, Simon Booth ^5^, Anirban Mandal ^1^, Dilnath Gurusinghe ^1^, Ciaran O’Boyle ^6^, Mohammad Anwar ^3^, Baljit Dheansa ^5^, Nora Nugent ^5^, David Ralston ^7^ and Kayvan Shokrollahi ^1^**
  ^1^  St. Helens & Knowsley Teaching Hospitals NHS Trust
  ^2^  Manchester Metropolitan University
  ^3^  Mid Yorkshire Hospitals NHS Trust
  ^4^  Chelsea and Westminster Hospital NHS Foundation Trust
  ^5^  Queen Victoria Hospital NHS Foundation Trust
  ^6^  Nottingham University Hospitals NHS Trust
  ^7^  Sheffield Teaching Hospital NHS Foundation Trust

**Introduction**: The World Health Organization (WHO) surgical safety checklist is a widely applied tool in operating theatres aimed at identifying potential faults or omissions before they can culminate into harm to patients. Its use has led to decrease in peri-operative errors and adverse events and promoted an increase in teamwork and communication in surgery worldwide. However, the generic checklist does not take into account certain considerations unique to sub-groups of surgical patients, like burns. Burn patients often have complex peri-operative requirements including need for specialist equipment, large volumes of blood products and a greater requirement for pre-operative optimisation. Therefore, a modified burn-specific checklist may be of value.

**Objectives**
  (1) To adapt the generic WHO surgical safety checklist into a burn-specific surgical checklist.
  (2) To recruit and engage as many burns services as possible to develop the concept.

**Methodology**
  1. Initial dialogue between a small number of burns services to develop a proof-of-concept burn-specific checklist.
  2. Development of a ‘version zero’ burn-specific theatre checklist.
  3. Engagement with more burns services with a view to using a modified Delphi methodology to establish a working group to develop a ‘version one’ burn-specific theatre checklist.

**Results**: A ‘version zero’ burn-specific theatre checklist has been developed following input from the full spectrum of the burn MDT. New parameters relevant to burns have been added to the SIGN IN, TIME out and SIGN out parts of the WHO checklist. It was also felt that there would be considerable benefit in starting the checklist the day before surgery. Some key parameters include:
  (1) Day before surgery:
    a. Ordering of allograft/skin substitutes;
    b. Cross-matching of various blood components;
    c. Investigations and pre-optimising patients for theatre including an anaesthetic assessment;
    d. Pre-operative microbiology discussion;
    e. Operating list order relevant to patient infection status (MRSA/CPE/COVID);
    f. Medical photography;
    g. Discussion of preferred graft donor sites.
  (2) SIGN IN:
    a. Theatre pre-warming;
    b. Confirmation of graft donor sites.
  (3) TIME OUT:
    a. Shaving of graft donor sites;
    b. Patient position and planning for position changes;
    c. Preparation of local anaesthetic/adrenaline infiltration.
  (4) SIGN OUT:
    a. Post-operative positioning and splintage;
    b. Post-operative warming.
**Conclusions**: The burn-specific theatre checklist would start the day before surgery and would potentially aid the appropriate pre-operative optimisation of patients and streamline care. It has the potential to improve surgical safety, staff communication, efficiency of theatre utilisation and time management, including reducing the incidence of cross-infection.

*O.083*

**Artificial Dermis, an Alternative to Flaps**

**Eric Dantzer**
  Instruction Military Hospital Sainte Anne

**Introduction**: Flaps are the gold standard to cover exposed tendons, bones or neuro-vascular pedicles. In difficult situations such as deep and large wound defects, flaps are difficult to realize. Artificial dermis (AD) could be used as an alternative solution.
**Materials and Methods**: after excision of death and infected tissue, large and deep defects with exposed vessels, nerves, tendons or bones, have been covered by a tridimentional collagen elastin matrix and an epidermal graft. NWPT have been used for deep and infected wound defects. Follow up was clinical and functional assessment.
**Results**: Overall, 39 patients (23 to 72 YO) 25 males, 14 females, were treated. In total, 20 patients were treated for burns, and 19 for necrotizing fasciitis. In total, 12 patients were treated for injury to the hands, 25 for the lower legs and feet and 5 were grafted for other locations. The surfaces grafted were 74 cm^2^ to 1280 cm^2^. NWPT was used for 18 patients, and changed every 4 days before grafting AD. Mean time before grafting AD after NWPT was 3 weeks. The mean healing time of the AD was 2 weeks. All of the exposed zones were covered. By restoring the shearing planes, the collagen–elastin matrix thus avoids deep-rotted adhesion and improves the tegumentary suppleness and the final functional by the free tendinous play obtained under the composite grafts. With a final healing time, the number of surgical procedures reduced. The results were obtained with a simple surgical reproducible technic, without the disadvantages of difficulties, potential complications, and donor site scarring of the flaps. The graft incorporated more naturally than a flap, i.e., allowing the patient to wear normal shoes.
**Conclusions**: Even in difficult situations, AD could be considered as a surgical alternative to flaps, and could be used without much risk, as the preferred solution while keeping the second possibility of using flaps.

*O.084*

**Corticosteroid Embedded Dissolving Microneedles (CEDMN) Are a Painless and Effective Alternative to Intralesional Injection in the Management of Hypertrophic Scarring**

**Ignace De Decker, Henk Hoeksema, Jozef Verbelen, Petra De Coninck, Els Vanlerberghe, Anna Szabo, Sandra Van Vlierberghe, Stan Monstrey and Karel Claes**
  Ghent University Hospital Burn Center, Department of Plastic, Aesthetic and Reconstructive Surgery

**Objectives**: Severe burn injuries or comparable full-thickness skin defects are frequently followed by hypertrophic scarring. Pressure therapy, scar hydration and UV protection are the main principles of scar management. Thick and rigid hypertrophic scars sometimes require the intralesional injection of corticosteroids to reduce excessive scarring by reducing intradermal inflammation and thereby inhibition of collagen synthesis. The intralesional administration of corticosteroids can be painful, lacks equivalent surface dosage and potentially induces subcutaneous fat atrophy. Dissolving microneedle arrays are a more recent method for painless local or even systemic drug delivery and were originally developed for immunization purposes by incorporation of antigens in the microneedles. Hyaluronic-acid-based corticosteroid-embedded dissolving microneedles (CEDMN) might provide an alternative to injection.
**Methods**: In a pilot study, five patients with hypertrophic scars requiring corticosteroid injection were intra-individually treated monthly with CEDMN patches with 600 and 800 micrometer (µm) long microneedles for 4 and 2 months, respectively. The 600 µm and 800 µm patches, respectively contain 0.23 mg/cm² and 0.70 mg/cm² of a 5:2 mixture of betamethasone dipropionate and betamethasone sodium phosphate. Skin hydration and trans epidermal water loss were assessed by the Corneometer and Tewameter, respectively. The Patient and Observer scar assessment scale 2.0 (POSAS) was used for subjective assessment. The Visual Analogue Scale (VAS) was used for pain assessment prior to, during and 1 min after insertion.
**Results**: Mean overall POSAS patient scores of the control, 600 µm patch and 800 µm patch treated sites, respectively improved with 20%, 47.5% and 35% during the treatment period. Mean overall POSAS observer scores of the 600 µm patch and 800 µm patch treated sites, respectively improved with 35% and 45%, while the mean overall observer score of the control site worsened with 6%. Skin hydration and evaporation remained comparable during the entire treatment period (*p* > 0.05). No pain was reported by any of the patients during treatment. No complications or side effects were encountered.
**Conclusions**: Hyaluronic acid based dissolving microneedles are an elegant method for adequate drug delivery in scars. Corticosteroid embedded dissolving microneedles provide a painless and effective alternative to intralesional corticosteroid injections for hypertrophic scarring with the potential futuristic option of self-administration by patients.

*O.085*

**Achieving Optimal Hydration in Scar Management: Moisturizers versus Fluid Silicone Gels**

**Ignace De Decker, Henk Hoeksema, Anse Beeckman, Jozef Verbelen, Els Vanlerberghe, Petra De Coninck, Phillip Blondeel, Stan Monstrey and Karel Claes**
  Ghent University Hospital Burn Center, Department of Plastic, Aesthetic and Reconstructive Surgery

**Objectives**: Non-invasive scar management includes hydration by means of silicones gel sheets, fluid silicone gels or moisturizers. It is accepted that scar dehydration is characterized by raised trans epidermal water loss (TEWL), which can lead to increased fibroblast activity and thereby hypertrophic scar for mation. There is no consensus on how optimal hydration can be achieved: silicone sheets, liquid gels or moisturizers. Many disadvantages are associated with the use of silicone gel sheets including difficulties in applying the sheets, itching, irritation, or maceration of the skin. To avoid these complications and to facilitate the application, liquid silicone gels were developed. Various studies have shown that the effects are comparable to these of the sheets. Based on excellent clinical results after using three specific moisturizers in our patients, we wanted to investigate whether these moisturizers induce comparable occlusion and hydration compared with both each other and the widely recognized liquid silicone gels.
**Methods**: In total, 36 healthy volunteers participated in this study. Increased TEWL was created by inducing superficial abrasions skin stripping with adhesive tape. Three moisturizers and a fluid silicone gel were tested: DermaCress, Alhydran, Lipikar and BAP Scar Care silicone gel, respectively. TEWL-reducing capacities and both absolute (AAH) and cumulative (CAAH)-absolute-added hydration were assessed at different time points for up to 4 h.
**Results**: There was an immediate TEWL increase in the zones that had abrasions. Controls remained stable over time. The mean percentage reduction (MPR) in TEWL kept increasing over time with Alhydran and DermaCress. Silicone gel reached maximal MPR almost immediately post-application and only declined thereafter. The silicone gel never reached the minimal MPR of Alhydran or DermaCress. Hydration capacity assessed through CAAH as measured by the Corneometer was significantly less with silicone gel compared with the moisturizers. Compared with silicone gel Lipikar provided similar occlusion and the improvement in hydration was highly significant 4 h post-application.
**Conclusions**: Based on the results of both our previous research and this study, it is demonstrated that the occlusive and hydrative effect of fluid silicone gel is inferior to the moisturizers used in our center. Lipikar hydrates well, but is less suitable for scar treatment due to the lack of occlusion. A well-balanced occlusion and hydration in this study, which was only provided by Alhydran and DermaCress, suggests that moisturizers can be used as a scar hydration therapy that replaces silicone products, is more cost-effective and has a more patient-friendly application.

*O.086*

**An Evidence-Based Laser Therapy Algorithm for the Treatment of Burn Scars**

**Rajiv Sood**
  Burn and Reconstructive Centers of America

**Objectives**: Hypertrophic scarring after burn injury can be extremely painful, cause profound itching, and affect the way patients view themselves and how the outside world perceives them. We have utilized laser therapy as a modality for scar modulation for our patients since 2013. We completed a prospective IRB approved study to evaluate the outcome of scars treated with fractional CO2 laser therapy (FLT) utilizing both objective and subjective tools. Recently, we have completed a second prospective study evaluating the use of pulse dye laser (PDL) therapy and the impact on post-burn pruritis. In reviewing the outcomes from these two studies, we have developed an evidence-based laser therapy algorithm for burn scar management.
**Methods**: The FLT study entailed a series of three CO2 laser treatments minimally four to six weeks apart with scar measurements, and POSAS for m completion performed prior to each laser treatment and four weeks after the last FLT. Scar measurements that included color, pliability, and scar thickness, and completion of the POSAS for m were obtained prior to each laser therapy session and four weeks after the third laser treatment. The measurements of color, pliability, and scar thickness were measured with the Colorimeter, Cutometer, and ultrasound. The PDL study utilized the 5-D Itch scale to evaluate post-burn pruritis. A baseline measurement was obtained prior to any laser treatments. Each patient underwent two PDL sessions, and a 5-D itch scale was completed four to six weeks after the second PDL session. The baseline measurement was then statistically compared with the final 5-D itch scale measurement.
**Results**: Data from the FLT study showed statistically significant improvements in the Patient and Observer POSAS scores, patient-rated Itch score, scar thickness, and measured skin density. Changes to patient-rated scar pain, scar color, and pliability were noted but were not of statistical significance. Data from the PDL study showed a statistically significant decrease in the treated patients’ post-burn pruritis.
**Conclusions**: Based on these studies, we have developed a laser algorithm. All patients who qualify for laser therapy receive two PDL sessions that are four to six weeks apart followed by three FLT sessions. The use of both PDL and FLT decreases post-burn pruritis, decreases scar thickness, decreases pain, and increases patient satisfaction as shown in our research. The recent addition of IPL lasers for the management of folliculitis and hyperpigmentation is also included in our algorithm.

*O.087*

**Electronic Patient-Reported Outcome Measures in Burn Scar Rehabilitation: A Guide to Implementation and Evaluation**

**Jill Meirte ^1,2^ and Zephanie Tyack ^3^**
  ^1^  Oscare Organisation for Burns Scar After-Care and Research
  ^2^  University of Antwerp Department of Rehabilitation Sciences and Physiotherapy REVAKI-MOVANT
  ^3^  Australian Centre for Health Services Innovation (AusHSI) Queensland University of Technology

**Introduction and Objectives**: In burn scar rehabilitation, electronic patient-reported outcome measures (ePROMs) are increasingly being used in research and clinical settings as part of patient- and family-centred care. These measures can identify patients’ needs and monitor the therapeutic progress of both adults and children. The feedback of information from ePROMs to clinicians treating patients with scarring and psychosocial issues may have therapeutic benefits. However, testing the effectiveness of ePROMs used in the routine clinical care of patients with burn scarring is in its infancy, and one of the greatest challenges remains the implementation of ePROMs in real-world clinical settings. This implementation may be conducted as part of a research, clinical, or quality assurance initiatives; and targets healthcare clinicians as well as policy makers and researchers working in acute hospital, subacute, or after-care settings delivering burn care. The aim of this work is to present a guide for clinicians and researchers involved in burn scar rehabilitation to assist in implementing ePROMs in clinical settings.
**Method and Result Section**: The guide arose from two real-world case studies of ePROM implementation in burn scar clinics in Belgium and Australia. In addition, systematic reviews, best-practice guidelines, original studies in burns and scars, were used to for mulate the guide.
The two case examples will be presented: a digital care pathway, Scarpath Belgium, in a multi-disciplinary after-care centre, and the implementation of paediatric ePROMs in Australia (PEDS-ePROM study) in a children’s hospital. Aspects of the guide that will be presented including how to select outcome measures for use in routine clinical practice, paediatric administration, moving from paper-based to electronically administered PROMs, and strategies to support clinicians and managers in using ePROMs in routine clinical practice. Resources to support implementation that accompany the guide will also be presented.
Finally, ten recommendations for the implementation of ePROMs will be presented based on research evidence and the lessons learned by the authors.
**Conclusions**: The guide presented and accompanying resources should pave the way for ward for using and testing these ePROMs in research and practice.

*O.088*

**Comparative Study of Intralesional Injection of Triamcinolone and Verapamil-Triamcinolone in the Treatment of Keloids: A Single-Blinded Randomized Clinical Trial**

**Zahra Haghani Dogahe and Mohammadreza Mobayen**
  Burn and Regenerative Medicine Research Center, Guilan University of Medical Sciences

**Objectives**: Keloids are abnormal wound healing due to excessive collagen for mation, causing limitations and cosmetic issues for patients. Caregivers use various treatment modalities such as intralesional therapies, pressure therapy, radiotherapy, surgical excision, and combination therapies to prevent and treat keloids; however, to date, no standard treatment exists. We aimed to compare the effects of intralesional injection of triamcinolone and a combination of triamcinolone and verapamil on enhancing the Vancouver scar scale of patients with keloids following a surgical incision.
**Methods**: In this randomized single-blinded clinical trial, we included 32 patients with keloids due to surgical incisions on the trunk and limbs. Overall, 16 patients were in group T, receiving triamcinolone acetonide (concentration 40 mg/mL). Other patients were in group VT, receiving triamcinolone and verapamil (concentration 2.5 mg/mL). The ultimate permissible injected volume of drugs was two ccs, depending on the size of the scars. Injection intervals were three weeks. We continued injecting drugs until the complete flattening of the scar (scar height less than one mm) or a maximum of eight sessions. Demographics, anatomical site of the scar, different scar parameters, and Vancouver Scar Scale (VSS) were used to evaluate the efficacy of injections.
**Results**: Among 32 patients, the mean age was 36 years. The most common place for keloids was the abdomen. Changes in measurements of keloids were statistically meaningful (*p* < 0/0001) in both groups from the first session until the three-month follow-up session. In the VT group, skin redness changed to regular skin color faster and in fewer sessions. While most of the scars in both groups had similar VSS scores, changes in VSS during sessions were more appealing in group VT and ended in a lower score as well. In group T, the flexibility score was near one in the fifth session and stayed the same, whereas in group VT, regular flexibility of the scar was reached in the seventh session and stayed the same.
**Conclusions**: Our trial showed that intralesional injection of verapamil and triamcinolone is more efficient than triamcinolone alone in the treatment of keloids. Combination therapy reaches the optimum result in a shorter time and improves skin redness better than monotherapy. This adds to the previous evidence of the potential of verapamil in flattening the raised scars.

*O.089*

**Mechanomodulation: Physical Treatment Modalities Employ Mechanotransduction to Improve Scarring**

**Ulrike Van Daele ^1^, Jill Meirte ^1,2^, Mieke Anthonissen ^1,2,3^, Tine Vanhullebusch ^1^, Koen Maertens ^2,4^, Lot Demuynck ^1^ and Peter Moortgat ^2^**
  ^1^  Research Group MOVANT, University of Antwerp, Rehabilitation Sciences and Physiotherapy
  ^2^  Oscare
  ^3^  Department of Rehabilitation Sciences, KULeuven
  ^4^  Department of Clinical and Lifespan Psychology, Vrije Universiteit Brussel

**Objective**: Every year, surgical interventions, traumatic wounds, and burn injuries lead to over 80 million scars. These scars often lead to compromised skin function and can result in devastating disfigurement, permanent functional loss, psychosocial problems, and growth retardation. Today, a wide variety of nonsurgical scar management options exist, with only few of them being substantiated by evidence. The working mechanisms of physical anti-scarring modalities remained unclear for years.
**Methods**: This perspective literature study aims to translate research findings at the cellular and molecular levels into working mechanisms of physical anti-scarring interventions.
**Results**: Currently, scar mechanobiology research has resulted in the identification of a considerable number of signaling pathways, mainly those in fibroblasts and myofibroblasts, which are often abundant in pathological scars and are the main effector cells of excessive extracellular matrix (ECM) deposition and contraction in scars. Fibroblasts align and change their structure according to the direction of mechanical strain. Tension-induced skin fibrogenesis is dependent on ECM cross-linking and stiffening and several mechanosignaling pathways involved in scarring, including integrin-mediated transforming growth factor β (TGF-β) signaling, the integrin–FAK pathway, calcium ion signaling, Wnt/β–catenin signaling, etc.
Nearly every physical scar management introduces mechanical load (pressure garments, silicones, manual massage techniques, mobilizations, shock wave therapy, adhesive tape). Research indicates that lower loading rates than normal already initiate a response cascade, and loading rates previously indicated as normal in healthy tissue induce pathological scarring. The aim of every physical intervention on young, inflamed scars should therefore be to soften the ECM and cytoskeleton pre-stress by utilizing lower loading rates than normally used in healthy tissue.
**Conclusions**: Recent evidence underpinned the important role of mechanical for ces in scar remodeling, especially the balance between matrix stiffness and cytoskeleton pre-stress. Mechanomodulation of scars applied with the right amplitude, frequency, and duration induces ECM remodeling and restores the ‘tensile’ homeostasis. Depending on the scar characteristics, specific (combinations of) non-invasive physical scar treatments are possible. Future translational studies are needed to define the dose dependency of mechanical interventions in human in vivo scar models through controlling mechanical load application and through measuring cellular and molecular responses in scar tissue by (immune)histological and molecular pathways’ analysis. The effect of the ECM rigidity and inflammatory mediators on the dose dependency of mechanotherapy are highly relevant to include in future research. In addition, these studies should include the association between cellular and molecular changes and clinically relevant changes of the scar.

*O.090*

**Acute, Non-Excisional Debridement under General Anaesthesia in Paediatric Burns Decreases Time to Re-Epithelialisation and Risk of Skin Graft: A Cohort Study**

**Bronwyn Griffin, Anjana Bairagi, Maleea Holbert and Roy Kimble**
  Centre Oof Children’s Burns and Trauma Research, Griffith University

**Background**: Reported advantages of early excision for larger burn injuries include reduced morbidity, mortality, and hospital length of stay for adult burn patients. However, a paucity of evidence supports the best option for paediatric burns and the advantages of non-excisional (mechanical) debridement. Procedural sedation and analgesia in the emergency department is a popular alternative to debridement in operating theatres under general anaesthesia. This study aims to evaluate the association between early (<24 h post-injury) non-excisional debridement under general anaesthesia with burn wound re-epithelialisation time and skin graft requirements.
**Methods**: This was a cohort study of children younger than 17 years who presented with burns of 5% total body surface area or greater. Data from January 2013 to December 2019 were extracted from a prospectively collected state-wide paediatric burns’ registry. Time to re-epithelialisation was tested using survival analysis, and binary logistic regression for odds of skin graft requirement to analyse the effects of early non-excisional debridement in the operating theatre.
**Results**: Overall, 292 children met eligibility (males 55.5%). Early non-excisional debridement under general anaesthesia in the operating theatre, significantly reduced the time to re-epithelialisation (14 days versus 21 days, *p* = 0.029)) and the odds of requiring a skin graft in comparison to paediatric patients debrided in the emergency department under Ketamine sedation (OR: 6.97 (2.14–22.67), *p* < 0.001.
**Conclusions**: This study is the first to demonstrate that early non-excisional debridement under general anaesthesia in the operating theatre significantly reduces wound re-epithelialisation time (Table 1) and subsequent need for a skin graft in paediatric burn patients (Table 2). Analysis suggests that ketamine procedural sedation and analgesia in the emergency department used for burn wound debridement is not an effective substitute for debridement in the operating theatre.

*O.091*

**The Effect of Burns on Children’s Growth Trajectory: A Nationwide Study**

**Maxime Dominique Cuijpers ^1,2,3^, Pauline J.H. van de Sande ^1^, Charlotte I. Cords ^3,4^, Sonja M. H. J. Scholten-Jaegers ^5^, Paul P. M. van Zuijlen ^1,2,6,7^, Martin G. A. Baartmans ^8^ and Anouk Pijpe ^1,2^**
  ^1^  Red Cross Hospital—Burn Center Beverwijk
  ^2^  Amsterdam Unversity Medical Center—Department of Plastic, Reconstructive, and Hand Surgery
  ^3^  Association of Dutch Burn Centers
  ^4^  Maasstad Hospital—Burn Center Rotterdam
  ^5^  Martini Hospital—Burn Center Groningen
  ^6^  Red Cross Hospital—Department of Plastic, Reconstructive, and Hand Surgery
  ^7^  Emma Children’s Hospital, Amsterdam University Medical Center, Amsterdam Reproduction and Development Research Insititute—Department of Pediatric Surgery, University of Amsterdam, VU University Amsterdam
  ^8^  Maasstad Hospital—Department of Pediatrics

**Objective**: To evaluate the short- and long-term effect of burn injury on children’s height and weight, by comparing their pre- and post-burn growth trajectory.
**Methods**: We invited children (≤17 years old), who sustained a burn injury requiring surgical treatment and/or admission at one of the three Dutch burn centers in 2013 (*n* = 175). As well as children who sustained a severe burn injury, covering >10% of the total body surface area (TBSA), throughout 2009–2018 (*n* = 228). Data were collected from a survey on health-related topics, Youth Health Care records, and the Dutch Burn Repository R3. for all participants, height and weight were converted to Z scores using Dutch reference values. Linear mixed modelling, nested on individual level, was used to examine the associations between burn injury and children’s height and weight Z scores.
**Results**: Children’s height and weight Z scores remained within the normal range throughout the study period. during the first year post-burn, children’s height and weight Z scores decreased by −0.21 (95%CI [−0.41, −0.01]) and −0.23 (95%CI [−0.46, −0.04]), respectively. Beyond the first year post-burn, estimates were consistent with a positive linear association between burn size and the overall effect of burn injury on participants’ height and weight Z scores. Including a modest, but statistically significant, effect among participants with a burn injury covering ≤4.5% TBSA and >14.0% TBSA. Sensitivity analyses did not alter our findings.
**Conclusions**: Children were on track or even surpassed their growth potential. Our findings could therefore be considered reassuring to patients, parents, and clinicians.

*O.092*

**Factors That Influence Pain in Children Undergoing a Burn Dressing Change: A Retrospective Cohort Review of 2013 Paediatric Burn Patients**

**Maleea Holbert ^1,2^, Roy Kimble ^2,3^, Lee Jones ^4,5^, Samiul Ahmed ^2^ and Bronwyn Griffin ^1^**
  ^1^  School of Nursing and Midwifery, Griffith University
  ^2^  Faculty of Medicine, The University of Queensland
  ^3^  Pegg Leditschke Paediatric Burns Centre, Queensland Children’s Hospital
  ^4^  Faculty of Health, Centre for Healthcare Transformation, Queensland University of Technology
  ^5^  Research Methods Group, Queensland University of Technology

**Objectives**: Pain remains a major issue following a burn. Optimising pain management for paediatric burn patients is critical, as untreated pain can lead to prolonged wound re-epithelisation. There is an absence of evidence regarding predictors of moderate to severe pain in children undergoing acute burn treatment. This investigation aimed to determine if relationships existed between patient and clinical characteristics, and pain at first dressing change for children with acute burn injuries.
**Methods**: A retrospective cohort investigation was conducted using clinical data from paediatric burn outpatients treated at the Queensland Children’s Hospital, Brisbane, Australia. Data extracted included patient and burn characteristics, first aid, and follow-up care. Observational pain scores were categorised into three groups (mild, moderate, and severe pain), and bivariate and multivariable relationships were examined using proportional odds ordinal logistic regression. Data from February 2016 to July 2019 were extracted and included for analysis (*n* = 2013).
**Results**: Factors associated with increased odds of procedural pain included: hand burns (OR 1.7, 95% CI 1.3 to 2.1, *p* < 0.001), foot burns (OR 1.5, 95% CI 1.1 to 2.1, *p* < 0.01), baseline pain (OR 5.5, 95% CI 2.8 to 10.8, *p* < 0.001), deep dermal partial-thickness injuries (OR 7.9, 95% CI 4.0 to 15.6, *p* < 0.001), increased burn size (OR 1.1, 95% CI 1.0 to 1.2, *p* < 0.01), four or more anatomical regions burned (OR 3.6, 95% CI 1.5 to 8.6, *p* < 0.01), initial treatment at a non-burns centre (OR 1.8, 95% CI 1.4 to 2.3, *p* < 0.001), and time to hospital presentation (OR 0.9, 95% CI 0.8 to 0.9, *p* < 0.001). These burn characteristics are associated with increased odds of moderate to severe procedural pain during a child’s first dressings change.
**Conclusions**: A better understanding of factors that influence pain in paediatric burn patients is vital to establish effective pain management guidelines for children with burn injuries, and to recognise patients who will require a more aggressive and targeted approach to pain management during their burn treatment. It is recommended that paediatric patients presenting with one or more of the aforementioned factors are identified before their first dressing change, so additional pain control methods can be implemented.

*O.093*

**High-Voltage Electrical Burn in Children**

**Hana Fredj, Abir Khorchani, Amel Mokline, Wala Brahmi, Imen Jami, Bahija Gasri, Manel Ben Saad and Amen Allah Messadi**
  Burn Intensive Care Unit, Traumatology and Burn Center

**Introduction**: In the literature, electrical burns are rare in the pediatric population. This pathology is potentially serious with disabling systemic and functional consequences and significant mortality. The aim of our study was to assess the incidence and prognosis of high-voltage electrical burns in children admitted to intensive care burn unit.
**Methods**: A retrospective study was conducted on children with electrical injuries hospitalized in the Intensive care Burn department in Tunis for four years and 10 months (March 2017–December 2021). Demographic, clinical, therapeutic and evolutionary data were collected and analyzed.
**Results**: during the study period, the total number burn admissions was 1918. Overall, 162 patients were victims of high-voltage electrical burns (8.4%), including 24 children (15%). The mean age of the children was 12 ± 4 years [4–18] with a male preponderance (*n* = 22).
The HVEB occurred on the rooftop of houses in 42% of cases. In 33% of cases, it was a leisure accident and in 12% an occupational accident. Overall, 12% of burns occurred during copper theft attempt. Mean Total Body Surface Area burned was 19% [2–74]. In total, 9 patients (38%) presented a compartment syndrome and required emergency fasciotomy. CPK elevation was noted in 19 cases with an average rate of 20736 UI/L [2030; 85558]. Renal failure occurred in one case. Myocardial involvement was present in 17 patients (70%) with a mean troponin of 229 ng/mL (11 times normal). Management was essentially based on hydroelectrolytic resuscitation.
Overall, 7 children had at least one limb amputated. The length of stay was 24 days [2,137].
**Conclusions**: In our study, the incidence of high-voltage electrical burn in children is high. It is associated with high rates of amputation (24%) and morbidity. Most of these injuries occurred on the rooftop of homes. Preventive measures especially the education of children and their awareness of the dangers of high-tension wires as well as fighting against anarchic construction in high-risk areas is necessary to prevent rooftop burns.

*O.094*

**Fatal Hyperacute Liver Failure in Acute Burned Children after Application of a Primary Wound Dressing Containing Polyhexanide Biguanide 0.3%**

**Cesar Centeno ^1^ and Sergio Lopez-Briones ^2^**
  ^1^  Instituto de Salud Publica del Estado de Guanajuato
  ^2^  Department of Medicine and Nutrition Health Sciences Division, Universidad de Guanajuato

**Objectives**: The aim of this study was to describe the association between using 0.3% polyhexanide biguanide dressings and fatal liver failure in children with acute burns.
**Methods**: a retrospective study was carried out in which 34 medical records from March 2018 to February, 2020 of children hospitalized for burns and treated with Suprasorb X^®^ or Suprasorb X^®^ + PHMB 0.3% were analyzed. To determine the effect of treatment on liver function, several serum parameters, such as: liver enzymes (AST and ALT), serum clotting times (prothrombin time and partial thromboplastin Time), leukocyte and platelet account, bilirubin, electrolytes (Ca and K), CRP, hemoglobin, albumin, glucose, and creatinine were evaluated by conventional laboratory methods. In addition, clinical changes were also registered.
**Results**: All the cases were children previously stable and without known factors of increased mortality before the application of the studied dressing. Treatment with PHMB 0.3% dressings was found to induce adverse reactions. Liver damage appeared in the first 24 to 48 h after exposure. In all children who died after treatment, plasma levels of AST, ALT, prothrombin time, partial thromboplastin time and number of leukocytes were significantly increased. Despite the low number of cases, we observed that the possible toxic effect of the treatment with PHMB 0.3% was dependent on both the dose and the elapsed days between burn and treatment application.
**Conclusions**: Here, we found that the application of 0.3% polyhexanide biguanide dressings on second-degree acute burn wounds in children, was associated with adverse reactions, including hyperacute liver failure and death, in a dose- and time-dependent relationship. We do not rule out the possible influence of other factors, such as genetic or environmental factors.

*O.095*

**The Use of a Unique Silicone-Lined Thermoplastic to Fabricate a Portfolio of Orthoses to Manage Burn Scar Hypertrophy and Contractures**

**Michael Serghiou and Jonathan Niszczak**
  Bio Med Sciences Inc.

**Introduction**: Recent advancements in medicine have vastly improved the survival chances of burn patients. The focus of the burn recovery has now shifted from survival to early rehabilitation. Our objective is to illustrate the use of a unique thermoplastic to prevent contractures and flatten hypertrophic scars. We have designed six head, one neck and three upper extremity splints that, if initiated early, could prevent contractures and deformities.
**Methods**: A low-temperature silicone-lined thermoplastic is utilized for the fabrication of the splints. The chin splint cups the chin and reverses lower lip eversion. The mouth splint is designed to stretch both commissures vertically. One nose splint is designed to expand the nostril diameter and the other is designed to depress scar hypertrophy around the nasal bridge-ala-epicanthal region. One of the ear splints is designed to increase the ear canal diameter and the other prevents the ear helix from contracting toward the head. The upper extremity splints are designed to soften the scar and increase ROM at the hand, wrist, and elbow joints.
**Results**: We have found that utilizing these splints at the first evidence of scar hypertrophy or tightness, outcomes can be very positive. Nostril and ear canal diameters can increase by 5 mm in about 10 days. Vertical and horizontal mouth opening can increase between 1 cm–1.5 cm in approximately 1 month. Lower lip eversion and scar hypertrophy around the ala/nasal bridge/epicanthal region can be inhibited when the chin and nose splint are worn underneath a garment mask. Wrist and elbow extension can increase about 10–15 degrees in 2 weeks.
**Discussion**: The unique splinting material is coated with silicone which provides for comfortable contact to the skin. The combination of silicone and thermoplastics in splinting theoretically enhances the principles of gentle, prolonged sustained stretch and promotes scar hydration/pliability that could lead into softening and elongation of tissues, and flat scars. The material can be adjusted multiple times and can be utilized in static progressive splinting successfully.

*O.096*

**Efficacy of Exercise Rehabilitation during the Acute Phase of Burns: A Multicenter Trial**

**David R. Schieffelers ^1^, Dorien Dombrecht ^1^, Eric van Breda ^1^, Cynthia Lafaire ^2,3^, Lieve De Cuyper ^2,3^, Thomas Rose ^4^, Nick Gebruers ^1,5^, Jill Meirte ^1,3^ and Ulrike Van Daele ^1,3^**
  ^1^  Multidisciplinary Metabolic Research Unit (M2RUN), MOVANT Research Group, Department of Rehabilitation Sciences and Physiotherapy, Faculty of Medicine and Health Sciences, University of Antwerp
  ^2^  Burn Unit, ZNA Stuivenberg
  ^3^  OSCARE, Organization for Burns, Scar After-Care and Research
  ^4^  Burn Unit, Military Hospital Queen Astrid
  ^5^  Multidisciplinary Edema Clinic, Antwerp University Hospital

**Objectives**: Exercise training is often only commenced once the acute phase of burns has passed. However, it is during this phase that most losses of muscle mass and muscle strength occur, and during which exercise might be most potent. This multicentre trial explored whether exercise could limit the amount of muscle wasting and muscle weakness experienced during burn centre stay.
**Methods**: Adult patients with burns ≥10% TBSA were allocated to receive an exercise intervention consisting of resistance and aerobic training 3–5 times per week in addition to standard care or standard care alone. The exercise intervention commenced as early as possible according to pre-determined starting and safety criteria, and lasted for up to 8 weeks depending on hospital discharge. Muscle wasting was quantified by B-mode ultrasound-derived quadriceps muscle layer thickness (QMLT) and the rectus femoris cross-sectional area (RF-CSA), and muscle strength was determined by handheld-dynamometry of grip strength, hip flexion, knee extension for ce. Preliminary analysis was carried out using mixed effects models with the group, time from baseline, and their interaction term as fixed effects, and subjects as random effects. Covariates of interest were added to the model in a stepwise for ward fashion to better explain the observed variance.
**Results**: In total, 58 adults (16 females; 51 ± 15 years) with burns 23 ± 15% TBSA (range 10–70%) with an average burn centre stay of 45 ± 34 days were allocated to the exercise group (*n* = 28) or control group (*n* = 30). Groups were comparable at baseline. Multivariable regression analysis demonstrated that the declines in QMLT, RF-CSA, grip strength, and hip flexion for ce over time were significantly less in the exercise group compared with the control group. There was no significant difference in knee extension for ce over time between groups. In all cases baseline values of the outcome of interest had a significant impact on the rate of decline over time, as such that those with highest baseline values showed the largest decline. Final models with β-coefficients for main effect and interaction effect terms per outcome are provided in Table 1.
**Conclusions**: Exercise training carried out during the acute phase of burns attenuates muscle wasting and muscle weakness throughout the duration of stay at the burn centre. Patients with higher muscle mass and strength are particularly affected by muscle wasting and weakness.

*O.097*

**Elastic Adhesive Tape in Post-Burn Scars, a Preliminary Study in Rehabilitation**

**Danila Toscano, Daniela Arena, Lorena Sarzi, Maurizio Stella, Nadia Depetris and Giuseppe Massazza**
  Città Della Salute e Della Scienza—(CTO) Orthopedic and Trauma Center

**Objectives**: The treatment and rehabilitation of post-burn scarring time, on average over two years, requires evaluating the possibility of using and implementing innovative treatments to shorten the period of scarring time. The best method of description is the structured observation of the treatment and its results.
The experience gained in the management of burn scars has made it possible to prepare and share a protocol (in line with international guidelines) for the rehabilitation of burn scars in clinical practise, the treatment with adhesive tape as a benefit for healing. Comparatively effective and safe compared with standard treatment.
**Methods**: Since 2011, the current rehabilitative protocol has been integrated with the treatment using elastic tape. The application of the tape is done by an experienced physiotherapist once a week, upon remission of the blistering phase. after the application, the patient is instructed on how to maintain and remove the tape and can enact physiotherapeutic actions to counter the phenomena of the retracting scar, in addition to wearing elastic compression sheaths for the prevention and or treatment of hypertrophy.
The rehabilitation treatment, integrated with the application of adhesive elastic tape, was applied to 84 patients with post-burn scarring with a TBSA (Total Burn Surface Area) greater than 9% in the following body areas: upper and lower limbs, torso, face, and cleavage. Patients with an allergy to the glue of the tape, patients with poor compliance, open lesions and skin pathologies were excluded from the treatment.
**Results**: All the patients involved have reported greater comfort after treatment, in terms of better skin elasticity, reduction in the inflammation of the scar area, in addition to benefits related to the symptoms of burning, itching and pain. The integration of this innovation with tension kinesiotherapy has improved the extensibility of retracting scars, scar adhesions and the aesthetic appearance of the scar.
**Conclusions**: This innovation, having already been introduced in rehabilitation programs for injuries in other situations other than burns, could decease the rehabilitation time of patients. However, the supporting evidence is still ongoing.
The integration of the adhesive elastic facilitates the treatment of scar areas that are difficult to treat with an elastic compression sheath, for example the neck, face and cleavage.
Furthermore, the low number and heterogenous baseline characteristics of these patients do not allow the design of a study or studies that test the clinical relevance of its use.

*O.098*

**Assessment of Burn Scar Contracture: A Call for Change**

**Marianne Nieuwenhuis ^1,2,3^, Anouk Oosterwijk ^2^, Hennie Schouten ^4^, Thom Hendriks ^5,6^, Matthijs Botman ^5,6^, Anuschka Niemeijer ^1,7^, Cees van der Schans ^3,8,9^, Noor Mouton ^2^ and Paul van Zuijlen ^4,5,6^**
  ^1^  Association of Dutch Burn Centres, Burn Centre Groningen, Martini Hospital
  ^2^  Department of Human Movement Sciences, University Medical Centre Groningen
  ^3^  Research Group Healthy Ageing, Allied Health Care and Nursing, Hanze University of Applied Sciences
  ^4^  Burn Center, Red Cross Hospital
  ^5^  Department of Plastic, Reconstructive and Hand Surgery, Amsterdam UMC
  ^6^  Global Surgery Amsterdam
  ^7^  Research Institute, Martini Hospital
  ^8^  Department of Health Psychology, University Medical Center Groningen
  ^9^  Department of Rehabilitation Medicine, University Medical Center Groningen

**Objectives**: Scar contractures are a persistent sequela of burns. The existence and severity of scar contractures are based on the assessment of joint range of motion (ROM) and are used to evaluate the effect of (new) treatments or analyze its prevalence and risk factors. To this end, ROM of a specific joint and movement direction is usually compared with norm values of the maximal ROM and assessment is mostly at discharge. The significance, however, of scar contractures lies in their limiting effect on function, and the impact they have on daily activities, participation and quality of life. Furthermore, it is questionable whether assessment at discharge represents scar contracture. Thus, this practice is in need of change.
**Methods**: First, in a number of studies, we investigated norm ROM in relation to ROM required to perform activities in daily life, and functional ROM. Secondly, our group conducted several prospective longitudinal studies with long term follow-up concerning recovery of ROM after burns, including, but not limited to discharge.
**Results**: for some joints and movement directions, norm ROM approximated functional ROM; for others, there was a large discrepancy between the two. Comparing the ROM of a specific joint and movement direction to function-based ROM cut-off values instead, gave a better understanding of the impact of ROM loss on activities of daily life. Regarding recovery, the prevalence and severity of ROM loss changed over time and were different per joint and movement direction. Furthermore, prevalence and severity at discharge were not predictive of outcome at 12 months after injury.
**Discussion**: Ours and most other studies so far have used standardised protocols assessing ROM in the anatomical position. This practice was recently challenged by the group of Parry and Richards et al. They showed that standard goniometry underestimated ROM impairments in patients with burn scars and proposed a revised goniometry protocol based on cutaneous functional units and functional positions of joints.
**Conclusions**: As the significance of scar contractures lies in their limiting effect on function, we strongly plead for assessment to reflect this. The assessment of ROM should furthermore be carried out with the revised goniometry protocol, and not be restricted to discharge, but involve a longer period, preferably until the end of scar maturation. Only this way will we obtain a better understanding of the impact of scar contractures and ways to improve the outcome of our treatments for patients.

*O.099*

**A Retrospective Study on Patients Admitted to the Major Burn Centre of the CTO of Turin with Outcomes Attributable to the Illness of a ‘Critically Ill Patient’**

**Lorena Sarzi, Laura Olino, Daniela Arena, Danila Toscano, Anna Morra, Nadia Affilastro and Giuseppe Massazza**
  Orthopedic and Trauma Center—Città Della Salute e Della Scienza, Physical Medicine and Rehabilitation University

**Targets**: The evaluation of the prevalence of patients admitted to the severe burn centre over a three-year period, 2019–2021, with outcomes correlating to the ‘critically ill patient’ in addition to our experience and comparison with literature data.
**Methods**: Patients who developed Critical Illness Polyneuropathy were reviewed, analysing risk factors such as the percentage of TBSA, period of assisted ventilation, sedation, immobility, and sepsis. Meanwhile considering the repercussions of rehabilitation treatment. The evaluation was completed using the Barthel scale at discharge and during remote follow-up.
**Results**: The rehabilitation programme implemented makes use of a first/second day visit from physiotherapy and the start of treatment with dedicated physiotherapists. In the acute department of the severe burns centre, the rehabilitation treatment includes, from the outset, a predominantly passive mobilisation therapy in bed, for the maintenance and recovery of motion. The therapy includes the recovery of muscle length together with a correct positioning, aimed at preventing both edema and venous stasis and incorrect postures with the use of orthoses.
Based on the evolving situation, with the suspension of sedation and the gradual decline and use of ventilator supports, the rehabilitation treatment is intensified and integrated with constant evaluation from dedicated respiratory physiotherapists and speech therapists. In compliance with the surgical timeline, mobilisation is intensified with passive but also active assistance and active techniques, with readjustment to the sitting position with legs out, up to standing and walking. The treatment is also extended to included weekends and respects the potential tiredness of the patient.
We have observed that patients with a higher TBSA rate and who have endured a clinical course complicated by severe septic episodes had their mobilisation treatment influenced by the achievement of haemodynamic and respiratory balance. These patients had a higher percentage of CIP/CIM.
**Conclusions**: The examined percentage of the prevalence of patients with critical illness polyneuropathy (during the above defined three-year period and confirmed by clinical examination and the literature data) was in line with the verified results (circa 4–5%).
The CIP influenced the longer hospitalisation times and the functional recovery documented by the lower Barthel index, with criticalities in identifying the specific settings of discharge.

*O.100*

**Correlation between Muscle Wasting and Muscle Weakness in Adult Burns throughout Burn Centre Stay**

**David R. Schieffelers ^1^, Eric van Breda ^1^, Cynthia Lafaire ^2,3^, Lieve De Cuyper ^2,3^, Thomas Rose ^4^, Nick Gebruers ^1,5^, Jill Meirte ^1,3^, Dorien Dombrecht ^1^ and Ulrike Van Daele ^1,3^**
  ^1^  Multidisciplinary Metabolic Research Unit (M2RUN), MOVANT Research Group, Department of Rehabilitation Sciences and Physiotherapy, Faculty of Medicine and Health Sciences, University of Antwerp
  ^2^  Burn Unit, ZNA Stuivenberg
  ^3^  OSCARE, Organization for Burns, Scar After-Care and Research
  ^4^  Burn Unit, Military Hospital Queen Astrid
  ^5^  Multidisciplinary Edema Clinic, Antwerp University Hospital

**Objectives**: The assessment of muscle for ce is often challenging in the acute burn setting, where sedation, patient cooperation, and pain, amongst other factors, limit its validity. Ultrasound measurement of muscle thickness has been suggested as an alternative; however, it is unclear whether muscle thickness and muscle for ce are representative of one another in the acute burn setting. The aim of this study was to define the relationship between changes in muscle thickness and muscle for ce throughout hospitalisation.
**Methods**: This is a secondary analysis of adults with acute burn injury enrolled in a multicenter trial of early exercise. Subjects with burns ≥10% TBSA and without history of conditions interfering with muscle assessment were eligible if they had undergone two or more simultaneous assessment of the quadriceps muscle layer thickness (QMLT) and the rectus femoris cross-sectional area (RF-CSA) by B-mode ultrasound, and peak for ce measurements of grip strength, hip flexion, knee extension by handheld dynamometry throughout hospital stay. Time points of assessment varied per patient according to alertness, cooperation, medical safety, and hospital discharge. Pearson’s correlation coefficients were calculated to assess the relationship between ultrasound and muscle for ce parameters.
**Results**: In total, 55 adults (15 females; 49 ± 14 years) with burns encompassing 23 ± 15%TBSA provided 73 change scores in muscle parameters. Changes in ultrasound-derived muscle thickness were moderately correlated with changes in grip strength (QMLT r = 0.49, *p* < 0.001; RF-CSA r = 0.4, *p* < 0.004) and hip flexion (QMLT r = 0.45, *p* < 0.001; RF-CSA r = 0.41, *p* < 0.002), but not to knee extension (QMLT r = 0.22, *p* = 0.142; RF-CSA r = 0.19, *p* = 0.206).
**Conclusions**: The results of this analysis show that, despite the limited overlap, ultrasound-derived muscle thickness and muscle for ce are not interchangeable, and should therefore be measured separately when tracking changes in musculature throughout burn centre stay. Both muscle thickness and muscle for ce describe distinct features that should not replace one another. Qualitative muscle parameters and neurodegenerative changes might amongst others explain why muscle thickness and muscle for ce are not better correlated.

*O.101*

**Somatosensory Rehabilitation of Neuropathic Pain in Burn Survivors: A Case Report**

**Miranda Venema**
  Martini Ziekenhuis

**Objectives**: Burn survivors often complain of pain which can be defined as neuropathic pain. Neuropathic pain affects their recovery and reintegration and ultimately the patients’ quality of life. The prevalence of chronic neuropathic pain was 6% according to the study from Klifto et al. (2020) [1]. A study examining the prevalence of chronic pain showed that neuropathic pain was prevalent in one third of the adult burn survivors [1]. There is a paucity of literature about treatment of neuropathic pain in burn survivors. Most describe treatment with medication and suggest that research into other potential treatment approaches is needed. The international association for study of pain (IASP) defines neuropathic pain as “pain caused by a lesion or disease of the somatosensory system”. Somatosensory rehabilitation of neuropathic pain (SRNP) is an intervention described by Claude Spicher based on the neuroplasticity of the somatosensory system [2]. This method diagnoses, treats and evaluates neuropathic pain. Concerning burn survivors, there is only one study about SRNP [3]. This study showed that SRNP seems to reduce the symptoms of neuropathic pain in burn survivors, and there are no apparent side effects to this treatment.
**Methods**: We report on a case of a 51-year-old male patient with burns on the dorsal side of the third finger caused by a pipe with a temperature of 200 degrees during his work. The burn was excised and grafted 9 days post-burn. Two weeks later, the graft was fully re-epithalised. The patient subsequently complained about pain in his finger. Ten months later, he still had pain, and he cannotcould not work because of the neuropathic pain, caused just by wearing a glove during his work.
**Results**: This patient had been treated with SRNP; he performedunderwent his home program daily, and he came to the occupational therapy every month for 6 months. after the treatment, the patient was free of pain. He is now working without any problem.
**Conclusions**: SNRP is frequently used, with good results, in hand rehabilitation. According to one study in patients with burns [3] and our own experiences, it can also be effective in the rehabilitation of burn survivors. However, the cases are too few to draw a definitive conclusion. It is a good start for further research in the future.

**References**
  1. Klifto, K.M.; Dellon, A.L.; Hultman, C.S. Prevalence and associated predictors for patients developing chronic neuropathic pain following burns. *Burns Trauma* **2020**, *8*, tkaa011. https://doi.org/10.1093/burnst/tkaa011.
  2. Spicher, C. *Handbook for Somatosensory Rehabilitation*; Sauramps Médical: Baltimore, MD, USA, 2006.
  3. Nedelec, B.; Calva, V.; Chouinard, A.; Couture, M.-A.; Godbout, E.; de Oliviera, A.; LaSalle, L. Somatosensory Rehabilitation for Neuropathic Pain in Burn Survivors: A Case Series. *J. Burn Case Res.* **2016**, *37*, e37–e46. https://doi.org/10.1097/BCR.0000000000000321.

*O.102*

**Lifelong Fitness Testing: The Steep Ramp Test in Dutch adults and Elderly: Age- and Sex-Related Norm Values, and Its Reproducibility, Validity, and Underlying Physiological Responses**

**Ingeborg A. Trul-Kreuze ^1,2^, Moniek Akkerman ^1,3^, Bart C. Bongers ^4,5^, Marianne K. Nieuwenhuis ^1,2,6^ and LIFT Consortium ^1,2,3,4,5,6,7,8^**
  ^1^  Association of Dutch Burn Centres, Burn Centre Groningen, Martini Hospital
  ^2^  Hanze University of Applied Sciences, Research Group Healthy Ageing, Allied Health Care and Nursing
  ^3^  Burn Centre Groningen, Martini Hospital
  ^4^  Department of Nutrition and Movement Sciences, School of Nutrition and Translational Research in Metabolism (NUTRIM), Faculty of Health, Medicine and Life Sciences, Maastricht University
  ^5^  Department of Epidemiology, Care and Public Health Research Institute (CAPHRI), Faculty of Health, Medicine and Life Sciences, Maastricht University
  ^6^  University of Groningen, University Medical Center Groningen, Department of Human Movement Sciences
  ^7^  University Medical Center Utrecht, Child Development & Exercise Center
  ^8^  Lode Holding B.V.

**Objectives**: One of the potential consequences of burns is a reduction in cardiorespiratory fitness (CRF). As CRF is predictive for (long-term) health and functioning, the recovery of CRF after hospital discharge is important. The gold standard for CRF assessment is determining the maximal oxygen uptake (VO2max) attained during a cardiopulmonary exercise test (CPET). However, the CPET is burdensome, especially for patients, and requires expensive equipment and specialized knowledge. From daily clinical (burn care) practice, there is an urgent need for a simple and feasible exercise test that can validly and reliably estimate an individual’s CRF. The steep ramp test (SRT) is such a short maximal exercise test on a cycle ergometer. Its main outcome measure, the highest work rate attained during the test (Wrpeak), is highly correlated with VO2max attained at a CPET in several (patient) populations. However, sex- and age-specific norm values for adults and the elderly are lacking thus far, which seriously limits the interpretation of the test results. The primary aim of this study is therefore to collect a set of representative sex- and age-specific norm values for SRT performance in healthy adults, including elderly (group 1). The secondary aims are to investigate the test–retest reliability of the SRT (group 2), as well as its validity to assess CRF, and the underlying physiological responses during SRT performance (group 3) in healthy adults and elderly.

**Methods**: Those eligible for this study are Dutch adults aged between 25 and 85 years, without evident health conditions, and without contra-indications for maximal exercise. A total of 720 participants will be recruited: *n* = 600 to perform a single SRT, *n* = 60 to perform the SRT twice with 1–2 weeks between both tests (group 2), and *n* = 60 to perform a series of isometric and isokinetic dynamometry, a SRT with respiratory gas analysis, electrocardiography, and blood pressure assessment, and a CPET (group 3). Age-related norm values for SRT performance will be constructed for both men and women, using Generalized Additive Models for Location, Scale and Shape (GAMLSS).
**Results (expected)**: Sex- and age-specific norm values for SRT performance in healthy adults, including elderly. Furthermore, we hope to gain an insight in testinto test–retest reliability and criterion validity of the SRT to evaluate CRF, and insights into cardiovascular, pulmonary, and metabolic responses during SRT performance.
**Conclusions:** Norm values for adults and elderly will increase the applicability of the SRT. Using the SRT to monitor the CRF of burn patients after hospital discharge, we will support burn care professionals to provide tailored care.

*O.103*

**Skeletal Muscle Thickness Assessment with Ultrasonography Is Reliable in Hospitalized Patients with Burns**

**Frederiek G. Bosch ^1,2^, Harriët Jager-Wittenaar ^2,3^, Gretha C. Wesseling-Keuning ^4^, Jakob Hiddingh ^5^, Hans J. Eshuis ^5^, Anuschka S. Niemeijer ^1^, Cees P. van der Schans ^2,6,7^ and Marianne K. Nieuwenhuis ^1,2,8^**
  ^1^  Association of Dutch Burn Centres, Burn Centre Groningen, Martini Hospital
  ^2^  Research Group Healthy Ageing, Allied Health Care and Nursing, Hanze University of Applied Sciences
  ^3^  Department of Oral and Maxillofacial Surgery, University of Groningen, University Medical Center Groningen
  ^4^  Department of Dietetics, Martini Hospital
  ^5^  Burn Centre Groningen, Martini Hospital
  ^6^  Department of Rehabilitation Medicine, University of Groningen, University Medical Center Groningen
  ^7^  Department of Health Psychology Research, University of Groningen, University Medical Center Groningen
  ^8^  Center for Human Movement Sciences, University of Groningen, University Medical Center Groningen

**Objectives**: Major burn injury causes inflammation and a prolonged hypermetabolic state, resulting in muscle loss. Muscle loss is associated with impaired physical functioning and decreased quality of life. To enable adequate analysis and monitoring of muscle mass, reliable and valid bedside assessment techniques are needed. Ultrasonography is gaining interest as technique to assess and monitor skeletal muscle thickness (SMT). In this observational longitudinal study, we aimed to determine intrarater and interrater reliability of SMT assessment with bedside ultrasonography in hospitalized patients with burns.
**Methods**: Adult patients with burns of ≥5% of their total body surface area (TBSA) were eligible for inclusion. SMT was assessed using A-mode ultrasonography, by five raters from different disciplines, i.e., a dietitian, three nurses and a physical therapist. SMT was assessed on the upper arm and leg, on the day of admission, post-burn dayand then on days 1, 3, 7, 10 post burn, and, subsequently, weekly until discharge. Only non-burned sites were assessed. To evaluate intrarater and interrater reliability, assessments were performed by the same rater twice or by two different raters. Reliability was determined in two ways, i.e., as procedural and measurement reliability, and evaluated by intraclass correlation coefficients (ICCs; two-way random, absolute agreement) with 95% confidence intervals (CI).
**Results**: In total, 34 patients, of which 9 were admitted to intensive care, were included. A total of 975 pairs of ultrasonography images were used for analyses. Procedural intrarater reliability was excellent (ICC 0.96; 95% CI 0.95–0.97). Procedural interrater reliability was good (ICC 0.84; 95% CI 0.76–0.89). Measurement intrarater and interrater reliability were both excellent (ICC 0.95; 95% CI 0.94–0.96, and ICC 0.90; 95% CI 0.86–0.93, respectively).
**Conclusions**: SMT assessment with bedside ultrasonography in hospitalized patients with burns is reliable, both within and between raters.
Powered by Health~Holland, Top Sector Life Sciences & Health.

*O.104*

**The Trigger Tool as a Method to Measure Adverse Events in a Burn Center**

**Paolo Marannino, Giuseppe Giudice, Pasquale Tedeschi, Aurelia De Pascale and Alessio De Cosmo**
  Plastic Surgery

A high frequency of medical errors indicates that more improvements in patient safety are needed in healthcare environments. Recently, the IHI Global Trigger Tool (GTT) has been developed to monitor adverse event rates, while working to improve patient safety.
In this study, for the first time, according to our literature research, we evaluate the efficacy of GTT in detecting triggers of possible events in burn patients, and we set new and specific triggers to be used for these patients.
In total, 60 sets of medical records belonging to patients admitted to the Burns Center of “Policlinico di Bari” were randomly selected in accordance with the selection criteria set out by the IHI.
During the analysis, it was considered opportune to introduce eight further triggers specifically for burns patients and referred to as the Burn Trigger Tool (BTT).
Results show that the new set of specific triggers (BTT) is very useful in detecting adverse events in burn patients; in our experience, the integration of these new triggers with GTT clinical triggers can provide a faster and more efficient evaluation of clinical risk in a burn center, improving and making healthcare safer for burn patients.

*O.105*

**Experience Experts as Team Members—Involvement of Burn Survivors in Burn Care and Research**

**Moniek Akkerman ^1,2^, Marianne K Nieuwenhuis ^1,3,4^, Jakob Hiddingh ^2^, Gerbrig Bijker ^2^, Anouk Pijpe ^5^, Robin Verwilligen ^6^, Matthea Stoop ^6^, Margriet E. van Baar ^7,8^, W. A. Ringelberg ^9^, Marscha C. Heijblom ^7^, Gert Versluis ^9^, Hendriët Wanders ^10^, Lotte van Dammen ^11^ and Carine van Schie ^11^**
  ^1^  Association of Dutch Burn Centres, Burn Centre Groningen, Martini Hospital
  ^2^  Burn Centre Groningen, Martini Hospital
  ^3^  Department of Human Movement Sciences, University of Groningen, University Medical Center Groningen
  ^4^  Research Group Healthy Ageing, Allied Health Care and Nursing, Hanze University of Applied Sciences
  ^5^  Association of Dutch Burn Centres, Burn Centre Beverwijk, Rode Kruis Hospital
  ^6^  Burn Centre Beverwijk, Rode Kruis Hospital
  ^7^  Association of Dutch Burn Centres, Burn Centre Rotterdam, Maasstad Hospital
  ^8^  Department of Public Health, Erasmus Medical Center, University Medical Center Rotterdam
  ^9^  Burn Centre Rotterdam, Maasstad Hospital
  ^10^  Dutch Association of Burn Survivors
  ^11^  Dutch Burns Foundation

**Objectives**: Three Dutch burn centres aim to improve patient-centred burn care. A value-based health care (VBHC) framework will be developed in which process indicators and patient-reported outcome measures are analysed and interpreted, and its results implemented in clinical practice. This VBHC framework will be built on data registries on patient and injury characteristics and clinical processes (Dutch Burn Repository R3) and outcomes (Burn centre Outcomes Registry Netherlands). The word ‘value’ in the VHBC framework refers to what outcomes are considered to be of value by a patient. Burn survivors, or experience experts, know best which outcomes matter to them and what was important for them during the different phases of recovery after burns. Their involvement is essential to realize the development of this framework. To this end, we aimed to for m patient panels.
**Methods**: A working group ‘active patient involvement’ was created, initially consisting of burn care professionals and researchers from the three Dutch burn centres. for mer patients from each burn centre were selected and informed (verbally or in writing) about the possibility of participation in a patient panel. Patients who were interested received an information brochure concerning general information about the importance of their involvement in burn care and research, and the various ways they can contribute.
**Results**: Each of the three Dutch burn centres has now for med its own patient panel, consisting of 5–15 experience experts. We started with an online meeting in which both patients and working group members introduced themselves and shared their previous experiences with active patient involvement. Furthermore, a timeline was presented showing the activities to which patients could contribute in the upcoming months. The extent of involvement can vary from being informed (e.g., newsletters, presentations, webinars), to providing information (e.g., sharing experiences, filling out questionnaires), providing solicited and unsolicited advice and feedback (on project proposals, patient information, burn care procedures, et cetera), co-operating actively in burn care and research activities (e.g., conducting interviews, informing patients, presenting results), or even initiating research activities. Depending on individual wishes and possibilities, an appropriate way of contributing for everyone will be found.
**Conclusions**: Together—health care professionals, experience experts, and researchers—we have embarked on a journey, with the improvement of patient-centred burn care as the ultimate goal. Issues that we need to address are time investment and compensation of the experience experts, and planning and content of training activities.

*O.106*

**Identifying the Best Treatment Strategies to Improve Burn Care by the Development of a Value-Based Healthcare Framework**

**Inge Spronk ^1,2,3^, Fiona Wood ^4,5^, Mark Fear ^5,6^ and Dale Edgar ^4,5,6,7^**
  ^1^  Dutch Burns Foundation
  ^2^  Association of Dutch Burn Centres
  ^3^  Erasmus MC, Department of Public Health
  ^4^  State Adult Burn Unit, Fiona Stanley Hospital
  ^5^  Fiona Wood Foundation
  ^6^  Burn Injury Research Unit, University of Western Australia
  ^7^  Burn Injury Research Node, The University of Notre Dame

**Objectives**: Surviving a burn has a tremendous impact on a patient’s life. Burn care is complex, requiring specialized multidisciplinary care. Globally, a main issue is the large number of treatment strategies for which no gold standard nor consensus exists. Furthermore, there is very limited knowledge on the outcomes of different strategies. The value-based healthcare (VBHC) approach is gathering momentum around the world. VBHC embeds constant learning from each patient by analysing provided care and associated patient-centred outcomes to quantify the outcomes of different strategies. The best treatment strategies are identified and implemented to improve care. The implemented strategies are monitored and analysed again, thereby continuously improving care. Outcomes are also used to inform patients and provide them with the opportunity to discuss with their caregiver what treatment best meets their values. However, no framework is available to systematically implement VBHC in burn care. Therefore, we develop a systematic and sustainable VBHC framework for burns.
**Methods**: Using the West Australia Burns Clinical Data Registry (WA-BDCR) that consists of both clinical and patient-reported outcome data, a systematic and sustainable VBHC framework for burns will be developed. The VBHC framework will be established using a standardized methodology with four steps (Figure 1). The healthcare provided is monitored by quality indicators, (patient-centred) outcomes and capture of costs (step 1). These data are analysed and evaluated (step 2). Interpreting the findings of step 2, the VBHC treatment strategies are identified (step 3) to inform patients and healthcare providers on outcomes of different strategies, and how to achieve the best outcomes. Lastly, the identified best treatment strategies are implemented to improve burn care (step 4) through the publication and marketing of results.
**Results**: In the framework, analysis models will be developed and programmed into predictive algorithms. The first analysis models will be presented at the conference. This framework monitors, analyses and evaluates care and its (patient-centred) outcomes to identify the best treatment strategies in a cyclical manner (Figure 1). Effects of implemented strategies, future care and research questions can be studied with this framework by following the four steps in a cyclical manner. This VBHC framework will be implemented after its development.
**Conclusions**: Developing and applying the VBHC framework identifies the best treatment strategies to improve burn care and patient-relevant outcomes. It supports patient-centric, continuous quality improvement with an added focus on cost containment. Where good evidence exists, strategies will be promoted to improve burn care and outcomes worldwide.

*O.107*

**Burns Care Training for Low-Income Countries: A Literature Review and Critical Appraisal**

**Tiffanie-Marie Borg ^1,2^, Anand Krishna ^1^ and Ali Ghanem ^2^**
  ^1^  National Health Service
  ^2^  Academic Plastic and Reconstructive Surgery Group, Barts and the London University of Medicine

**Objectives**: Low- and middle-income countries account for over 90% of burns worldwide. Though mission trips, public health interventions and educational strategies have been introduced in recent years, there remains a disparity in treatment provided between high- and low-income countries. This analysis aims to review the available literature pertaining to strategies for training in burns management, with a focus on those applicable to low-income countries.
**Methodology**: Mesh terms including “burns”, “burns care”, “burns management”, “training”, “teaching” and “education” were inputted into Medline and EMBase. Studies were included on the basis that they include an educational intervention to train doctors to provide surgical burns care in low-income countries. The included literature was analysed using scoring tools, then a critical appraisal was performed.
**Results**: Fourteen studies were included in this analysis. These describe e-learning (*n* = 1), video-based teaching (*n* = 1), lecture-based teaching (*n* = 1), simulation training (*n* = 8) and hospital-based training achieved through collaborative efforts between high and low-income countries such as mission trips and fellowship programmes (*n* = 3). The strategies described have been summarised and presented.
**Conclusions**: Burns care training should be accessible at a global scale and involve training methods including simulation, courses and fellowship programmes that are affordable and accessible to surgeons in low-income countries.

*O.108*

**Optimising Highly Specialised Burn Care, Education and Research in The Netherlands**

**Inge Spronk ^1,2,3^, Tsjitske Haanstra ^3^, Carine Van Schie ^3^, Margriet E. van Baar ^1,2^, Anouk Pijpe ^4^, Annebeth de Vries ^4,5,6^, Marianne K. Nieuwenhuis ^7,8,9^, Sonja M. H. J. Scholten-Jaegers ^7^, Esther Middelkoop ^4,10,11^, Eelke Bosma ^7,12^, Cees H. van der Vlies ^1,13^ and Paul P. M. van Zuijlen ^4,5,6,10,11^**
  ^1^  Association of Dutch Burn Centres, Maasstad Hospital
  ^2^  Department of Public Health, Erasmus MC, University Medical Center Rotterdam
  ^3^  Dutch Burns Foundation
  ^4^  Burn Centre, Red Cross Hospital; and Association of Dutch Burn Centres
  ^5^  Paediatric Surgical Centre, Emma Children’s Hospital, Amsterdam UMC, location AMC
  ^6^  Department of Surgery, Red Cross Hospital
  ^7^  Association of Dutch Burn Centers, Burn Centre Martini Hospital
  ^8^  Hanze University of Applied Sciences Groningen, Research group Healthy Ageing, Allied Health Care and Nursing
  ^9^  Department of Human Movement Sciences, University Medical Center Groningen, University of Groningen
  ^10^  Amsterdam UMC location Vrije Universiteit Amsterdam, Plastic, Reconstructive and Hand Surgery
  ^11^  Amsterdam Movement Sciences, Tissue Function and Regeneration
  ^12^  Department of Surgery, Martini Ziekenhuis
  ^13^  Trauma Research Unit Department of Surgery, University Medical Center Rotterdam
  ^14^  Department of Plastic and Reconstructive Surgery, Red Cross Hospital

**Objectives**: The mission of the three Dutch Burn Centres is to achieve the best possible quality of life, autonomy and reintegration into daily life for every burn patient. We aim to reach this through optimal person-centred care matching a patient’s preferences and goals through providing the right care, at the right time, in the right place for the right price for every patient. To achieve this, we have recently started the Highly Specialised Burn Care, Education & Research Programme. Within this programme, we are developing and implementing an approach in which the patient is an equal partner and includes the principles of value-based health care (VBHC) and shared decision-making (SDM). The abstract describes the development of a sustainable systematic VBHC framework that shapes the evaluation of our specialised care and aligns with our current and future care and research priorities.
**Methods**: Our VBHC framework builds on our existing data registries on patient and injury characteristics and clinical processes (Dutch Burn Repository R3) and patient-reported outcomes (Burn Centre Outcomes Registry, The Netherlands), with outcomes such as length of hospital stay, quality of life and scar quality assessment over time. With this VBHC framework we will learn from each and every patient by monitoring, analysing and evaluating provided care and its (patient-relevant) outcomes to identify best treatment strategies in a cyclical manner. Quality improvement teams will enhance uptake of this approach in our care. Outcomes will be used to develop decision aids and a blended aftercare programme to inform patients and support them and burn care providers in SDM on burn care and treatment decisions.
**Results**: Three pivotal areas of burn care were selected to study using the same approach based on six work packages (WP): the timing of surgery of intermediate depth burns, the added value of tissue-engineered skin constructs, and the role of self-management in aftercare. These areas have been studied; however, there is no evidence-informed consensus on best practice. As such, this project has an unprecedented opportunity to inform standards of burn care. By choosing the VBHC framework to address these knowledge and practice gaps, we are prioritizing the patient’s perspective and values.
**Conclusions**: This project will address urgent questions in burn care, result in improved evidence to inform best practice and strengthen a sustainable VBHC and SDM approach. Most importantly, this approach will support ongoing innovation and improvements as we strive to achieve our mission.

*O.109*

**Reconstruction of the Subcutis in Adherent Burn Scars Using Autologous Fat Grafting through Washing as Processing Technique: A One Year Prospective Follow-Up Study**

**Danielle Rijpma ^1,2^, Mariëlle Jaspers ^2^, Anouk Pijpe ^1,2,3,4^, Matthea Stoop ^1,2^, Antoine van Trier ^1,5^ and Paul van Zuijlen ^1,2,4,5,6^**
  ^1^  Burn Centre, Red Cross Hospital
  ^2^  Amsterdam UMC location Vrije Universiteit Amsterdam, Plastic, Reconstructive and Hand Surgery
  ^3^  Association of Dutch Burn Centres
  ^4^  Amsterdam Movement Sciences Institute, Amsterdam UMC
  ^5^  Department of Plastic, Reconstructive and Hand Surgery, Red Cross Hospital
  ^6^  Department of Pediatric Surgery, Amsterdam UMC, Location AMC

**Objectives**: In burns or other severe injuries, the subcutis can be damaged, whilst it is indispensable as a sliding layer between the scar and underlying structures. It is hypothesized that a new subcutical layer can be reconstructed with adhesiolysis and autologous fat grafting (AFG). Our previous study showed a skin elasticity improvement of more than 20% after single treatment AFG by the Coleman technique. Subsequently, we were interested in AFG by a different fat-processing technique. Therefore, the aim of this study was to evaluate the effectiveness of single-treatment AFG in adherent scars, processing the fat through a washing technique.
**Methods**: In this prospective observational intra-patient cohort study, validated measurement tools were used to assess the change in pliability 12 months post AFG, compared with pre AFG (baseline). Pliability is strongly related to the function of the subcutis and was measured using the Cutometer®. Scar quality was evaluated by the Patient and Observer Scar Assessment Scale (POSAS) and DSM II ColorMeter (erythema and melanin). The POSAS items are scored on a scale ranging from 1 (‘like normal skin’) to 10 (‘worst scar imaginable’). Measurements were performed pre-operatively and the same locations were evaluated after 3 and 12 months. Harvested fat was processed through washing in the Puregraft System (www.puregraft.com).
**Results**: In total, 47 consecutive patients were included, and 35 patients completed follow-up at 12 months. The median scar age was 13 years (IQR 7–27). The pliability parameters as measured by the Cutometer remained unaltered over 12 months follow-up (*p* > 0.05). POSAS scores for the item ‘pliability’ ameliorated significantly: patients scores decreased from 7.3 to 5.6 (*p* < 0.001) and by observers from 5.3 to 4.8 (*p* = 0.111). In addition, the patient scores for the item ‘pain’ decreased from 3.9 to 2.4 (*p* = 0.001). Additionally, the patients mean POSAS score of all items decreased over 12 months from 4.8 to 3.8 (*p* < 0.001), indicating an improved scar quality. Erythema and melanin differences between the scar and normal skin became smaller at 12 months follow-up (*p* = 0.51 and *p* = 0.18, respectively).
**Conclusions**: AFG through a fat washing technique is a minimally invasive procedure that reconstructs a small subcutical layer. No improvement regarding pliability was measured by the Cutometer®. However, subjective scar quality and pliability scores showed significant improvement after AFG.

*O.110*

**Comparison in the Treatment of Burn Scars Using Lipofilling and Ablative and Non-Ablative Fractional Lasers**

**Giuseppe Spaltro, Marco Schirosi, Tiziana Pagliarini, Simone Moroni, Andrea De Bellis, Carmela La Greca and Paolo Palombo^1^**
  Uoc A.S. Centro Ustioni E Chirurgia Plastica Ospedale S. Eugenio Roma

**Objectives**: The purpose of this work was to evaluate the results in the treatment of burn scars through the combined use of lipofilling with ablative and non-ablative fractional laser, comparing the methods and observing the best outcome not only from an aesthetic-functional point of view but also histological.
**Methods**: We grouped the study patients with homogeneous burn scars, dividing the areas to be treated into three areas for each patient: only with lipofilling, lipofilling and ablative fractional lasers, lipofilling and non-ablative fractional lasers. Subsequently, the areas were treated with another two cycles of ablative and non-ablative fractional lasers before the final evaluations also by means of biopsies and histological examination.
**Results**: The combined methods of lipofilling and ablative and non-ablative fractional lasers led to a reduction in the improvement time of scars compared with laser treatment alone; however, from a histological point of view, in our experience, the best result was obtained through lipofilling and non-ablative fractional lasers.
**Conclusions**: In view of the treatment of retracting and disabling burn scars, the combined action of lipofilling with laser technologies drastically affects recovery times with better aesthetic and functional outcomes.

*O.111*

**Effects of Intradermal Autologous Platelet Rich Plasma Injection on Graft and Donor Site on Healing and Scar for mation**

**Elif Asfuroglu, Burhan Saban and Zeynel Asfuroglu**
  İskenderun State Hospital

**Objectives**: The application of (platelet-rich plasma) PRP has been shown to improve healing in various wounds; however, there are limited data on its effect in burn wounds. We aimed to investigate the effect of PRP application on donor site and skin graft healing and scar for mation in patients with full-thickness burns and treated with split-thickness skin grafting.
**Methods**: Sixty burn patients who were treated with meshed split-thickness skin grafting were included to the study. The total burned body surface area was 20% or lower. Patients were appointed in each group as comparable burn sites and were assigned into two groups. In group I (prp +graft group) (*n* = 30), PRP was applied to patients on the donor site after graft harvesting and on the burn site before grafting. Patients were called for the second and third PRP applications on postoperative days 15, 30 and 60. In group II (control group, *n* = 30), patients were grafted without PRP application. All of the patients were called for control monthly for a year. They were asked to answer the Patient and Observe Scar Assessment Scala (POSAS). In each control, a Vancouver Scar Scala (VSS) was assessed.
**Results**: There was no statistically significant difference between the graft and donor site epithelization rate, infection rate or graft lysis rate between the two groups. during the assessment of the graft site, there was no significant difference between the VSS scores on day 30; however, the pain score in the POSAS was significantly higher in the control group. On day 60, the VSS pigmentation score was significantly higher in the control group (21/5). On day 90, the itching score was significantly higher in the control group (20/7). Six months after the surgery, the mean vascularity (2.2/1), pigmentation (1.8/1), pliability (3.3/2) and height (2.5/1.5) scores were significantly higher in the VSS of the control group. The POSAS assessment showed a significantly higher pain and itching score in the control group during all the controls for a year. Donor site healing time, infection rate was similar between groups. On day 60, the mean itching and pigmentation scores of the donor site were significantly higher in the control group.
**Conclusions**: Application of PRP to the graft and donor sites before and after surgery significantly decreases the scar pigmentation, pain and itching of the surgical sites and provides higher comfort for the patient.

*O.112*

**Reconstruction of a Burn Sequel of the Hand with Long and Narrow Free Radial Forearm Flap**

**Burak Ozkan ^1^, Cagri A. Uysal ^1^, Emin Turk ^2^ and Mehmet Haberal**
  ^1^  Baskent University Faculty of Medicine, Department of Plastic, Reconstructive and Aesthetic Surgery
  ^2^  Baskent University Faculty of Medicine, Department of General Surgery and Burn Center

**Introduction**: Although flap planning is easier to cover broad defects, the reconstruction of long and narrow defects is challenging. Therefore, the general strategy is to cover the defect with a free flap and de-bulk the flap in a later stage. This study presents a reconstruction of a burn sequel of hand with a long and narrow free radial for earm flap (FRFF) in one-stage reconstruction.
**Case Report**: A 72-year-old otherwise healthy man was admitted to the Burn Unit with a fire burn injury in his right hand. His right hand was severely burned while he was trying to extinguish a fire that started from the curtains of his house. In his physical examination, he had second- to third-degree burns in the dorsum of the right hand. The patient was hospitalized and started daily wound care with silver sulfadiazine. Eschar was debrided in the first week of hospitalization. after debridement, tendons of extensor pollicis longus, extensor indicis proprius, and the radial side of the dorsum of the hand were exposed. A free serratus anterior fascia flap and skin grafting were performed to cover wide skin defects. As a complication, partial necrosis of the flap developed. The distal phalanx of the index finger, second metacarpophalangeal joint, and second metacarpal corpus were exposed. An FRFF with 3 cm in width and 20 cm in length was elevated. The donor site was closed primarily.
**Conclusions**: The free radial for earm flap can be used for long and narrow skin defects with tendon or bone exposure after deep dermal burns.

*O.113*

**Microsurgical Free Tissue Transfer for Deep Scalp Burns: Our Experience in Seven Patients in a Major Burn Unit**

**Alex Arteaga Pérez, Gorka Reyes Aguilera, Jordi Serracanta Domenech and Joan Pere Barret Nerin**
  Vall D’Hebrón Hospital

**Objectives**: Scalp reconstruction in patients with deep scalp burns poses a surgical challenge. Different reconstructive techniques have been used historically, including skin grafts and local flaps. Nevertheless, when dealing with full-thickness burns with a wide extension of the defect, exposure of the cranial vault with injured periosteum or damaged adjacent tissue makes the use of free flaps advisable to achieve an optimal reconstruction. The aim of this study is to expose our experience in microvascular free tissue transfer to treat deep scalp burns
**Methods**: From April 2011 to April 2022, seven patients underwent microsurgical procedures for scalp burn reconstruction in the Burn Unit of the Hospital Vall d’Hebron.
**Results**: Data from seven patients (six men and one woman) were collected. The mean age was 55 years with a range from 28 to 84 years. The etiology of burns was three from an electrical cause, two by flame, one by deflagration and one unknown. Nine free flaps were performed: four Latissimus Dorsi (LD), one quimeric flap LD + Anterior serratus, two Rectus femoris and two Anterolateral thighs (ALT). Receptor vessels were the superficial temporal artery and vein, external carotid artery, internal jugular vein, thyrolinguofacial trunk and retroauricular vein. The success rate of free flaps was 78%, achieving an optimal and definitive result in six out of seven patients.
**Conclusions**: LD and ALT free flaps are excellent options for scalp reconstruction after deep burn injuries due to their reliability and adaptability to cranial anatomy. Its constant pedicles with adequate length and caliber allow anastomoses to different cranio-cervical receptor vessels. In our experience, skin grafting or local flaps using adjacent tissue to burned areas could increase the risk of complications and the need for multiple reinterventions. Free flaps have been proven to be a safe option with good functional and aesthetic results in most patients.

*O.114*

**Bilateral Upper and Lower Lid Ectropions Caused by Domestic Off-Label Use of a Liquid Unblocker—Case Report**

**Sophia Papadopoulou, Andrew Joycey, Argyro Pipinia, Zoi Tzimorota and Eleni Karagergou**
  Plastic Surgery Department & Burns Unit, “G. Papanikolaou” Hospital

**Objectives**: Liquid drain unblockers, although meant to be used by professionals with protective equipment, are sometimes used in the household without any precautions. This could lead to severe chemical burns, as in the case we present. The purpose of this study is to stress the need for preventive measures regarding the domestic use of chemicals and for close observation and timely surgical intervention in such cases to prevent and limit disfigurement.
**Methods**: A 45-year-old woman attempted to use an unblocker at home suggested by a neighbor as very “effective”. However, the bottle fell and some of the liquid (sulfuric acid 40%, as it proved to be) was spilled on her face. She was initially referred to an ophthalmology department because of the obvious ocular involvement and the cutaneous component was underestimated. On the third day post-burn. She was examined as an outpatient in our clinic and because of the soft consistency and mostly linear pattern of the burn, asked to revisit in a week. She only reappeared in our clinic almost 2 months post-burn with severe ectropions of all four eyelids, putting her at risk for corneal abrasion, desiccation and damage of the already injured left eye as well as the right eye. She underwent four operations in 6 months and a fifth 14 months after the accident, with release of the scarred eyelids and full-thickness skin grafts, Z-plasties and V-Y plasties and release of the scarred upper lip.
**Results:** after five operations and sessions of triamcinolone acetonide intralesional injection, the patient has satisfactory eyelid position and function with adequate closure and scar maturation. However, there is still space for improvement, especially with surgical refinements and methods such as laser treatments, nanofat techniques and needling.
**Conclusions**: Domestic unguarded use of strong industrial chemicals is a dangerous procedure, and public education for prevention is urgently needed. On the other hand, it is mandatory to follow-up very closely, especially with chemical burn patients, and prevent severe sequelae, especially in the delicate and contraction-prone periocular and perioral area. Reconstruction in these cases is a complex task. Sometimes, several surgeries are needed to restore acceptable post-trauma function and appearance, a procedure that will follow the patients throughout their life.

*O.115*

**The Indications and Outcomes of Synthetic and Biological Skin Substitutes in Burn Patients**

**Tristan Urselmann ^1^, Robin Verwilligen ^1^, Anouk Pijpe ^1,2,3,4^ and Esther Middelkoop ^1,2,3,4^**
  ^1^  Burn Centre, Red Cross Hospital
  ^2^  Association of Dutch Burn Centers
  ^3^  Amsterdam UMC location, Vrije Universiteit Amsterdam, Department of Plastic Reconstructive and Hand Surgery
  ^4^  Amsterdam Movement Sciences (AMS) Institute, Amsterdam UMC

**Objectives**: Dermal substitutes are proposed as a promising treatment of deep partial- and full-thickness wounds in patients with burns as it could improve functional outcomes, including scar quality after healing. There are two major types of skin substitutes: fully synthetic and biological skin substitutes.
In patients with partial-thickness wounds, skin substitutes are mainly used as biological dressings. To date, the relevance of different skin substitutes as treatment for burn injuries are mainly studied using animal models. Human trials often lack a suitable control, as mostly, only split-thickness skin grafts (STSG) are used instead of comparing different skin substitutes. In this study, the indications and outcomes of different types of skin substitutes in patients with thermal burn wounds are investigated by conducting a narrative review with literature research.
**Methods**: A literature search was performed on Cochrane library and PubMed, including articles published after 2014. All articles were screened on title and abstract after which it was included or excluded, based on specified criteria. Reference tracking performed in Google Scholar or PubMed allowed to find additional articles that could be included. for the included trials, the MINORS or JBI critical appraisal were conducted. The study design was determined through the use of the SIGN algorithm.
**Results**: In total, 49 articles were suitable for inclusion, of which 11 were randomized clinical trials (RCTs). The indications assessed were (deep) partial- and full-thickness burns, donor site injuries and reconstructive surgery. The treatment outcomes included graft take, scar quality, length of hospital stay, pain, infection, and wound contracture. Biological skin substitutes were mostly used in partial-thickness burns as biological dressings or in full-thickness burns, whereas synthetic skin substitutes seem less suitable for this purpose due to the elevated risk of a for eign body response.
**Conclusions**: From the narrative literature study, only general indications and outcomes can be given which have to be substantiated by newly performed trials. Biological skin substitutes seem the best option for treatment of partial or full-thickness burns. However, comparative studies were scarce and follow-up period was often too short to make definitive conclusions about the effect on scar quality. To gain knowledge about more specific indications and outcomes of the different skin substitutes, more RCTs with direct comparison of substitutes need to be performed using well-defined objective and patient-reported outcome parameters.

*O.116*

**Challenges in the Application of Dermal Substitutes: A Qualitative Study**

**Iris Sussenbach ^1^, Anouk Pijpe ^1,2,3,4^, Robin Verwilligen ^1^, Matthea Stoop ^1^, Paul van Zuijlen ^1,3,4,5^, Kim Gardien ^1^, Annebeth Meij-de Vries ^1,5,6^, Pieter Joosse ^1,6^, Annabel Snoeks ^1^, Toine van Trier ^1^ and Esther Middelkoop ^1,2,3,4^**
  ^1^  Burn Centre, Red Cross Hospital
  ^2^  Association of Dutch Burn Center
  ^3^  Amsterdam UMC location, Vrije Universiteit Amsterdam, Department of Plastic Reconstructive and Hand Surgery
  ^4^  Amsterdam Movement Sciences (AMS) Institute
  ^5^  Amsterdam UMC Location, University of Amsterdam, Paediatric Surgical Centre, Emma Children’s Hospital
  ^6^  Department of Surgery, Noordwest Ziekenhuisgroep

**Objectives**: Deep partial- and full-thickness wounds in patients with burns represent a challenge regarding the coverage of the wound with autologous skin possibly affecting the functional and cosmetic outcome. for many years, studies have shown that dermal substitutes could improve these outcomes, including the quality of the scar. On the other hand, the use of dermal substitutes seems promising, and the use of dermal substitutes have not been internationally adopted as the standard of care. In this study, the challenges in the application of dermal substitutes physicians face during this treatment are examined. we aim to gain more insight in what is needed to facilitate the implementation of dermal substitutes for the treatment of burn injuries.
**Methods**: Data were collected using semi-structured interviews and one focus group discussion with physicians (plastic surgeons, general surgeons, burn physician) who regularly apply dermal substitutes (*n* = 5–6). The interviews and focus group discussion were coded inductively using a thematic analysis. Themes covered in the interview included the physicians view on dermal substitutes; indications, contra-indications; disadvantages and advantage; facilitators and barriers. Facilitators and barriers were perceived on a patient, institutional and provider level.
**Results**: Thematic analysis revealed four main themes on the current application of dermal substitutes by physicians: (1) when to apply dermal substitutes related to perceived indications and contra-indications, (2) choosing a dermal substitute considering its perceived advantages and disadvantages, (3) facilitators or barriers for the application of a dermal substitute influencing the consideration of the physicians and (4) challenges. The first three themes could be captured in a treatment algorithm. The fourth theme challenges revealed that logistics, gaps in evidence, and varying knowledge and experience are the main challenges in the application of dermal substitutes by physicians.
**Conclusions**: We identified several challenges in the application of dermal substitutes. These challenges need to be addressed in order to optimize the use of dermal substitutes. Therefore, it is recommended to investigate the gaps in evidence on a larger scale, provide education and engagement of the treatment team and organize central management of logistics. Moreover, combining international experience and research is essential for improving burn care and outcomes using dermal substitutes.

*O.117*

**A 25-Year Review of the Helsinki Skin Bank**

**Jyrki Vuola, Kaarlo Antila and Andrew Lindford**
  Helsinki Burn Centre, Helsinki University Hospital and University of Helsinki

**Objectives**: The Helsinki Skin Bank was established in 1995 in the Helsinki Burn Centre, which is the national centre for all severe burns in Finland (population 5 million). The skin allografts are harvested from multi-organ donors from the Greater Helsinki area (1.52 million people) and preserved with glycerol according to the method originally developed by the Euro Skin Bank in The Netherlands in the early 1980s. This review focuses on changes in practice during the development and evolution of the Skin Bank and how legislation has affected it.
**Methods**: The procurement and processing of the skin is organized by the Plastic Surgery theatre nurses which is a unique approach in Europe. All files of the Helsinki Skin Bank were reviewed to identify allograft donors and recipients between 13 June 1995 and 31 December 2020. Data collected from the donors included: number of donors per year, donor age, harvested skin area and microbiological findings. From the recipient patients we identified: number of recipients, recipient age, number of operations, the number of allografts (in surface area) used to treat a recipient and the number of donors per recipient.
**Results**: The procurement has evolved to become very professional, and currently, three theatre nurses are assigned according to an on-call list. Since its inception, the costs have increased substantially, at least partially due to the changed EU directives that consider the skin bank to belong to the legislation of tissue banking. Skin was harvested from a total of 250 organ donors in the operating room setting. In total, 180 patients received allografts. The number of donors per year varied from 5 to 32 donors, although since 2012 the number of donors per year has not fallen below 20. The mean age of donors varied yearly from 49.7 to 61.6 years. The age has steadily increased during the study period. The area of harvested skin per year varied from 11 632 cm2 to 179 270 cm2. The amount of skin harvested per donor ranged from 1385 cm^2^ to 13,923 cm^2^ with a mean of 5325 cm^2^.
**Conclusions**: Running a small skin bank for only one burn centre is possible and cost-effective. The activity must be professional, and sufficient resources need to be allocated. Continuous education results in the harvesting of larger amounts of good-quality skin.

*O.118*

**Establishment of an Amnion Bank—A Sustainable Dressing**

**Jennifer Berg Drejoee, Rikke Holmgaard and Christian Lyngsaa Lang**
  Rigshospitalet, University Hospital Copenhagen

**Objectives**: With the progress made in the treatment and survival of extensive burns, there is a compelling need for comprehensive dressings, especially while we “wait” for donor sites to heal. The industry provides a wide variety of dressings, both xenografts and synthetic materials. In our department, we receive about 300 burn patients annually, but only 20 exhibit major burns (over 20% TBSA). This calls for a flexibility in capacity and dressings. Some months, we have major burns at the OR twice weekly and multiple dressing changes, whereas in other months it is primarily simple skin grafts. This calls for a flexible setting and the availability of different types of dressing. Most of the dressings commercially available are expensive and have limited durability. We need a cheap well-documented dressing available when required, and one that is easily stored. Amnion is a well-documented biological dressing, known for centuries, safe and applicable. In our hospital with a big obstetric department and weekly planned cesarians on healthy women, the harvest is easy and flexible. AdditionallyMoreover, we have a well-run and professional tissue bank, experienced in the storage of tendons and bone.
**Methods**: With help from elaborate documentation and guidance from Shriners Children’s hospital in Galveston, Texas, we prepared a plan for donor selection, harvest, preparation and the storage of amnion. In close collaboration with plastic surgeons, obstetricians and the Tissue Bank manager we prepared guidelines and applied for different approvals. Numerous SOP’s (Standard Operating Procedure) were made, and a collaboration agreement was signed.
**Results**: We now have a small amnion bank available for use. The challenges were mainly logistical, management is core to the success of the project. Additionally, in the beginning, contamination was a challenge, and we determined that a separate table for the dividing of the amnion was needed.
**Conclusions**: We will present the challenges of establishment of an amnion bank and the initial experience with the use of amnion. It gives us great satisfaction to revisit and reapply old, well-known methods of wound management. In the jungle of dressings and materials, amnion is a safe, applicable, cheap and sustainable solution for wound management in burns.

*O.119*

**The Tissue and Cell Factory Turin Skin Bank**

**Irene Cambieri ^1^, Daniela Alotto ^1^, Mara Fumagalli ^1^, Stefania Casarin ^1^, Johanna Del Carmen Saavea ^1^, Giorgia Calasso ^1^, Carlotta Zavatto ^1^, Stefania Minzon ^1^, Maurizio Stella ^2^ and Carlotta Castagnoli ^1^**
  ^1^  AOU Città Della Salute e Della Scienza di Torino, Banca Della Cute
  ^2^  AOU Città Della Salute e Della Scienza di Torino, SC Centro Grandi Ustioni

**Objectives**: The Tissue and Cell Factory Turin Skin Bank has been the Regional Reference Centre for the skin preservation for the last 23 years. It has been authorised by the Italian National Transplant Centre to harvest, manipulate and distribute human alloplastic skin and acellular dermal matrix (HADM), employed in various reconstructive procedures (burns, breast, pelvic and abdominal wall reconstruction) as a scaffold for autologous tissue regeneration, from multi-tissue and multi-organ donors. The Turin Skin Bank is also authorized to store autologous skin and autologous adipose tissue for reconstructive plastic and orthopaedic purposes.
**Methods**: Before distribution, tissues undergo strict quality controls, which include a microbiological and viability screening to certify their suitability for clinical use. The Bank operates based on GMP regulation in new laboratories (composed of four sterile rooms and two research labs and a quality control laboratory) opened in 2016. An articulated quality system documentation has been realized in order to codify and regulate all the operative procedures concerned in the handling and preparation of the tissue products, in the laboratories and in the training and updating of the specialised personnel.
**Results**: From 2017 to the end of 2021, 264 alloplastic donors were subjected to clean room processing for a total of 370,246 cm².
The clinical application of alloplastic skin has developed rapidly over time and has witnessed a great deal of therapeutic success. Indeed, a more than 343,044 cm² of skin and 15,077 cm² of HADM has been distributed over the National territory over the last five years, for clinical use in 452 skin allograft and 205 breast reconstructions. The skin grafts, packaged in units of product, may be sent to the Transplant Centre that has requested the skin along with a technical sheet and information as to clinical application.
**Conclusions**: The Turin Skin Bank is also active in the field of research and scientific innovations. Its laboratories carry out studies on projects involving new bio-substitutes and research on mesenchymal stem cells from stromal vascular fraction of adipose tissue.

*O.120*

**Cultured Fibroblast Gel-Based Biokol in the Combined Treatment of Deep Burns**

**Kamaliddin Salakhiddinov, Aney Alekseev, Madina Salakhiddinova**
  Andijan State Medical Institute

We have studied various possibilities of transplantation of cultured fibroblasts and wound dressings to restore the integrity of the skin.
**Purpose**: The development of an improved method for the restoration of the skin.
**Materials and Methods**: Various methods of surgical treatment were used to prepare burn wounds for autodermoplasty. The technique of surgical treatment was traditional, with subsequent transplantation of cultured fibroblasts distributed in the Biokol cell gel, Parapran wound dressing was used as additional fixation and protection.
The number of patients was 59; of these, 29 had combined autodermoplasty and transplantation of cultured fibroblasts distributed in Biocol cell gel, and 30 underwent thetraditional method of treatment using autodermoplasty. Parapran temporary wound dressing was used for protection and additional fixation of skin grafts.
The area of burns in patients of group 1 was 10–62% of the body surface. Deep burns accounted for 5–40% of the body surface. Flame burns were noted in 65.5% of cases, with boiling water in 27.6% and contact burns in 6.9%.
Group 2 consisted of patients with a total area of burns from 6% to 52% of the body surface. Deep burns covered 1.5–20% of the body surface. Flame burns were noted in 56.7% of cases, with boiling water in 33.3% of cases and combined in 10% of cases.
Treatment results were assessed on the basis of clinical, laboratory, cytological and microbiological studies of wounds.
In combined autodermoplasty with transplantation of cultured fibroblasts distributed in a cell gel, a rapid growth of a skin flap perforated in a ratio of 1:4 and filling of its cells was observed. Active epithelialization of the cells was observed 4–5 days after plastic surgery.
The average period of complete epithelialization of wounds was 8 + 1.4 days; in the traditional group, it was 14 ± 1.8 days.
**Conclusions**: The proposed method provides the implementation of the stimulating properties of Fibroblasts on the proliferation of autokeratinocytes in vitro and accelerates the epithelialization of skin autografts.
As a cell carrier, Biocol gel is a favorable medium for the transfer of fibroblasts to the wound surface. It has a number of important properties: economical in production, easy to use, creates an optimal microenvironment for wound healing; high absorption capacity; sufficient permeability for gases (oxygen, carbon dioxide) and the flow of reparative processes; eliminates drying of the bottom of the wound the effect of “wet environment”; the ability to model surfaces with complex relief.

*O.121*

**Use of Human Amniotic Membrane (HAM) for Burn Treatment: Clinical Aspects and Outcomes in 10 Years of Experience**

**Alex Pontini ^1^, Francesco Marena ^1^, Diletta Trojan ^2^, Giulia Montagner ^2^ and Bruno Azzena ^1^**
  ^1^  Padova University Hospital
  ^2^  Treviso Tissue Bank Foundation

**Objectives**: The use of Human amniotic membrane (HAM) for burn treatment it’s well known from several years in international literature but with poor numbers. The collaboration between our Burn Unit and a Tissue Bank developed a collaboration that increase every year and we analyzed 10 years of experience with more than 500 HAM implanted.
**Methods**: HAM is the inner layer of the fetal membranes, and is now widely used in different clinical application including as reconstruction of the ocular surface, treatment of chronic ulcers, dural repair or substitution, nerve wrapping and burn treatment. Due to their intrinsic antimicrobial, antifibrotic, anti-inflammatory functions, immunomodulatory and antiangiogenic properties. HAM promotes epithelization thanks to its content of growth factors (as epidermal growth factor (EGF) and keratinocytes growth factor (KGF)) and several anti-inflammatory and reparative cytokines (as transforming growth factor beta -TGF-beta) and chemokine.
We retrospectively review the almost 10 years of experience of HAM usage in our unit largely for the treatment of burns but also for the treatment of chronic ulcers, acute wounds and nerve exposure evaluating the outcomes in terms of quality and timing of healing, different fields of application and the development of the relationship with the tissue bank in terms of research and tissue improvement.
**Result**: Since 2013, the Plastic Surgery and Burn Unit at Padua University hospital has employed 550 HAM both in urgencies and in elective surgeries in a total of 260 different patients, among which 515 were treated for burns, 38 for chronic ulcers and 7 for other applications.
The placenta is sourced from donors undergoing caesarean sections and processed shortly after retrieval. Clinical aspects and outcomes were reported in different kind of injury and patients.
**Conclusions**: In 10 years of employment of HAM for the treatment of burned patients, we developed major experience in the country, particularly on pediatric patients. The clinical outcomes we reported demonstrate its efficacy, versatility and feasibility that allow us to achieve such result to determine its use as routinary in our practice.

*O.122*

**Evaluation of Time Impact on Cryopreservation of Human Donor Skin by Means of LC-OCT**

**Carmen Orte Cano ^1,2^, Jean Pierre aye ^1^, Gilbert Verbeken ^1^, Daniel De Vos ^1^, Nicolas Delmotte ^1^, Esther O. Adeleye ^1^, Ms Lieke Convents ^1^, Laora Spinosi ^1^, Bruno Pascual ^1^, Mariano Suppa ^2^, Véronique del Marmol ^2^, Anne Pierlot ^1^, Thomas Rose ^1^ and Jean Paul Pirnay ^1^**
  ^1^  MCT Lab-Queen Astrid Military Hospital
  ^2^  Dermatology Department-Erasmus Hospital

**Objectives**: Line-field confocal optical coherence tomography (LC-OCT) is a non-invasive real-time imaging technique that allows imaging of horizontal, vertical and 3D sections of the skin with a penetration depth of up to 500 µm. It has proven good correlation with histopathology, allowing the visualization of skin structures. The objectives of the study were to characterize by means of the LC-OCT the general architecture of skins that had been cryopreserved for 6, 7 and 8 years, and to determine whether cryopreservation affects skin structure.
**Methods**: Cryopreserved (<−135 °C) skin samples were obtained from deceased human donors according to the standard procedure at the Military Hospital in Brussels. Three different samples of cryopreserved donor skin from the Military Hospital Biobank were evaluated after 6, 7 and 8 years of cryopreservation. Samples were defrosted at room temperature and thawed for 5 min in 0.9% NaCl. Slices of 1.5 × 1.5 cm were selected for imaging. The assessed qualitative variables were characteristics of the epidermis and of the dermal-epidermal junction (DEJ), and presence of blood vessels and hair follicles within the dermis. Qualitative variables were assessed on all images for an overall result. Quantitative variables were the thickness of the epidermis and size of nuclei on stratum spinosum. Three measures per slice for each quantitative variable were acquired on one good quality representative image.
**Results**: Epidermis was well observed in all samples and did not show hyperkeratosis, parakeratosis or acanthosis. Cell nuclei could be distinguished in all samples. Their shape/size were comparable to that of healthy skin (no nuclear pleomorphism, abnormal proliferation or dyscohesion). The DEJ was present in all samples and was not disrupted. Well preserved hair follicles could be seen in the 6- and 7-year samples. Blood vessels were present in the dermis for all samples and did not show alterations (flattened/compressed/dilated). Concerning quantitative measures, epidermis thickness for the three samples varied from 100 µm to 197 µm. Nuclei size measures were comparable between the three samples, with a mean size of 5 µm. Some rare big nuclei (7 µm) were observed in the 7- and 8-year-old samples
**Conclusions**: Neither qualitative nor quantitative alterations were observed, and there were no differences between the three samples (6, 7 and 8 years of cryopreservation). The overall architecture of the skin was preserved in all samples. In conclusion, the investigated cryopreservation times did not affect the skin architecture. The present evidence supports the storage of human skin at <−135 °C for up to 8 years.

*O.123*

**How the Use of Appropriate Skills Has Safeguarded the Health of Burn Patients during the COVID-19 Pandemic. Experience of Milano Burn Unit**

**Antonella Marisa Citterio and Franz W. Baruffaldi Preis**
  Niguarda Hospital

The COVID-19 pandemic, which emerged in December 2019, has affected the Health Care System. The possible transmission route is person to person, as well as direct transmission by droplet inhalation. As of today, there is no clinically proven and specific antiviral drug available for treating the SARS-coV-2 infection. The problem, from the beginning, was the identification of the infected patients or the carriers to avoid the transmission of the virus to others. The development of skills for the first acceptance, the isolation procedures and treatment of burned patients has permitted the reduction in the viral transmission.
A grey area was identified for the admission of burn patients. Nasopharingeal and oropharingeal samples and, in a symptomatic patient, radiological investigations were also performed two times. after 48 h, COVID-19- negative COVID 19 patients were admitted to the Burn Unit. Restrictive procedures for staff and visitors were in use. A swab was performed at the discharge of each patient.
In total, 14 COVID-19- positive COVID 19 patients, from February 2020 to March 2022, were hospitalized in specific wards, and 2 patients were isolated in grey areas for a while. Surgical or conservative treatment were performed, and there were no deaths in these patients. All patients admitted to the Burn Unit tested negative. Eight COVID-19-positive nurses were identified.
Appropriate skills and the adherence to the procedures led to the prevention of viral transmission in hospitalized patients.

*O.124*

**A UK Burns Unit Experience during the COVID-19 Pandemic**

**Isabella Stevens-Harris, Salma Eltoum Elamin and Nola Lloyd**
  Salisbury District Hospital

**Objectives**: Since the start of the COVID-19 pandemic, various health care settings had to be reshaped and adjusted to match the challenges presented by the pandemic. In this paper, we present our experience at a UK Burns Unit, including the service restructure, innovative utilization of the telecommunication in the Burns Outreach Service to ensure safe and adequate patient care and its potential application in remote areas of the world.
**Methods**: This paper describes the challenges faced from the COVID-19 pandemic and the subsequent adaptations that have been made in the Burns Service in a UK Burns Unit. We describe our new burns triage, assessment and management protocol whilst focusing on the implementation of telecommunication and virtual consultations. We then give an overview and compare the burns referrals reviewed and managed at our Unit over three different time periods: pre-COVID, the first wave of COVID-19 and the second wave of COVID-19 and discuss the financial benefits of this change in the service structure. Finally, we present a case of a hand burn injury, which was managed solely virtually, discussing the advantages of virtual patient management and challengespplication in the wider management of burns worldwide.
**Results**: The comparison of the burn’s management pre-COVID pandemic to those during the first wave and the second wave revealed a high percentage of patients being managed at home and in the community. Those managed in the hospital continued to represent the most vulnerable groups (extremes of ages and mental health patients). Burns injuries previously solely managed in the hospital, e.g., hand burns, were managed in the community with adequate outcomes (healing times). With the success of virtual management seen throughout the pandemic, this raises the question of how we can develop our burns service nationally and even globally to provide specialist care to patients in remote or deprived areas through utilisation of telecommunication. Through the introduction of this novel virtual service and nurse-led triage, the unit was able to continue to provide excellent patient care, reduce hospital admission rates and save the department over GBP 100,000 per annum.
**Conclusions**: This report highlights the challenges faced by a UK Burns Unit during the COVID-19 pandemic and details its effects on service provision and structure, safe patient care and reducing infection transmission. It suggests that more burns could be managed safely at home or in the community. We propose revision of our national burns management guidelines to suit evolving national and global challenges.

*O.125*

**Burn Injury during the COVID-19 Pandemic: The Greek Experience**

**Eirini Nikolaidou ^1^, Argyro Pipinia ^1^, Athena Lavrentieva ^2^, Krystallo Makarona ^1^, Despoina Kakagia ^3^, Alexana K. Tsaroucha ^4^, Eleni Kaldoudi ^5^, Glykeria Pantazi ^1^ and Sophia Papadopoulou ^1^**
  ^1^  Plastic Surgery Department and Burns Unit, “G. Papanikolaou” Hospital
  ^2^  A ICU and Burns Unit, “G. Papanikolaou” Hospital
  ^3^  Department of Plastic Surgery, Faculty of Medicine, Democritus University of Thrace
  ^4^  2nd Department of Surgery and Laboratory of Experimental Surgery and Surgical Research, Medical School Democritus University of Thrace
  ^5^  Physics Medical Imaging–Telemedicine, Faculty of Medicine, Democritus University of Thrace

Burn Injury during the COVID-19 Pandemic: The Greek Experience
**Objectives**: The aim of this study is to present burn patients’ admission and management during the coronavirus disease 2019 (COVID-19) pandemic at a Tertiary Referral Burn Center, in Greece. A patient’s management protocol was developed to prevent virus transmission.
**Methods**: A retrospective observational study was conducted at the Burn Center of G. Papanikolaou General Hospital, Thessaloniki, Greece, comparing trends in burn emergency and admissions during two time periods: the pandemic period (Period 1), from March 2020 to January 2022, and the previous, in the pre-COVID corresponding area (Period 2), from March 2018 to January 2020. The total number of admissions to the Plastic Surgery Ward, Burn Ward and Burn Intensive Care Unit (BICU), burn patients and burn injury’s characteristics and treatment was collected for adults with any type of burn injury.
**Results**: A total of 1258 admissions were analyzed during the two set time periods. for the COVID-19 period, Period 1, there was a 21% reduction in General Admissions, 30% reduction in admissions in the Burn Ward and 44% reduction in admissions at the BICU. The mean age of the admitted patients for this group was lower compared with the non-COVID-19 period (59, 7 vs. 63, 5), since the lockdown that was impaired led to domestic accidents in younger individuals. A similar total burn surface area (TBSA) was noticed for both groups (32% vs. 34,6%). Due to in-hospital restrictions for non-transmission of COVID-19, fewer procedures were performed on each patient during Period 1. Instead, enzymatic debridement was an alternative choice to surgery. The screening protocol that was developed by our clinic, ensuring the non-transmission.
**Conclusions**: This study is the first describing the experience and the gained knowledge from a large tertiary Burn Referral Center in Greece. The most important lesson learned is that burn injury remains an emergency during the pandemic crisis, reinforcing the need of appropriate Burn Center staffing and medical resources. Our screening protocol was essential in mitigating the transmission of COVID-19.

*O.126*

**How Did the COVID-19 Lockdown Affected the Number and Characteristics of Burn-Related Injuries in Catalonia (Spain)?**

**Bernat López-Masramon, Alejana Monte-Soldado, Jordi Serracanta, Danilo Rivas Nicolls, Jorge Aguilera-Sáez and Juan P. Barret**
  Hospital Universitari Vall d’Hebron

The aim of this study is to characterize the clinical and epidemiological characteristics of acute burn patients who received urgent health care or admission to our Burn Center during the mandatory confinement period in Spain for ced by the COVID-19 epidemic.
Medical records of burn patients who received urgent care and/or admission to our Burn Center during the mandatory confinement period in Spain (Period 1: from 14 March to 9 May 2020) and during the same period of the previous year (Period 2: from 14 March to 9 May 2019) were analyzed. Both groups were compared in order to find differences in the epidemiologic profiles of burned patients.
A total of 350 burns cases were analyzed. A 36% reduction in the number of emergency department visits was identified during Period 1. However, we found an increase in the rate of hospital admissions in Period 1 (20% of the burn cases) compared with Period 2 (13% of the burn cases). Overall, 76 burn-related primary admissions were analyzed: 37 patients were admitted during Period 1 and 39 patients during Period 2. No differences were found between the two periods in the proportion of patients that underwent surgical treatment: 59.5% of patients admitted during Period 1, and 61.5% of patients admitted during Period 2. A statistically significant increase was noted in the rate of paediatric (aged 0–16 years old) admissions during Period 1 (40.54%, *n* = 15) compared with Period 2 (20.5%, *n* = 8). Among paediatric patients, an increase in the rate of surgical procedures was noted in Period 1 (47% of children), compared with Period 2 (37% of children). The proportion of patients that were admitted to the Intensive Care Unit was higher among burn children admitted during Period 1 (46.7%, *n* = 7) than those admitted during Period 2 (25%, *n* = 2). Among the 37 patients admitted during the lockdown period, two positive COVID-19 patients were confirmed.
This study gives an overview of the clinical and epidemiologic profile of burned patients during the stringent lockdown in Spain for ced by the COVID-19 epidemic. Our data show a stable trend in the number of burn-related admissions and burn-related surgeries during the confinement period. A significant increase in the rate of burn children admitted and an increase in the severity of injuries in this population was noted.

*O.127*

**Influence of COVID-19 Pandemic Conditions on the Features of Pediatric Minor Burn Injuries**

**Ayse Ebru Abali ^1^, Cem Aydogan ^1^, Semra Kamilova ^2^, Nurse Nigar Turkmen ^3^, Santiago Santelis ^1^ and Mehmet Haberal ^1^**
  ^1^  Department of General Surgery, Burn and Fire Disasters Institute, Baskent University
  ^2^  Department of General Surgery, Baskent University
  ^3^  Department of General Surgery, Burn Center, Baskent University

**Objectives**: The majority of pediatric burns are minor injuries. Social life changes due to COVID-19 pandemic may have influenced their characteristics. We compared the features of pediatric burn cases which were treated as outpatients at our center before and during the COVID-19 pandemic.
**Methods**: The records of 658 patients were reviewed. The study group was evaluated in two groups: Group I: patients treated between March 2018 and February 2020 (pre-pandemic) (*n* = 350); Group II: patients treated between March 2020 and February 2022 (Pandemic) (*n* = 308). The data collected for each case were age, sex, insurance status, place of residence; burn-causes, extent of burns, site affected, time and environment where the injury occurred, time-interval between occurrence of injury and admissions to the burn-center (mean ± SE, *p* < 0.05).
**Results**: The mean age (years) was 4.32 ± 0.24 for Group I, and it was 4.32 ± 0.18 for Group II; the male:female ratio was 0.87:1 in Group I, and it was 1.1: 1 in Group II. The mean total surface area burned was 1.94 ± 0.14% (min: 0.5, max: 16) in Group I, and it was 1.98 ± 0.26% (min: 0.1, max: 16.1) in Group II (*p* > 0.05). Most of the patients were from urban areas and were covered by social security system in both groups (*p* > 0.05). Injuries occurred between 06:00 and 18:00 in 63.7% of Group I and in 62.6% of Group II (*p* > 0.05). The most common scenario was the domestic environment for both groups with a rise in rates of outdoor burns (Group II (*p* < 0.05)), and this rise was observed during the ‘new normal’ period, but not during the ‘lockdown’ (*p* < 0.05). The most common burn cause was the scalds for both groups (*p* > 0.05). Hands, trunk, head, and neck regions were less frequently involved in group II (*p* < 0.05). Rates of direct admission were similar for both, but children in Group II were more frequently brought to our burn center on the day of injury (*p* < 0.05).
**Conclusions:** The decrease in injuries to hands, trunk, head and neck, and the increase in admissions on the day of injury were remarkable during the pandemic period. These results may be clues for enhanced care-giver precautions against injuries during ‘stay home’ days. Increased frequency of outdoor burns during ‘new normal’ may indicate the negative effects of this new unfamiliar social life on caregiver watchfulness. However, increased admissions on the day of the injury may be influenced both by the changing social conditions of caregivers and the uninterrupted service of our burn center, whereas others were serving COVID-19 patients rather than burn victims.

*O.128*

**Changing in Pediatric Burn Demographic during COVID-19 Pandemic: Data from Two High-Volume Italian Pediatric Burn Centers**

**Valeria Malvasio ^1^, Maria Grazia Cortese ^1^, Patrizia Magro ^1^, Elisa Zambaiti ^1^, Giorgia Speca ^1^, Enrico Pinzauti ^2^, Giorgia Libro ^2^, Ilaria Infantino ^2^, Simone Pancani ^2^, Antonino Morabito ^2^ and Fabrizio Gennari ^1^**
  ^1^  General Pediatric Surgery
  ^2^  Department of Pediatric Surgery, Meyer Children’s Hospital

**Objectives**: The majority of burn injuries in children occurs at home. COVID-19 pandemic has increased time spent at home by children and parents. Some studies have analyzed pediatric burn epidemiology during COVID-19 pandemic, showing contradictory results. The objective of the present study is to evaluate the impact of the pandemic on the epidemiology of burns in a large Italian pediatric population, with the aim to analyze and update themes for targeted burn-prevention campaigns.
**Method**: We conducted a retrospective, observational study of all new burn children, admitted to the Emergency Department (ED) of two Italian high-volume pediatric hospitals, Regina Margherita Children’s Hospital of Turin and Meyer Children’s Hospital of Florence. We compared the COVID-19 pandemic period, from March 2020 to December 2021, with the previous two years (control period), from January 2018 to February 2020. for each patient, we collected demographic information and burn-specific variables tabulated using our electronical management system. Statistics were conducted as appropriate.
**Results**: In total, 1380 patients were included in the study period, of which 736 were males (53%). Overall, 580 patients were during the pandemic (28 ED admission/month) and 800 in the control period (30 ED/month). Pediatric burns occurred similarly, mainly at home (88–90%), with an increase in scald injuries during the pandemic (65% vs. 59%, *p* = 0.01). Interestingly, the face and trunk were both increasingly involved during the pandemic period (23% vs. 18% for head and face, *p* = 0.03; 26% vs. 20% for trunk, *p* = 0.02), whereas other injury sites were equally involved (54–56% for upper limbs, 33–35% for lower limbs, 3–4% for perineum). The age and rate of patients with more than 10% of total body surface area (TBSA) were similar. Despite surgical ward admission significantly increasing during the pandemic (19% vs. 13%, *p* = 0.003), the need for operative surgical procedures was not different (7% vs. 5%, *p* = 0.2).
**Conclusions**: Despite more children being in the home, the potential for more accidents did not translate into an overall increased rate of ED burn attendance. Scald injuries remain the most common cause of pediatric burns and the related greater involvement of burns on the face and trunk confirms the frequent dynamic of liquid spilled onto the upper body area. An improvement of the prevention campaign targeting these identified aspects may be helpful to reduce the demographic changes of the pandemic.

*O.129*

**Cuty Firephant Project: An Innovative School-Based Education Campaign to Prevent Burn Injuries in Children**

**Valeria Malvasio, Paola Curto, Samanta Marocco, Vincenzo Lo Vermi, Enzo Amelio, Dorina Caldarescu, Sonia Zavaglia, Roberta Ferro^1^, Donato Caldarella, Daniele Capece, Loredana Silivestro, Tamara Palmieri, Claudia Barberis and Daniele Bollero**
  Cute Project

**Objectives**: Burns are serious accidents at any age, but preschool and school-age children are at greater risk for burn injuries because they are curious and like to explore. Burn prevention campaigns are usually aimed at children rarely at adults. We present the Cuty Firephant project, a school-based burn education campaign, with the possibility to reach even the adults in the same project.
**Methods**: The Project started in 2016, supported by the Cute Project. The Cute Project is a non-profit organization, founded in Turin (Italy) in 2012, whose main purposes are the theoretical and practical training for health professionals of developing countries in the field of treatment of burns and the prevention of burn injuries through education campaign in schools. The Cuty Firephant project bases its education program on a mascot, a cute little elephant, who warns children of domestic dangers related to burns. Burn prevention materials include age-appropriate activities, posters, cartoons and coloring books. At the end of the session, children, while having fun, wear a surgeon’s uniform, making them more involved and responsible towards burns. Moreover, a coloring book, with dangerous situations exposed during the lesson is donated to the child, with the aim of involving parents, at home, in the prevention program.
**Results**: The Cute Project’s volunteers presented the Cuty Firephant prevention campaign in nursery schools and primary schools of 27 Turin district areas and neighboring provinces. In total, 4042 children between 5 and 7 years old have been involved from 2016. In 2016, 2017, 2018 and 2019, 660, 668, 612 and 753 children have been reached, respectively. In 2020, due to COVID-19 restrictions, 202 children have been involved. Verbal feedback from the teachers and the parents suggested that the method utilized was appreciated by children. We had also positive comments directly from the adults on the different scenes showed in the prevention campaign.
**Conclusions**: Two key factors make Cuty Firephant a successful project: the active participation of children during the lessons and, at the same time, the involvement of adults in the prevention campaign. Through an innovative and communication method, the game of wearing a surgeon’s uniform and the gift of a coloring book to share with parents, Cuty Firephant reaches its objective: to provide both children and adults with the means to develop an ever greater awareness of the dangers related to burns.

*O.130*

**A Systematic Review of the Epidemiology of Burn Injuries in People with Epilepsy**

**Janet Choi, Brother Moses Ikpeme and Mable Nakubulwa**
  Imperial College

Epilepsy is a neurological condition that causes recurrent seizures. People with epilepsy (PWE) have a greater risk of injury than the general population due to the uncontrollable movement of the body. Specifically, burn injuries are the most frequent injuries sustained in PWE during seizures. Such injuries are a significant cause of morbidity and mortality in PWE. The healthcare cost of burn injuries, including treatment and hospitalisation, poses a heavy burden on the healthcare system and the patients. Since the epidemiology of burn injuries in PWE may have changed due to increased access to care, increased awareness of seizure-related injuries, and technological advancement, it is critical to review current knowledge and identify research gaps. Therefore, this review examines the risk of burn injuries in PWE during their episodes.
**Methods**: Four databases, namely: Ovid Medline, Ovid Embase, Web of Science, and Scopus, were searched using relevant keywords and phrases to identify relevant studies. All relevant studies published in English after 2000 were imported into the Zotero reference management software for screening. Duplicates and studies retracted by the software were removed. Titles and abstracts of the remaining studies were screened, and studies not relevant to the topic were excluded. Potentially relevant studies were retrieved and imported into Covidence for a full narrative review. The Critical Appraisal Skills Programme (CASP) checklists assessed the studies’ quality.
**Results**: of the 8219 studies generated by the initial search, 11 studies met the study criteria and were utilised for the narrative synthesis. The incidence of burns in PWE was higher than in the general population, and scalds were the most reported type of burn injury. Burns sustained during a seizure can be fatal and are in the second-degree or worse. Split- or full-thickness skin grafting was the primary surgical intervention required by the patients. Overall, among PWE, women were at a higher risk of sustaining burn injuries, as were those aged 15 years or above, those in employment, or those diagnosed with generalised tonic–clonic seizures (GTCS). AdditionallyMoreover, more burn injuries were reported among PWE during the winter season.
**Conclusions**: Seizure-related burns remain among the most critical public health concerns and burden the healthcare system and patients, especially for the most at-risk groups identified above. Thus, global preventive and intervention strategies need to account for these factors to minimise the mortality and morbidity of seizure-related burns in PWE.

*O.131*

**When Pasta is Not “Perfettamente al Dente”–Paediatric Burns Associated with Cooking Pasta. A 5-Year Experience of a Paediatric Burns Centre**

**Francesca Ghini, Mehul Thakkar and Bartlomiej Bednarz**
  North Bristol NHS Trust

**Objectives**: Pasta dishes are common and universally loved around the globe. It is not uncommon for even the most able home chef to sustain a small accidental burn whilst cooking. We decided to investigate how many pasta-related accidents existed in our paediatric population in our region.
**Methods**: We searched our local burns centre database for any burns related to injuries sustained in relation to cooking pasta. We collected retrospective data covering a period of five years between 2015 and 2019. The reason behind the exclusion of 2020–2021 was because of the COVID-19 pandemic and its effect on both the presentation to our unit and the change in habits within families. The data collected included the age of the patient, mode of the injury, size, depth and location of the burn, And need for surgery/inpatient stay.
**Results**: Our burns centre covers population of about 4 million people. We identified 37 patients (22 females, 16 males) ranging from between age 6 months and 15 years and 9 months (average age was 5 years and 5 months, median 3 years)
There were 27 scald injuries and 10 contact burns. The mode of injury was mainly splash/partial spillage of pasta water (15), followed by spillage of a whole pan of cooking pasta/sauce pan full of water (14) and direct touch of the hot hob/pan (8). The majority of burns were superficial/mid-dermal in nature, with size ranging from 0.1% to16% (average of 2.05% median 1%). Two patients had deep dermal/full-thickness components (1.25% and 4%). The largest burn was 16%. In total, 5 patients required inpatient stay ranging from between 4 to 33 days with an average of 11.8 days. Overall, 1 patient required readmission for 3 days. In total, 3 patients required surgery (13%, 16%, 5% of TBSA) with a total of 9, 3 and 1 trips to theatre, respectively. The most common areas affected were the hands, for earms, and head and neck area (each representing 10 out of 66 injured areas). Areas such as the abdomen, lower limbs and fingers were also affected.
**Conclusions**: Burns associated with cooking pasta occur at any age; however, the majority of patients are below the age of 3. Although most burns were treated in an outpatient setting, the most severe ones had multiple surgeries and grafts. Considering the latter, this is a great topic of prevention education for parents by health visitors, considering that most of the patients were young. A clear education message from our data shows three main dangers: hot hob, spillage during drainage of the pasta and kids pulling saucepans from the hob.

*O.132*

**Would You Like a Pineapple with That? Pizza-Related Injuries among the Paediatric Population over 5 Years: A British Burns Centre Experience**

**Francesca Ghini, Mehul Thakkar, Florin Panduru and Bartlomiej Bednarz**
  North Bristol NHS Trust

**Objectives**: Pizza is one of the favourite foods of the Western world, especially amongst children. It is usually served out of wood-fired ovens, making it piping hot at the time of arrival at the table. Additionally, melted cheese and tomato sauce retain their heat for a considerable amount of time; therefore, there might be high incidence of pizza-related injury amongst paediatric patients.
**Methods**: We searched our local burns centre database for any burn related to pizza cooking/eating. We collected retrospective data covering a period of five years between 2015 and 2019. The reason behind the exclusion of 2020–2021 was because of the COVID-19 pandemic, which changed the amount and pattern of our referrals significantly. The data collected included the age of the patient, and the size, depth and location of a burn, as well as the need for surgery/inpatient stay.
**Results**: Our burns centre covers a population of about 4 million people. We identified 21 patients (8 females 13 males) with ages ranging from 5 months to 13 years and 8 months (average 3 years 9 months, median 2 years 3 months). All the burns were superficial and of partial thickness in nature, and were treated in an outpatient setting (no need for admission or surgery). The average size of the burn was 0.33% (min 0.01%, max 2.5% and median 0.2%). The areas affected were mainly hands, but feet and lower limbs were also affected. The majority of cases happened at home (11) or in the garden (5), with only 3 occurring at restaurants. over 71% of cases were contact burns associated with ovens (7) or cast iron/pizza stone/trays (8). The remaining 6 patients had injuries caused by touching hot pizza or a pizza ingredient falling on them. The most common areas were hands and fingers (16/23 injured areas), followed by the face (3) and feet (2).
**Conclusions**: Pizza is always associated with excitement and great memories; however, accidents can happen when excitement dampens the attention of child. Although it seems that these injuries are not common, we speculate that we only see the more severe injuries in our centre, with the majority being dealt with by parents or smaller units. We also note that these burns are small and superficial in nature, allowing for them to be treated locally and thus saving patients travel time and time off work to reach a burns centre being designed as a hub and spokes model.

*O.133*

**Burn Injuries Caused by Contact with Motorcycle’s Exhaust Pipes: A Double Risk for Children**

**Verónica Yañez, Rolando Saavea, Johanna Diaz and Orlando Flores**
  Coaniquem

**Objectives**: In recent years, non-frequent mechanisms for burn injuries have been gaining growing attention from clinicians and researchers alike. One such mechanism is the burn injuries caused by contact with the motorcycle’s exhaust pipe. In Chile, there are no epidemiological or clinical data to describe the importance of this burn mechanism. The objective of this study is to (a) describe a paediatric population affected by burn injuries due to contact with a motorcycle exhaust pipe in 2006–2009 and 2016–2019, (b) compare the epidemiological characteristics of this population between both periods and (c) describe risk factors associated with burn injuries caused by the contact with motorcycles’ exhaust pipes in children.
**Methods**: An observational, retrospective, single-centre study was designed. The clinical records of a burn reference centre for children in Chile were used to extract epidemiological data from patients that sustained burn injuries caused by contact with motorcycles exhaust pipes in two periods: 2006 to 2009, and 2016 to 2019. Demographic and clinical data were obtained and analysed using descriptive statistics. To compare the cumulative incidence between periods, the total number of admissions and the number of admissions with contact burns were used. The association between burn depth, the requirement for rehabilitation services and age were analysed using the chi-squared test, Fisher’s test, *t*-test, and one-way ANOVA tests as appropriate.
**Results**: The cumulative incidence in the period 2016–2019 was significantly lower compared with 2006–2009. Hand burns were more frequent among children under 2 years who came in contact with an exhaust pipe while the vehicle was parked. There are significant associations between burn depth and the requirement of rehabilitation, and between the anatomical area affected and the burn mechanism.
**Conclusions**: Despite improvements made in safety features on motorcycles, burns due to contact with exhaust pipes are still a significant problem in the paediatric population in Chile. The data suggest that the anatomical area affected is associated with the age of the patients. This information is key for the better design of prevention campaigns in Chile.

*O.134*

**Global Burn Prevention: Ukraine**

**Robert Dabek ^1^, Myroslava Decik ^3^ and Gennadiy Fuzaylov ^2^**
  ^1^  Ascension Saint Agnes Hospital
  ^2^  Harvard Medical School
  ^3^  National Health Service of Ukraine

**Objectives**: Burn injury accounts for a large proportion of surgically treatable disease. It is estimated that over 180,000 flame burn deaths occur annually across the globe, with roughly 95% occurring in low- or middle-income countries (LMIC). Within these countries, children account for a disproportionately high number of burn injuries. As such, the WHO has identified burn prevention as a topic of interest, with an increased need in low- and middle-income countries. Here, we describe the creation and implementation of a burn-prevention program in Ukraine.
**Methods**: We instituted a four-step burn-prevention initiative consisting of data gathering and program design, implementation, impact evaluation, program maintenance and expansion.
**Results**: The burn-prevention initiative has been adopted nationally, leading to policy change. Active education and an information campaign were used to target pediatric scald injuries and improve first aid care.
**Conclusions**: The authors have successfully implemented a targeted multi-faceted, national, burn prevention program within Ukraine. The described approach may be used as a guide and adapted to create similar prevention programs within other countries or regions.

*O.135*

**EPIBURNS: A Project to Assess the Burden of Seizure-Related Burns among Epileptic Patients**

**Antonio Bulla, Marc Illa-Boixaderas, Jorge Aguilera-Saez, Jordi Serracanta, Danilo Rivas-Nicolls and Joan-Pere Barret**
  Hospital Universitari Vall D’hebron

**Objective**: Epilepsy is a chronic disease characterized by recurrent seizures with neurobiological, psychological and social complications.
Despite mixed results, published studies consistently suggest that people with epilepsy are at greater risk of accidents and injuries than nonepileptic individuals.
The incidence of seizure-related burns as a reason for admission to a burn center has been estimated to be between 1% and 10%.
The aim of this study was to ascertain the impact of seizures on burns among patients with epilepsy. The working hypothesis was that burns that occur during seizures are more severe due to the absence of defensive mechanisms.
**Methods**: A retrospective longitudinal cohort study was conducted on all patients with epilepsy treated for burns at Vall d’Hebron Hospital from 2012 to 2020. The Burn Center database was queried to find all inpatients and outpatients with a history of epilepsy.
The data gathered comprised the date of the burn, patients’ gender and age, medical history, burn modality, characteristics, and its evolution.
**Results**: In total, 64 patients (33 women, 31 men) treated between 2012 and 2020 met the criteria of our search. The median age was 49.5 years, and the IQR 35–59 years.
The burn happened in 23 (36%) patients during a seizure and in 41 (64%) patients during the interictal phase. Burns were referred by 24 (51%) patients as being related to cooking, 7 (11%) were scalds with sanitary hot water and 24 (37%) occurred in other circumstances (among them three were work injuries). Multiple logistic regression analysis showed that after adjusting for age and gender, the burns that occurred during a seizure were more likely to be deep (requiring surgical intervention) than those occurring in the interictal phase (OR 7.3 SE 2.01, *p* = 0.00279).
Females were more likely than men to be burned during a seizure, according to simple logistic regression analysis (OR = 6.2, 95% CI 2.03–22.2, *p* = 0.002298). This association can only marginally be ascribed to women being more likely than males to be active in household tasks.
**Conclusions**: According to the findings of our study, burns that occur during an epileptic seizure are more severe, i.e., deeper, often requiring surgical intervention, than burns that occur during the interictal phase. Female patients are at higher risk of burns during a seizure. Epileptic patients should be educated to avoid the risks of burn accidents and adopt preventive measures, especially at home.

*O.136*

**Identifying Acquired Methemoglobinemia in Pediatric Burns: A 10-Year Survey**

**Iulia Nacea ^1^, Dan Mircea Enescu ^1,2^, Cristina Stoica ^1^, Bogdan Prisecaru ^1^, Simona Stoicescu ^1,2^ and Raluca Tatar ^2^**
  ^1^  Grigore Alexandrescu Clinical Emergency Hospital for Children
  ^2^  Carol Davila University of Medicine and Pharmacy Bucharest

**Objectives**: Pediatric burn cases represent an important patient subset, and any detail associated with the pathophysiology and treatment may influence the short- and long-term outcome. Acquired methemoglobinemia (MetHb) is a rare complication in medical practice, which sometimes may occur concomitantly with burn injuries. It may appear as a consequence of internal or external exposure to oxidizing agents. In the absence of timely diagnosis and treatment, it may lead to death. The aim of this study is to review the pediatric burn cases identified in our center and the evolution of this complication over the past decade.
**Methods**: We performed a retrospective study, analyzing the electronic files of all burn patients admitted to the Plastic Reconstructive Surgery and Burns Department of the “Grigore Alexandrescu” Clinical Emergency Hospital for Children, Bucharest, during the period 2012-2021. The inclusion criteria included the presence of a burn injury (ICD-10 code T2*-T3*), concomitantly with MetHb (ICD-10 code D74).
**Results**: during the study period, 27 burn patients who developed methemoglobinemia were identified. of these, 63% had a single episode of MetHb, 26% had two episodes, 7.3% of them had four episodes and 3.7% had five episodes. The age of the patients included in the study ranged from 9 months to 17 years and 10 months. The extent of the burns varied from 10 to 90% TBSA. The analysis of the cases distribution per year had shown an increase in the number of cases starting from 2019. This was due to improvements occurred in our department’s staff for the diagnostic of this complication in burn patient. The main factors responsible for the occurrence of methemoglobinemia identified in this patients’ series, used locally or systemically, are represented by: benzocaine, acetaminophen, rifampicin, silver sulfadiazine and metoclopramide. Once the methemoglobinemia is diagnosed, the specific treatment (metilen blue) has to be administered promptly. This approach leads normalization of both blood MetHb levels and respiratory parameters, with no further complications.
**Conclusions**: Burn care teams must be aware that although rare, acquired MetHb may occur in burn patients. Suspicion raised by decreasing oxygen saturation and cyanotic lips must be rapidly followed by blood workup that identifies the high levels of MetHb, and the proper treatment should be initiated. Our study highlighted the value of a trained staff in recognizing this situation and the good outcome resulting from early and targeted therapy.

*O.137*

**Full-Thickness Burn to the Fingers—Free Flap in a 2-Year-Old Child?**

**Simon Kuepper, Bernd Hartmann and Jenny E. Dornberger**
  BG Unfallkrankenhaus Berlin

**Objective**: Toddlers often use their hands to explore their surroundings. That is why the hand can be a primary site of injury. Burns to the paediatric hand are relatively common, thermal injuries being the most frequent entity. Electrical aetiology contributes a minor portion of the burn injuries in the paediatric population, but can be very destructive.
**Method**: We report the management of an acute burn injury due to low-voltage electricity (230 V) to the hand of a 2-year-old boy who touched a multiple plug. The index to his little finger showed full-thickness burns of his left hand, mainly on the middle and distal phalanx. The extensor tendons of the index and middle finger were destroyed with opened PIP Joints. Furthermore, the child showed a partial-to-full-thickness burn on the right foot of 0.5 × 0.5 cm, and a 1 × 0.5 cm full-thickness burn on the right index finger.
**Result**: Surgical intervention started on day 2 after trauma. Temporary wound closure was carried out with a polyvinyl alcohol sponge. after a second look at the debridement on day 5, (expected) findings on the middle and distal phalanx of the index lead to amputation in the PIP joint. The extensor indices tendon was preserved for tendon reconstruction of the middle finger, and the full-thickness defect of the middle phalanx of the little finger was covered with a reversed crossfinger flap. To fix the finger position, the middle and ring fingers had been syndactylized in a splint with K-wires which was for med to an external fixation. Finally, a free ALT flap (7 × 4 cm) was performed to cover the area on the middle and ring fingers and dorsum on day 9 after trauma. The anastomose site was the snuffbox, and the vessel diameter was 1mm. The total hospital stay was about 21 days, until outpatient care began. Three months after initial treatment, syndactyly correction was performed. after a short time, the child presented a nearly normal usage of his hands, playing building blocks and overall showing good function.
**Conclusions**: Treatment of paediatric hand burns are exceedingly challenging and differ from adults because of lack of compliance. Meticulous wound care, positioning, splinting and exercise-stable repair are required to safely accomplish the goals of rapid healing with minimal loss of function. Free flapping a hand of a very active 2-year-old boy was not easy; however, with the realized treatment, our patient was able to obtain an acceptable aesthetic and functional result.

*O.138*

**Use of Homologous Skin Grafts in Pediatric Burns—A Case Series**

**Giuseppe Spaltro, Tiziana Pagliarini, Marco Schirosi, Anea De Bellis, Simone Moronissa, Carmela La Greca and Paolo Palombo**
  Uoc A.S. Centro Ustioni E Chirurgia Plastica Ospedale S. Eugenio Roma

**Introduction**: Pediatric burns are a major cause of injury and long-term morbidity in the absence of adequate care, and they can lead to lifelong functional loss and disfigurement. While split-thickness skin autografts are the current standard of care for deep partial-thickness (DPTB) and full-thickness (FTB) burns, this approach is associated with considerable morbidity. for this reason, alternative skin substitutes such as cadaver grafts have gained interest.
**Materials and Methods**: We present a case series of 12 children affected by DPTB and FTB who underwent surgery with cadaver skin grafts in the period from 2017 to 2021. The average age was 5.28 years and the total body area surface (TBSA) ranged between 19% and 40%.
In total, 5 patients were treated with a single surgical procedure using tangential escharectomy followed by homologous skin grafting. Thanks to the integration of homologous skin, the dermis was reinstated, and the patients healed completely.
In total, 5 patients had a two-step surgery: tangential escharectomy followed by homologous skin grafting in the first step, and a second surgery with autologous skin grafting to treat the remaining uncovered areas.
Only 2 patients received a three-step surgery, where the initial homologous skin grafting was followed by two different surgeries with autologous skin grafting.
Patients were followed up monthly after discharge from the hospital.
Scar quality was assessed with the Vancouver Scar Scale.
**Results**: We show that treatment with homologous skin grafts is associated with an excellent rate of healing comparable to autologous split-thickness skin grafts in terms of scar quality and contracture.
No relevant scars were recorded in the areas where the autologous skin was collected.
No pathological healing processes with relevant inflammatory response have been reported.
**Discussion**: Cadaver skin grafts has become a common surgical procedure for DPTB and FTB in adults, but its use remains constrained in children by the difficulty of finding sufficiently thin grafts, and by several concerns regarding the risk of infectious disease transmission and the inflammatory process related to the histocompatibility problem who may lead inevitably to skin rejection.
**Conclusions**: While homologous skin graft is associated with an increased upfront cost, in our experience it represents a valid tool to treat not only deep partial-thickness, but also full-thickness burns in pediatric patients. Indeed, it allows us to treat patients with moderate-to-large-sized burns without increasing the morbidity related to the donor site injury, minimizing scar for mation, and avoiding further reconstructive procedures.

*O.139*

**Surgical Strategy for Extensive Pediatric Burns in Pandemic Times**

**Dan Mircea Enescu ^1,2^, Iulia Nacea^2^, Cristina Stoica ^2^, Bogdan Prisecaru ^2^, Simona Stoicescu ^1,2^ and Raluca Tatar ^1,2^**
  ^1^  Carol Davila University of Medicine and Pharmacy Bucharest
  ^2^  Grigore Alexandrescu Clinical Emergency Hospital for Children

**Objectives**: Victims of extensive burns are a special patient category that may experience high mortality in the absence of adequate fluid resuscitation and proper conservative and surgical management of the wounds. The significant advances regarding early resuscitation for mulas, infection management and wound excision and coverage possibilities have enabled the survival for patients with massive burns that would once have been fatal. The burn teams have to adapt constantly to the new technologies and surgical techniques, and also to the societal challenges of the COVID-19 pandemic, with the aim of maintaining and improving burn care protocols and survival.
**Methods**: Patients who presented extensive burns admitted in the Plastic Reconstructive Surgery and Burns Department of the “Grigore Alexandrescu” Clinical Emergency Hospital for Children, Bucharest, over the past 2 years were analyzed. All cases aged 0–18 years old, with a diagnosis of burn injury, with at least 30% TBSA, admitted between February 2020 and February 2022, were included. The therapeutic protocol included individualized surgical, rehabilitation and intensive care approaches. Early surgery was performed for deep burns and optimal conditions were provided for the spontaneous epithelialization for partial-thickness burns. The rehabilitation program was initiated from the first days.
**Results**: for the pandemic period, 69 patients met the inclusion criteria (admitted pediatric burn with at least 30% TBSA affected), accounting for 13.35% of all admitted cases over this time. Adequate and prompt fluid resuscitation and intensive care monitoring is fundamental for the prognostic of extensive burns, together with early initiation of surgical excision of burn wounds. Wound coverage can best be achieved with autografts, and the use of Meek micrografting technique proved to be a resourceful technique for burns reaching up to 90% TBSA, discarding the need for temporary synthetic skin substitutes or skin bank grafts. The mortality rate was 0.96%. At the same time, functional recovery for those massive burns can only be ensured with adapted and early physical therapy programs.
**Conclusions**: Although extensive burns continue to be a challenge for pediatric and adult burn centers all over the world, experienced burn teams with well-established protocols may satisfactorily deal with any emerging problems or complications. The Meek micrografting technique proved to be a convenient tool for solving complex cases without the need for skin banks or synthetic skin substitutes. Finally, the complex team approach is the key feature that may ensure survival, functioning and social reintegration of children with extensive burns.

*O.140*

**A New Technique in the Treatment of Full-Thickness Scalp and Skull Burns in Children: Combination of for ming Multiple Holes with Burr Hole and Vacuum-Assisted Closure (VAC)**

**Sabri Demir ^1^, Suleyman Arif Bostanci ^1^, Ahmet Erturk ^1^, Fahri Akkaya ^1^, Can Ihsan Oztorun ^2^, Elif Emel Erten ^1^, Dogus Guney ^2^, Vildan Selin Cayhan ^1^, Mujdem Nur Azili ^2^ and Emrah Senel ^2^**
  ^1^  Department of Pediatric Surgery, Pediatric Burn Center, Ankara City Hospital
  ^2^  Department of Pediatric Surgery, School of Medicine, Ankara Yildirim Beyazit University

**Objectives**: Treatment of full-thickness burn injuries of the scalp with the burn of underlying bone is a challenging therapeutic problem. We aimed to share our experience with five children with full-thickness burns on their heads, whose firstly multiple holes were for med on their skulls and closed with VAC, and the intermittent dressings were applied until covered with granulation tissue and then covered with an autograft split-thickness graft.
**Methods**: Five children with full-thickness scalp burns were included in the study. Multiple holes were for med at the outer table of the skull with a Burr hole device until bleeding on the base was seen. The holes were drilled down to the Diploe range. At the same time, necrotic bone tissues were removed by abrasion with bone rasps. Then, the wound was covered with a paraffin gauze dressing and closed with VAC. This wound care process was repeated twice a week until the entire wound surface was covered with granulation tissue. When the wound was covered by granulation, it was grafted with split-thickness autografts (Figure 1A–H).
**Results**: of the cases (*n* = 5), four (80%) were male and one (20%) was female. The mean age of the patients was 1.24 years (min:12 days, Max: 3.16 years). The cause of burns in all patients was flame, and the mean total burned surface area was 26.4%. While two patients were admitted to our pediatric burn center on the first day post burn, the other three patients were referred to us when they did not heal in other centers. The mean length of stay of the patients in these centers was 53.7 days. The mean wound covering with granulation after the holes for ming was 45 days. Then, the wounds were grafted with a split-thickness autograft. No patients were lost. No graft complications were observed.
**Conclusions**: In the management of full-thickness scalp burns, the bones are abraded, or holes are for med in order to create granulation. The wounds covered by granulation are covered with rotational flaps or split-thickness autografts. In our case series, for the first time in children, granulation was constituted by combining for ming multiple holes and VAC application in the management of deep scalp and skull burns. VAC both accelerates the development of granulation tissue and prevents the development of infection. Therefore, we recommend a combination of for ming multiple holes with Burr hole and VAC technique in the management of full-thickness scalp burns.

*O.141*

**Cell Manufacturing Facility at Lausanne University Hospital, GMP ATMP Productions into a Public Institution: We Do It**

**Jean-François Brunet, Stéphanie Droz-Georget, Joëlle Ven, Sarah Stijve and Farshid Sadeghipour**
  Lausanne University Hospital

**Aim and objectives**: Lausanne University hospital has long offered the possibility to treat high-burn patients with cell epidermal autograft (CEA-CHUV) and has developed clinical trials with advanced therapy medical products (ATMP) in the clinical field for use as reconstructive surgery, and in the treatment of orthopedics or rare disease. Regenerative medicine is a challenging task for clinical application into public institution since the manufacturing of ATMP has to be performed under GMP conditions, and due to the limited volume that has to be produced, infrastructure and equipment have adapted to this specific activity. In answer to this challenge, the concept of cell production into dedicated isolators was studied and in collaboration with the manufacturer, this specific equipment was installed and qualified to be able to produce cell products for clinical applications authorized by our medical product agency, Swissmedic.
**Methods**: The workflows of activities such as reception of raw materials, quality control, batch of production, and the release of the final product into a public institution have to be implemented including directives such as for the purchase of material and equipment, for both personal and financial management. This implementation has also to take in account the requirement of production of therapeutic product, clinical applications, academic research and conflict of interests. The public purchase of specific equipment was also a challenge.
**Results**: Lausanne University Hospital implemented a Cell Production Center (CPC) that is now accredited by Swissmedic (authorization n°511127). Due to regulation issues, this implementation has to take in account GMP guidelines for a small unit of 14 collaborators in a context of huge public institution of more than 14,000 collaborators.
**Conclusions and relevance**: In summary, adapting a small GMP unit into a huge public institution is possible if all partners share their experiences and work together to a general win–win development of their respective quality systems.

*O.142*

**The Effect of Burn Injuries on Brain Transcriptomics and Metabolomics**

**Amira Allahham**
  Fiona Wood Foundation

Burn patients, especially children, are more prone to mental health conditions following their injury, as recently demonstrated using population-based epidemiological studies in Western Australia. The inflammatory response to a burn, coupled to a leaky blood–brain barrier may lead to immune and inflammatory changes in the brain that underlie the long-term increase in mental health hospital admissions observed. However, there are scant mechanistic data examining the effects of burns on the brain causing these mental health problems. In this study, a mouse model was used to: (1) determine the genes in the brain with altered expression following burn injury, and (2) investigate the changes in the brain metabolomics after burn injury. Mice were allocated into two intervention groups: a burn group that received burns over 7–8% of the total body surface area, and a sham group that received no injury burns. Mice were euthanized three months after their intervention procedure and their brains were collected for two “omics” analyses: transcriptomic analysis through RNA sequencing and metabolomic analysis using the high-resolution magic angle spinning (HR-MAS)’s nuclear magnetic resonance (NMR). RNA sequencing of the hippocampus revealed significant changes in several genes related to inflammation and neurodegenerative diseases including genes that regulate TNF and cysteinyl leukotriene receptors. Analysis of the cerebellum using NMR also revealed significant changes between groups where metabolites including N-acetylasparatate, myoinositol, lactate, alanine, glutamine, glutamate were all reduced in the cerebellums of mice with burns. These findings show that non-severe burns can have a physiological impact on the brain even after the burn site has healed, which may explain the increase in mental health hospital admission observed in burn patients. This research may inform future treatments in burn patients to alleviate the burden of long-term mental health conditions in these patients post injury.

*O.143*

**Non-Contact Dressing by Electrospinning—The Future of Wound Treatment?**

**Sebastian Nischwitz ^1^, Hanna Luze ^1^ and Lars-Peter Kamolz ^1,2^**
  ^1^  Medical University Graz
  ^2^  Coremed—Joanneum Research for schungsgesellschaft mbH

The development of modern dressing materials is a milestone in burn surgery in the 21st century. Although an ideal dressing material has not been developed to date, there are dressing materials that combine some of the optimal properties. One relatively new technology is dressing application using “electrospinning”. The SpinCare system creates an individually for mable, nanofibrillar and skin-like wound dressing that is applied once, does not require any dressing changes, does not restrict mobility and thus represents a comfortable, low-pain and contact-free alternative to conventional dressing materials. Further advantages are a high permeability for gases and for exudate drainage with simultaneous waterproofness from the outside.
In this presentation, we report on the novel technology of dressing by electrospinning as well as our experience with Spincare in the field of superficial burn and split-skin donor site wounds. We will also highlight potential future applications of the technology.
The therapy was well tolerated and accepted by all patients. In our patient population to date, there have been no wound infections or other wound complications. Long-term studies with a large number of patients in a controlled setting are needed to investigate the potential long-term superiority of the technology compared with conventional methods.

*O.144*

**Illusory Movements Physiotherapy Attenuates Muscle Wasting and Catabolism in Acute Large Thermal Injury**

**Bohumil Bakalář, Robert Zajíček, Magdalena Švecová, Marcela Lipperová-Grünerová**
  Kralovske Vinohrady University Hospital and 3rd Medical Phaculty of Charles University

**Backround**: Large thermal trauma is accompanied by hypermetabolism and devastation of both skeletal muscle and visceral proteins. Even after successful recovery from an injury, patients are often in a very unsatisfactory functional condition with a low quality of life, mainly due to muscle weakness.
Intensive rehabilitation is offered as a solution, but it is difficult for burn victims. A suitable alternative could be focal rehabilitation with functional proprioceptive stimulation (FPS), so-called illusory movements, which, using vibrations, create the illusion of muscle activity in the patient’s brain.
**Objectives**: To determine the impact of illusory movements on protein balance and catabolic markers in burns on >20% TBSA.
**Patients and methods**: A pragmatic cross-over study in patients with extensive burns in the acute phase of the burn disease, who are expected to be hospitalized for 40 days or more. Enrolled patients are trained with FPS either on days 10 to 24 or days 25 to 40 after the injury. during the study, the following parameters are measured and the following examinations are performed in addition to standard biochemical, hematological and imaging examinations:
  **Daily**: Intake and output of nitrogenous substances from the body, i.e., nitrogen balance; Resting Energy Expenditure using indirect calorimetry. On Days 10, 25 and 40 after the accident:
  - Glucose consumption in the body using a hyperinsulinemic euglycemic clamp;
  - Degree of insulin resistance according to HOMA-IR;
  - Evaluation of mitochondrial functions by muscle biopsy;
  - Determination of mitochondrial mass and dynamics in myocytes using MitoTracker® Red Cm-H2XRos;
  - Plasma and muscle levels of selected myokines;
  - Uncoupling protein 1 (UCP1) in white adipose tissue;
  - New sonographic methods for the diagnosis of muscle atrophy: shear wave elastography, microvascular imaging and contrast ultrasound.

**Results**: To date, 11 patients have been examined. None of the patients had any effect on vital signs during FPS. The current metabolic results are presented and discussed during the presentation.
**Conclusions**: FPS is a safe method for use in extensively burned patients. The results so far show a significant improvement in protein metabolism.

*O.145*

**Computational Modeling of the Post-Burn Immune Response**
**H. Ibrahim Korkmaz ^1,2,3,4,5^, Vivek M. Sheraton ^6^, Anouk Pijpe ^1,3,5^, Bouke B. Boekema ^1,5^, Evelien de Jong ^7^, Stephan G. F. Papendorp ^7^, Esther Middelkoop ^1,5^, Peter M. A. Sloot ^6,8^, Paul P. M. van Zuijlen ^1,3,4,9^**
  ^1^  Department of Plastic Reconstructive and Hand Surgery, Amsterdam Movement Sciences (AMS) Institute, Amsterdam UMC, Location VUmc
  ^2^  Department of Molecular Cell Biology and Immunology, Amsterdam UMC, Location VUmc
  ^3^  Burn Center, Red Cross Hospital
  ^4^  Department of Plastic and Reconstructive Surgery, Red Cross Hospital
  ^5^  Association of Dutch Burn Centres (ADBC)
  ^6^  Institute for Advanced Study, University of Amsterdam
  ^7^  Department of Intensive Care, Red Cross Hospital
  ^8^  ITMO University
  ^9^  Paediatric Surgical Centre, Emma Children’s Hospital, Amsterdam UMC, Location AMC

**Objectives**: Burns are characterized by a massive and prolonged acute inflammation, which persists for up to months after the initial trauma. Even though acute inflammation is initially necessary for wound healing, prolonged “out of control” inflammation may cause further damage, both locally and systemically. Despite detailed data about the cellular and molecular processes involved in inflammation, little progress has been made in modeling severe inflammation after burns. Until now, understanding of the inflammatory response after burn injury from data generated in basic biology cannot be translated into mechanistic understanding sufficient to predict system behaviour. Due to the complexity of the inflammatory process, the traditional scientific analysis may be inappropriate for describing the behaviour of complex biological systems. Computational simulation could meet this need. The aim of this study is to develop a computational model of the post-burn immune response.
**Methods**: The simulation domain was separated into blood and tissue compartments. Each of these compartments contained solutes and cell agents. Solutes comprise pro-inflammatory cytokines, anti-inflammatory cytokines, and inflammation-triggering factors. The solutes diffuse around the domain based on their concentration profiles. The cells include mast cells, neutrophils, and macrophages, and were modeled as independent agents. The cells are motile and exhibit chemotaxis based on concentration gradients of the solutes. In addition, the cells secrete various solutes that in turn alter the dynamics and responses of the burn wound system.
**Results**: We have developed an agent-based model to simulate and predict the innate immune response after burns. The model shows an overall increase in pro-inflammatory cytokine level at the initial stages followed by relative stabilization. The model further captures the sporadic influx of inactive neutrophils and macrophages to the wound site guided by chemotaxis. The cells are later activated based on the cytokine levels within the wound site providing an estimate of inactive, active, apoptotic and necrotic cell counts within the wound area.
**Conclusions**: The current model successfully demonstrates initial processes of the post-burn innate immune response. The agent-based model helps to quantify the cell count observed in the burn wounds with respect to solute levels. In the future, individual pro- and anti-inflammatory cytokines will be included.

*O.146*

**Surgical Simulation Training for Escharotomy: A Novel Course, Improving Candidate’s Confidence in a Time-Critical Procedure**

**John Gibson ^1^, Ian Pallister ^2^, Sarah Hemington-Gorse ^1^ and Jonathan Cubitt ^1^**
  ^1^  The Welsh Centre for Burns and Plastic Surgery
  ^2^  Swansea Bay University Health Board

**Background**: Circumferential deep burns on the limb lead to a constrictive, tourniquet-like effect causing critical limb ischaemia. The treatment, escharotomy, is a time-critical procedure, sometimes required before the patient arrives at a burn centre. At present, no practical method of teaching this procedure is incorporated into for mal educational courses.
**Methods**: The feasibility of a comprehensive education package to teach upper limb escharotomy was assessed in a group of plastic and general surgery trainees in Wales. Small group workshops focused on the clinical presentation of patients requiring escharotomy. Participants then executed this on a custom-made high-fidelity simulation upper limb model. The articulated limb has subcutaneous silicone fat which bulges upon decompression and a fingertip which turns pink indicating satisfactory reperfusion. A before and after five-point Likert scale was used to evaluate changes in participants’ self-assessed confidence in the surgical management of escharotomy. Statistical significance between scores was assessed using the Wilcoxon signed-rank test.
**Results**: A total of 34 participants took part. following completion of the course, general surgery trainees’ confidence in executing the procedure increased from a median score of 1.00 “not confident at all” (IQR 1.00–2.00) to 4.00 “fairly confident” (IQR 4.00–5.00, *p* < 0.01). Plastic surgery trainees’ confidence increased from a median score of was 3.00 “somewhat confident” (IQR 1.75–4.00) to 4.00 “fairly confident” (IQR 3.00–4.25, *p* < 0.01).
**Discussion**: We have developed a comprehensive simulator course that has been demonstrated to improve candidate’s confidence in performing escharotomy. The next stage in the course development is to confirm the results in a larger cohort. By developing this simulator course, we aim to improve emergency burn care education in the UK and globally.

*O.147*

**Estimation of Laser Doppler Imaging from Digital Photos: Towards a More Affordable Burn Assessment Solution**

**Andrea Rozo ^1^, Vanja Miskovic ^1^, Thomas Rose ^2^, Elkana Keersebilck ^2^, Carlo Iorio ^1^ and Carolina Varon ^1^**
  ^1^  Université Libre de Bruxelles
  ^2^  Hôpital Militaire Reine Astrid

**Objectives**: The attention received after suffering a burn injury is critical to ensure a satisfactory recovery. An adequate diagnosis and proper treatment can increase the probability of survival and reduce the healing time. Recently, laser doppler imaging (LDI) has been used alongside visual and tactile inspection of the burns by doctors, to assess severity and determine the need of surgery. The inclusion of this technology has improved the accuracy of the diagnosis. However, in places with limited specialized burn care, such as low- and middle-income countries, this technology is not available due to its high costs. This often leads to delays and sometimes incorrect diagnosis and treatment, resulting in higher mortality rates and morbidity cases. To overcome these challenges, this study proposes a novel approach to estimate the LDI from digital photos based on machine learning.
**Methods**: Pairs of digital and LDI images of burns of different depths, sizes, and locations on the body were collected during routine examinations of patients of the Burn Wound Unit of the Queen Astrid Military Hospital, Belgium. for each image, a mask of the body was manually created to remove the background. Then, a U-net convolutional neural network, with four levels, was trained with 500 pairs of the masked images (digital and LDI), using a loss function that only considered the pixels inside the mask. Tenfold cross-validation was used to obtain the best weights for the network. The model was tested on 87 pairs of images, and the performance was evaluated using mean absolute error (MAE), also computed only on the pixels within the mask.
**Results**: An average MAE of 25.19% (± 9.47%) was obtained. However, it was found that a very poor quality of the digital images in terms of contrast, brightness and sharpness deteriorated the performance of the model. The results could be improved by making the model more robust to quality changes of the images, by including more examples and using data augmentation.
**Conclusions**: These results suggest that it is possible to obtain an estimate of the LDI of a burn from its digital image, using convolutional neural networks. Additionally, they indicate that the proposed approach could be used for the development of a more affordable solution for burn assessment.

*O.148*

**New Approach to the Production of a Biovital Skin Graft Based on Human Acellular Dermal Matrix Produced In-House, In Vitro Revitalized Internally by Human Fibrobasts and Keratinocytes on the Surface**

**Wojciech Łabuś, Diana Kitala, Marcin Gierek, Artur Wielgórecki, Karolina Ziółkowska, Karolina Mikuś-Zagórska and Przemysław Strzelec**
  Centre for Burn Treatment in Siemianowice Slaskie

**Objectives**: Patients with extensive and deep burns who do not have enough donor sites for autologous skin grafts require alternative treatment methods. Tissue engineering is a useful tool to solve this problem. The aim of this study was to find the optimal method for the production of a biovital skin substitute based on acellular dermal matrix (ADM) and in vitro cultured fibroblasts and keratinocytes.
**Methods**: In this work, 9 methods of ADM production were assessed. The proposed methods are based on the use of the following enzymes: dispase II, collagenase I/EDTA, collagenase II/EDTA and mechanical perforation using DermaRoller and mesh dermatome. The obtained ADMs were examined (both on the side of the basement membrane and on the “cut off” side) by means of scanning electron microscopy, immunohistochemistry tests and strength tests. ADM was revitalised with human fibroblasts and keratinocytes. The ability of the in-depth revitalisation of cultured fibroblasts and their ability to secrete collagen IV was examined.
**Results**: The microscopic (H&E) evaluation of decellurization procedures showed totally decellurized dermal samples in all tested groups. The tests by scanning electron microscope revealed some significant differences in nanostructure of tested extracellular matrix of dermal basal membrane. Immunohistochemical tests (mice anti collagen IV A/B) did not show any presence of collagen type IV after application of decellurization procedures in all tested samples. Additionally, strengthStrength testing also revealed some significant differences. H&E and immunohistochemical tests showed some differences in revitalization level in the tested groups as well as the ability to secrete collagen type IV by inhibiting fibroblasts.
**Conclusions**: The optimal method of production of live skin substitutes is the colonisation of autologous fibroblasts and keratinocytes on the scaffold obtained using the two-step incubation method: trypsin/EDTA and dispase II.

*O.149*

**Challenges and Management of Burn Patients Transferred from the War Area: A Review of Eight Years**

**Elif Asfuroglu, Turgut Karaca, Zeynel Asfuroglu and Burhan Saban**
  İskenderun State Hospital

**Objectives**: We aimed to present our experience in patients who were burned in Syria and then transferred to our burns unit in Turkey for further treatment and discuss the unexpected challenges we encountered.
**Methods**: Retrospectively, we analysed the records of every Syrian patient who was admitted to our burns unit and treated in our unit between January 2014 and January 2022. Age, gender, cause of burn, time to transfer to our center, total burned body surface area, burn degree, hospitalization time, wound infection, sepsis, culture results, grafting/surgical interventions of every patient were recorded.
**Results**: A total of 1413 Syrian burn patients (children/adult = 745/668) were admitted to our burns unit. The most common cause of burn was flame burns in the winter, but scald burns was more common in children during the summer. Inhalation injury was present in 230/1413 patients on admission. Overall, 350/1413 patients have been intubated during transfer to our facility. Mean total burned body surface area was 35.8 ± 25.12% in children and 55.19 ± 22.59% in adults. Admission time was during the first 24 h of burn incident in 750/1413 patients. 645/1413 patients had a positive wound culture on admission. A positive blood culture was determined in 513/1413 patients at the time of admission. The most common bacteria obtained from the wound cultures were pseudomonas aeruginosa and Acinetobacter spp. Pandrug resistant pseudomonas aeruginosa was determined in the blood cultures of 15 patients at the time of admission. A total of 1309 patients were discharged from our burns unit, whereas 104 patients had mortal complications. The cause of death was pulmonary emboli in 5/104 patients, inhalation injury in 29/104 patients and sepsis in 70/104 patients.
**Discussion**: The most difficult challenge during the management of these patients has been the complications due to late admission and infection. Most of the patients were admitted to our unit with dehydration or systemic infection. We had to find a new algorhythm for these patients. Based on the analysis of the cultures from these patients two different antibiotic algorhthym was determined for children and adults. All patients were underwent surgery within 24 h and excision was performed. Hospitalisation was significantly longer among the patients with late admission. Sepsis was the most common cause of death among all the patients.
**Conclusions**: War is a humanitarian crime which has destructive effects on many areas. The civilian war in Syria has led to new challenges in the management of burns.

*O.150*

**Help from the Czech Republic to Burn Community in Ukraine**

**Robert Zajicek, Bohumil Bakalar and Vitaly Fetissov**
  Prague Burn Centre, Charles University, Third Faculty of Medicine, Faculty Hospital Kralovske Vinohrady

The war in Ukraine has had a devastating effect on the Ukrainian healthcare system. This conflict has practically destroyed the medical infrastructure and has severely limited access to medical services, meanwhile creating a massive influx of traumatic injuries. Burns have historically accounted for approximately 5% to 20% of military casualties in conventional warfare, depending on the tactical situation. Burns in civilians usually occur at home, as in peaceful conditions, thus the incidence of war-related civilian burn injuries remains low.
With the beginning of the conflict, the Czech Republic actively participated in assisting burn centers in Ukraine with the support of the MEDEVAC program. MEDEVAC is a permanent, government funded program in the Czech Republic that focuses on providing medical care and assistance to vulnerable groups in warzones. The Czech Republic has also provided material assistance in the for m of bandages, machines, as well as necessary medications. With the help of the Czech Red Cross, we have managed to build a secure distribution network in Ukraine for the transport of material and people between the burn centers in this active warzone and the Czech Republic. Our distribution network has also been used by members of the European Burn Association to send material assistance in the for m of three battery-driven dermatomes. The first critically burned Ukrainian patient, an 18-month-old child with 60% TBSA, was transported and treated at the Prague Burn Center. Here, the authors of this lecture present their experience with the organization, transport, and treatment of the child at the Burn Center in Prague.

*O.151*

**Response of a Single European Burn Center to Centelles Mass Casualty Burn Disaster. Enzymatic Debridement Utility**

**Jon Ander Aguirrezabala ^1^, Jordi Aguilera ^1^, Jordi Serracanta ^1^ and Juan Pedro Barret ^1,2^**
  ^1^  Department of Plastic Surgery and Burn Center, Vall d’Hebron Hospital Universitari
  ^2^  Department of Surgery, Universitat Autònoma de Barcelona

**Objectives**: Mass burn casualty disasters present with a big challenge due to the complex multidisciplinary management of severely burned patients and the limited capacity of the specialized centers. The literature is scarce, and so is the management of these disasters with enzymatic debridement (ED). The aim of this study is to look over the response of the emergency and health system to learn lessons for similar episodes in the future.
**Methods**: Retrospective observational analysis of nine patients transferred to Vall d’Hebron University Hospital Burn Center (Barcelona, Spain), as a consequence of a bell tower explosion on 30 December 2019.
**Results**: Fourteen people were injured after the explosion of gunpowder-containing bags in a bell tower during a cultural celebration. Nine casualties (six men and three women) suffered burn injuries that required admission in our Burn Center; all of them were directly transferred to our hospital and most arrived at the same time and not staggered. The mean age was 44.33 years (range 19–61 years), with burns covering a mean total body surface area (TBSA) of 15% (range 5–48% TBSA). One patient required invasive mechanical ventilation and intensive care management.
Most burns were located in face and limbs circumferentially and seven patients required ED in the first 24 h (Figures 1 and 2), with an average debrided TBSA of 6.1% (range 3–10% TBSA). Seven out of nine patients required at least one surgery. The average hospital stay was 23.33 days (range 2–53 days). No patients died.
**Conclusions**: Our experience proves that local resources can be overwhelmed, even without a huge number of casualties. The coordination with the emergency services was not optimal and it highlights the importance of actualized protocols to guide the assistance in mass casualty events.
ED was found a useful tool as it enabled us to treat a large number of patients in the early stages, preventing potential complications such as compartment syndrome.

*O.152*

**Management of Burned Patients during the Wildfires in Athens, July 2018**

**Dionysia Vasdeki ^†^, Dimitrios Krikonis ^‡^, Leonidas Koukoulomatis ^‡^, Maria Kalofonou ^‡^ and Panagiotis Bethanis ^†^**
  “Latseio” Burns Unit, “Thriassio” General Hospital of Elefsina
  † Resident.
  ‡ Consultant Plastic Surgeon.

**Objectives**: In July 2018, a series of wildfires begun in the coastal areas of Attica, which resulted in 102 deaths and 172 non-fatal injuries. More than 4000 buildings were either destroyed or badly damaged. This has been the second deadliest and destructive wildfire incidence in the 21st century, following the 2009 Black Saturday bushfires in Australia.
The Latseio burn centre is located in the west part of Attica. It has a capacity of 10 beds, whose number can extend up to 18 in urgent situations. during the wildfires in 2018, we treated 15 burn patients in our unit, which represents a great number of severely burned patients treated simultaneously in our unit. We present our experience in the management of these patients and their long-term follow up.
**Methods**: Data were collected from the medical inpatient and outpatient records of the 15 patients which were admitted and managed in our unit during the wildfires in 2018.
**Results**: In total we treated 15 patients with a mean age of 62 (18–83) years of age. All 15 patients had full-thickness burns. The mean total burned surface body area was 32% (ranging from 13% to 67%). All patients required at least one surgical intervention in the operating theatre, and in total, 53 surgical operations were performed. In total, 7 patients required intubation, either once or multiple times, and were hospitalised in the ICU of our Burn Centre. The mean inpatient stay was 47 (21–264) days. There were three deaths, in patients older than 73 years and with burns exceeding more than 30% of TBSA.
**Conclusions**: We present our experience in the management of severely burned patients during the wildfires in July 2018. Co-ordination between the burn centres, during such mass casualties and adequacy of staff specialised in the management of burned patients have been the cornerstones in the successful management of these casualties and continuous efforts need to be made, for burn centres to evaluate and apply well designed and up to date prehospital, hospital and rehabilitation plans for such devastating burn mass casualties.

*O.153*

**Impact of COVID-19 on the Antimicrobial Resistance Trends at the Bulgarian National Burn Center**

**Dario Carlo Premuselli ^1^, Maya Argirova ^1^, Magdalena Lesseva ^2^ and Ivan Balabanski ^1^**
  ^1^  Department of Burns and Plastic Surgery, UMHATEM “N. I. Pirogov”
  ^2^  Clinical Microbiology, UMHATEM “N. I. Pirogov”

**Objectives**: Antimicrobial resistance (AMR) is one of the main challenges for the world public health since years. This is particularly relevant among burns patients. Significant thermal injuries induce a state of immunosuppression that predisposes these patients to infectious complications. In the year 2020, the COVID-19 outbreak was declared a pandemic by the world. Already a complex challenge, AMR now needs to be addressed in a changing healthcare scenario. Theoretically, increased hand hygiene, attempts to limit patient contact, and decreased elective hospital procedures may reduce AMR pathogen selection and spread in the short term. However, the potential propagation and selection of AMR may also be worsened by an increased rate of antimicrobial prescription in the absence of clear guidelines regarding their use in COVID-19 patients. Burns patients may require multiple invasive procedures. This increases exposure to, and risk of, infections with hospital-associated pathogens that are often highly resistant such as Staphylococcus aureus (MRSA), coagulase-negative staphylococci (MR-CoNS), Pseudomonas aeruginosa, Acinetobacter baumannii and Klebsiella pneumoniae (CR-KP).
Our aim is to provide a comprehensive analysis of evolving trends of infections and antimicrobial resistance in pre- and post-COVID-19 era at the National Burn Center of UMHATEM “N.I.Pirogov” in Sofia, Bulgaria.
**Methods**: A retrospective study has been performed. A total of *n* = 32,077 samples were collected from wounds specimens and other biological specimens of patients with thermal injury during a 5-year period (January 2017 to December 2021). Culture, isolation, identification and antimicrobial susceptibility among *S. aureus*, CoNS, *A. baumannii* and *K. pneumoniae* have been performed according to microbiological laboratory routine practice guidelines.
**Results**: The present study showed significantly increased resistance rates to various antimicrobial agents during the COVID-19 pandemic compared with the previous years. Regarding *K. pneumoniae*, carbapenems resistance increased from 31.3% to 64.3%, ceftazidime-avibactam from 20% to 71.4%, doxycycline from 43.2% to 71.4%. Considering CoNS, the highest increment has been observed towards rifampicin, from 16.7% to 86.7%. *A. baumannii* confirmed its multiple resistance with a decrease in the clinical efficacy of doxycycline despite an in vitro sensibility of 80%.
**Conclusions**: Our findings highlight a significantly increased resistance to various classes of antibiotics, including: cephalosporins, β-lactam and β-lactamase inhibitor combinations, carbapenems, aminoglycosides, rifamycins and fluoroquinolones among *S. aureus*, CoNS and *K. pneumoniae*. The increased and inappropriate usage of antimicrobial agents and consequently future prevalence of AMR reinforce the urgency to implement appropriate antimicrobial stewardship programs (ASP) in the management of COVID-19 and burns patients.

*O.154*

**Procalcitonin-Guided Antibiotic Therapy in Septic Burns: An Effective Strategy?**

**Amel Mokline, Sajeda Sboui, Hana Fredj, Manel Ben Saad, Lamia Thabet, Imen Jemi and Amen Allah Messadi**

Intensive Burn Care Department

**Objectives**: In severely burned patients, elucidating systemic inflammatory response syndrome (SIRS) from an infectious source (sepsis), can be challenging since the clinical features are similar. Our study aims to assess the interest of plasma procalcitonin (PCT) biomarker to guide the initiation, modification and discontinuation antibiotics in septic burn patients.
**Methods**: We conducted a prospective, observational study in a 20-bed Burn Intensive Care Unit in Tunisia, for 13 months (August 2018–September 2019). Burn patients admitted in ICU who developed sepsis were included. Sepsis was retained according to the French Burn Association Criteria for the presence of infection. Patients who died or were discharged before day 3 of the protocol were excluded. Serum PCT was measured over the entire septic episode every 48 h until resolution of infection, based on clinical signs and decrease in PCT about 80% compared with its initial value.
**Results**: A total of 120 septic patients admitted to the Burn ICU were included in our study. The mean age was 32 ± 17 years. TBSA was 32 ± 14%. UBS was 40 ± 25 and ABSI was 6 ± 2. Secondary transfer was reported in 55% of cases. Burns were related to domestic accidents in 35% of cases, suicide attempts in 33% and work-related accidents in 25.5% of cases. They were mainly thermal burns in 79% of cases and electrical burns in 21%. Sepsis occurred 3 days after burns. Initial PCT was positive (>0.69 ng/mL) in all patients with a median value of 12 ng/mL (maximum 200 ng/mL).
Monitoring of kinetics of PCT allowed us to judge the effectiveness of the initial antibiotic therapy with a threshold of 43.5% decrease at day 3 of treatment with a better sensitivity and specificity of 79.6% and 87.7%, respectively. In addition, PCT monitoring allowed a reduction in the duration of antibiotic therapy of 5 ± 2.8 days versus 8 to 10 days before the use of PCT.
**Conclusions**: PCT-guided antibiotic in septic burns was effective in assessing effectiveness of the initial antibiotic therapy and in reducing antibiotic exposure and selective pressure of multidrug-resistant bacteria.

O.155

**Multidrug-Resistant *Pseudomonas aeruginosa* Isolates from Burn Patients: Phenotypic Profile and Molecular Typing**

**Marzak Metref ^1,2^**

^1^  University Hospital Kouba Algiers
^2^  Department of Medicine, Benyoucef Benkhedda University

**Objectives**: Infections caused by multidrug-resistant (MDR) organisms are common in burn patients and have been mainly implicated in higher morbidity and mortality rates, increased length of hospital stay, and considerably higher healthcare-related cost following burns. Given the relevance of Pseudomonas aeruginosa (PA) in burn wound infections we sought to investigate the prevalence of multidrug-resistant (MDR) Pseudomonas aeruginosa (PA) producing extended-spectrum beta-lactamases (ESBLs) and metallo-betalactamases (MBLs) in our burn center in Algeria. Due to its great adaptability and its ability to acquire antimicrobial resistance traits, PA is considered a model pathogen in the field of antibiotic resistance and becomes, for many reasons, an issue in burn centers, limiting antibiotic choices. Our study first reviewed the mechanisms of antibiotic resistance, and the prevalence of MDR bacteria isolated from burn wounds and finally focuses on PA stains. Methods: Between April 2016 and October 2019, 1585 wound swabs were collected from 258 patients admitted in our burn center with suspected burn wound infections. Antibiotic susceptibility testing was performed by agar diffusion and the phoenix automated method. Resistance genes were identified by PCR, and molecular typing of isolates was carried out by enterobacterial repetitive intergenic consensus (ERIC) sequences-polymerase chain reaction (PCR). Results: The prevalence of bacteria isolated from burn wound surface swabs was 79.12% (1254 swabs) Gram-negative bacteria were the most isolated 68.95% followed by Gram-positive cocci, 27.7%. PA was the most common Gram-negative bacteria isolated, 73 (33.9%), 47 strains were carbapenem-resistant and 26 were carbapenem-sensitive. Among the 47 non-redundant MDR PA strains 59.57% were phenotypically ESBL-positive, and 100% were phenotypically MBL-positive isolates. The ESBL-positive isolates were subsequently screened for six groups of bla genes encoding ESBL-type enzymes. out of the 28 ESBL-producing strains, 23 (82.14%) were blaCTX-M2-positive, 18 (38.29%) were blaPER-positive, and 16 (34.04%) were blaTEM-positive, whereas 5 (17.9%) were co-harboring blaCTX-M2, blaTEM, and blaPER genes. The blaSHV, blaVEB and blaGES genes were not detected in any of the ESBLs-positive isolates. All 47 strains were screened for the bla NDM-1, blaIMP and, blaVIM genes; however, none of these genes were detected. In total, 45 (95.74%) of the isolates were positive for the oprD gene. Finally, ERIC PCR revealed six distinct PA clones among the blaCTX-M2 positive strains.
**Conclusions**: This study reveal the presence of three ESBL genotypes in the clinical PA strains isolated in our burn center, two of which (blaCTX-M2 and blaPER) were detected for the first time. In Algeria, they are very likely to impact patient outcomes adversely. Therefore, our findings could potentially facilitate clinical decision-making and participate in an antibiotic stewardship program implementation in order to control nosocomial infections in burn units.

*O.156*

**Sepsis and Multiorgan Dysfunction Syndrome (MODS) in Severe Burns: A Focus on Data in the Turin Burn Center**

**Anna Pensa ^1,2^, Giulia Zerbinati ^1,2^, Valeria Malvasio ^3,4^, Francesco Giuseppe De Rosa ^5^ and Maurizio Stella ^2^**
  ^1^  Surgery Department, Departemente of Surgical Science
  ^2^  Burn Center and Plastic Surgery, Surgical Department, CTO Hospital
  ^3^  Department of Pediatric Surgery, Regina Margherita Childrens Hospital, Turin, Italy
  ^4^  Department of Pediatric General Surgery, City of Science and Health, Regina Margherita Childrens Hospital
  ^5^  Department of Medical Sciences, Infectious Diseases, University of Turin

**Objective**: Extensive burn injuries represent a severe trauma associated with high morbidity and mortality. Despite the improvement in survival rate due to advances in intensive care treatment, wound management, surgery and infection control practice, sepsis is the most common complication of burns and remains the main cause of mortality. Moreover, sepsis represents a severe risk for Multiorgan Dysfunction Syndrome (MODS) even today.
**Materials**: This review aims to analyze recent literature concerning sepsis and related MODS resulting from severe burns. Data from Turin Burn Center from January 2015 to July 2019 including demographic and clinical variables, inhalation injury, revised Baux score, Charlson score, surgical approach, sepsis rate and in hospital mortality are presented.
**Results**: According to several studies the most important risk factors for development of MODS are TBSA > 20%, age, male gender, hypoperfusion, inhalation injury and sepsis. We enrolled 324 patients with male predominance (68%) and with a median age of 55 years. At admission the median TBSA was 19%, 13,3% had an inhalation injury and a median RBS of 79.44; the etiology of burn was flame (77.8%), the median of Charlson score was 2.1. The prevalence of mechanical ventilation was 33.6%, a central venous line was inserted in 55.6% of patients, an arterial line in 30.2%, dialytic treatment was applied in 12% of cases. Sepsis was diagnosed in almost 20% of the patients, whereas in-hospital mortality was 12.5%.
**Conclusions**: Nowadays, in burn patients sepsis remains the main cause of death. In the literature, the incidence of sepsis is between 3% and 30%, 20% in our population. To reduce mortality an optimal treatment of sepsis is mandatory: early diagnosis, prompt administration of antibiotics, appropriate haemodynamic and organ dysfunction support represent lifesaving procedures.

*O.157*

**Early Bloodstream Infection and Their Impact on In-Hospital Mortality in Severe Burn Patients**

**Silvia Scabini ^1^, Anna Pensa ^2,3^, Nour Kassam ^1^, Silvia Corcione ^1^, Simone Mornese ^1^, Maurizio Stella ^3^ and Francesco Giuseppe De Rosa ^1^**
  ^1^  Departement of Medical Sciences, Infectious Diseases, University of Turin
  ^2^  Surgery Department, University of Turin, Italy; Burn Center and Plastic Surgery, Department of General and Specialized Surgery, City of Science and Health, CTO Hospital, Torino, Italy
  ^3^  Burn Center and Plastic Surgery, Department of General and Specialized Surgery, City of Science and Health, CTO Hospital

**Objectives**: Severely burn patients are at high risk for bloodstream infections (BSI) due to loss of the skin barrier function and state of immunosuppression induced by significant thermal injuries, invasive procedures and prolonged hospitalization. However, the effects of BSI on mortality remains controversial in the literature. This retrospective study was conducted to describe the epidemiology, risk factors for BSI and their impact on mortality in a burn intensive care unit. Additionally, we investigated risk factors and mortality associated with multidrug-resistant Gram-negative bacteria BSI [multidrug-resistant *Pseudomonas aeruginosa* (MDRPA), carbapenem-resistant *Acinetobacter baumannii* (CRAB), carbapenem-resistant Klebsiella pneumoniae (CPKP)].
**Materials/methods**: All patients with burn injury admitted to the 8-bed burn unit at CTO Hospital of Turin between January 2015 and July 2019 were included in the study. Demographic and clinical variables, including inhalation injuries, revised Baux score, BSI and mortality were collected.
**Results**: There were 183 episodes of BSI among 324 cases with 241 microbiological isolates. In total, 88 patients had at least one positive BSI (27.2%). Among them, 26 patients (29.5%) had more than three episodes of BSI during their stay. The median time to first positive blood culture was 10 days. Most of the first positive blood cultures were documented during the first week of hospitalization (37/88; 42). The leading isolate was A baumannii (24.1%), followed by *P. aeruginosa* (20.7%). Klebsiella spp accounted for only 7% of total isolates. Many isolates showed antimicrobial resistance, particularly in Gram-negative bacteria. Among *A. baumannii* isolates, 93% of them were CRAB, while the rate of MDR strains in *P. aeruginosa* isolates was 48%. The prevalence of BSI due CPKP was around 5% (5.5%). At multivariate analysis, early invasive lines placement (OR: 3.576 95% CI: 1.596–8.017) surgical procedures (OR: 3.608 95% CI: 1.238–10.517) and a higher revised Baux score (OR: 1.019 95% CI: 1.008–1.031) increased the risk of acquiring BSI. Prior rectal colonization (OR: 13.543 95% CI: 3.553–51.621) was a predictor of MDR Gram-negative BSI. Kaplan–Meier survival curves showed no difference in survival between patients with BSI and those without BSI Log rank [*p* = 0.1169 HR: 0.6318 (95% IC 0.3399–1.1745)].
In our local setting, BSI are predominantly caused by non-fermenting Gram-negative bacteria, with high rate of carbapenem resistance since the earliest days of hospitalization. These data emphasize the need for effective infection control measures, early invasive lines and tube removal and appropriate use of antibiotics.

*O.158*

**Comparison of Infections among Critically Ill Burn Patients versus Toxic Epidermal Necrolysis and Other Critical Skin Syndromes**

**Jesús Soto Gómez Cambronero, Javier Vejo Gutierrez, Raquel Yébenes Calvo, Javier Rábano Alonso, Lucía Cachafeiro Fuciños, Alexander Agrifolio, Irene Isabel Seises García, Alba López Fernández, Maria Eugenia Graciani Cantisan, Marina Lavara Ruiz, Belén Guzmán Bernardo, Santos Manuel Sánchez Sánchez and Abelardo García de Lorenzo Mateos**
  University Hospital La Paz

**Objectives**: Some cutaneous pathologies such as Toxic Epidermal Necrolysis(TEN)/Steven Johnson Syndrome(SJS), drug reaction with eosinophilia and systemic symptoms(DRESS) or blistering diseases may affect large skin areas and require admission to intensive care unit (ICU) in burn center. Due to its low incidence, we do not have much data on infections in this group of patients.
The aim of this study is to compare microbiological infection timeline and etiology between these two groups of patients.
  **Methods**: This was an single-center retrospective study carried out from January 2018 to December 2021 in a burn center in University Hospital La Paz, a third-level hospital. All demographic and microbiological (blood, bronchoalveolar secretions, urine and skin cultures) data were analysed. Differences in timeline to infection since admission to ICU and most frequent pathogens were compared between the two groups of patients.
  **Results**: In total, 118 samples were analysed from 42 different patients, of which 15 were ETN/SJS/DRESS(mean TBSA 39%) and 27 critically burn patients (mean TBSA 33%). The mean age was 63 years in the first group and 50 in the second one. In the group of burn patients, most of them were men (81%), whereas in the group of cutaneous syndromes just 8 (53%) were men.
*Staphylococcus epidermidis*, *Escherichia coli*, *Pseudomonas aeruginosa* and *Haemophilus influenzae* were the most frequent pathogens isolated in all kinds of samples.
Among the burn patients group, Gram-bacteria were predominant, being *Escherichia coli* (blood and urine), *Haemophilus influenzae* (bronchoalveolar secretions), *Pseudomonas aeruginosa* (skin) the most frequent pathogens. The mean time to infection since admission to ICU was 11.68 days.
  TEN/SJS/DRESS group, Gram + pathogens were isolated in a higher rate although there was no significant statistical difference between groups, probably because of a small sample size. In general terms, *Staphylococcus epidermidis* (blood) and *Pseudomonas aeruginosa* (bronchoalveolar secretions) were the most frequent pathogens isolated. As in the other group, *Escherichia coli* and *Pseudomonas aeruginosa* were more frequent in urine and skin cultures, respectively. The mean time to infection since admission to ICU was 3.33 days being statistically significative different (*p* < 0.05).
  Burn patients needed mechanical ventilation (77% vs. 26%) and vasoactive drugs (70% vs. 33%) more frequently. However, the mortality rate in this group was lower (11% vs. 26%).
**Conclusions**: TEN/SJS/DRESS patients had positive cultures earlier than burn patients.
  The most frequent bacteria isolated were similar between groups. However, there is a higher rate of infection by Gram positive bacteria in TEN/SJS/DRESS group compared with the burn patients group.

*O.159*

**Is There a Place for PSP (Pancreatic Stone Protein) in the Critically Ill Burn Patient?**

**Alba Lopez-fdez and Manuel Sanchez-Sanchez**
  La Paz University Hospital

**Objective**: to evaluate the response of the PSP in the critically ill burn patient both in the initial insult and in subsequent therapeutic insults, as well as to assess its ability to differentiate the inflammatory response from sepsis.
**Methods**: prospective, single-centre study, where 261 samples were collected from 20 patients in the Critical Burn Unit between November 2020 and September 2021. Plasma levels of PSP, C-reactive protein (CRP), procalcitonin (PCT), leukocytes and lactate were collected daily, in addition to other data on infection, sepsis and external aggressions (surgeries, enzymatic debridements and aggressive dressings), until discharge from the Burn Intensive Care Unit (BICU). We statistically studied the results using the Mann–Whitney *U* test, the Kruskal–Wallis test and Spearman’s correlation.
**Results**: Overall, 20 patients were included with a mean age of 51 ± 16 years, with a burned body surface area (BSA) percentage of 29 ± 16% and an Abbreviated Burn Severity Index (ABSI) of 7.5 ± 2.3, 20% of whom were female. A significant elevation of PSP was observed during the infection phases compared with measurements during the non-infection phases, with a mean PSP of 360 ± 196 ng/mL vs. 149 ± 155 (*p* 0.001), respectively. 35% of patients had at least one episode of infection, with mean day of onset being 15 ± 6.5. The most frequently isolated germs were *Klebsiella pneumoniae* and *Pseudomonas aeruginosa* (in 3% and 2.2% of the samples analysed, respectively). In the first 3 days, considered the resuscitation phase, PSP levels were higher than the normal range, with the mean on day 1 being 135 ± 145 ng/mL, day 2 143 ± 187 ng/mL and day 3137 ± 180 ng/mL, with higher levels found later (on day 9 mean 305 ± 229 ng/mL). No significant differences were found in measured PSP levels during new therapeutic aggressions, 157 ± 89 vs. 160 ± 91 with *p* 0.9. PSP showed correlation with the other classical markers CRP, PCT, leukocytes and lactate, with *p* < 0.05 but with low significance.
**Conclusions**: Our data suggest that in infection, there are more significant increases in PSP than during the resuscitation phase and the therapeutic aggressions required by the burn patient; therefore, we could conclude that PSP is useful to discriminate between inflammatory response and sepsis.

*O.160*

**Development of the Patient Scale of the Patient and Observer Scar Assessment Scale (POSAS) Version 3.0**

**Michelle Carrière ^1,2,3,4^, Paul Van Zuijlen ^1,2,5,6^, Zephanie Tyack ^7^, Marjan Westerman ^3,8^, Anouk Pijpe ^1^, Jon Pleat ^9^, Annekatrien van der Kar ^10^, Jason Brown ^11^, Riekie de Vet ^3^ and Wieneke Mokkink ^3^**
  ^1^  Burn Center and Department of Plastic, Reconstructive and Hand Surgery, Red Cross Hospital
  ^2^  Department of Plastic Reconstructive and Hand Surgery, Amsterdam UMC Location, Vrije Universiteit Amsterdam, The Netherlands
  ^3^  Department of Epidemiology and Data SCience, Amsterdam UMC, Amsterdam Public Health Research Institute, Vrije Universiteit
  ^4^  Association of Dutch Burn Centers
  ^5^  Paediatric Surgical Centre, Amsterdam UMC Location, University of Amsterdam, Emma Children’s Hospital
  ^6^  Amsterdam Movement Sciences (AMS) Institute, Amsterdam UMC
  ^7^  Child Health Research Centre, University of Queensland
  ^8^  Division of Life Science, Amsterdam UMC, VU University
  ^9^  Department of Plastic, Reconstructive en Handsurgery, Restore Research and Southmead Hospital
  ^10^  Department of Plastic, Reconstructive en Handsurgery, Onze Lieve Vrouwe Gasthuis
  ^11^  Burn Center, Royal Brisbane and Women’s Hospital

**Objective**: The Patient and Observer Scar Assessment Scale (POSAS) is widely used for measurements of scar quality. The current version 2.0 was due for an extensive update, especially the patient scale. This study elaborates on the choices that were made in the development of the Patient Scale of the POSAS 3.0, based upon new information obtained from patients during focus groups and pilot test interviews.
**Methods**: Focus group interviews were held in The Netherlands and Australia to identify scar quality characteristics and included 45 participants. All focus groups were audio-recorded and transcribed ad verbatim. The anonymized transcripts were analyzed using a thematic analysis. Thereafter, item generation and refinement took place. Pilot test interviews were performed with 15 participants in Australia, The Netherlands, and the United Kingdom. The Three-Step Test-Interview method was used to test the comprehensiveness of the scale, as well as the relevance and comprehensibility of the included items.
**Results**: We discussed the selection and wording of nine included items. The merging of eight characteristics into four items was discussed. Finally, the reasons for exclusion of twenty-three characteristics were given.
**Conclusions**: Based upon the unique and rich material of patient input obtained, two versions of the Patient Scale of the POSAS3.0 were developed: the Generic version, and the Linear scar version. The discussions and decisions taken during the development are informative for a good understanding of the POSAS 3.0 and are indispensable as a background for future translations and cross-cultural adaptations.

*O.161*

**Longitudinal Follow-Up of Scar-Related Outcome Parameters in Children after a Burn Injury**

**Mieke Anthonissen ^1,2,3^, Peter Moortgat ^1^, Jill Meirte ^1,2^, Cynthia Lafaire ^1,4^, Lieve De Cuyper ^1,4^ and Koen Maertens ^1,5^**

  ^1^  Oscare, Organisation for Burns and Scars Aftercare and Research
  ^2^  Rehabilitation Sciences and Physiotherapy, University of Antwerp
  ^3^  KU Leuven, Faculty of Kinesiology and Rehabilitation Sciences
  ^4^  ZNA Stuivenberg, Burn Center
  ^5^  Clinical and Lifespan Psychology, Vrije Universiteit Brussel

**Objectives**: According to literature, hypertrophic burn scars mature within 2 years with scar erythema as the key indicator of scar maturation. No longer follow-up periods than 2 years were mentioned. Especially in children, we expect an important influence of growth on scar tissue. The aim of this study was twofold. (1) Are hypertrophic burn scars in children still significantly more erythematous than normal skin 5 years after injury? (2) Can we still observe improvement of various scar characteristics after 5 years compared with 2 years after injury?
**Methods**: Children aged 14 years or younger with burn scars were eligible for this study. Scars were measured at baseline (=at least 2 years after burn injury) and at follow-up (=3 years after baseline). All scars were assessed using patient and observer version of POSAS 2.0. Minolta Chromameter and DermaLab were used to measure, respectively scar redness and elasticity. All patients without significant baseline differences were excluded.
A one-sample *t*-test was used to determine differences in erythema between scars and healthy skin 5 years after the injury. A paired-sample *t*-test was used to investigate significant improvements of scar parameters after 5 years compared with 2 years after injury.
**Results**: In total, 86 patients were eligible, but 19 patients were excluded. Mean scar age was 30 months.
  (1) Five years after burn injury, there was still a significant difference (1.81 ± 1.60) between scars and healthy skin in scar redness (*p* < 0.0005; d = 1.11).
  (2) There was a significant difference between POSAS-P/O sum of scores of baseline and follow-up measurement (*p* < 0.0005), with a mean difference of 6.77 ± 8.11 for POSAS-P sum of scores and 7.77 ± 6.76 for POSAS-O sum of scores. AdditionallyMoreover, a significant difference in redness of 1.20 ± 2.44 between baseline and follow-up measurement was found (*p* < 0.0005, d = 0.50). No significant difference in elasticity between baseline and follow-up measurement was seen.

**Discussion**:
  (1) Even 5 years after a pediatric burn injury, scar redness can still be significantly higher compared with healthy skin, which is in contrast with the general idea that burn scars are considered mature 2 years after injury.
  (2) Scar-related outcomes like POSAS and redness can still improve up to 5 years after burn injury.
**Conclusions**: Burn scars do not seem to be fully matured after 5 years, which could be related to the natural growth of children. However, pediatric burn scars tend to improve up to 5 years after the injury; therefore, further follow-up and rehabilitation therapy could be required.

*O.162*

**Patients’ Satisfaction and Scar Quality of the Donor Site after Split-Thickness Skin Grafting**

**Danielle Rijpma ^1,2^, Annebeth Meij-de Vries ^1,3,4^, Annika Reuvers ^1,2^, Tjitske Haanstra ^5^, Paul van Zuijlen ^1,2,4,6,7^ and Anouk Pijpe ^1,2,5,7^**
  ^1^  Burn Centre of the Red Cross Hospital
  ^2^  Plastic, Reconstructive and Hand Surgery, Amsterdam UMC Location Vrije Universiteit Amsterdam
  ^3^  Department Surgery, Red Cross Hospital
  ^4^  Department of Pediatric Surgery, Amsterdam UMC, location AMC
  ^5^  Association of Dutch Burn Centres
  ^6^  Department of Plastic, Reconstructive and Hand Surgery, Red Cross Hospital
  ^7^  Amsterdam Movement Sciences Institute

**Objectives**: Split-thickness skin grafts (STSG) play an important role in the surgical treatment of skin defects. STSG harvesting causes a new wound: the donor site. Since donor sites are iatrogenic, the patients’ satisfaction with and outcomes of these wounds are essential for quality of healthcare. However, little is known about this topic. Therefore, the aim of this study was to investigate patients’ satisfaction with donor sites and patients’ opinions on the scar quality. The findings might clarify leads on which aspects of donor site management could be improved.
**Methods**: This cross-sectional observational study investigated patients’ satisfaction with donor sites 12 (+/−3) months after STSG surgery in the burn center of the Red Cross Hospital, Beverwijk, The Netherlands. Patients of all ages who underwent STSG surgery between November 2020 and May 2021 were considered eligible for this study. Exclusion criteria were legal incapacity, or insufficient knowledge of the Dutch language. Patients received a survey including (1) nine questions on patients’ donor site satisfaction: seven multiple-choice questions, scored as very satisfied, satisfied, neutral, dissatisfied, very dissatisfied, and two open questions, and (2) the patient scale of the Patient and Observer Scar Assessment Scale. Statistical analysis consisted of descriptive statistics and analysis of the potential association of satisfaction with patient, clinical, and scar quality characteristics.
**Results**: In total, 65 eligible patients received the survey. The preliminary data of 20 patients, including 9 males, were analyzed. The general satisfaction item showed that 10 patients (50%) were ‘very satisfied’, 7 patients (35%) were ‘satisfied’, and 3 patients (15%) were ‘neutral’ with their donor site; none of the patient was dissatisfied. Satisfaction on the size of the donor site: 5 patients (25%) ‘very satisfied’, 10 patients (50%) ‘satisfied’, 4 patients (20%) ‘neutral’, and 1 patient (5%) was ‘dissatisfied’. Satisfaction on post-operative complaints: 5 patients (25%) ‘very satisfied’, 7 patients (35%) ‘satisfied’, 7 patients (35%) ‘neutral’, and 1 patient (5%) ‘dissatisfied’. Questions on other aspects of donor site satisfaction showed comparable results.
**Conclusions**: Preliminary results suggest patients are considerably satisfied with their donor sites. Data collection and analysis is currently being completed. The results could give insights into improvements of donor site management to optimize outcomes and patients’ satisfaction on donor sites.

*O.163*

**Working towards Holistic Scar Assessment and Improved Shared Decision-Making in Global Burn Care**

**Elleke Munk ^1^, Anna Davies ^2^, Zephanie Tyack ^3^, Craig McBride ^3^, Dale Edgar ^4^, Mariëlle Vehmeijer ^1^ and Amber Young ^5^**
  ^1^  Department of Plastic and Reconstructive Surgery, Radboudumc
  ^2^  Centre for Academic Child Health, University of Bristol
  ^3^  Centre for Children’s Burns and Trauma Research, Child Health Research Centre, The University of Queensland
  ^4^  State Adult Burn Unit, Fiona Stanley Hospital, Murdoch, Australia, Burn Injury Research Node, The Institute for Health Research, The University of Notre Dame Australia, Fremantle, Australia, Fiona Wood Foundation, Fiona Stanley Hospital
  ^5^  Bristol Centre for Surgical Research, Population Health Sciences, Bristol Medical School, Bristol University

**Objectives**: Heterogeneity in burn care outcome reporting hampers evidence-based treatment. Cutaneous burn scars impact various aspects of life. Current scar assessment tools, however, typically focus on scar quality outcomes. Consensus is needed on which outcomes to capture, ensuring they are relevant to patients, clinicians and researchers. Outcomes related to cutaneous scarring after a burn injury were identified, discussed and analysed, incorporating the views of both patients and healthcare professionals.
**Methods**: Secondary analysis of data collected during a Delphi survey for a burn Core Outcome Set. Outcomes related to burn scarring were identified from this dataset by an international panel consisting of patients, healthcare professionals and researchers. Outcomes were selected through two online survey rounds and an international consensus meeting. Outcomes from the second survey round were considered agreed on if ≥80% of the participants and both patient and parent voted them to be related or unrelated to scarring. Undecided outcomes were carried through to the consensus meeting. Views of patients and healthcare professionals or researchers were compared.
**Results**: Fifteen healthcare professionals, a patient and a parent participated in the survey rounds. during the two survey rounds, 40 outcomes reached ≥80% agreement related to scarring and 10 outcomes unrelated to scarring. The remaining 50 undecided outcomes were grouped into 11 domains: (psycho)social, burn-wound-related, complications, dysphagia/dysphonia, short-term systemic effects, organ dysfunction or failure, pain and discomfort, long-term (systemic) effects, hospital stay, treatment and costs. In an international consensus meeting with representatives from four countries, six patients and thirteen healthcare professionals of various disciplines reached consensus that 8 outcomes from these domains were related to scarring (≥80% votes) and 11 outcomes were near consensus (60–79% votes). Patient input gave a different perspective on various outcomes such as short- and long-term systemic health issues, unexpectedly relating them to scarring.
**Conclusions**: Outcomes related to cutaneous burn scarring go beyond outcomes used in current scar assessment tools. Although scar quality remains important and can be adequately assessed with scar scales, a more holistic approach appears to be necessary to cover domains such as systemic effects. Patient input is essential in identifying outcomes directly or indirectly related to scarring. Since our panel only consisted of Western participants, future work should incorporate the views of patients and healthcare professionals from low- and middle-income countries.

*O.164*

**Evidence of Effectiveness of Massage on Rehabilitation Treatment of Burn Scars**

**Henri Bibaki ^1^, Daniela Arena ^2^ and Danila Toscano ^2^**

  ^1^  Centro Move Different
  ^2^  Città della Salute e della Scienza—CTO

**Objective**: Scar massage is often used in the rehabilitative treatment of pathological burn scars. However, even though this treatment is suggested in various textbooks, scientific articles and guidelines, its real effectiveness is uncertain. In fact, from the clinical experience of the physiotherapists of the M.F.R.U. of the C.T.O. Hospital in Turin, it emerged that massage therapy, other than being less influential on the ROM and on the scar functionality, could aggravate some characteristics of pathological burn scars. Therefore, the objective of this study is to review, in the literature, the evidence of effectiveness of manual massage therapy in the treatment of pathological burn scars.
**Methods**: This systematic literature review was carried out in compliance with the PRISMA Statement’s international directives and with the consultation of these biomedical database: PubMed, CINAHL Complete, Cochrane Library, Embase and OVID MEDLINE. Other scientific sources, such as textbooks and guidelines, were used for the completion of this study.
**Results**: In total, 12 studies were selected through the research process: 4 RCTs, 2 CCTs, 2 literature reviews and 4 systematic literature reviews. Overall, the outcome measures of the pathological burn scars taken into account in these 12 studies were: ROM, pliability, vascularity, pruritus, pain, anxiety, depression, skin status, burn-specific health, thickness, melanin, TEWL, sebum and elasticity.
**Conclusions**: From the analysis of the studies taken into account, there was no definitive confirmation of the effectiveness, the ineffectiveness or the harmfulness of manual massage therapy on pathological burn scars. This conclusion was achieved because the outcome measures of the studies were conflicting. In order to achieve, if not a definitive answer, at least a more homogenous and updated view on this subject, other RCTs are needed. These RCTs should be carried out combining the use of objective assessment technologies and the use of the scar assessment scales for the evaluation of the characteristics related to the patients’ symptoms.

*O.165*

**The Course of Patient-Reported Scar Quality in Patients with Intermediate Burns up to 5–7 Years Postburn**

**J.N. Dijkshoorn ^1^, M.E. van Baar ^2,3^, A. Pijpe ^4,5,6^, M.K. Nieuwenhuis ^7,8,9^, H. Goei ^1,10^, C.H. van der Vlies ^1,11^ and I. Spronk ^2,3,12^**
  ^1^  Burn Centre, Maasstad Hospital, Rotterdam, The Netherlands
  ^2^  Association of Dutch Burn Centres, Maasstad Hospital, Rotterdam, The Netherlands
  ^3^  Department of Public Health, Erasmus MC, Rotterdam, University Medical Center, Rotterdam, The Netherlands
  ^4^  Burn Centre, Red Cross Hospital, Beverwijk, The Netherlands
  ^5^  Department of Plastic, Reconstructive and Hand Surgery, Amsterdam Movement Sciences, Amsterdam UMC, Location VUmc, Amsterdam, The Netherlands
  ^6^  Association of Dutch Burn Centres, Beverwijk, The Netherlands
  ^7^  Association of Dutch Burn Centres, Martini Hospital, Groningen, The Netherlands
  ^8^  University of Groningen, University Medical Center Groningen, Center for Human Movement Sciences, Groningen, The Netherlands
  ^9^  Research Group Healthy Ageing, Allied Health Care and Nursing, Hanze University of Applied Sciences, Groningen, The Netherlands
  ^10^  Department of Surgery, Amsterdam Movement Sciences, Amsterdam UMC, Vrije Univeristeit, Amsterdam, The Netherlands
  ^11^  Department of Surgery, Erasmus MC, University Medical Centre Rotterdam, Rotterdam, The Netherlands
  ^12^  Dutch Burns Foundation, Beverwijk, The Netherlands

**Introduction**: Burn scar maturation can take several years but is generally studied relatively shortly after injury.
**Objective**: We investigated the course of patient-reported scar quality up to 5–7 years postburn.
**Methods**: Patients with intermediate burns and ≤20% total body surface area (TBSA) burned completed the Patient Scale of the Patient and Observer Scar Assessment Scale (POSAS 2.0) at T1 (median: three months), T2 (median: 28 months) and T3 (median: 63 months) postburn.
**Results**: In total, 58 patients (21 children; 37 adults) participated with a median TBSA burned of 6.3%. Median patient-reported scar quality was 4.2 at T1, 2.2 at T2 and 3.4 at T3. Two-thirds of patients (66%) reported a higher score (worse score) at 63 months compared with 28 months postburn, whereas 14% reported an identical, and 21% a lower score (better score). At any assessment, largest differences with normal skin were reported for scar color. Univariate predictive factors of long-term patient-reported scar quality were scar quality at three months (*p* = 0.002) and 28 months postburn (*p* < 0.001), %TBSA full-thickness (*p* = 0.033), length of hospital stay (*p* = 0.003), and number of surgeries (*p* < 0.001).
**Conclusions**: Our study showed that the course of patient-reported burn scar quality worsened over time in the majority of our sample. The course of patient-reported burn scar quality fluctuates considerably over time. These insights are useful in the counseling of burn patients’ expectations on the maturation of their scars.

*O.166*

**PAM and Prevention Committees Session “Burn injury beyond Survival”**

Presentation of patients’ perspective or from burns patients’ organizations from Germany (Heidi Gottwald—Paulinchen), France (Chloé Gaudens, association Burns & Smiles—Swann Sigel, volunteer at the French Burns Association). Professor Diana Harcourt, health psychologist and Co-Director of the Centre for Appearance Research (UK) told us something on ‘Online support for children with burns, and their parents’. PAM member Christine Rosch from Switzerland, presented an overview of existing Support groups in Europe and Carine Van Schie from the Dutch Burns Foundation spoke about Participative care, putting the patient in a central role in his/her care pathway.

## 6. Poster Presentations

*P.001*

**Adult Major Burn Injury Complicated by Classical Homocystinuria—The Undesired Outcome**

**Tiffanie-marie Borg, Natasha Kershaw and Niall Martin**
  St Andrew’s Centre for Plastic Surgery and Burns, National Health Service

**Objectives**: Homocysteinuria is a rare metabolic disorder with a varied clinical presentation. Complications arise due to inadequate homocysteine control and include vascular, musculoskeletal and cognitive impairment. The risk is higher in the event of concurrent burns whereby the injury itself causes further metabolic derailment through hypermetabolism and catabolism. The objective of this report is to optimise future burn care in patients with homocystinuria.
**Methodology**: We present the case of an 18-year-old homocysteinuria patient who sustained a sixty-five percent flame burn. He underwent extensive wound debridement and grafting procedures. However, despite ongoing input from the burns team, metabolic care team and a low protein diet, he developed intestinal failure, sepsis, hepatic failure, renal failure, and ultimately died. A literature search was performed using the online databases Google Scholar and Medline to identify studies pertaining to the pathogenesis, management and nutritional support required in patients sustaining major burn injuries on a background of homocysteinuria. Literature was evaluated and the care provided was compared with that of the patients managed at our centre.
**Results**: The management of metabolic complications in both homocysteinuria and burns is complex. Vigilant monitoring of homocysteine and methionine levels is essential to ascertain the severity of ongoing derangements. There is a need for improved guidelines detailing the appropriate management of burns in patients with underlying homocystinuria as existing literature is sparse.
**Conclusions**: Prompt management of homocystinuria patients sustaining burn injuries at a specialist unit together with vigilant control of homocysteine and methionine levels is essential so that outcomes can be optimised.

*P.002*

**Initial Burn Resuscitation Guided by the Parkland for mula Leads to High Fluid Volumes, Increasing Mortality and the Risk for Kidney Injury**

**Laura Lindahl, Tuomas Oksanen, Andrew Lindford and Tero Varpula**
  HUS Burn Center U2 ICU

**Objectives**: Our burn center has used the Parkland for mula (4 mL/kg/TBSA%) adjusted by physiological parameters to guide fluid resuscitation in burn patients. Our main objective was to examine fluid resuscitation in patients with major burn injury and its effect on mortality, need for renal replacement therapy (RRT) and the length of stay (LOS) in the Intensive Care Unit (ICU). Further aims were to determine which factors were associated with fluid resuscitation volumes during the first 24 h, and whether these fluid volumes had an association with the volumes infused during the next 48 h.
**Methods**: This retrospective observational study accrued patients (*n* = 46) admitted to the Helsinki Burn Center between 2016–2018 with burn injuries ≥20% TBSA. The national intensive care registry and the electronic patient record system provided data on fluid infusions, urine output, laboratory measurements, presence of inhalation injury, surgical procedures within 72 h from injury, patient demographics, need for renal replacement therapy and mortality. Patients were divided into groups based on infused fluid volumes and univariate regressions were performed to identify factors associated with fluid volumes.
**Results**: Overall, 48% of the patients received fluids more than 6 mL/kg/TBSA% during the first 24 h. In total, 35% of the patients received fluid volumes exceeding the Ivy index (250 mL/kg/d) and was associated with higher TBSA%, SOFA and SAPS scores as well as increased mortality and need for RRT. Higher lactate and lower base excess were associated with higher fluid volumes. Urine output had no association with the resuscitation volumes. Larger resuscitation volumes during the first 24 h were associated with larger fluid volumes given also during the next 48 h. Higher cumulative fluid volume in 0–72 h resulted in an increased need for RRT and higher ICU mortality.
**Conclusions**: Using the Parkland for mula and adjusting the infusion based on physiological parameters leads to over resuscitation in many of the patients. It seems that the more fluids are given during the initial resuscitation phase, the more fluids are also administered during the subsequent phase. Higher cumulative fluid volumes are associated with RRT requirements and higher mortality. We postulate that starting fluid resuscitation with a lower infusion rate could be beneficial, as it may lead to smaller cumulative fluid volumes during the first 72 h, leading to reduced mortality and kidney injury.

*P.003*

**Pain Management during a Bromelain-Based Selective Enzymatic Debridement in Paediatric and Adult Burn Patients**

**Karel Claes ^1^, Sarah Amar ^2^, Henk Hoeksema ^1^, Rachel Kornhaber ^3,4^, Stan Monstrey ^1^, Josef Haik ^3,4,5,6^, Erik Biros Erik Biros ^7^ and Moti Harats ^3,4,6^**
  ^1^  Ghent Burn Center, Ghent University Hospital
  ^2^  Department of Anesthesiology, Ghent University Hospital
  ^3^  Sackler Medical School
  ^4^  National Burns Centre, Sheba Medical Center
  ^5^  College of Health and Medicine, University of Tasmania
  ^6^  Institute for Health Research, University of Notre Dame
  ^7^  College of Medicine and Dentistry, James Cook University

**Objectives**: Pain associated with surgical or enzymatic burn wound debridement prevents many burn centres from working outside an operating theatre, creating a burden. Alternatives for general anesthesia to manage pain in burn patients treated with enzymatic debridements, such as regional anesthesia, have not been studied in detail. This study explores the different possibilities for pain management during a bedside NexoBrid™ procedure.
**Methods**: We performed a single-centre retrospective study that included 82 paediatric, adolescent, and adult patients with deep dermal and full-thickness burns treated bedside with NexoBrid™ under regional or general anesthesia. Outcome measures included pain during the NexoBrid™ procedure, the safety of the anaesthesia and the NexoBrid™ procedure, logistics of the bedside NexoBrid™ procedure, and time to wound closure.
**Results**: In total, 43 patients in the adult group (43/67, 64%) only presented with burn wounds on one upper or the one or two lower extremities. In 29 of them (29/43, 67%), a NexoBrid™ procedure was performed under regional anesthesia, which resulted in low pain levels without any adverse events. All 7 patients in the pediatric group, where only one upper or one or two lower limbs were involved (7/15, 47%), underwent a NexoBrid™ procedure performed under regional anesthesia where no adverse events were reported. In these children, the use of regional anesthesia was associated with a significant decrease in time to wound closure (average treatment effect on the treated = −22.5 days, *p* = 0.021).
**Conclusions**: This study highlights that regional anesthesia administered at the bedside should be the method of choice for pain management during NexoBrid™ procedures because often, it can be adequately and safely performed in all age groups. This approach will reduce the burden on operating theatres. A flow chart has been developed to guide pain management during a NexoBrid™ procedure.

*P.004*

**Effect of Dexmedetomidine vs. Propofol and Midazolam in Mechanically Ventilated Adult Burn ICU Patients**

**Athina Lavrentieva, Chrysavgi Giannaki, Athina Georgopoulou, Melanthi Entiaroglou, Christina Theocharidou, Evi Andreopoulou and Militsa Bitzani**
  Papanikolaou Hospital, A-ICU, Burn ICU

**Background**: Long-term sedation with midazolam or propofol in intensive care units (ICUs) has serious adverse effects. Dexmedetomidine, a 2-agonist available for ICU sedation, may improve the patients’ arousal status and enhance patient comfort.
**Objective**: To determine the effectiveness of dexmedetomidine when added to standard care in reducing the duration of mechanical ventilation and the need for additional analgesia in burn patients receiving mechanical ventilation.
**Method**: Data from patients admitted to a four bed Burn ICU from 2016 to 2018 were analyzed. Sedation level, delirium diagnosis, duration of sedation during the period of the weaning process until patients was able to sustain spontaneous breathing, duration of mechanical ventilation and requirement for additional analgesia were recorded. Patients were randomly divided into two groups of 12 (group 1-dexmedetomidine group) and 15 (group 2- usual care (midazolam and propofol sedation) group). Group 1 received dexmedetomidine 1 mcg/kg over 15 min as a loading dose, followed by 0.4–0.1 mcg/kg/h. Group 2 received midazolam 0.08 mg/kg/h and propofol 15–30 mcg/kg/min), with doses adjusted by bedside nurses to achieve target sedation goals set by clinicians according to the Richmond Agitation–Sedation Scale (RASS, scores range from −2 to 1). Delirium was assessed with the use of Confusion Assessment Methods for the ICU, CAM-ICU. All other care was at the discretion of the treating physician.
**Results**: of the 27 randomized patients (48,29 (± 20.985) median age, 49 years; 1 woman), 12 patients were included in the dexmedetomidine group and 15 patients in the usual care group.
The median duration of receipt of the trial drugs was 6.0 days for dexmedetomidine (interquartile range, 2.0 to 8.0) and 5 days for the usual care group, and the median RASS score was −1.0 (interquartile range, −3.0 to 0.0).
Dexmedetomidine did not increase ventilator-free days compared with usual care (median, 9.3 vs. 7.5 h, respectively, *p* = 0.3). Requirements for additional analgesia were higher in usual care group of patients (23.1% vs. 53.3%, *p* = 0.05). The duration of sedation during the weaning period did not differ significantly in the dexmedetomidine group vs. the usual care group (67 vs. 80 h, *p* = 0.6).
**Conclusions**: Adequate sedation level was achieved with all agents. The dexmedetomidine group required less supplemental analgesia. The addition of dexmedetomidine to standard care compared with standard care did not result in more sedation-free h during the weaning or decreased duration of mechanical ventilation in Burn ICU patients.

*P.005*

**Intracranial Pressure Incidence on Severe Burns Patient**

**Maria Notaro ^1^, Francesca Russo ^1^, Giuseppe Foreste ^1^, Gaetano Panico ^1^, Simona Cotena ^1^, Anna Lanza ^1^, Maria Rosaria Cavezza ^2^ and Romolo Villani ^1^**
  ^1^  Department of Burn Intensive Care and Cardarelli Hospital
  ^2^  Ethics Committee, ASL Na3

**Objective**: Studying the incidence of intracranical hypertension in the severe burn victim.
The shock in the burn victim is due to the combination of the hypovolemic shock and the cells shock, which is characterized by specific microvascular and hemodynamic alterations.
In burn patients, the brain is severely impaired; this correlates to high mortality.
Under normal conditions, the blood–brain barrier regulates the passage of molecules, which enter into the brain tissue; this regulation, under conditions of systemic inflammatory response, sepsis or severe burn, is severely impaired because the levels of the systemic inflammatory mediators are excessive, disproportional and correlated to unfavourable outcomes.
The increased permeability of the brain microcirculation determines the extravasation in the brain tissue of many molecules, such as albumin, exudate and inflammatory cells. These events cause neuronal damage, cerebral oedema and can determine an increase in the intracranial pressure, which is a fatal complication because its intensity and duration are linked to unfavourable outcome.
Moreover, pro-inflammatory mediators can cause the breakdown of the brain blood barrier, causing the activation of microglia cells and the astrocytes, which respond with the release of others inflammatory mediators, determining a massive neuroinflammatory response, leading to cerebral oedema.
**Method**: We made use of the measurement of the diameter of the optic nerve, by considering a cut-off of 5 mm. Using the Doppler effect, we have measured the systolic and diastolic flow velocity, the average speed and the pulsatility index of the middle cerebral artery.
**Data analysis**: The evaluation of the intracranical incidence has been studied on a total of 20 patients arrived in intensive care within 8 h from the injury, at the time 0, 24, 48, 96 h with burns that affected more than 60% of the body surface. The diameter of the optic nerve (ONSD) > 5 mm is considered of intracranial hypertension (ICP) > 20 mmHg. (PI) > 14, mean flow velocity (Vm) < 30 cm/sec, diastolic flow velocity (Vd) < 20 cm/s are considered to be pathological pulsatility indexes. The combination of at least two pathological values out of the three has been considered highly suggestive of endocranial hypertension.
**Conclusions**: Collected data showed that in any patient has been found a significant alteration of the studied parameters, suggesting that, even in the event of an increase in inflammatory status in severe burn patients, the intracranial pressure is still at physiological levels.

*P.006*

**Dotting the ‘Eyes’ and Crossing the Ts’. An Audit of Ophthalmic Assessment and Management, of Patients Admitted to a Tertiary Burns Centre**

**Christopher Van Wyk, Russel Emammdee, Dilshan Arawwawala, Anjalee Prasad and Alexia Campbell-Cherian**
  Saint Andrew’s Burns Unit

**Objectives**: Ocular burns represent 7–18% of eye injuries seen in Emergency Departments, 84% are chemical burns and thermal burns account for 16% of cases. 15–20% of patients with facial burns exhibit ocular injury. Medical skin loss (e.g., TENS) can lead to significant eye injuries. Given the potential for reduced visual acuity, the impact on quality of life and psychological morbidity, early detection through examination and timely ophthalmology review with appropriate management is critical.
This audit examines adherence to local guidelines, focusing on initial examination and subsequent ophthalmology input.
**Method**: This audit, approved by our local audit committee, included all Burns ICU patients, admitted during 2021. These were identified from our electronic clinical information system. Patients were excluded if admitted for non-acute burns or identified for immediate palliative care. The criteria for high-risk injury included any patient engulfed in a flame burn; blast injury; exposure to liquid or vapourised chemical agent; a significant medical skin loss condition; or with facial burns.
**Data collected included**:
  1. Patient demographics;
  2. Burn injury or medical skin loss details;
  3. Record of eye examination at admission;
  4. Time to ophthalmology review (if indicated) and commencement of treatment.
**Results**: In total, 47 patients (40 adults and seven children; 30 males and 17 females) were identified. The median total body surface area burn/skin loss was 33% (range 1 to 90%). Mechanism of injury included: flash/flame (32), scald (7), medical skin loss (4), other (4).
40 patients fulfilled our criteria for high-risk of eye injury, warranting for mal eye examination. 18 (45%) had a documented eye examination on admission. Nine were not examined due to eye oedema. Documentation was absent or ambiguous in 10 cases.
Only 13 ophthalmology referrals were documented. A similar number (but not all the same patients) were reviewed by ophthalmology at (median) 33 h post-admission.
Following ophthalmic review, nine patients were prescribed ocular chloramphenicol; all were prescribed supportive eye care (e.g., artificial tears).
**Conclusions and recommendations**: Local guidelines promote a reliance on specialist ophthalmological review, which is often not immediately available and may be unnecessary for most cases. We recommend the creation of a burns-specific decision tree, based on the internationally recognized Roper-Hall classification with protocolized initial treatment, initiated by the resident team, for eye injuries identified as low risk. We anticipate this should reduce the time to ophthalmic treatment and better utilize the on-call ophthalmology service.

**References**
  1. Merle, H.; Gerard, M.; Schrage, N. Ocular burns. *J. Français D’ophtalmol.* **2008**, *31*, 723–734.
  2. Roper-Hall, M.J. Thermal and chemical burns. *Trans. Ophthalmol. Soc. UK* **1965**, *85*, 631–653.

*P.007*

**Preliminary Results of Weaning Using High Flow Nasal Cannula in Severe Burns Patients**

**Crescenzo Sala ^1^, Francesco Coletta ^1^, Antonio Tomasello ^1^, Giuseppe Foreste ^1^, Anna Lanza ^1^, Gaetano Panico ^1^, Mario Brita ^1^, Francesca Schettino ^1^, Elena Santoriello ^1^, Rossella Pirolli ^2^ and Romolo Villani ^1^**
  ^1^  Aorn A. Cardarelli
  ^2^  ASL CE

**Objective**: In combination with skin burns, inhalation injuries increase the incidence of pulmonary complications and require adequate and timely airway management. The weaning phase from mechanical ventilation, followed by extubation, is often carried out through non-invasive ventilation devices (NIV, CPAP, Helmet).
The presence of severe injuries to the patient’s back and/or severe burns localized to head and neck areas make the adoption of weaning devices impracticable, impoverishing the therapeutic devices, which are uncomfortable and often painful in patients with head and neck II–III-degree burns. The high-flow nasal cannula (HFNC) represents a suitable device for weaning, and its use has been shown to significantly reduce post-extubation respiratory failure, decrease respiratory rate, and increase PaO_2_ safely and during planned extubation.
**Methods**: This cross-sectional observational study analyzed patients from the Burn Intensive Care Unit (BICU) of the Cardarelli Hospital. The preliminary results presented in this abstract refer to a period of 6 months, between January 2021 and June 2021, selecting patients affected by severe flame injuries (>30% of total body surface area, TBSA) involving the head and neck district, that underwent invasive mechanical ventilation. for all patients, clinical data, comorbidities, BMI, biochemical blood analysis, gas concentrations in arterial blood and modalities of ventilatory assistance were collected.
The data obtained were organized in an electronic database and differences in P/F values before and after HFNC treatment were tested, according to the normal distribution, by the parametric paired Student’s *t*-test using RStudio.
**Results**: In total, 7 patients (4M, 3F) were enrolled in this study. The average duration of mechanical ventilation was 10.41 (Standard Deviation, SD: 1.31 days), the Baseline PaO_2_/FiO_2_ at the intubation was 142.66 ± 38.10. At the time of extubation, shortly before starting treatment with HFNC, the PaO_2_/FiO_2_ was 292.58 ± 41.25.
After 48 h of oxygen therapy, patients showed a remarkable gain of PaO_2_/FiO_2_ compared with the beginning of therapy, with a mean of 379.33 ± 31.56 (+86.75; *p* = 0.00000219; 95% CI: 65.43–108.06).
The arterial blood lactate concentration did not significantly increase under HFNC therapy. HFNC improved patient comfort after extubation, reducing the discomfort linked to interface devices and demonstrating a good tolerance of respiratory mechanics and improving PaO_2_/FiO_2_ after extubation.
**Conclusions**: The study proposed a new approach to weaning and administering O_2_ in patients with severe burns who cannot use full-face interface devices. The use of HFNC can be a reasonable therapeutic opportunity for weaning from ventilatory support.

*P.008*

**Establishment of a Hypertrophic Scar Model and the Role of Inflammatory Processes in the Duroc Pig**

**Sebastian Nischwitz ^1^, Marlies Schellnegger ^1,2^, Julia Fink ^2^, Hanna Luze ^1^, Vladimir Bubalo ^1^, Petra Kotzbeck ^1,2^, Thomas Birngruber ^2^, Stephan Spendel ^1^ and Lars-Peter Kamolz ^1,2^**
  ^1^  Medical University Graz
  ^2^  Joanneum Research for schungsgesellschaft mbH

**Background**: Despite great progress in the treatment of burns, hypertrophic scars are still a major complication, causing great psychological and physical morbidity for those affected. Although a widespread consensus deems inflammatory processes during wound healing (partly) responsible for the development of hypertrophic scars, the exact pathogenesis is still unknown. Accordingly, there are a number of possible therapeutic options, most of which have little evidence. Standardized models are essential for further investigation of pathogenesis and development of targeted therapeutics. While in vitro models cannot represent the complexity of wound healing, in vivo models also present a variety of challenges, and thus no standardized model for scar research has been established to date. The aim of this study was to establish a standardized model of hypertrophic scar in the Duroc pig.
**Methods**: In total, 22 standardized burn and 22 full-thickness skin wounds were placed on the backs of 6 Duroc pigs, whereas half of both had a prolonged inflammation with resiquimod induced. A modified Vancouver Scar Scale, hyperspectral photography and qPCR, and histology from biopsies were performed at defined time points to examine the different processes in the resulting wounds and scars.
**Results**: The burn and full-thickness skin wounds that underwent prolonged inflammation by resiquimod had a significantly higher Scar Score than the untreated wounds as early as 77 days. Differences in native burn and full-thickness skin wounds were not significant until 105 days. Differences in wound oxygenation and gene expression appeared at similar time points.
**Conclusions**: According to our experiments, the use of resiquimod is suitable for provoking an earlier-occurring hypertrophic scar. Differences in wound types during scar maturation support the role of inflammatory processes. Further experiments to optimize scar for mation by re-inflammation could create an even more reliable model for scar research.

*P.009*

**First Measurements of the Influence of pH and Polylactide Membrane (Suprathel^®^) on the Healing of Burn Wounds**

**Matthias Rapp, Herbert Haller, Robert Schappacher, Frank Sander, Lars Peter Kamolz and Ulrich C. Liener**
  Clinic for Orthopedics, Trauma Surgery and Sports Traumatology—Burn Center, Marienhospital, Stuttgart

**Objectives**: The physiological “acid mantle” of the skin has pH values of 4–6, depending on the location and age of the person, and is of essential importance for its complete, functional integrity, as well as its resistance to noxae and bacterial colonization. In acute injury to the skin, the normal pH shifts in the alkaline direction ≥8. during healing and repair of the intact stratum corneum, the wound returns to a more neutral or even acidic pH.
In the literature, optimal pH values for vitality, proliferation, migration and adhesion to the wound bed for fibroblasts and keratinocytes are described as between 7.5 and 8.5. The optimal pH value of a chronic wound prior to split-skin transplantation is 7.2–7.5, the optimal pH value for preventing bacterial colonization of the wound is between pH 7 and 7.5.
Due to the degradation of polylactide membranes such as Suprathel^®^, the release of lactate causes the alkaline pH values to shift towards a pH value between 7 and 8 that is more optimal for wound healing.
**Methods**: The pH value of fresh burn wounds of different ages and after they had been covered with a polylactide membrane (Suprathel^®^) was determined using commercially available litmus test strips. Depending on the pH value, the litmus paper changes color from red (acidic) to black (alkaline). The litmus test strips were moistened with wound fluid or placed on a moist wound bed. The discoloration of the test strips was compared with a color scale and the pH value was estimated using the color scale.
**Results**: The assessment of the pH values of burn wounds using litmus test strips showed a pH value in the wound secretion of freshly opened blisters between 8 and 9. The pH value was between 9 and 10on the moist wound bed of newly opened blisters and on the still-weeping wound bed of blisters a few days old. The measurement of the pH value on the non-epithelialised, weeping wound bed after 8 days yielded a value between 8 and 9.
**Conclusions**: Shifting the alkaline pH values of recent burn wounds towards a pH value between 7 and 8, which is more optimal for wound healing, can accelerate healing and restore the full, functional integrity of the skin. The release of lactate through the degradation of polylactide membranes like Suprathel^®^ could accelerate this process.

*P.010*

**Burn-Induced Inflammation: Systematic Review and Meta-Analysis of the Local and Systemic Immune Response in Animal Models**

**Patrick Mulder ^1,2^, Hans Koenen ^2^, Marcel Vlig ^1^, Irma Joosten ^2^, Rob de Vries ^3^, Carlijn Hooijmans ^3^ and Bouke Boekema ^1^**
  ^1^  Association of Dutch Burn Centres
  ^2^  Laboratory of Medical Immunology, Radboudumc
  ^3^  SYRCLE, Department of Healthy Evidence, Radboudumc

As burn injuries are often followed by a derailed immune response and excessive inflammation, a thorough understanding of the cellular and soluble reactions is key to prevent secondary complications. Human studies are limited by the absence of baseline values, heterogeneity among cases and restrictions in (the timing of) blood and wound sampling. Animal experiments, executed in controlled and standardized settings, can improve our understanding of the mechanisms underlying the burn-induced immune response in humans.
Data on immune cells and factors that are involved in immediate and long-term effects, in both wound tissue and circulation, were extracted from more than 300 articles. Using meta-analyses, this review displays the temporal dynamics of immune cells and factors in the post-burn immune response. Furthermore, the risk of bias and differences based on TBSA, animal species, burning agent, age and sex were explored in subgroup analyses.
Peripheral blood neutrophil and monocyte numbers increased directly after burns, whereas thrombocyte numbers increased near the end of the first week. Lymphocyte numbers, however, were decreased for at least two weeks. In burn wound tissue, neutrophil and macrophage numbers accumulated during the first three weeks. Burns also altered cellular functions as we found increased migratory potential of leukocytes, impaired antibacterial activity of neutrophils and enhanced inflammatory mediator production by macrophages. Neutrophil surges were positively associated with burn size and were highest in rats. Analyses on the soluble immune factors in blood and burn tissue are ongoing.
Altogether, this comprehensive overview of the temporal immune cell dynamics shows that unlike normal wound healing, burn injury induces a long-lasting inflammatory response. It provides a fundamental research basis to improve experimental set-ups, burn care and outcome.

*P.011*

**Mitochondrial Dysfunction in Extensive Burns**

**Magdaléna Švecová ^1^, Bohumil Bakalář ^2^, Tomáš Urban ^3^ and Robert Zajíček ^1^**
  ^1^  Department of Burn Medicine, 3rd Faculty of Medicine, Charles University and Královské Vinohrady University Hospital
  ^2^  Department of Anaesthesiology and Resuscitation, 3rd Faculty of Medicine, Charles University and Královské Vinohrady University Hospital
  ^3^  3rd Faculty of Medicine, Charles University

**Introduction**: Mitochondrial dysfunction is one of the main pathophysiological mechanisms of multiorgan dysfunction in critically ill patients. Mitochondria are cellular organelles in which the oxidation of macronutrients occurs, and the for mation of ATP takes place. Mitochondrial dysfunction is associated with the insulin resistance (IR) as well as increased production of reduced NADPH, and thus increased oxidative stress and the for mation of reactive oxygen species (ROS) and nitrogen species (RNS). ROS and RNS directly damage DNA, structural proteins, and lipids; they act indirectly via intracellular stress-induced systems which leads to the production of proinflammatory cytokines. Burns have been shown to reduce mitochondrial oxidative phosphorylation and significantly reduce ATP production. As found in 69 pediatric patients with burns, the respiratory capacity of mitochondria in skeletal muscle remains significantly reduced for 1 year following the injury; mitochondrial dysfunction in adult burned patients has been reported sporadically.
**Objectives**: Determine the extent of mitochondrial dysfunction in adults with severe burn injury and the dynamics of this dysfunction during the treatment process. Severe injury being defined as an injury with at least 20% of total body surface area (TBSA) being second- and third-degree burns.
**Methods**: Prospective intervention non-randomized clinical study. Inclusion criteria: age >18 years, both sexes, one signed informed consent, second- and three-degree burns on at least 20% TBSA. Exclusion criteria: age <18 years, burns <20% TBSA, injury older than 3 days on admission to hospital, ongoing infections at the time of admission. In enrolled patients, a muscle sample from the quadriceps femoris will be taken on days 10, 25, and 40 after the injury and examined on an Oxygraph O2K respirometer for high-resolution mitochondrial respirometry. On the aforementioned days, the subjects will be insulin clamped to determine the degree of IR and indirect calorimetry will be measured to determine energy expenditure (Q-NRC +). These results will be related to appropriate clinical status indicators (SOFA score) and plasma markers of metabolism.
**Results**: In total, 11 patients have been examined. The metabolic results are presented and discussed during the presentation.
**Conclusions**: The dynamics of mitochondrial dysfunction are not yet known. The results of mitochondrial functions presented in skeletal muscle in 11 patients with severe burns on days 10, 25, and 40 after the injury document these changes.

*P.012*

**Characterisation of Neutrophil-Derived Cell Free DNA (cf-DNA) in Burns and Trauma**

**Ali Asiri, Jon Hazeldine, Janet Lord, Naiem Moiemen and Paul Harrison**
  University of Birmingham

**Objectives**: Increased plasma levels of cell free DNA (cf-DNA) are observed in patients after severe thermal and traumatic injuries. The majority of cf-DNA is generated from neutrophil extracellular traps (NETs) and not only implicated in the pathophysiology of multiple organ failure (MOF) but may provide an early biomarker for sepsis (Hampson et al., Annals of Surgery, 2017). DNAse degrades NETs into nucleosomes with ~150 base pairs (bp) of DNA wrapped around a histone core. Recently, large circulating chromatin fibres have also been observed in burns (Otawara et al., Scientific Reports, 2018). Here, we measured the size of cf-DNA in burns and trauma patients to understand the post-injury dynamics of NET-cf-DNA generation and breakdown.
**Methods**: Cf-DNA concentrations were measured in plasma samples obtained from severe burns (>15% TBSA) and traumatically injured (injury severity score > 8) patients across a timescale of days 1–28 and ≤1–72 h, respectively. Concentrations were compared with those measured in plasma samples from healthy controls. DNA was also extracted from plasma and analysed by the high sensitivity Agilent 2100 Bioanalyzer to determine DNA size (bp). As an in vitro control, DNA was also measured from purified NETs generated from neutrophils stimulated with 50 nM PMA for 4 h and digested with Dnase, Mnase or Dnase 1L3.
**Results**: Unresolved high molecular weight DNA was detected in the acute post-burn phase (days 1–4), which was digested down to nucleosome bands at ~150 (bp) at later time-points (days 6–14) that persisted up to 28 days. The density of nucleosomes correlated with cf-DNA levels. Healthy donor plasma, with low cf-DNA levels, contains no nucleosome bands. A ~150 bp nucleosome band was detected in trauma patient plasma at all sampling time-points. In samples acquired 1 h post-injury, higher molecular weight nucleosome oligomers (i.e., ~300 and 450 bp) were also detected. Dnase 1L3 and Mnase digestion of NETs generated nucleosome bands in vitro, although DNAse-1 completely digests remnant nucleosomes.
**Conclusions**: This study demonstrates the nature of circulating NET-derived DNA fragments in post-injury burn and trauma samples with a predominant ~150 (bp) nucleosome band apparent at most time points which correlates with cf-DNA levels. Ultra-early trauma samples (<1 h after injury) also contain nucleosome oligomers. In vitro digestion of NETs confirms the role of Dnases in breaking down NET-derived chromatin into nucleosomes. These studies will help elucidate the role of NET-derived chromatin and nucleosomes in the pathophysiology of DIC, MODS and sepsis.

*P.013*

**Comparison of Two Growth Supplements for the Production of Human Keratinocyte Sheets**

**Carmen Orte Cano ^1,2^, Laora Spinosi ^2^, Jean Pierre Draye ^2^, Gilbert Verbeken ^2^, Daniel De Vos ^2^, Nicolas Delmotte ^2^, Bruno Pascul ^2^, Lieke Convents ^2^, Esther O. Adeleye ^2^, Mariano Suppa ^1^, Véronique del Marmol ^1^, Anne Pierlot ^2^, Thomas Rose ^2^ and Jean Paul Pirnay ^2^**
  ^1^  Dermatology Department-Hôpital Erasme
  ^2^  MCT Lab—Queen Astrid Military Hospital

**Objectives**: The LabMCT of the Queen Astrid Military Hospital produces keratinocyte sheets used to stimulate burn wound healing. Two growth supplements (S7 and S125 from Gibco, Ghent, Belgium) were used in the culture medium for the production of human keratinocyte sheets. The aim of this study was to evaluate by means of LC-OCT and histology if the resulting sheets from keratinocyte cultures with S7 or S125 were comparable.
**Methods**: Neonatal for eskin keratinocytes were cultured according to the standard operating procedure at the Queen Astrid Military Hospital in Brussels. during expansion phase, two culture mediums were prepared with either S7 or S125 growth supplements. The resulting keratinocyte sheets were cryopreserved at <−135 °C. for the LC-OCT and histological evaluation, the sheets were defrosted at room temperature and samples of about 1 cm^2^ were cut at different locations on the sheets. These samples were then blindly evaluated with LC-OCT (DAMAE MEDICAL, Paris, France; 1 µM of resolution) and histology. The criteria for evaluation were presence of holes within the sheet (dyscohesion), visualization of individual nuclei, nuclei size, and sheet thickness.
**Results**: Concerning qualitative measures, keratinocytes were well observed in the different samples and did not present dyscohesion. Keratinocytes’ nuclei could be distinguished in all samples. Their shape and size were comparable between the two different treatments (no signs of nuclear pleomorphism) except for some rare big nuclei (10 µm) that could be observed in both sheets. Regarding quantitative measures, sample thickness varied between 8 and 20 µm.
**Conclusions**: Due to LC-OCT and histology, no qualitative or quantitative difference was observed between the two treatments. To the best of our knowledge, this is the first characterization of keratinocyte sheets with LC-OCT. LC-OCT was able to evaluate the three-dimensional architecture of the sheets. We can conclude that, with the methods used, there were no observable differences between both supplement cultures.

*P.014*

**Exosomes Derived from Human Hypertrophic Scar Fibroblasts Induces Smad and TAK1 Signaling in Normal Dermal Fibroblasts**

**Cheong Hoon Seo**
  Hallym University

Post-burn hypertrophic scars are characterized by excessive accumulation of extracellular matrix secreted by fibroblasts. Exosomes are membrane lipid extracellular vesicles that play a pivotal role in cellular communication. Previous studies revealed the role of stem cell-derived exosomes in repairing damaged tissues, and also showed that cancer cell-derived exosomes could affect the disease pathogenesis. However, the functional properties of exosomes derived from hypertrophic scar fibroblasts (HTSFs) have not yet been studied extensively. In this study, we aimed to investigate whether HTSF-derived exosomes can change the fibrosis-related signaling pathways in human normal fibroblasts (HNFs). HTSFs and HNFs were isolated from human hypertrophic scar tissues. HTSFs-derived exosomes were extracted and treated to HNFs. Reverse transcription-quantitative polymerase chain reaction and Western blotting were used to detect mRNA and protein expression, respectively, and cell proliferation and mobility were also assessed. Exosome treatment markedly increased cell proliferation and migration, and induced small mother against decapentaplegic (SMAD) signaling by increasing the levels of phosphorylated SMAD2 and SMAD1/5/8. The levels of TAK1 signaling components were also increased after exosome treatment to HNFs, including phosphorylated TAK1, p38, ERK, and JNK. HTSFs-derived exosomes further induced the epithelial- mesenchymal transition by decreasing the expression level of E-cadherin and increasing the expression levels of N-cadherin and vimentin. Consequently, the expression levels of fibronectin, type Ⅰ collagen, and type Ⅲ collagen were increased. Our results demonstrate the fibrotic property of HTSFs-derived exosomes, which suggests a potential functional role in hypertrophic scar development and a new therapeutic target.

*P.015*

**Evaluation by LC-OCT of Human Skin Allografts before and after the Enzymatic Removal of the Epidermis**

**Carmen Orte Cano ^1,2^, Jean Pierre Draye ^1^, Gilbert Verbeken ^1^, Daniel De Vos ^1^, Nicolas Delmotte ^1^, Esther O. Adeleye ^1^, Lieke Convents ^1^, Laora Spinosi ^1^, Bruno Pascual ^1^, Mariano Suppa ^2^, Anne Pierlot ^1^, Véronique del Marmol ^2^, Thomas Rose ^1^ and Jean Paul Pirnay ^1^**
  ^1^  MCT Lab—Queen Astrid Military Hospital
  ^2^  Dermatology Department—Erasmus Hospital

**Objectives**: To characterize by means of line-field confocal optical coherence tomography (LC-OCT) the alterations in the collagen and elastic fibers in the dermis after enzymatic removal of the epidermis of skin allografts, using dispase II.
**Methods**: Cryopreserved human skin samples stored in the Queen Astrid Military Hospital Biobank were thawed in phosphate-buffered saline. Samples were then incubated (24 h at 4 °C + 2 h at 37 °C) with and without dispase II (2.5 U/mL). The epidermis of the samples treated with dispase was carefully removed, and the dermis was washed with phosphate-buffered saline. The samples of both treatments were then evaluated either by LC-OCT imaging or histology. Concerning histology methods, sections of 5 μm were obtained and stained with H&E, orcein and Masson’s trichrome. Slices were analyzed by a pathologist for: presence/absence of the epidermis, characteristics of elastic fibers, collagen and blood vessels.
**Results**: Before treatment with dispase II, epidermis and dermal epidermal junction (DEJ) were well observed with LC-OCT in all samples. Blood vessels were present and preserved in the dermis. The reticular dermis showed a mesh of homogeneous and continuous fibers. after treatment with dispase II, the epidermis was not present. Papillae could be distinguished in the horizontal sections and appeared collapsed compared with pre-treated skin. Vascular spaces within the papillae were preserved and clearly visible. In the dermis, fibers appeared stretched and discontinuous, giving a feeling of chaos.
**Conclusions**: The effects of dispase II on a human donor skin could be characterized by LC-OCT. These findings were comparable to those seen with histology and are similar to those obtained with other imaging techniques (RCM and HD-OCT). This description may contribute to a better understanding of the visualisation of the skin.

*P.016*

**Experimental Study of the Effectiveness of Different Treatment Methods in a Rat Model of Superficial Partial-Thickness Burn Injury**

**Alexandra Csenkey, Emma Hargitai, Eszter Pákai, Béla Kajtár, Lívia Vida, Aba Lőrincz, András Garami and Józsa Gergő**
  ^1^  Department of Thermophysiology, Institute for Translational Medicine, University of Pécs
  ^2^  Department of Pathology, University of Pécs
  ^3^  Department of Paediatrics, Division of Paediatric Surgery, Traumatology, Urology and Paediatric Otolaringology, University of Pécs
  ^4^  1st Department of Medicine, University of Pécs

**Background**: There are several options available for conservative treatment of partial-thickness burns; however, reliable, affordable, and easily obtainable animal testing models are hard to find for the comparison of the different treatment methods. We aimed at developing a preclinical testing model and at comparing four treatment methods of superficial partial-thickness burns.
**Methods**: Burn injury was induced in 90 adult male Wistar rats by placing the 130 °C hot tip of a commercially obtainable soldering device for 30 s on the clipped skin of the interscapular region at a steady pressure. Skin histology was studied on days 5, 10, and 22 after the induction of the burn injury, on which days, respectively, the ratio of the not epithelialized wound (%), the extent of re-epithelialization (score), and the scar thickness (µm) were assessed. We compared silver-sulfadiazine cream, zinc-hyaluronic acid gel, silver foam dressing, and the combination of zinc-hyaluronic acid gel with a silver foam dressing.
**Results**: On day 5, we confirmed that superficial partial-thickness burn injury was induced in the rats. The zinc-hyaluronic acid gel and the combination treatment resulted in markedly smaller ratio of the not epithelialized area (29 ± 10% and 28 ± 13%, respectively) than silver-sulfadiazine cream (69 ± 4%; *p* < 0.01). On day 10, the extent of re-epithelialization was the lowest (~0.2) in the silver-sulfadiazine cream group, while the other 3 treatments performed significantly better. The combination treatment led to the maximal score of 2 in all rats, which was higher than in the other three treatment groups. On day 22, the scar thickness was the smallest in the combination treatment group (560 ± 42 µm), which was significantly less than in the silver–sulfadiazine cream group (712 ± 38 µm; *p* < 0.05).
**Conclusions**: We designed and histologically confirmed a reproducible method for induction of superficial partial-thickness burns for preclinical testing. The combination of zinc-hyaluronic acid gel with silver foam dressing was the most effective among the studied treatment options in our model.

*P.017*

**The Effect of the Arginine-Carnitine Drug Usage on Immunological Parameters and Indicators of Endothelial Dysfunction in Patients with Burns**

**Oleksandra Lynnyk ^1,2^, Heorgii Kozynets ^1,2^, Oksana Osadcha ^2^ and Olha Kovalenko ^3^**
  ^1^  Shupyk National Healthcare University of Ukraine
  ^2^  Institute of Haematology and Transfusiology of NAMS of Ukraine
  ^3^  Bogomolets National Medical University

Thermal injury disrupts the immune system homeostasis, leads to the for mation of severe hemodynamic disorders, cytokines release, systemic inflammatory response syndrome development, dystrophic processes occurrence, organs and systems dysfunction. One of the leading mechanisms for disorders development is systemic damage to the vascular endothelium. Endothelial dysfunction plays a substantial contributory role in the pathogenesis of burn wound healing and holds potential as a target for therapeutic intervention.
The objective is to study the effect of the combined arginine-carnitine drug (CACD) on some immunological parameters and indicators of endothelial dysfunction in patients with burns.
**Methods**: Studies were conducted in the acute period of a burn disease: from 2–3rd days, from 7–8 days and from 13–14 days. The use of CACD was carried out from 2–3 days after the burn injury for 5 days, intravenously in a volume of 100 mL 1 time per day.
The contents of such markers of endothelial dysfunction as endothelin-1 (ED-1) and homocysteine in blood and wounds and the contents of pro- and anti-inflammatory cytokines as interleykin-1 (IL-1), IL-6, IL-10 and TNF-α in blood were investigated.
After a course of CACD usage, the tendency to pro-inflammatory cytokines (IL-1, IL-6) levels decrease in the main group is determined, while in the comparison group, there is a tendency to IL-1 and IL-6 levels. On the 13–14th day after the burn in the main group, a significant increase in the anti-inflammatory IL-10 level compared with this indicator in the comparison group. A tendency to decrease TNF-α levels in the main group is more pronounced than in the comparison group.
After a course of CACD use, there is a decrease in peripheral blood ED-1 levels and a significant decrease in wound ED-1 (*p* < 0.001), significant decrease in homocysteine levels in peripheral blood and wound on the 13–14th day (*p* < 0.001).
**Conclusions**: As a result of our research, we found that combined arginine–carnitine drug usage in the complex treatment in the early period of burn disease and in a burn wound healing promotes reduction in inflammatory reaction manifestations and reduces the degree of endothelial dysfunction.

*P.018*

**The Impact of a Burns Facility on Inpatient Repatriated Patients with Significant Burns**

**Ardit Begaj and Reena Agarwal**
  Leicester Royal Infirmary

University Hospitals of Leicester (UHL) is a burns facility and is part of the Midlands burn care network. after the initial treatment at a burns unit in Leicestershire, patients are repatriated to UHL.
The aim of this audit was to evaluate the number of inpatient burn repatriations at UHL in the past 7 years. This audit aims to outline the care a facility can provide in the ongoing management of burn treatment.
Data was collected from international burn injury database (IBID) to determine the number of patients that were repatriated to UHL between 2014 and 2021 and the care they required.
During the past 7 years, 30 burn patients (18 male and 12 female) were repatriated for inpatient care to UHL. The median age of the patients was 58 [20–84]. The median percentage of the burn was 9% [1–60]. The length of stay in the burns unit was 14 days [3–88] followed by a median of 8 days at UHL [1–44]. Overall, 28 patients required physio and OT input, 11 required psychological input and only 3 patients required further surgery due to unhealed burns. The median number of dressing clinic appointments post discharge was three [1–31].
In conclusion, repatriated burns can require equally substantial care at a facility level. Some of these patients require significant input from an MDT setting and UHL staff have been trained to manage significant burns after repatriation. This is also due to UHL having an ECMO centre which aids in further training of staff. This audit does not look at the majority of repatriations which include dressing follow up only.

*P.019*

**The Reimplementation and ‘New Rise’ of NexoBrid at the Helsinki Burn Centre**

**Charlene Pivat, Saara Laine and Andrew Lindford**
  Helsinki Burn Centre, Helsinki University Hospital

**Objectives**: NexoBrid is a bromelain-derived enzymatic debriding agent used in the treatment of serious burn injuries. It is a topically applied treatment that removes dead or damaged tissues, known as eschars, in approximately four hours while preserving the healthy tissues surrounding the wounds.
The aim herein is to describe the different steps involved in reimplementing the medical treatment, NexoBrid, at the Helsinki Burn Centre. We discuss the difficulties encountered during the first implementation of Nexobrid, and what we have learned from it. The latter being essential for the reimplementation of NexoBrid treatment in our unit.
**Methods**: after surgeons and nurses had received education on its use, NexoBrid was first introduced to our Burn Centre in 2014. At the same time, the Helsinki burn centre was also facing big changes: relocating to new facilities and uniting with a general ICU, which had not treated burn patients before, and the loss of many experienced burn nurses.
After 6 years of rebuilding and developing our Burn Centre, we are now able to provide a solid foundation for the relaunch of NexoBrid. Education of nursing staff concerning NexoBrid treatment was commenced with lectures and wound care weekly teaching as well as hands-on teaching in the clinical setting by experienced burn nurses.
**Results**: Our new start with NexoBrid began on February 2022 with a facial burn patient, when application and removal were performed by experienced burn nurses working side by side with new burn nurses.
With continuous education and experience follow-up, nurses feel more confident and more involved in burn wound care. It also gives them more responsibility and assurance regarding NexoBrid treatment and burn wound care overall. NexoBrid is all set to become a part of our standard of procedure.
**Conclusions**: Relaunching NexoBrid in our department has been a great challenge. However, via regular educational meetings and brainstorming with our multi-professional team, we are now able to tackle NexoBrid use from different angles and make it a success. A one-year follow-up period will allow us to gather experiences of Nexobrid use from both the nurses’ and surgeons’ points of view, and also evaluate patients’ outcome.

*P.020*

**Sexual Diversity: An Inclusive Vision for the Burn Units**

**Sara Guila Fidel-Kinori, Maria Sonsoles Cepeda-Diez, Antonio Bulla, Jordi Serracanta-Domenech and Joan-Pere Barret-Nerin**
  Hospital Universitario Vall d’Hebron

**Objectives**:
  − To perform a Systematic Literature Review of Guidelines for attention to Sexual Diversity in healthcare contexts.
  − Find and adapt general criteria to the specific characteristics of Burns Units.
**Methodology**:
  − A Systematic Literature Review of Guidelines for attention to Sexual Diversity in healthcare contexts were developed in Database and internet searchers.
  − A summary of the main ideas for inclusive clinical practices, allow to drafted the basic principles for health care for people with Sexual Diversity in Burns Units contexts.
**Results**: A literature search in databases (PubMed, Psycoinfo, Weboffscience) and Internet search engines (Google, Ask, Duckduckgo), resulted in more than 6500 references on the keywords: “sexual diversity”, “hospitals”, “healthcare”. No guide was found on Burn Units. Fourteen clinical guides were selected, specific according to the criteria of: hospitals, inclusive attention to sexual diversity, clinical and social guidelines.
Suggestions based on Lund & Burgess, Clinical Care Points, to reduce health disparities with sexual diversity identity patients:
  - Understand the minority stressors faced by LGTBQ+ patients and how those may affect physical and mental health. NEED: sensibilitation, learning and training.
  - Include assessments and verbal inclusive language, no discriminator to identify the health care system as inclusive and affirming NEED: to adapt clinical records in an inclusive identity, without binary, that allows patients to indicate their pronouns and preferred names.
  - Use inclusive and positive LGTBQ+-related posters/brochures/signage in the healthcare organization to signal and affirmative environment: NEED: design a campaign to facilitate confidence and safety disclose.
**Conclusions**: The considerable increase in the introduction of sexual diversity in clinical setting but not in Burn Units, is an issue that must be considered today in our organizations. Offering minimum contents for the creation, with a local and cultural perspective of inclusive guides, should be an objective for the European Units in the coming years.

*P.021*

**The Role of Dynamic and Static Risk Factors in Predicting Mortality of Severe Burn Patients: A Retrospective Single-Center Study of 398 Patients**

**Khaled Dastagir and Peter M. Vogt**
  Hannover Medical School

**Objectives**: To determine the mortality risk in severely burned patients, the ABSI score takes into account risk factors that are already present before intensive care treatment. In the course of treatment, many other factors influence the mortality risk of patients with severe burn injuries.
**Methods**: In a retrospective study, the data of 398 patients with burn injuries with more than 10% BSA 2a-4° were collected. In addition to the influence of the ABSI criteria, other influencing factors such as comorbidity, the origin and the psychosocial status of the patients as well as other factors that develop in the course of treatment were examined.
**Results**: We analyzed the impact of 68 influencing factors on the mortality of patients with severe burn injuries. In addition to the ABSI criteria, the following factors in particular had a significant impact on patient mortality:
Comorbidities such as cardiovascular disease (*p* = 0.0004). It must be mentioned that organ diseases such as renal (*p* = 0.0925), endocrine (*p* = 0.1701), gastrointestinal (*p* = 0.0653) or psychological (*p* = 0.1554) diseases had no significant influence on the mortality of the severely burned patients. Patients from South Asia and oriental origin (*p* = 0.0027) were associated with a significantly higher mortality than patients with a European background.
Dynamic risk factors that can develop over the course of treatment and affect patient survival, such as antibiotic therapy (*p* = 0.0412), transfusion of blood supplies (*p* < 0.0001), ventilation times (*p* = 0.0035), ARDS (*p* = 0.0017) or pneumonia (*p* < 0.0001), increase in SOFA score (*p* < 0.0001), increase in SIRS criteria (*p* < 0.0001).
**Conclusions**: Our results show that the mortality risk of severely burned patients changes constantly over the course of treatment. In addition to the static assessment of the patient’s survival risk, which plays a major role at the beginning of treatment, dynamic risk factors should also be given special attention during the course of treatment. With this knowledge, risk factors can be avoided and early therapy decisions that are important for the survival of the patient can be made.

*P.022*

**Predicting Elderly Mortality due to Burn Injuries Using Machine Learning**

**Zahra Haghani Dogahe and Mohammadreza Mobayen**
  Burn and Regenerative Medicine Research Center, Guilan University of Medical Sciences

**Objective**: Burn injuries are unpredictable and devastating traumas among the elderly. With all the progress made in geriatric medicine, we expect to have a growing population among the elderly soon. It is critical to measure the risk of mortality in burn patients to support them. In this study, we measured mortality risk using different algorithms of machine learning and deep learning in the geriatric population based on the epidemiologic characteristics of burn injuries.
**Method**: We used five different machine learning and one deep learning algorithm to calculate the mortality risk of burn patients. Our analysis was based on epidemiological characteristics including age, sex, marital state, occupation, location of the injury, hour, day, month, and season of the injury, total body surface area (TBSA), burn degree, burn cause, anatomical site, and past medical history. We collected data on geriatric burn patients admitted to our hospital from 2010 to 2020. Our algorithms were: Logistic Regression, Random for est Classifier, Hist Gradient Boosting Classifier, Gaussian Naive Bayes, XGBoost Classifier, and Neural Network.
**Result**: In this study, we analyzed the data of 612 admitted burn patients. We divided the data into a training set (75%) and test set (25%) and trained our models accordingly. Epidemiologic characteristics were considered features, and mortality was the label in models. Among all, Hist Gradient Boosting Classifier with an accuracy of 90.19% was the most convenient algorithm for predicting mortality in burn patients, given the epidemiologic data. We used confusion matrix, precision, recall, F1 Score, specificity, precision–recall (PR) curve, receiver operating characteristics (ROC) curve, and PR vs. ROC curve to check the performance of our models. Other models’ accuracy was: Logistic Regression: 88.8%, Random for est Classifier: 87.58%, Gaussian NB: 85.62%, XGB Classifier: 88.23%, and Neural Network: 89.5%.
**Conclusions**: This study shows the role of machine learning in predicting mortality. Interpreting more data allows us to better train models and identify high-risk patients based on their epidemiologic features. As a result, we can set prevention programs and identify high-risk patients on admission.

*P.023*

**Early and Easy House Post-Surgery Management of Patients with Donor Sites Treated with Cryopreserved Cultured Epidermal Allograft (Epifast^®^)**

**Maria Zulema Cantú-Cantú, Ignacio A. Ricaud-Vélez and Paloma Vázquez-Balboa**
  Hospital Pediátrico de Tacubaya

**Objective**: Determinate the hospital discharge post-surgery time (days) in patients who required autografts and the donor site was covered with Cryopreserved Cultured Epidermal Allograft (CCEA) epifast^®^; also evaluated the post-surgery ambulatory house handling.
**Methodology**: Prospective, descriptive, and observational study in burn paediatric patients. 42 patients were surgically intervened from 1–31 January 2020 and their autograft donor site were treated with cryopreserved cultured epidermal allograft. From 42 patients, 10 were randomly selected and an inquest were performed to their families (mainly parents).
**Results**: after the analysis of the results, we found that 90% of the inquest were discharged in the first 5 post surgery days. To answer the question about the post-surgery handled, 90% said that it was easy; regarding the CCEA take-off, 80% answered that was easy. Neither patient presented bleeding or infection.
**Conclusions**: In conclusion the autograft donor site management with Cryopreserved Cultured Epidermal Allograft, epifast^®^, allow the early hospital discharge (in the first five post-surgery) days and greatly facilitates the patient ambulatory handled at home, altogether help to reduce the burn patient treatment costs.

*P.024*

**A 5-Year Study on Epidemiological Characteristics of Electrical Burns in Lithuania**

**Viljamas Sipavičius ^1^, Monika Rimdeikaitė ^2^ and Rytis Rimdeika ^3^**
  ^1^  Faculty of Medicine, Lithuanian University of Health Sciences
  ^2^  Faculty of Medicine, Vilnius University
  ^3^  Hospital of Lithuanian University of Health Sciences Kaunas Clinics, Plastic and Reconstructive Surgery Clinic

**Objective**: To evaluate the epidemiological characteristics of electric burns in Lithuania over five years.
**Methods**: Data were used from the Health Information Center of the Institute of Hygiene, calculated from the information system SVEIDRA of the State Health Insurance Fund of the Ministry of Health of Lithuania. Statistical analysis of the data was performed using IBM SPSS statistics 23.0 software. Mann–Whitney U criteria were used to make comparison between the percentage distributions of electrical injuries during the study period.
**Results**: A total of 355 people were affected by electric burns in a 5-year period. In total, 270 of these patients were adults. Electric burns affected 217 men and 53 women, with a statistically significant difference between both genders (*p* < 0.05). A decreasing trend of electric burns was observed in the adult group. over 5 years, electrical burns decreased by 4.08% in the male group and by 2.23% in the female group, Figure 1.
85 children were affected by electric burns. Overall, 47 patients were boys, and 38 were girls (*p* > 0.05). A decreasing trend of electric burns was also observed in the group of children. over 5 years, electric burns decreased by 7.06% in the boy’s group and by 8.24% in the girl’s group, Figure 2.
There was a statistically significant difference (*p* < 0.05) between adults and children who were affected by electric burns, Figure 3.
**Conclusions**: during 5-year study period, a decreasing trend of electric burns was observed in Lithuania. Adults were affected by electric burns more often than children. Most of the victims affected by electric burns were men. Furthermore, electric burns are a serious public health issue. Nevertheless, these injuries can be prevented, as more attention needs to be paid to the prevention of electrical burns.

*P.025*

**A 10-Year Review of Sunburn Injuries Presenting to the Manchester Adult and Paediatric Specialist Burn Services**

**Poh Tan ^1^, Lewis Dingle ^1^, Parisha Malik ^1^ and Samantha McNally ^1,2^**
  ^1^  Burns and Paediatric Plastic Surgery Service, Royal Manchester Children’s Hospital, Oxford Road, Manchester M13 9WL, England
  ^2^  Whythemsahwe Hospital Trust

The aims of this study were to determine the burden and severity of sunburn injuries on the Manchester childrens’ burns service, including rates of admission or need for surgical intervention and to identify any temporal variation
**Methods**: An eleven-year retrospective review was performed of paediatric patients with sunburn injuries, presenting to the Royal Manchester Children’s Hospital (RMCH) between 2010 and 2020 (inclusive). Data were collected from the International Burn Injury Database (iBID), electronic patient record (EPR) and local data collection systems. The data extracted included patient demographics, sunburn characteristics and management of the burn injury including need for admission and any documented surgical interventions. A linear regression was performed to determine correlation.
**Results**: Overall, 142 paediatric patients with sunburn injuries were managed by the burns service at RMCH over the 11-year period. The majority of patients (69%, 98/142) were aged between 5 and 15 years of age. More than half of the patients had burn injuries greater than 1% TBSA (69%, 96/142, 0.1–9%),37% (53/142) of patients were admitted to the hospital. With the majority being admitted for at least one day (Length of Stay, LOS, Mode = 1, Median = 1, Range = 1–10). Most patients were treated in outpatients (<24 h hospital admission, 27% 38/142). 87% (123/142) of injuries occurred during the northern hemisphere summer months between May and August, with 51% occurring in the UK. Overseas, sunburn injuries mostly occurred in Spain and Turkey (25% and 6%, respectively). There was a correlation coefficient of 0.73 between year of injury and number of cases. When 2020 was excluded, the r2 was 0.91.
**Conclusions**: This eleven-year retrospective cohort study indicates an increasing incidence of sunburn injury. When excluding 2020 due to the pandemic, the correlation strengthens. This highlights that despite increased awareness, young patients continue to suffer significant numbers of sunburn injuries. Although not a major burden, most sunburn injuries will not present to specialist burn services; therefore, these data reflect only a fraction of paediatric sunburn injuries. There is an obvious need for enhanced public awareness campaigns regarding sun protection. This educational and preventative role of burns care services is a key service tackling both consequences of burn injuries themselves and associated risks of burn injury such as skin cancer development.

*P.026*

**Human Cadaver Model as a Training Tool for Enzymatic Burn Debridement (Nexobrid^®^): A Feasibility Study**

**Jiaxin Wen ^1^, Kirsty Johnson ^1^ and Ciaran O’Boyle ^1,2^**
  ^1^  Nottingham University Hospitals NHS Trust
  ^2^  The University of Nottingham Medical School

**Objectives**: To determine the feasibility of using a human cadaver to demonstrate enzymatic burn debridement (Nexobrid^®^) as a training aid for clinical staff.
**Methods**: An observational proof-of-concept study was carried out. A single, fresh-frozen human cadaver was used. Prior consent had been given. Burns was created by flame and scalding. Unburned control sites also had Nexobrid^®^ applied and were assessed. Nexobrid^®^ enzymatic burn debridement paste was applied to all sites in adherence to the local clinical protocol for treating burned patients. after removal of Nexobrid^®^, wounds were assessed to determine if the cadaveric issue appeared similar to what would be expected in living burned patients and whether the technique could be viable for the training of burn care staff.
**Results**: Nexobrid^®^ had a very similar effect upon burned cadaveric skin to what would have been expected in living burned skin. Both scalded and flame-burned sites of partial-thickness burn depth and full-thickness burn depth was debrided and could be clearly identified. Control sites exhibited no skin loss after Nexobrid^®^ application.
**Conclusions**: Fresh-frozen human cadaveric tissue is a valid means of provision of training in the technique of enzymatic burn debridement. This permits advanced planning of hands-on training courses in the technique. Timetabled cadaveric courses may allow training of greater numbers of students than the more direct but sporadic clinical experience in acute burns units.
In addition, the degree to which the tissues of the cadaveric model mimicked those of living patients and reacted with the Nexobrid^®^ paste was unexpected. This demonstrates that our understanding of the mechanism of action of Nexobrid^®^ at a molecular level is incomplete.

*P.027*

**To Intubate, or Not to Intubate, That Is the Question: Nexobrid Treatment for Burn Injuries in Patients with Chronic Obstructive Pulmonary Disease and Home Oxygen Therapy**

**Marc Daniels, Jennifer Schiefer, Paul Fuchs**
  Department of Plastic, Reconstructive, Hand and Burn Surgery, Hospital Cologne Merheim, University of Witten-Herdecke

**Objectives**: There is an increased risk for burn injuries associated with home oxygen therapy of patients with chronic obstructive pulmonary disease (COPD) since 10% to 50% of these patients continue to smoke. Enzymatic eschar removal of facial burns is gaining popularity, but intubation of this specific patient group often leads to prolonged weaning and can require tracheostomy. This study dealt with the question if enzymatic debridement in these patients can also be performed in analgosedation.
**Methods**: A retrospective analysis of all patients with burn injuries associated with home oxygen use and COPD that were admitted to the study clinic, as well as a selective review of the literature regarding burn trauma associated with home oxygen use in patients with COPD, were performed.
**Results**: In the literature, 1746 patients with burns associated with home oxygen use are described, but none of them received enzymatic debridement. Between 2017 and 2020, a total of 17 COPD patients with home oxygen-associated burns were treated at the study clinic. All three patients in this study with facial full-thickness burn injuries received enzymatic debridement. The mortality rate in this cohort was 17.6% (3/17). In total, 6 patients (35.3%) required intubation and were ventilated for a median of 182 h (25–314 h). Overall, 5 patients (29.4%) required tracheostomy.
**Conclusions**: To date, there is limited experience performing regional anesthesia debridement in patients with COPD. This is the first study describing the use of enzymatic debridement in patients with COPD and home oxygen therapy. We could confirm other studies that intubation of these patients leads to prolonged ventilation hours and increases the probability for poor prognosis. Enzymatic debridement of these patients is well practicable in analgosedation, avoiding intubation and prolonged weaning or tracheostomy.

*P.028*

**Enzymatic Debridement as Standard of Care for the Treatment of Functional and Aesthetic Especially Important Body Areas-Experiences after More Than 300 Treatments**

**Jennifer Schiefer and Paul Christian Fuchs**
  Cologne Merheim Medical Center, Clinic of Plastic and Burn Surgery, University of Witten/Herdecke

**Aim**: after a certain skepticism in the beginning, enzymatic debridement has revolutionized traditional burn wound treatment in the last decade and become more and more popular. Nevertheless, it usually takes a while until enzymatic debridement is fully integrated in the clinical routine and becomes standard of care.
**Method**: after experience with more than 300 patients, enzymatic debridement has become the standard of care for body regions where tissue preservation has especially high priority such as hands, faces, feet and genitals in our hospital.
**Results/Discussion**: Early obstacles helped us to develop special treatment guidelines for easy integration in daily practice, with low human resources, without the necessity of an operation theater and intubation as well as a feasible post-debridement treatment protocol with a poly-lactic-acid-based synthetic skin substitute or a skin graft.
**Conclusions**: Especially in facial burns intubation can often be prevented.
Furthermore, scarring could improve considerably compared with traditional surgical debridement.

*P.029*

**Enzymatic Debridement in Scalds Is Not as Effective as in Flame Burns Regarding Necessity of Additional Surgical Debridement: A Retrospective Matched-Control Study**

**Christian Tapking, Laura Siegwart, Yannick Jost, Gabriel Hundeshagen, Dimitra Kotsougiani-Fische, Emre Gazyakan, Björn Bliesener, Ulrich Kneser and Sebastian Fischer**
  BG Unfallklinik Ludwigshafen

**Objective**: Enzymatic debridement (ED) of burn eschar became an accepted and widely used technique for acute burn wound treatment over the last few years. However, it is not exempt from failure, and recent experimental studies indicated that it may not be as efficient in scalds as in flame burns. This matched case–control study aimed to evaluate the efficacy of ED defined as the absence of additional surgical debridement in scald and flame burns representing the two most common mechanisms of burn.
**Methods**: Patients that were admitted to the burn intensive care unit between June 2017 and February 2021 and received ED within the first 72 h after scald and flame burn were included. Patients with scald burns were matched regarding age, sex and per cent total body surface area (%TBSA) burned in a 1:2 ratio with patients presenting with flame burns.
**Results**: In total, 18 patients with scald burns were matched to 36 patients with flame burns. after matching, both groups were similar in terms of age (flame burns 44.5 ± 21.1 years vs. scald 41.8 ± 22.6 years, *p* = 0.666), and%TBSA burned (11.0 ± 8.2% vs. 10.6 ± 9.6%, *p* = 0.851). Patients with scald burns significantly more often needed further surgical debridement compared with controls (scald 16 (88.9%) vs. flame 19 (52.8%), *p* = 0.016). Length of stay per%TBSA was significantly longer in scald burns (scald 7.8 ± 9.2 days vs. flame 3.7 ± 3.8, *p* = 0.013).
**Conclusions**: This study is the first to compare the clinical efficacy of ED in different burn mechanisms. It indicates that ED may not be as effective in scalds as in flame burns. It was shown that patients with scalds and subsequent ED more frequently needed additional surgical intervention and that the size of the transplanted area was larger compared with control. Moreover, those patients had a longer length of stay at the hospital per%TBSA burned. We believe that larger, multicenter studies would be beneficial to further evaluate the efficacy of ED in different mechanisms of burn.

*P.030*

**Enzymatic Debridement for Burn Wound Care: Interrater Reliability and Impact of Experience in Post-Intervention Therapy Decision**

**Laura Siegwart, Arne Böcker, Christoph Hirche, Sebastian Fischer and Ulrich Kneser**
  BG Trauma Center, Hand-, Plastic and Reconstructive Surgery, Microsurgery, Burn Center, University of Heidelberg

**Objectives**: Enzymatic debridement (ED) has become a reliable tool for eschar removal. for tunately, ED application is simple. However, wound bed evaluation and therapy decision post-intervention are prone to subjectivity and failure. The objective was to analyse interrater reliability (IR) and impact of experience in post-intervention wound bed evaluation and therapy decision. The authors introduce video assessment as a valuable tool for post-ED decision-making and education.
**Methods**: A video-based survey was conducted among physicians with various experiences in ED. The survey contained multiple-choice and 5-point Likert scale questions about professional status, experience in ED, confidence in post-ED wound bed evaluation and therapy decision. Overall, 15 videos of mixed pattern to full-thickness burns after removal of the enzyme complex were shown. Participants were asked to evaluate each burn wound, including bleeding pattern and consequent therapy decision. IR ≥ 80% was considered as a consensus. Responses were stratified according to participants’ experience in applying ED (<10, 10–19, 20–49, and ≥50 applications). IR was assessed by chi-square test (raw agreement [RA]; ≥80% was considered as a consensus). Participants were asked for their opinion on video as an assessment tool for post-ED wound bed evaluation, decision-making, and training.
**Results**: In total, 31 physicians from 11 burn centers participated in the survey. The overall consensus (raw agreement [RA] ≥ 80%) in post-ED wound bed evaluation and therapy decision was achieved in 20 and 40%. Subgroup analysis disclosed that physicians with high experience in ED achieved significantly more consensus in post-intervention wound bed evaluation and therapy decision compared with physicians with moderate experience (60% vs. 13.3%; *p* = 0.02 and 86.7% vs. 33.3%; *p* = 0.04). Video analysis was considered a feasible (90.3%) and beneficial (93.5%) tool for post-intervention wound bed evaluation and therapy decision, as well as being useful for training purposes (100%).
**Conclusions**: The reliability of wound bed evaluation and therapy decision is related to experience with ED application. Video analysis is deemed to be a valuable tool for ED evaluation, decision-making, and user training

*P.031*

**Infrared Thermography for the Assessment of Burns after Bromelain-Based Enzymatic Debridement**

**Javier Pérez-Rodríguez, Luis Díaz-Ojeda, Alvaro González-Miranda and José Ramon Martínez-Méndez**
  La Paz University Hospital

**Objectives**: Clinical evaluation of burn wound healing potential can be challenging. Aware of the increasing utility of thermography in burn care, we wanted to combine it with the promising results of bromelain-based enzymatic debridement (ED). The aim of this study was to explore the feasibility of a thermographic assessment of the debrided wound bed. The burn injury produces a microvascular alteration that would result in a decrease in surface temperature. We hypothesized that after complete ED, changes in skin surface temperature due to thermal injury would be reversed.
**Methods**: A prospective study was conducted. Patients undergoing Bromelain-based Enzymatic Debridement in the acute setting in our Burn Unit (2020–2021) were evaluated with thermal imaging. The exclusion criteria were: <18 years of age, >9% total burned surface area (TBSA), hypothermia, hemodynamic instability, inhalation injury, and organ failure. Two images of each burn wound were taken, before and immediately after debridement, and the difference in temperature between the burn wound and healthy skin was recorded in both assessments. The primary outcome was the difference in temperature after enzymatic debridement.
**Results**: We included 19 patients (11 women, 8 men) with 24 burns. The average burned area was 3% (1–7%) and most burns were caused by flame (75%) and located in the upper limb (91.7%). Time to Enzymatic Debridement was, on average, 7.5 h from injury.
The temperature difference between the burn wound and the healthy skin was 2.6 °C on average before the ED and 0.7 °C after the ED (*p* < 0.001). Before debridement, the mean temperature of the wound was 31.8 °C and that of healthy skin was 33.7 °C (*p* < 0.01). after debridement, the average temperatures of the burned and healthy skin were 31.9 °C and 32.6 °C (*p* > 0.05).
**Conclusions**:
  - Thermal imaging can detect burn wound changes after Bromelain-based enzymatic debridement.
  - Burn wound surface temperature changes due to the burn injury were reversed after debridement. These changes could be related to the microvascular status of the wound bed.
  - Thermography is a promising imaging modality for burn care, particularly in combination with enzymatic debridement.

*P.032*

**Enzymatic Debridement for the Prevention of Burn Induced Compartment Syndrome: Utility or Futility?**

**Laure Ruyssinck, Ignace De Decker, Henk Hoeksema, Stan Monstrey and Jozef Verbelen, Karel Claes**
  Ghent University Hospital

**Objectives**: Burn induced compartment syndrome (BICS) of the extremities or abdominal region requires escharotomy to maintain tissue viability. Until now, surgical escharotomy was the main option for prevention and treatment of BICS. However, this carries a risk of damaging anatomical structures, bleeding and infection. Enzymatic debridement with NexoBrid^®^ (EDNX) has emerged as a valuable alternative for early, selective burn eschar removal.
**Methods**: This case series includes 4 patients where, due to clinical signs of BICS, EDNX was used at a very early stage post-burn (<24 h). Our standard protocol for NexoBrid^®^ application consists of 2 h pre-soaking, 4 h NexoBrid^®^ application, 2 h post-soaking and finally application of allografts.
**Results**: Patient one (32-year-old male) had an 8.4% TBSA, third-degree, circumferential burn on his right lower leg resulting in BICS. In addition to clinical symptoms (paresthesia, delayed capillary refill, cold temperature and blue color of his foot and toes), pressure measurement revealed intra-compartment pressures of 60 mmHg. Within the first hour after NexoBrid^®^ application, refill, temperature and color normalized. Intra-compartment pressures dropped to 20 mmHg post-procedure.
Patient two (60-year-old female) had third-degree, circumferential burns on the trunk and abdomen. Because she was intubated, BICS was diagnosed based on increasing intra-abdominal and ventilation pressures. In total, 30 min after EDNX application, intra-abdominal pressures lowered to normalize by the end of the procedure. Ventilation pressures remained high due to a ventilation acquired pneumonia.
Patient three (32-year-old male) had deep second-degree circular burns on both wrists and the dorsum of the hands. Increased swelling of his hands and fingers and delayed capillary refill urged the need for NexoBrid^®^ treatment. Swelling progressively decreased and refill normalized within the first hours of Nexobrid^®^ application.
Patient four (21-year-old male) had deep second and third-degree burns on both wrists (circumferential) and hands (circumferential). A consistent increase in swelling of both hands with reduced capillary refill (nail beds) urged us to perform urgent EDNX. No additional surgical escharotomies were necessary, swelling of both wrists and hands was significantly reduced at the end of the procedure.
**Conclusions**: To the best of our knowledge, this is the first case series that aims to provide clinical evidence for safe and effective use of NexoBrid^®^ in the prevention of BICS in upper extremities, lower extremities, thoracic and abdominal regions. Surgical escharotomy was prevented in all cases.

*P.033*

**Enzymatic Debridement (Nexobrid^®^) in Burned Hands. Retrospective Review from a Burn Referral Center in Spain**

**Zhan Qiao Lin Wu, Danilo Antonio Rivas Nicolls, Antonio Bulla, Jon Ander Aguirrezabala del Río and Jordi Serracanta Domenech**
  Vall D’Hebrón University Hospital

**Objetives**: To evaluate the outcome of Nexobrid^®^ treatment on intermediate and deep burns on the hands, as well as to determine which of the intermediate-second-degree or deeper burns in the hands required surgical treatment after Nexobrid^®^ and which did not, being the largest series described in the literature until now.
**Methods**: We conducted a descriptive retrospective study of all patients who underwent enzymatic debridement for hand burns at the Vall d’Hebrón University Hospital between May 2015 and April 2020. Senior surgeons from the burns team chose the patients who were eligible to Nexobrid^®^ treatment. after debridement, the medical staff determined if the burn required conservative or surgical treatment. All information was gathered from the hospital database.
**Results**: Overall, 139 patients were included, 108 males (77.7%) and 31 females (22.3%). The average age was 42.12 years (range 17–87). In total, 76 of these patients had unilateral involvement of the hand (54.7%) and 63 bilateral involvement (45.3%); therefore, 202 hands were analyzed. Right hands were involved in 109 cases (53.96%) and left in 93 (46.04%). Burn depth was more frequently deep second-degree (122; 60.4%) followed by intermediate (71; 35.15%), third-degree (7; 3.47%) and superficial- second-degree (2; 0.99%). The most common burn etiology was flame (89; 64.03%) followed by scald (27; 19.42%). Mean TBSA burned was 15.05% (range 0.5–85%).
Surgery was performed in approximately half of the hands (99; 49%). The average healing time following Nexobrid® was 38.35 days (range 11–212). The majority of hands that underwent surgery had deep second-degree burn (61; 61.62%) following intermediate (30; 30.3%). during the follow-up, 24 hands required surgery for sequelae (11.88%), whereas 116 did not (57.43%), and 62 were lost (30.69%). Most hands (21, 87.5%) that needed surgery to correct scar sequelae were those that had required surgery after Nexobrid^®^ and only 3 (12.5%) treated with conservative care required scar correction surgery (Fisher’s test, *p* < 0.001).
**Conclusions**: Our findings suggest that enzymatic debridement reduces the number of surgeries in intermediate-deep hand burns since it allows for a more cautious approach (of the 122 burns initially classified as deep, and therefore as surgical, only 61 (50%) were operated). Furthermore, enzymatic debridement seems to lessen the need for surgery due to burn scar sequelae: of the 24 hands that underwent scar revision surgery, only 3 (12.5%) had received conservative treatment (*p* < 0.001). Nevertheless, the healing time of burned hands after enzymatic debridement is longer (whether or not surgery is required).

*P.034*

**The Impact of Nexobrid in the Treatment Course in Adults with Partial-Thickness Burns Compared with Standard of Care—A Systematic Review**

**Anja Imsirovic, Simon Booth, Baljit Dhensa and Paul J. H. Drake**
  Brighton Additionally, Sussex Medical School

**Objectives**: Burn injury causes significant morbidity, functional impairment and disfigurement. Reducing the impact of such injury has led to early surgical intervention and methods to preserve or replace dermis where possible to reduce scar contracture. Enzymatic burn wound debridement has been proposed to preserve dermis, reduce the amount of burn requiring skin grafts and reduce time to treatment. The objective of this study is to present a systematic review of the role of enzymatic debridement in burn treatment compared with standard of care (debridement and skin grafting) in adult patients with burns.
**Methods**: A systematic review of randomized controlled and case–control studies reporting and comparing healing outcomes of enzymatic debridement with the standard of care, published on Embase, Medline, PubMed, Google Scholar and Cochrane databases in the period from 1946 until August 2021 was performed. Relevant studies were extracted and analysed with the help of Review Manager Version 5.4.1, The Cochrane Collaboration, 2020. The systematic review was registered with the International prospective register of systematic reviews (PROSPERO) with registration number CRD42021283286.
**Results**: The database search identified thirteen articles that met the inclusion criteria. of these, four were systematic reviews, one a consensus document, one randomized controlled trial, five non-randomized prospective studies and two were retrospective reviews. A combined number of 441 patients were included. The average age of patients in the enzymatic debridement intervention group was 42 years and in standard of care group 48 years. Nexobrid application average time was one day. The combined mean total body treated mean surface was 6.5%. Studies investigating Nexobrid mention the combined average success of debridement of 82.8%. Overall, Nexobrid reduces time to treatment, need for surgery and shortens hospital stay.
**Conclusions**: This systematic review presents the current data supporting Nexobrid usage for enzymatic debridement of partial-thickness to deep dermal burns. Moreover, it also discusses trends and directions for further research in this field. Further randomized controlled trials or feasibility studies are warranted. The relevant areas of future research with Nexobrid use include the reduction in local inflammatory response, optimization of perfusion and drainage due to reduced post-traumatic oedema, its role in preventing compartment syndrome, dermal layer preservation, patient satisfaction perspective, role in mass casualties and systemic response after enzymatic debridement.

*P.035*

**A Novel Technology—Enzymatic Debridement—Nexobrid in Combination with Hy Tissue Micrografts**

**Panche Taskov ^1,2^, Mircea Nemes ^1^, Daniela Corodati ^1^ and Zorin Crainiceanu ^1,2^**
  ^1^  Plastic Additionally, Reconstructive Surgery Clinic—Casa Austria, Emergency County Hospital “Pius Branzeu”, Timisoara, Romania
  ^2^  University of Medicine and Pharmacy “Victor Babes”

**Objectives**: We present a novel concept consisting of enzymatic debridement—Nexobrid of deep partial-thickness and full-thickness burns in combination with Hy Tissue Micrografting technique using autologous micrografts and “smart” dressings, avoiding SOC treatment using tangential excision and split-thickness skin graft (STSG) coverage.
**Methods**: In a retrospective case study, three patients (first—85% TBSA, second—45% TBSA, third—15% TBSA) with deep partial-thickness and full-thickness burns were treated with enzymatic debridement and autologous cell therapy.
Our novel technique was applied to up to 5% TBSA.
Other injuries were treated with enzymatic debridement, surgical excision and skin grafting—split-thickness skin graft (STSG) and MEEK micrograft coverage.
**Results**: All three patients in areas treated with our new technique did not require additional surgery and coverage. In less than 2 weeks, the lesions completely epithelized.
**Conclusions**: The use of selective enzymatic debridement in combination with the tissue technique is a regenerative technique of the future, in order to reduce the need for grafting using its own donor area or allografts/xenografts currently unavailable nationally.
This technique will show its efficacy when higher standardization and experience is achieved.
We observed a reduced healing period and length of hospital stay, improvement in time to full epithelialisation; the areas treated did not require additional surgery and coverage. The elasticity, quality of and the aesthetic aspect of the scars are clearly superior to scar quality in comparison with traditional excision and skin grafting.

*P.036*

**Influence of Bromelain Based Enzymatic Debridement in Hemodynamic Evolution of the Burn Patient—Case Series**

**Silviu Marinescu, Anca Bordianu, Siramona Toma, AnaMaria Stefanescu and Andreea Badeana Carmen Giuglea**
  Bagdasar-arseni Clinical Emergency Hospital

**Introduction**: Since its approval in Europe, our Plastic and Reconstructive Department has started implementing enzymatic debridement in the protocol of care of burn patients with mixed pattern burns as an alternative of the classic surgical eschar removal. This article aims to present the impact of enzymatic debridement on the hemodynamic evolution of a series of burn patients who suffered thermal injuries by flames mechanism.
**Materials and Methods**: Between January 2019 and March 2021, 27 patients with mixed-pattern burns underwent enzymatic debridement. The trunk and the lower extremities were the main areas where the enzymatic agent was applied. Patients were treated following the European Consensus Protocol, in the early stage of the burn (between 12 and 72 h since the injury moment), but 2 particular patients underwent enzymatic debridement on more than 15% TBSA. The bromelain-based treatment was evaluated for the systemic inflammatory reaction and hemodynamic evolution.
**Results**: Overall, 25 out of 27 patients had an average of 12% TBSA application area. In particular, 2 patients had an off-label application area of 18%. We found no significant differences concerning body temperature and blood pressure during and the couple of days following the enzymatic debridement procedure. Additionally, no side effects of the debridement product were observed during and after the treatment. Except hemoglobin drop due to bleeding which was manageable in the ICU if needed and did not put patient at risk, improvement of the hemodynamics could be seen compared with the blood tests before the debridement procedure. We observed a leukocyte count drop, lactate, CRP and CK and CK-MB value drop the days following the treatment, but no significant differences of the fibrinogen values. AdditionallyMoreover, there were no major changes in the electrolyte levels before and after product application.
**Conclusions**: Bromelain-based enzymatic debridement is a safe alternative to eschar burn removal in mixed pattern burs, especially when following the Consensus Guidelines, which can bring an improvement non only on the local evolution of the condition, but also in the hemodynamic stability of the burn patient. Selective eschar removal preserves local healing resources, does not compromise the general state of the patient and copes with downsizing the inflammatory systemic response.

**Keywords**: bromelain; enzymatic debridement; hemodynamics

*P.037*

**Enzymatic Debridement in High-Risk Surgical Patients: Management and Clinical Outcomes**

**Alex Pontini, Roberto Salmaso and Bruno Azzena**
  Padova University Hospital

**Objectives**: Enzymatic debridement represents a well-recognized and widely used technique for burn wound treatment over the last years. Its role became routinary from 2019 in our acute burn treatment in selected patient, especially for those who suffered severe burn injuries in important anatomical and functional areas (hand, joint, foot) and in high-risk surgical patients.
**Methods**: We retrospectively analyzed all patient treated by enzymatic debridement in last three years in our burn unit in terms of indication, off-label treatment, histological evaluation of pre- and post-treatment areas and clinical outcomes. We also focused on treatment of high-risk surgical patient represented by elder patient with several major comorbidities or patient with coagulopathy. All these patients were considered not suitable for traditional escarectomy and have no other surgical options for burn treatment. We analyzed the outcome of burn treatment, blood needing and early and late complication.
**Results**: In high-risk surgical patients, enzymatic debridement represent and effective and saving life treatment, particularly in patients with no surgical options due to their clinical preoperatory conditions. No major complications were observed even in off-label treatment (compassionate use), blood and plasma needling were significantly decreased due to fewer bleeding complications and also registered an important surgical time reduction because the patient was prepared for wound coverage by enzymatic action.
**Conclusions**: Enzymatic debridement plays and important role as a non-surgical tool for selective and rapid eschar removal. Its role is increasing in our experience, particularly in high-risk burned patients in the general population with an even more increased age, comorbidities and multiple drug treatment that could represent contraindication for surgical escharectomy or lead to a high mortality rate.

*P.038*

**Costs and Cost-Effectiveness of Enzymatic Debridement at a Tertiary Center in Spain**

**Patricia Martin-Playa, Iker Ustarroz-Aguirre, Leire Aparicio-Elizalde, Guillermo Fermin Ibarrondo-Arzua, Paula Rodriguez-Ruiz, Borja Garcia-Lorenzo, Juan Carlos Bayon-Yusta and Juan Jose Garcia-Gutierrez**
  Cruces University Hospital

**Objectives**: Enzymatic debridement with NexoBrid^®^ has shown to have many advantages compared with surgical debridement (SOC) on second- and third-degree burns to a point that it has now become the standard of care in many centres. Although its clinical effectiveness has been proven, there is scarce literature regarding its real costs and its cost-effectiveness. With this study, we aim to show the total cost of patients treated with NexoBrid^®^ and its cost-effectiveness at our Great Burn Unit.
**Methods**: A retrospective observational cohort study was designed to compare patients treated with NexoBrid^®^ and SOC between October 2016 and April 2020. Demographic data, burn extension (TBSA) and characteristics, length of hospital stay, number of surgical procedures and number of transfusions needed per patient were recorded. Real costs of each single patient were obtained (not estimated by mean costs per department or diagnosis-related groups). Patients were contacted at least one year after discharge to respond the European Quality of Life-5 Dimensions (EQ-5D) questionnaire, only those responding to the questionnaire were included in the cost-effectiveness analysis.
**Results**: A total of 80 patients (39 patients in the SOC group and 41 in NexoBrid^®^ group) were analysed. No statistically significant differences between the subgroups were found before debridement. There was a significant decrease in the number of surgeries and transfusions needed between cohorts. Mean cost per patient treated with SOC was 44,841 EUR, and for those treated with NexoBrid^®^ was 36,190 EUR, meaning a reduction in costs per patient of 8651 EUR. Only 46 patients responded to the EQ-5D questionnaire, showing that quality-adjusted life years (QALY) in patients treated with SOC were slightly superior to those treated with NexoBrid^®^: 0.0129 (± 0.0021).
**Conclusions**: Patients treated with NexoBrid^®^ had shorter hospital stays, needed fewer transfusions, and underwent fewer surgical procedures than patients treated with SOC, this translated into a significative decrease in total costs associated with patients treated with NexoBrid^®^. Unfortunately, we could not prove a significant difference between NexoBrid^®^ and SOC regarding cost-effectiveness; we believe that further analysis with a greater number of patients should be conducted.

*P.039*

**Comparison of Health-Related Quality of Life in Burn Patients after Enzymatic Debridement (Nexobrid^®^) vs. Surgical Debridement: Our Experience at a Tertiary Center**

**Paula Rodriguez Ruiz, Patricia Martín-Playá, Leire Aparicio-Elizalde, Guillermo Ibarrondo-Arzua and Juan José García-Gutiérrez**
  Department of Plastic Surgery and Great Burns Unit, Cruces University Hospital

**Objectives**: Burn patients develop physical and psychological sequelae that have a negative impact on daily functioning and decrease their health-related quality of life. Bromelain-based enzymatic debridement (NexoBrid^®^) is a treatment option that has changed burn care. Increasing evidence has been published regarding its advantages compared with surgical debridement, which constitutes the standard of care (SOC). This study aims to determine the impact of enzymatic debridement on the health-related quality of life of burn patients.
**Methods**: A retrospective study was carried out on 80 patients admitted to the Cruces University Hospital Great Burns Unit between 2016 and 2020. Demographic data, comprising information regarding burn characteristics and type of treatment received during hospital admission, were collected. Health-related quality of life was measured using EuroQol-5D (EQ-5D) and Burn Specific Health Scale-Brief (BSH-B) scales, both in Spanish, at least one year after patient discharge from hospital.
**Results**: Patients in the NexoBrid^®^ group significantly underwent less surgical interventions, had a shorter admission time in the Great Burn Unit and a lower healthcare expenditure. Most patients (85–90%) in both treatment groups did not refer mobility or self-care problems. Only 30% experienced problems performing usual activities or suffered a moderate degree of pain, and around 20% presented a moderate degree of anxiety or depression. No statistically significant differences were found regarding the quality of life in burn patients after enzymatic debridement compared with SOC.
**Conclusions**: Measurement of health-related quality of life has become a relevant strategy to study the health status of a population and to analyze the effectiveness and the efficacy of healthcare interventions. Burn patients have a decreased health-related quality of life when compared with the general population. This difference increases in patients where a greater percentage of the body surface is affected. Bromelain-based enzymatic debridement (NexoBrid^®^) enables a statistically significant reduction in hospital admission times, number of surgical interventions and healthcare expenditure. No statistically significant differences were found between bromelain-based enzymatic debridement (NexoBrid^®^) and the SOC in the health-related quality of life scales analyzed in our study.

*P.040*

**Safety and Efficacy of NexoBrid Enzymatic Burn Debridement in Children: Results of the CIDS Multicenter RCT**

**Yaron Shoham ^1^, RP Narayan ^2^, Torsten Hannmann ^3^, Raphael Staubach ^3^, Stan Monstrey ^4^, Henk Hoeksema ^4^, Ruzsena Bene ^5^, Mohan Kakola ^6^, Yvonne Wilson ^7^, Giavonni Lewis ^8^, Jha Manoj ^9^, Shawn Larson ^10^, Serhii Tatsyuk ^11^, Oleksiy Kravtsov ^12^, Elena Hanganu ^13^, Gheorghe Horea Gozar ^14^, Guga Kashibadze ^15^, Tam Pham ^16^, Tamas Decsi ^17^, Robert Sheridan ^18^, Paul Glat ^19^, Adam Singer ^20^, Frank Sander ^21^, Bernd Hartmann ^21^ and Yehuda Ullman ^22^**
  ^1^  Soroka University Medical Center
  ^2^  ESIC Medical College & Hospital
  ^3^  Klinikum Stuttgart
  ^4^  Gent University Hospital
  ^5^  Bethesda Hospital
  ^6^  KR Hospital
  ^7^  Birmingham Children’s Hospital
  ^8^  University of Utah Burn Center
  ^9^  ABVIMS & Dr. RML Hospital
  ^10^  University of Florida
  ^11^  Odessa Regional Clinical Medical Center
  ^12^  Zaitsev V. T. Institute of General and Urgent Surgery
  ^13^  Spitalul Clinic de Urgenta
  ^14^  Spitalul Clinic de Urgenta
  ^15^  S. Khechinashvili University Hospital
  ^16^  Harborview Medical Center
  ^17^  University of Pecs
  ^18^  Shriner’s Children’s Hospital
  ^19^  St Christopher’s Hospital for Children
  ^20^  Stony Brook University
  ^21^  Unfallkrankenhaus Berlin
  ^22^  Rambam Medical Center

**Objectives**: Nexobrid^®^ (NXB) enzymatic debridement of deep burns is approved for use in adults in several regions worldwide. Clinical trial experience and off label reports point to NXB safety and efficacy in children as well. The objective of this study was to further assess the safety and efficacy of NXB in children, in efforts to support regulatory approval for its use in children.
**Methods**: In total, 145 children ≤18 years old suffering from deep thermal burns between 1% and 30% TBSA were enrolled in a multicenter, multinational, open label, randomized, controlled phase III study. In total, 72 children were randomized to debridement with NXB and 73 children to standard of care (SOC) surgical and/or non-surgical debridement methods, at the investigators’ discretion. Patients who did not achieve complete debridement after NXB application were rescued with SOC debridement methods. Wound care after achieving complete debridement was according to routine methods, at the investigators’ discretion. Patients were then followed up for >2 years. This Abstract reports the acute stage results of the study and the first year of follow-up.
**Results**: Baseline characteristics were similar between the arms. The median age was 3.4 years in the NXB arm and 3.9 years in the SOC arm. The average burn area was 7.0 ± 4.9% TBSA in the NXB arm and 6.2 ± 4.8% TBSA in the SOC arm. The study met all three primary endpoints: the median time to complete debridement was 1 day for NXB and 6 days for SOC (*p* < 0.001), the percentage of the wound area excised in order to complete debridement was 1.5% for NXB and 48% for SOC (*p* < 0.0001), and MVSS scores at 12 months were 3.83 for NXB and 4.86 for SOC (non-inferiority endpoint). Secondary endpoints demonstrated 8.3% incidence of surgical excision to complete debridement for NXB and 64.4% for SOC (*p* < 0.0001), mean debridement associated blood loss of 32 ± 284 mL for NXB and 202 ± 409 for SOC (NS), a 25.9% incidence of autografting in DPT wounds for NXB and 37.7% for SOC (*p* = 0.054), and a mean percentage area of DPT wound autografting of 15.9 ± 38.6 for NXB and 22.8 ± 43.7 for SOC (NS). Safety endpoints demonstrated a non-inferior time to complete wound closure (median 32 days for NXB, 34 days for SOC) and no significant safety issues were demonstrated during the study.
**Conclusions**: NXB was shown to be a safe and effective debridement agent in pediatric burns.

**Funding**: Funded with US Federal funds from the Office of the Assistant Secretary for Preparedness and Response, BARDA, under Contract No. HHSO100201500035C.

*P.041*

**Tips and Tricks in the Treatment of a Burn with a Large Surface and Depth Associated with an Evolving Pregnancy**

**Mihaela Pertea ^1,2^, Nadia Aladari ^1,2^, Ștefana Luca ^1,2^ and Oxana-Mădălina Grosu ^1,3^**
  ^1^  Grigore T. Popa, University of Medicine and Pharmacy
  ^2^  Department of Plastic Surgery, Reconstructive Microsurgery and Burn Unit “Sf. Spiridon” Emergency Hospital
  ^3^  Elytis Hospital Hope

**Objectives**: The purpose of the presentation is to report the efforts and the combination of techniques for the treatment of a deep burn with an area of 40% TBSA in a patient with a pregnancy in the 17th week of evolution.
**Methods**: We present the case of a 30-year-old patient, with a 40% TBSA burn by flame, located on the face, cervical posterior, right thoracic limb, posterior and internal face of the left arm, dorsal face of the left hand, both thighs and calves. The patient was 17 weeks pregnant with a viable fetus. A combination of surgical and non-surgical techniques is used for treatment: applying negative pressure to the burning areas of IIA and partially IIB to stimulate vascularization and seal the burned wound to avoid contamination. Serial excision of the burn areas and covering them with skin micrografts using the Meek technique, to avoid harvesting large areas and massive bleeding. The evolution of the pregnancy was monitored during the hospitalization by the obstetrician.
**Results**: The use of negative pressure kept the burned wound uncontaminated, the micrografts reduced the area of skin needed to cover the remaining skin defects after excision. The patient was discharged after 53 days of hospitalization, cured and with a pregnancy with a viable fetus.
**Conclusions**: The combination of surgical and non-surgical techniques, the reduction in blood loss during surgery, a good collaboration between the plastic surgeon, the anesthesiologist and the obstetrician led to a good result in the case of a 40% TBSA burn, as well as a good evolution of the pregnancy.

*P.042*

**Use of Nexobrid in the Treatment of the Critically Burned Patient. How Much Does It Cost to Treat 1% of Burned Skin Surface? Is It Cost-Effective?**

**Jacinto Baena ^1,2^, Angel Arevalo ^1,3^, Antonio Bulla ^1,4^, Andres Fernando Jimenez ^1,2^, Danilo Rivas ^1,4^, Sara Valles ^1,2^, Estela Novoa ^1,2^, Ariadna Gracia ^1,3^, Jordi Serracanta ^1,4^, Marcelino Baguena ^1,2^ and Joan Pere Barret ^1,2^**
  ^1^  Burn Unit of the Vall d’Hebron University Hospital
  ^2^  Neurotraumatology Intensive Care Unit of Vall d’Hebron University Hospital
  ^3^  Pharmacist Unit of the Vall d’Hebron University Hospital
  ^4^  Plastic and Reconstructive Surgery and Burn Unit of Vall d’Hebron University Hospital

**Objective**: To evaluate the cost-effectiveness of enzymatic debridement with Nexobrid in the critically burned patient who required surgical intervention (SI) during hospitalization.
**Methods**: We evaluated critically burned patients admitted to the Burn Unit of the Vall d’Hebron University Hospital from 2019 to 2021. Consecutive cohort of burned patients who required invasive mechanical ventilation (IMV) or amines given their severity on admission is studied.
With a total of 82 patients, we excluded 17 patients that did not require SI and 3 patients that required hospitalization for more than 100 days.
We collected demographic data, severity scales, presence of inhalation injury (IH), percentage of Burn Skin Surface (%BSS), days of IMV and mortality.
For the evaluation of economic expenditure, we collected the following data: need for tracheotomy, days of stay in the Intensive Care Unit (ICU), need for blood products, grams of Nexobrid used and number of SI performed to calculate the cost required to treat each 1% of BSS.
**Results**: of 62 patients, 35 were treated with Nexobrid plus SI and 27 with SI alone. of the Nexobrid group, 68.5% were men, mean age 45.6 years, abbreviated burn severity index (ABSI) of 8.9, mean BSS 41.2%. In total, 16 patients had IH, 13 required tracheotomy and a mean of 16.7 days of IMV with a mean ICU stay of 24.2 days. Average consumptions of 23.8 red blood cell concentrates (RBCC), 5.6 plasma concentrates (PC) and 1.3 platelet pool (PP) with an average of 3.3 interventions were performed per patient, along with an average consumption of 25 g of Nexobrid per patient. In total, 5 patients died.
About the group with only SI, 70% are men with a mean age of 52 years, a ABSI of 7.9 and an average of 27.6%BSS. of those 27 patients, 13 had IH, 9 required tracheotomy and a mean of 14.7 days of IMV. Mean ICU stay of 23.3 days with an average consumption of 23.3 (RBCC), 4.7 PC and 1.5 PP, requiring an average of 3.6 SI per patient. 8 patients were deceased.
The economic cost of Nexobrid group was 2054.8EUR 8 EUR/1% BSS compared with the group who just received surgical intervention which had a cost of 2800EUR 2800 EUR/1% BSS.
**Conclusions**: Patients in the Nexobrid group have higher ABSI, higher% BSS, more days of stay in ICU and more days of IMV.
The economic cost to cure 1% of BSS was lower in the group where Nexobrid was used.

*P.043*

**Multiple Application of Bromelain-Based Enzymatic Debridement (Nexobrid^®^) Combined with Tangential Debridement for Eschar Removal in Deep Thermal Burns: A Case Report**

**Diego Maté Martín and Eugenia López Suso**
  Plastic Surgery Department and Burn Center, Complexo Hospitalario Universitario de A Coruña

**Objectives**:
  − To compare the efficacy of the bromelain-based enzymatic debridement (Nexobrid) before and after the removal of persistent dry eschar by surgical debridement based on a case treated in our center.
  − To analyze the indications of the manufacturer of the product and the current literature about the adequate approach to persistent dry eschar in thermal burns before application of the bromelain-based enzymatic debridement (Nexobrid).
**Methods**:
  − A bibliographic search was carried out in the PubMed search engine for the terms “enzymatic debridement” and “Nexobrid”, selecting the articles related to the subject of this work by reading the abstract.
  − The manufacturer’s guidelines of the bromelain-based enzymatic debridement (Nexobrid) were reviewed.
  − The clinical history of a patient with a thermal burn who was admitted to our center was reviewed. The patient presented a full-dermal-thickness burn with dry eschar on its surface. Due to the depth of the burn, it was decided to perform enzymatic debridement with Nexobrid. This dry eschar limited the effect of the enzymatic debridement. after the poor result, we decided to perform a bedside escharectomy by surgical debridement to perform a new application of Nexobrid.
**Results**: The second application of the enzymatic debriding agent based on bromelain (Nexobrid) after surgical exeresis of the dry eschar achieved satisfactory debridement, removing all the devitalized tissue and respecting the healthy dermis. No adverse effect attributable to the second application of Nexobrid was detected. Seven days later a partial skin graft was performed. This graft evolved favorably, and complete re-epithelialization of the area was achieved 3 weeks later.
**Conclusions**: This case demonstrates that in cases of thermal burns with dry eschar at the time of application of the bromelain-based enzymatic debridement (Nexobrid), the efficacy of the product may be limited. In these cases, it seems necessary to remove previously the dry eschar to subsequently apply the product. Furthermore, performing a second enzymatic debridement with Nexobrid in the same area seems to be safe. However, more evidence is needed to prove it.

*P.044*

**Further Experience of Using Nexobrid Enzymatic Debridement in Burns Management**

**Katie Hilder ^1,2^, Andrew Warwick ^2^, Lydia Robb ^1,2^, Daniel Widdowson ^2^ and Hilal Bahia ^2^**
  ^1^  Ninewells Hospital
  ^2^  St. John’s Hospital

**Objectives**: Nexobrid is a pineapple-based enzyme used in burns debridement. It removes only eschar and necrotic tissue while preserving the available dermis, which allows for spontaneous healing. Our unit began using Nexobrid as a method of enzymatic debridement in 2017. This study shares our further experience of using Nexobrid in the management of eighty patients with full-thickness and mixed depth burns.
**Method**: Eighty patients presenting with full-thickness or mixed depth burns suitable for treating with Nexobrid were included in this study. Patient and burn demographics were noted, including cause, size (% Total Body Surface Area (TBSA)), depth and anatomical location of the burn, along with subsequent treatment and any complications.
**Results**: Overall, 88% of burns treated were less than 10% of the total body surface area, with 67% less than 5% TBSA. The majority (65%) were on the limbs, and more commonly of a mixed depth. Thermal burns accounted for 94% of cases. A delay in presentation of two or more days occurred in 22% of patients yet enzymatic debridement occurred within four days of injury in 86% of patients. Pain was a significant factor in this treatment modality. Subsequent skin grafting was required in only a fifth of all patients.
**Discussion**: Nexobrid has proven to be a beneficial tool in the management of full and mixed depth burns in our unit. Bedside administration of the treatment—made possible by our pain protocol—has been therapeutically advantageous to our cohort of patients who have avoided general anaesthesia and surgical debridement in the operating theatre. This has been particularly beneficial during the COVID-19 pandemic.
Multi-ethnicity in this patient cohort led to the development of patient information leaflets in more languages than English to enhance patient understanding and preparation for the procedure.
**Conclusions**: We have found Nexobrid to be an excellent for m of Burns management. This treatment option may be particularly useful in units where access to dedicated Burns theatres can be limited (e.g., during the global COVID-19 pandemic); however, adequate analgesia is a vital factor that must be considered when using enzymatic debridement.

*P.045*

**The Livingston Nexobrid Pain Protocol—Further Experience in 80 Patients**

**Katie Hilder ^1,2^, Lydia Robb ^1,2^, Andrew Warwick ^2^, Joanna Renee ^2^, Daniel Widdowson^2^ and Hilal Bahia ^2^**
  ^1^  Ninewells Hospital
  ^2^  St. John’s Hospital

**Objectives**: Our unit has employed Nexobrid, a pineapple-based enzyme used in the debridement of burns, for five years. It is a painful process; however, there is no for mal analgesia protocol in the literature. We developed a successful bedside pain protocol which is fast-acting, long-lasting, and has a quick recovery time. It is administered by our Acute Pain Team nurses, and reduces the need for invasive techniques and specialist medical staff. We present further data on the success of this protocol.
**Methods**: A fentanyl-based patient-controlled analgesia (PCA) protocol with clinician-administered boluses is used in this unit. Patients’ pain scores were documented throughout the procedure, as well as their perception of their worst pain and their overall satisfaction with the treatment. All patients undergoing enzymatic debridement with the ITU environment utilised this protocol.
**Results**: Eighty patients undergoing Nexobrid burns debridement were included in this study. The PCA ran for an average duration of 4 h and 15 min with a typical dose of 421 micrograms being administered. Pain was most severe on the removal of the enzyme; however, after bolus analgesia, the mean score at this point was 3/10. Addressing the significant psychological element of the pain response was an important role of the Pain Nurses in preparing patients for the debridement process. Patient satisfaction with the treatment was reported as “very satisfied” or “satisfied” in 95% of cases. No complications were reported.
**Conclusions**: Our analgesia protocol is safe, easy to administer, and remains well tolerated, providing a high level of patient satisfaction and low rate of complications in the enzymatic debridement of burns. The bedside use of Nexobrid, made possible by our pain protocol, was particularly beneficial during the COVID-19 pandemic when access to operating theatres was restricted. The role of the Pain Team Nurse in this protocol is important.

P.046

**European Regulatory Aspects of Phage Therapy: Magistral Phage Preparations**

Gilbert Verbeken ^1,^*, Thomas Rose ^2^, Jean-Pierre Draye ^1^ and Jean-Paul Pirnay ^1^
  ^1^  Laboratory for Molecular and Cellular Technology, Queen Astrid Military Hospital, Bruynstraat 1, 1120 Brussels, Belgium
  ^2^  Burn Wound Centre, Queen Astrid Military Hospital, Bruynstraat 1, 1120 Brussels, Belgium
  * Correspondence: gilbert.verbeken@mil.be

Bacteriophages (phages) are bacterial viruses and have been used for more than a century to combat bacterial infections, particularly in Poland and in the for mer Soviet Union. The antimicrobial resistance crisis has triggered a renewed interest in the therapeutic use of natural phages. The capacity of phages to specifically target pathogenic strains (sparing commensal bacteria), to adapt to these strains, and to rapidly overcome bacterial resistance, makes them suitable for flexible therapeutic approaches. To maximally exploit these advantages phages offer over conventional ‘static’ drugs such as traditional small-molecule-type antibiotics, it is important that these sustainable phage products are not submitted to the traditional (long and expensive) medicinal product development and licensing pathways. This poster discusses the Belgian ‘magistral preparation’ phage therapy framework and the extrapolation of this framework to the European level, enabling an expeditious re-introduction of personalized phage therapy into Europe. The magistral preparation pathway is a short and feasible pathway allowing patients’ access to personalized and sustainable phage therapy products. Physicians, pharmacists, phage active pharmaceutical ingredient (pAPI) producers and EDQM reference laboratories each play their specific roles. Physicians prescribe personalized (tailored) phage preparations for use in specific patients. Pharmacists prepare these phage products according to the individual prescriptions, using pAPIs. Industry and non-profit players produce these pAPIs according to a phage monograph, and reference laboratories perform the QC release testing of these pAPIs. Industry can market these pAPIs. Pharmacists can also outsource the production of magistral phage preparations to industry. A general phage chapter, once included in the European Pharmacopoeia (Ph. Eur.), can (non-restrictively) guide the quality of the produced and released pAPIs. Further efforts are needed to incorporate general and specific phage monographs into the Ph. Eur. This process should be driven by industry, if and when they feel the need.

*P.047*

**Use of the New Filtration Membranes for Sepsis: A Valid Opportunity in Burn Patients Admitted in the Intensive Care Unit. Experience from Our Burn Center**

**Giuseppe Spaltro ^1^, Tiziana Pagliarini ^1^, Lorenzo Secondi ^2^, Valerio Cervelli ^2^, Marco Schirosi ^1^, Simone Moroni ^1^, Paolo Palombo ^1^ and Vincenzo Angeloni ^3^**
  ^1^  Uoc A.S. Centro Ustioni E Chirurgia Plastica Ospedale S. Eugenio Roma
  ^2^  Università degli studi di Tor Vergata
  ^3^  UOC Nefrologia e Dialisi

**Introduction**: Acute Kidney Injury (AKI) is a common complication in burned patients affected by sepsis. Moreover, it represents an important risk factor since it increases the mortality of the patients admitted to an intensive care unit (ICU). The sepsis induces the release of inflammatory cytokines who may determine an acute renal failure aggravating the prognosis of the patient.
A consistent percentage of burn patients hospitalized in ICU may develop infections in the areas of burned skin; therefore, they may undergo to blood infection and subsequently sepsis-associated AKI. The exact timing for receive renal replacement therapy for those patients remains disputed.
**Materials and Methods**: From January 2018 to January 2020, 11 burn patients affected by sepsis were treated with Continuous Renal Replacement Therapy (CRRT) with Special Filters (SF) in the Burn Center of the “Sant’Eugenio” hospital in Rome. In total, 5 patients were affected only by blood infection without AKI, whereas 6 patients experienced acute renal failure secondary to multiple etiology and sepsis. All patients received an accelerated strategy of renal-replacement therapy (within the first 12 h after admission). The treatment proposed was the use of the new membranes (Septex and Oxiris) for both groups. Patient selection was performed according to the guidelines of SEPSI and KDIQO.
Regional Citrate Anticoagulation (RCA) was used in patients treated with Oxiris filter, while systemic anticoagulation with unfractioned heparin was performed when Septex filter has been used.
SOFA score was recorded for each patient at the time of hospitalization.
The primary endpoint was the mortality at 30 days after hospitalization.
**Results**: In total, 7 patients were alive at 30 days after hospitalization, and 4 of them were treated with the Oxiris filter and the remaining 3 patients treated with the Septex filter. Overall, 4 of these patients were not affected by AKI.
Filtering efficiency in terms of timing for depletion of filtration capacity was better with Oxiris than Septex: the half-life recorded was 44 h for Oxiris and 24 h for Septex. Moreover, the efficacy of the RCA in the prophylaxis of clotting during hemodialysis with Oxiris filter was shown to be higher than that with systemic unfractional heparin administered with Septex filter.
**Conclusions**: Burn patients affected by sepsis without AKI who have been treated with CRRT showed a better 30-day survival in comparison with those patients who were also affected by AKI. A better filtering efficiency was detected with Oxiris than Septex filters. Regional Citrate Anticoagulation was better than the heparin strategy.

*P.048*

**Burns Colonized by *Pseudomonas aeruginosa*: A Fast and Efficient Track down and Treatment**

**Maryline Vandeputte and Flore Van Hyfte**
  Uz Leuven

**Introduction**: after burn injury, the loss of the protective skin barrier, the presence of dead tissue, and the humid wound environment contribute to ideal breeding conditions for microorganisms. Pseudomonas aeruginosa is a feared and frequent cause of burn infections. It can lead to delayed wound healing, wound sepsis, and the failure of skin grafts. The rapid and efficient detection and treatment of burns colonized by Pseudomonas is therefore crucial.
**Objective**: The objective of this study is to propose the approach used by the burn centre/ICU of UZ Leuven to track down and treat a patient with burns colonized by Pseudomonas.
**Methods**: We describe an existing care protocol in the perspective of the retrieved scientific evidence based on a detailed literature review.
**Results**: The UZ Leuven approach consists of three pillars: early recognition, treatment, and prevention.
Early recognition of a wound colonized by *Pseudomonas* is crucial to prevent wound sepsis and is guided by the typical smell and a green biofilm. Wound cultures should be taken when there is the slightest suspicion of a wound infection, in addition to fixed-time surveillance cultures. The treatment should not be delayed until the results of the wound cultures are available.
Our standard treatment for critical colonization consists of mechanical cleaning and/or debridement to destroy the biofilm and of increased frequency of dressing changes. Treatment for *Pseudomonas* colonization typically includes hydrotherapy, the application of acetic acid 1% for 30 min, and the application of Flammazine^®^.
To prevent a *Pseudomonas* outbreak, precautionary measures are followed. This includes a weekly weighing of the hydrotherapy equipment (stretcher, mattress, and cushions), for the early detection of leakage of water through shell defects; a weekly bacteriological examination of water samples; and changing of the water filter, guided by a strict calendar. The equipment for hydrotherapy is thoroughly disinfected after every single patient with DialoxTM containing 1 g peracetic acid and 7 g hydrogen peroxide per 100 g.
The impact of this protocol appears to have historically the prevention of Pseudomonas outbreaks, and thereby, wound healing. Prospective comparative, perhaps clustered randomized studies are required to for mally validate our approach.
**Conclusions**: The treatment of burns colonized by Pseudomonas aeruginosa is challenging. There is no contemporary consensus between different burn centers, given the lack of hard evidence. While awaiting such evidence, an approach based on a multi-center and multi-disciplinary consensus would be valuable for patients and caregivers.

*P.049*

**Investigation of Novel Circulating Biomarkers in Burn Septic Shock Patients**

**Martina Schiavello ^1^, Barbara Vizio ^1^, Filippo Mariano ^1^, Anna Pensa ^2^, Ornella Bosco ^1^, Maurizio Stella ^2^, Enrico Lupia ^1^, Giuseppe Montrucchio ^1^**
  ^1^  Department of Medical Sciences, University of Turin
  ^2^  Burn Centre, CTO Hospital, A.O.U. Città della Salute e della Scienza

**Objectives**: Septic shock is the main cause of mortality in patients with severe burns. Extracellular vesicles (EVs) have emerged as novel cell-to-cell communication mediators. Non-coding RNAs and proteins encapsulated by EVs could result in either pro-inflammatory or anti-inflammatory effects in the recipient cells. The aim of this study is to evaluate whether alteration exists in the quantification and characterization of EVs circulating in burn septic shock patients. These results will clarify the molecular mechanisms of EVs involved in burn septic shock patient outcomes.
**Methods**: Twenty-five burn patients were enrolled in the Burn Centre of Turin, including burn-septic shock patients (33.69 ± 14.94% TBSA; 11 ± 2.34 SOFA score) and burn-non-septic patients (16.67 ± 7.64% TBSA). Healthy subjects were used as controls. Blood samples were collected and plasma-derived EVs were isolated by high-speed ultracentrifugation and charge-based precipitation methods. EV concentrations and sizes were analyzed by nanoparticle tracking analysis (NTA). EV characterization was performed by transmission electron microscopy (TEM) and flow cytometry analysis.
**Results**: Plasma-derived EVs were successfully isolated, and their presence was confirmed by TEM, which exhibited the characteristic cup-shaped morphology of EVs, and by the flow cytometry analyses of small EVs-tetraspanins markers, which showed enrichment in CD63 and CD9. Preliminary results show that the EV concentration is increased in burn-septic shock patients compared with healthy subjects, whereas the EV diameter shows no significant differences. The EV concentration was correlated with the clinical parameters of the patients.
**Conclusions**: In this population of patients, preliminary results provide evidence that EV concentrations are potentially related to burn patient’s critical inflammatory states.

*P.050*

**Is the Use of a Powered Dermatome an Aerosol-Generating Procedure (AGP)? Implications for Personal Protection against COVID-19**

**Kayvan Shokrollahi, Ioannis Kyriazidis, Shomari Zack-Williams, Laura Cappuyns, Claire Jones, Elisa Murgatroyd and Dilnath Gurusinghe**
  St. Helens & Knowsley Teaching Hospitals NHS Trust

**Introduction**: Many healthcare workers have contracted SARS-CoV-2 during the COVID-19 pandemic, many cases of which have resulted in severe illness and death. No studies have assessed the potential for powered dermatomes to generate aerosols, an essential technique in burns and plastic surgery.
**Objective**: The primary aim of the present study was to capture video footage to illustrate the potential for a powered dermatome to generate significant spray, and hence, aerosol.
**Methods**: We utilised a simulated skin graft harvest experimental method. Fluorescein-stained saline was used with ultraviolet (UV) backlighting to demonstrate fluorescent spray from a popular brand of air-powered dermatome. Ultra-slow-motion (960 frames/s) video was used to demonstrate the oscillation of the dermatome blade and the origin within the machine of any spray generated, as well as the extent of spray generated.
**Results**: The key finding from this study is the captured video footage linked with this paper. Droplets of various sizes are seen spraying out from the leading edge at the sides where the blade oscillates. UV backlighting provides a clear demonstration of the dermatome generating fine spray.
**Conclusions**: Our study demonstrates that powered dermatome usage is likely to generate aerosol from blood or blood-contaminated fluid; however, it does not demonstrate or quantify to what extent this may be clinically relevant in terms of viral transmission potential. We suggest ways to reduce the risk of spray from dermatomes including limiting donor-site bleeding and avoiding a wet donor area.

*P.051*

**Experience with Cadaveric Skin Processed as Temporary Coverage in Children with Full-Thickness Burns and Positive Cultures for Pan-Resistant Bacteria in the Burn Unit IMSS**

**Claudia Berenice Hernández Valverde**
  Imss

**Objective**: We assessed the usefulness of cadaverican donation skin (biotissue) for temporary coverage in burn patients in pediatric patients with full-thickness burns who had delayed treatment due to infection.
Standardization of the methodology of the use of biotissue if the use of the product is significant.
**Method**: I sequentially selected pediatric patients with positive cultures for *S*. *aureus* and *P*. *aeuruginosa* panresistante, admitted to the burn unit with full-thickness burns, who had a delay in reconstructive treatment due to infection. Culture biopsies are reported.
Each surgery was carried out, with surgical cleansing and tangential excision if necessary, always taking a biopsy, culture, and placement of biotissue fixed with skin staples and furated gauses.
The final reconstructive management was performed with skin grafts.
The evolution was documented photographically and with the results of sequential culture biopsy.
Biotissue is the name of the final product of the procurement, process, sterilization, and conservation of cadaverican skin. Granted to the Mexican Social Security Institute through the national organ donation program.
**Outcome**: The usefulness of biotissue was assessed in seven patients with full-thickness burns with various injury mechanisms: immersion, explosion and electrical conduction with an age range of 2 to 14 years.
Since the first application of biotissue, clinical improvements have been observed in granulation tissue, reductions in the bloody area, increased vascularity, decreased biofilm development, and hypertrophic granulation tissue. The ABSI rating was 6 to 9 in this group.
In the same patient, the photographic control demonstrated epithelization and improvements in the bed.
Together with the Institute of Biotechnology, the procurement of skin was carried out as a team to determine the required specifications regarding the thickness and length of the pieces.
The children treated with temporary coverage were successfully grafted, epithelized in some areas, and only in one case an advancement flap was used for bone coverage.
**Conclusions**: for the burn unit of the VFN Traumatology Hospital of the Mexican Institute of Social Security, it was greatly useful to have this resource.

*P.052*

**Novel Approach to Early Burn Eschar Removal in Massive Casualty Events**

**Agnieszka Surowiecka, Jerzy Struzyna and Tomasz Korzeniowski**
  East Center of Burns Treatment and Reconstructive Surgery

Mass burn casualties are no longer only a potential scenario. Our Burn Center has been prepared for mass casualties for several years. Early burn eschar removal up to 20% TBSA is advised even in severe conditions. In mass casualty events, there are limitations in the blood supply, operating room, medical staff, and equipment. In our opinion, enzyme debridement optimizes the treatment, as well as its costs. In terms of the shortage of supplies, enzymatic eschar removal might be an alternative to early surgery. The dermal tissues left after enzymatic debridement significantly reduce the area of the burn and promote self-healing. Time gained can be used to transfer patients and stabilize severe situations. Additionally, enzymatic debridement performed in selected cases may prevent from progression of the depth of the burn. The aim of this study is to share our internal plan for dealing with mass burn casualty events.
**Material and Methods**: The plan was developed basing on military experience of the Head of the Department and the current literature. There are many descriptions of prehospital triage methods. However, there are limited data on patient selection, the timing of early burn excision, and surgical techniques. Our plan precisely described segregation of duties and timing of surgical intervention in a burn unit. The plan prepares for severe circumstances with limitations of resources, equipment, and medical staff.
**Results**: We created burn teams that included burn surgeons, 2–3 burn nurses, and a hospital ward. Each burn team can perform up to three early eschar removals during 12 h. Having three burn teams, we could perform enzymatic debridement in even nine patients during 12 h, which is impossible in case of surgical excision. The indications for enzymatic debridement in our frontal hospital include deep burns with an extension of up to 20% TBSA. We also have experience in younger patients with good prognoses with burns of 60% TBSA. In these cases, we performed enzymatic debridement of 15% each day. There is a nurse training team and a plan to create burn teams in non-burn centers that could cooperate with us.
**Conclusions**: Mass burn incidents are an example of a situation where the resources of the rescuers may prove insufficient. Surgical excisions call for operating rooms, specialist equipment, blood supply, and experienced burn surgeons. Early enzymatic debridement should be considered instead of surgical excision in some cases. Domestic and international burn mass casualty response plans should be developed.

*P.053*

**Potential Benefits of NexobridTM vs. Standard of Care for Burn Center Mass Casualty Management**

**Jasminka Minic, Edoardo Dalla Pozza, Enrico Vigato and Maurizio Governa**
  Plastic Surgery and Burn Unit, A.O.U:I. of Verona

**Objectives**: Burn management is resource-intensive. NexobridTM (NXB) is efficacious for enzymatic debridement (ED), (1) indicated for deep partial- and full-thickness thermal burns, (2) and may reduce resource usage. (3) We assessed its effects on resource utilization in anticipation of mass casualty management.
**Methods**: Data on patient characteristics, and the utilization of human resources, operating room time, and blood transfusions, were retrospectively collected for adult patients treated at the Verona burn center. Patients who underwent enzymatic debridement (ED) (*n* = 20) were compared with patients matched for age, burn area, and burn depth (*n* = 20) who had received surgical debridement as the standard of care (SOC). Results were analyzed using descriptive statistics and log Gamma modeling. Institutional Review Board approval #2214CESC.
**Results**: Mean (±SD) ages were similar between groups: SOC, 54 ± 15 years, 13/20 men; NXB, 56 ± 15 years, 15/20 men. One or more major comorbidity was present in 10 patients in each group, mean (±SD) 2.1 ± 1.1 comorbidity per affected patient in SOC, and mean 1.8 ± 0.92 per affected patient in ED. Initial burn parameters were also similar (Table). during debridement, the use of ED was associated with fewer mean operating room procedures (0.15 ± 0.366 vs. 1.5 ± 0.607; *p* < 0.01), fewer surgeon hours (77 ± 22 vs. 159 ± 76; *p* < 0.01), and lower hospital resource utilization (Figure 1A). The log Gamma model confirms that performing debridement was significantly associated with fewer days of hospitalization, fewer operating room procedures, and significantly lower hospital staff involvement, total healthcare utilization, and blood units consumed (all *p* < 0.001). At discharge, ED was associated with fewer OR procedures (1.05 ± 0.605 vs. 1.8 ± 0.616; *p* < 0.01) and the involvement of fewer surgeons (2.2 ± 0.894 vs. 3.85 ± 1.268; *p* < 0.01), anesthesiologists (1.2 ± 0.41 vs. 1.8 ± 0.616; *p* < 0.01), and nurses (3.6 ± 1.57 vs. 5.7 ± 1.455; *p* < 0.01) (Figure 1B). The log Gamma model confirmed that ED was significantly associated with fewer operating room procedures (*p* = 0.011), a lower number of hospital staff involved (*p* = 0.003), and less total staff time utilization (*p* < 0.001); total hospital days at discharge were similar (ED 38 ± 12 vs. SOC 43 ± 17).
**Conclusions**: Enzymatic debridement is an effective means to reduce hospital resource utilization for burn eschar removal. By allowing non-surgeons to perform debridement outside of the operating theater, it may overcome surgical bottlenecks associated with the management of multiple burn patients, e.g., in the event of a mass casualty incident involving burn patients. The implementation of enzymatic debridement may increase burn center surge capacity and should be considered in preparedness assessments.

*P.054*

**The Use of Intact Fish Skin as a Novel Treatment Method for Deep Dermal Burns following Enzymatic Debridement: A Retrospective Case–Control Study**

**Björn Behr, Jana Holtermann, Drysch Marius, Schmidt Sonja, Reinkemeier Felix, J. Maximillian Wagner, Mehran Dadras, Alexander Sogorski, Houschyar Khosrow, Mustafa Becerikli, Marcus Lehnhardt and Christoph Wallner**
  University Hospitalbergmannsheil Bochum

**Background**: Optimal therapy for deep burn wounds is based on the early debridement of necrotic tissue followed by wound coverage to avoid a systemic inflammatory response and optimize scar-free healing. The outcomes are affected by available resources and underlying patient factors, which represent challenges in burn care and suboptimal outcomes. In this study, we aimed to determine optimal burn wound management using enzymatic debridement (NexoBrid™, MediWound Germany GmbH, Rüsselsheim, Germany) and intact fish skin (Kerecis^®^ Omega3 Wound, Isafjordur, Iceland).
**Methods**: In this retrospective case series, 12 patients with superficial or deep dermal burn wounds were treated with enzymatic debridement followed by fish skin, Suprathel^®^ (PolyMedics Innovations GmbH, Denkendorf, Germany), or a split-thickness skin graft (STSG). Patients’ outcomes regarding healing and scar quality were collected objectively and subjectively for 12 months after the burn injury. Results: Wounds treated with fish skin demonstrated accelerated wound healing, a significantly higher water storage capacity, and better pain relief. Furthermore, improved functional and cosmetic outcomes, such as elasticity, skin thickness, and pigmentation, were demonstrated. The pain and itch, expressed as POSAS scores (Patient and Observer Scar Assessment Scale), for fish skin decreased compared with those for wounds managed with an STSG or Suprathel. Importantly, fish-skin-treated wounds had significantly improved sebum production and skin elasticity compared with those treated with Suprathel, but showed no significant superiority compared with STSG-treated wounds. Conclusions: Enzymatic debridement in combination with intact fish skin grafts resulted in the faster healing of burn wounds and better functional and aesthetic outcomes than split-thickness skin grafts and Suprathel treatment.

*P.055*

**Virtual Reality in Specialized Burn Care: Health Care Professionals’ Perceived Barriers and Facilitators**

**Saskia Sizoo ^1^, Moniek Akkerman Akkerman ^2^, Marscha Heijblom-van Dinteren ^1^, Inge Spronk ^3,4^, Marianne K. Nieuwenhuis ^2,5,6^ and Margriet E. van Baar ^3,4^**
  ^1^  Burn Centre Maasstad Hospital
  ^2^  Association of Dutch Burn Centres, Martini Hospital
  ^3^  Association of Dutch Burn Centres, Maasstad Hospital
  ^4^  Department of Public Health, Erasmus MC, University Medical Center Rotterdam
  ^5^  Research Group Healthy Groningen, Allied Health Care and Nursing, Hanze University of Applied Sciences
  ^6^  Department for Human Movement Sciences, University Medical Center Groningen

**Introduction**: Virtual reality (VR) is a promising technique in specialized burn care. In The Netherlands, VR is sometimes used in burn wound care procedures to distract patients from high levels of pain. This study investigated barriers and facilitators for health care professionals (HCPs) on the implementation of VR in Dutch burn care.
**Methods**: An online survey study was conducted in the three Dutch burn centres in November–December 2021. HCPs completed an adapted version of the Measurement Instrument for Determinants of Innovations questionnaire to identify barriers and facilitators of VR application in burn care. If ≥20% of participants responded with ‘totally disagree/disagree’, items were considered barriers; if ≥80% responded with ‘agree/totally agree’, items were considered facilitators.
**Results**: A total of 64 HCPs completed the survey. The majority of participants were nurses (54.7%) or physicians (10.9%), with experience of up to 5 years in burn care (37.5%). A total of 33 HCPs (51.6%) used VR in clinical burn care practice. No differences were found between VR users and non-users in terms of demographic and work-related characteristics. A variety of VR systems were used, all being immersive systems with a head-mounted display.
Fifteen barriers and eight facilitators to the application of VR in clinical burn care were identified. Barriers included time constraints, lack of experience, limited awareness of VR components, and the limited use of VR by colleagues.
Facilitators were associated with the opportunity to include patient values, and the opportunity to make their own considerations on the application of VR. Additionally, VR was considered easy to use and was expected to result in positive outcomes for patients, mainly related to distractions from pain and relaxation.
**Conclusions**: To further implement VR in clinical burn care, it is important to be aware of the identified barriers and facilitators when considering implementation strategies.

*P.056*

**Scales Used for the Assessment of ADL Applied to the Burn Patient: A Clinical Case**

**Mahtab Nikzat ^1^, Daniela Arena ^2^, Danila Toscano ^3^, Luciano Braghin ^4^**
  ^1^  Ospedale Civico di Settimo Torinese
  ^2^  Città Della Salute e Della Scienza (CTO)
  ^3^  Presidio Sanitario San Camillo
  ^4^  Città Della Salute e Della Scienza (CTO)

**Objectives**: The physical and psychological complications resulting from burn injuries often have a considerable impact on the patient’s daily activities, and consequently, on the quality of life.
The assessment of ADL through special tools is crucial to treating severe burns.
Therefore, from the early stages of disease, the patient’s functional status should be determined.
It is recommended to constantly monitor the patient’s functional development in order to recognize the degree of independence at each stage of disease.
In the literature, there is only one specific scale to assess the quality of life and ADL of burn patients: the “Burn Specific Health Scale” (BSHS). It has two different versions:
  − Abbreviated BSHSA;
  − Brief BSHSB.
**Methods**: This study was conducted in 2018 using recently published scientific articles found on Pedro and PubMed databases.
In this study, the scales used for the evaluation of one burn patient followed them throughout all phases of the disease, derived from the scientific articles reviewed.
**Results**: In all stages of the disease, in addition to the review scales, the Brief Pain Inventory (BPI) was administered to measure the pain caused by the burn and the level of its interference with certain daily activities.
  According to collected data, the best scales for each phase are:
  − Acute phase;
The Modified Barthel Index (MBI) scale has been found to be an appropriate tool for ADL assessment, because its items are better adapted to the patient’s clinical conditions and show the best sensitivity to functional changes.
  − Sub-acute phase;
Again, the previous scale is used in this phase because it enables us to monitor and evaluate every single change in the patient’s functional progress.
  − Final phase.
In this phase, instead of the MBI scale, the abbreviated BSHS and the Brief BSHS can also be used. These scales are known for their capability to evaluate burn patients in terms of their physical, mental, social, and general health development.
**Conclusions**: The study demonstrates that the scales used at all stages of the disease are suitable for patient evaluation.
However, further analysis of the scales is needed through conducting new studies on a wider range of burn patients.

*P.057*

**First Application Results of a New Type of Synthetic Polylactide Matrix (SupraSDRM^®^) for Deep Dermal to Full-Thickness Burns as a Dermal Skin Substitute with Two-Step Split-Skin Covering**

**Matthias Rapp, Robert Schappacher and Ulrich C. Liener**
  Clinic for Orthopedics, Trauma Surgery and Sports Traumatology—Burn Center, Marienhospital Stuttgart

**Objectives**: Dermal substitutes restore or reconstruct the dermal skin layer in order to make the skin quality flexible and supple again after wound closure. Dermal materials currently available on the market mostly contain ingredients of xenogeneic, animal (porcine, bovine, and pescine) origin based on collagen or hyaluronic-acid-based components or modifications, or they are of allogeneic origin. Xenogeneic and allogeneic materials carry the risk of an immunological reaction or disease transmission.
**Methods**: SupraSDRM^®^ is a purely synthetic, degradable, polylactide-based copolymer in a bimodal foam–membrane structure. The bimodal structure promotes cell migration, vascularization, and collagen incorporation into the polylactide foam membrane structure of SupraSDRM^®^. The lactate resulting from hydrolytic degradation promotes angiogenesis and dermis for mation and reduces inflammation and oxidative stress. after deep or epifascial necrectomy, SupraSDRM^®^ was applied to fascia, muscle, tendon, and fat tissue, as well as to decorticated bone. after the for mation of a well-vascularized wound bed, the surface of the dermal skin replacement was refreshed, and a split-skin mesh graft transplantation was carried out in a two-step-procedure.
**Results**: In *n* = 6 patients with a mean age of 60.2 years, a mean affected skin surface area of 11% TBSA, and a mean ABSI-score of 7.3, SupraSDRM^®^ was applied in 13 locations. after deep dermal or epifascial necrectomy, SupraSDRM^®^ was placed on 5 different wound bases: fat *n* = 3, fascia *n* = 3, muscle *n* = 4, tendon *n* = 2, and bone *n* = 1, with a mean wound area of 1.9% TBSA. after progressive vascularization of the bimodal structure of SupraSDRM^®^, plastic coverage with split-skin mesh grafts could be performed after an average of 13.5 days. It was shown that the split-thickness skin grafts had largely healed, with good mobility and little or no shrinkage of the scars.
**Conclusions**: The purely synthetic, polylactide-based matrix of SupraSDRM^®^ combines the advantages of the established Suprathel^®^, which is also based on polylactide, with the high porosity known from other dermal skin replacement materials. Progressive vascularization of the bimodal matrix with increasing cell migration leads to remodeling of the matrix with the for mation of dermal granulation tissue, which can be covered in two stages with split-skin mesh graft transplants in a timely manner. The first applications of SupraSDRM^®^ on burns show good mobility of the soft tissues without the for mation of hypertrophic scars and scar keloids, with good functional, mechanical, and aesthetic results. SupraSDRM^®^ could represent a good alternative as a novel dermis replacement.

*P.058*

**The Effect of Burn Injuries on the Brain and Behaviour**

**Amira Allahham**
  Fiona Wood Foundation

Burn patients, especially children, are more prone to mental health conditions following their injury, as recently demonstrated using population-based studies in Western Australia. The inflammatory response to a burn, coupled with a leaky blood–brain barrier may lead to immune and inflammatory changes in the brain that underlie the long-term increase in mental health hospital admissions observed. A mouse model was used to: (1) investigate the changes in behaviour following burn injury; and (2) to determine the genes in the brain with altered expression following burn-injury. Mice were allocated into three intervention groups: a burn group which received a non-severe burn injury 7–8% of the total body surface area administered under anaesthesia; an excision group which received a 7–8% wound to control for the effects of trauma without the burn; and a sham group that received the anaesthesia and no injury. Mice were tested through a series of behavioural tests before and after their intervention. At the conclusion of the experiment, mice were euthanized, and their brains were collected for genetic analysis through RNA sequencing. Behavioural tests showed no significant difference before and after the burns; however, significant changes were shown in genes associated with pathways of neurodegenerative diseases. More investigation of the genetic pathways after burns is required to understand the impact of burn injuries on the brain to eventually be able to understand and treat the mental health conditions that arise after burns.

*P.059*

**Does a Combined Treatment via Nexobrid^®^ and Kerecis^®^ Perform on a Par with Conventional Severe Burn Injury Treatment? A Case Report Study by a Centre for Severely Burned People**

**Maresa Dorothee Berns, Tamas Püski and Bert Reichert**
  Klinik für Plastische, Wiederherstellende und Handchirurgie, Zentrum für Schwerbrandverletzte, Universitätsklinik der Pmu

**Objectives**: In deep partial-thickness burns, early debridement is known to be essential for later outcome as well as the choice between wound dressing or skin grafting. Since 2012, people with burn injuries can be treated with enzymatic debridement as an alternative to conventional eschar removal using a knife (e.g., Humby knife).
The aim of this case report study was to determine the advantages and disadvantages of enzymatic debridement and subsequent wound care via fish skin grafts. We wanted to explore the handling during the procedure and the short- and long-term outcome. Especially considering scar quality and the resulting range of motion, we followed up people with large-scale burn lesions and close-to-joint burn wounds.
**Methods**: This study was a retrospective 1-year case report of a single-center population undergoing enzymatic debridement and wound dressing with decellularized fish skin. We present patient cases from our burn unit who were treated with a combination of enzymatic debridement and fish skin wound dressing.
**Results**: In most of the explored cases, we recognized a full resorption of the fish skin by tissue. Only in one evaluated case did we need to transplant skin secondarily. Thus, the addressed issues as indications, the technique and timing of application, after-intervention care, and outcome were evaluated and were shown to be successfully accomplished. As presumed, satisfactory scar quality results led to the loss of need of skin grafting and a near-to-normal range of motion during the first 6 months after an accident event.
**Conclusions**: Significantly accelerated wound healing was observed in most of our cases treated with fish skin. The re-epithelialization after enzymatic debridement and usage of fish skin led to reduced need of skin graft operations, reduction in surgery time and reduction in post interventional infection. By avoiding immobilisation of treated body areas early functional exercise and high patient satisfaction result. Further studies are needed to deduce systematic guidelines or a consensus paper regarding the combination of enzymatic debridement and fish skin wound dressing.

**Keywords**: surgery reconstruction; wound healing; outcomes; enzymatic debridement

*P.060*

**Combination of “Sandwich” Grafts and Autologous PRP in the Treatment of Severe Burns**

**Tiziana Pagliarini ^1^, Simone Moroni ^1^, Giuseppe Spaltro ^1^, Marco Schirosi ^1^, Andrea De Bellis ^1^, Paolo Palombo ^1^, Valerio Cervelli ^2^ and Lorenzo Secondi ^2^**
  ^1^  Uoc A.S. Centro Ustioni E Chirurgia Plastica Ospedale S. Eugenio Roma
  ^2^  Università degli studi di Tor Vergata UOC Chirurgia Plastica

**Introduction**: The main factor limiting reconstructive procedures and their effectiveness in burns is the insufficient availability of autologous skin to cover the loss of tissue.
The aim of this study was to evaluate the possible advantages of the combination of the Alexander surgical technique and platelet-rich plasma (PRP) as a catalyst and adjuvant, in the treatment of extensive burns.
**Rationale**: Platelet-rich plasma (PRP) is the concentration of autologous human platelets in a small amount of the Alexander procedure is a “sandwich” grafting technique where homologous skin covers the underlying widely meshed skin autograft. In this way, a small portion of autologous skin can cover a large area after superficial and deep tangential escarectomy while remaining protected by the homologous graft which acts as a mechanical barrier.
**Methods**: The study was conducted in the Burn Center and Plastic Surgery Department, St. Eugenio Hospital in Rome, starting in February 2022, and involved patients with third-degree burns extending to more than 20% TBSA. The characteristics of the patients, burns, areas, etiology, and prognostic factors were taken into consideration to better evaluate and interpret the results.
The major inclusion criterion was the candidacy of patients for homologous skin grafting.
Thus, the only exclusion criterion was thrombocytopenia, as a condition incompatible with the PRP technique.
The classical Alexander surgical technique was integrated with multiple PRP injections in the subcutis after the escharotomy and haemostasis phases and before the grafting, to enhance the protective and regenerative function of the skin covering.
**Results**: The results showed that there was a better coverage of the loss of substance, with faster re-epithelialization and autologous skin graft take, and reduced hospitalization time. In some cases, a single surgery allowed the patients to heal completely.
**Conclusions**: In extensive third-degree burns, the homologous graft allows temporary coverage and acts as a barrier both to the external environment and to the loss of fluids. At the same time, the widely meshed skin autograft takes and for ms a definitive cover. The PRP stimulates the engraftment both by stimulating re-epithelialization in the spaces of the meshes, and by inducing neoangiogenesis which allows the survival of the autologous graft and the antibiotic coverage of the affected area. In conclusion, the combination of PRP and the Alexander technique can achieve complete healing and discharge of patients with fewer surgeries and reduced hospitalization.

*P.061*

**Skin Donor Site Management Using the Meek Technique in Severely Burned Patients**

**Giulio Maggio, Claudia Corrao, Alessio De Cosmo, Antonio Staffa and Giuseppe Giudice**
  Plastic Surgery

In severely burned patients, skin donor site healing is always a real problem.
The cause of unhealing wounds is nevertheless the infection favoured by immunosuppression and typical unbalanced catabolism of severely burned patients.
According to contemporary guidelines, the standard of care for skin donor sites is represented by Vaseline gauze only, consecutively to cleansing and disinfection.
Usually, the Meek technique represents an original method to expand a very small skin graft with a ratio from 1:2 to 1:9, thanks to novel pre-folded manufactured gauze, producing square skin islands easy to use in the management of burned patients for the reconstruction of very large areas.
This study was finalized to verify the “healing time” and the “percentage of healing” of donor site using autologous skin 1:6 obtained with the Meek technique versus Vaseline gauze (standard of care).
From January 2021 to January 2022, we enrolled 10 patients (6 males and 4 females), aged between 18 years old and 60 years old, with a TBSA (total body surface area) of 40–60%; each patient had two donor site areas, the one treated with Meek technique (Study group) and the other treated with the standard of care (Control group).
The results show that the healing of donor site treated with Meek technique was 95.3% in 10 days (at the scaffold removal), whereas in the area treated with Vaseline gauze, the percentage of healing was 54.7% in 15 days (according to guidelines).
The complete healing of donor site areas treated with the Meek technique was obtained in 14 days, whereas the complete healing of donor site areas treated with Vaseline gauze was obtained in 41 days.
In conclusion, the use of the Meek technique 1:6 to treat the donor site areas has been very effective in order to reduce the surface of unhealing areas in severe patients with very extensive wounds, avoiding the unhealing of donor site areas, reducing the time of hospitalization, and improving outcomes in these difficult-to-treat patients.

*P.062*

**Minimal Invasive Modality (M.I.Mo) in a New Technological Approach for Severely Burned Patients**

**Giuseppe Di Gioia, Giulio Maggio, Alessio De Cosmo, Martina Rosa Iuliano and Claudio Torrisi**
  Plastic Surgery

Not so long ago, the conventional treatments of burn patients were long, expensive, painful, and not very effective. These were based on surgical escharotomy, constant medication, and final coverage with partial thin skin grafts.
In recent years, the standard of care, has considerably changed and turned into a more selective and less invasive procedure. Nowadays, when it comes to burn patients, the minimally invasive modality treatment (MIMO) is mandatory.
It consists of a first step that is performed by the end of 48 h from the burn event; the patient undergoes enzymatic debridement using NEXOBRID^®^, which contains a mixture of enzymes enriched in bromelain, which dissolve burn wound eschar. It is spread in sedation at bedside on a maximum of 15% of TBSA of the patient per time; it is left on for 4 h and then removed so that medication with collagenase ointment can be performed.
After 7 days, a pseudo eschar is for med on the treated area, and it is removed in the surgery room with idro-surgical treatment. Subsequently, medication with dermal substitutes soaked with stem cells obtained through lipoaspirate is performed. Ten days later, the dermal substitute is removed, and from that time, serial dressings are performed.
These steps are repeated on each burn area, with the target of obtaining the best wound bed with second intention healing, so as to reduce the necessity of skin graft coverage.
The MIMO protocol has been investigated in different studies with the evidence of obtaining better scars, reducing costs, reducing patient hospitalization, and enhancing surgical procedures.

*P.063*

**Pediatric Burns Treated on an Outpatient Basis with Excellent Results with Epifast Cultured Skin Graft**

**Alfonso Masse Sanchez**
  Imss

**Introduction**: Burns in pediatric patients are very common. In Mexico, it is estimated that every minute, 10 children under 5 years of age are burned. Additionally, management in those cases that do not meet hospitalization requirements is very difficult.
**Objective**: We aimed to provide adequate, fast, and safe treatment to pediatric patients with first- and second-degree burns that can be managed in outpatient clinics, for which we used Epifast cultured skin grafts.
**Methodology**: There are several cases of pediatric patients who were managed on an outpatient basis because, in their case, they did not meet the requirements to be hospitalized. In some cases, the patients underwent anesthesia and after surgery were discharged; in other cases, the Epifast was placed, fastening it with a micropore and bandage and immobilization, and the patients were discharged with the indication of recovering at home.
**Results**: After 8 days, they returned to the outpatient clinic and the epiplasty was removed from the wound, with total epithelialization in most cases. In adolescent patients, management was more difficult, requiring the two or three applications of Epifast.
**Discussion**: Epifast shortens the recovery time of burns, yielding a good-quality epithelium and with a single procedure in most cases (cooperating patient); in cases of burns that were already complicated, poor cooperation requires two to three applications.
**Conclusions**: The management of first- and second-degree burns in pediatric patients with Epifast cultured skin grafts is an excellent option because excellent results were obtained with a single procedure, leaving no esthetic or functional problems, reducing discomfort, and no reported rejection or infection complications.

*P.064*

**Decellularized and Lyophilized Fish Skin as a Skin Substitute for the Treatment of a Major Burn**

**Alfredo Cordova ^1^, J. C. Chen ^1^, Samuel Miller ^2^, Margaret Heller ^1^ and Alfredo Cordova ^1^**
  ^1^  The Ohio State University Wexner Medical Center
  ^2^  Yale University School of Medicine

**Objectives**: Biologic and synthetic skin substitutes are commonly utilized to enable the development of an optimal wound bed for grafting, to obtain a more natural neo-dermis, enable excellent re-epithelialization, or just for temporary wound coverage. The ultimate goal is to achieve an ideal skin substitute that provides effective and scar-free wound healing. Decellularized and lyophilized North Atlantic cod fish skin is a promising alternative. Its efficacy was tested on a major burn.
**Methods**: A 28-year-old male with history of schizophrenia presented after experiencing a psychotic episode that led him to pouring gasoline on himself and setting himself on fire. He suffered 93% TBSA full-thickness burns. after initial resuscitation, within 36 h, he underwent fascial excision and resurfacing with allograft, initially of his anterior trunk and bilateral lower extremities. These areas were subsequently exchanged for fish skin, then resurfaced with widely meshed (6:1) split-thickness autograft and cultured epithelial autograft (CEA).
**Results**: Xenograft integration and adequate granulation tissue was evidenced in 95% of the surface area as early as 10 days after application of the product. This was considered optimal for resurfacing. Skin coverage with widely meshed STSG and CEA revealed a >75% skin graft.
**Conclusions**: Fish skin may represent an excellent alternative for wound coverage and to enhance the for mation of the optimal wound bed for grafting. Further experience may be added to replicate these findings and to prove the alleged benefits of omega 3 and 6.

*P.065*

**Regenerative Medicine Approaches after Enzymatic Debridement**

**Bong-Sung Kim ^1^, Mauro Vasella ^1^, Andre Barth ^1,2^, Riccardo Schweizer ^1^, Matthias Waldner ^1^, Jan Plock ^1,3^, Nicole Lindenblatt ^1^ and Pietro Giovanoli ^1^**
  ^1^  Department of Plastic Surgery and Hand Surgery, University Hospital Zurich
  ^2^  Clinic for Plastic, Reconstructive and Handsurgery, Burn Care Center, Hospital Nuremberg South, Paracelsus Medical University
  ^3^  Plastic Surgery and Hand Surgery, Kantonsspital Aarau AG

**Objectives**: The enzymatic debridement of burn wounds is a standard procedure for burn eschar removal. Upon the selective enzymatic digestion of thermally injured tissue, the choice with regard to subsequent treatment, however, is still subject to debate. Mid-dermal wounds in particular present a challenge for classic burn surgery approaches, i.e., excision and split-thickness skin grafting, and clashing with more conservative approaches.
Regenerative medicine is an innovative and developing field in science and medicine that fosters autologous reparative functions. Various treatment strategies have evolved over the years which are primarily used in cosmetic and reconstructive surgery. Surprisingly, modern regenerative medicine techniques are rarely applied for burn wounds, even though burn patients may significantly benefit from minimally invasive treatment modalities.
In the present study, the feasibility of regenerative medicine techniques, including fat progenitor cells (stromal vascular fraction, SVF) and growth factor concentrates (platelet-rich fibrin, PRF), on enzymatically debrided wounds were investigated.
**Methods**: The study was conducted at the Burn Center of the University Hospital Zurich. The SVF was isolated through a semi-automated system with lipoaspirates harvested from non-injured areas. Additionally, PRF was harvested from peripheral venous blood through standard kits. Both were mixed and applied on mid-dermal burn wounds 24–48 h after enzymatic treatment with Nexobrid®. The outcomes were analyzed retrospectively.
**Results**: A total of seven patients (four male and three female: average age 47.71 years) were treated with the outlined technique and evaluated to date. Despite complete healing in four patients, three patients required additional tangential excision with subsequent split-thickness skin grafts (STSGs). No long-term risks or complications were observed.
**Conclusions**: In theory, Nexobrid® maintains a feasible matrix, similar to scaffolds used in tissue engineering approaches, for progenitor cells and regenerative factors. Despite excitement about the great potential in regenerative medicine, clear understanding and correct decision-making is pivotal.

*P.066*

**Knowledge and Assessment of the Hospitalization–Operating Room Transfer Sheet in a Burn Unit**

**Desiree Torres Andrés and María José Cano Carmona**
  Institut Catala de La Salut, Hospital Vall d’Hebron

**Objectives**: The transfer of patients from one service to another is a critical process linked to an increased possibility of errors in patient safety. These errors are associated with the communication process between the professionals involved. The objective of this study was to evaluate the knowledge of standardized tools already implemented and to propose a plan to improve it.
**Methods**: This was a cross-sectional descriptive study. An anonymous survey was sent to all nurses who regularly worked in the burn unit of a tertiary care hospital. Information was collected about seniority in the unit, work shift, level of knowledge, and the use of a standardized data collection sheet, as well as proposals for improvements. It was estimated that a minimum 50% response rate to the survey was necessary. The results were assessed globally and according to work shift.
**Results**: In total, 24 of the 38 regular nurses in the unit answered (64%); of these, 19 (79.2%) worked the day shift and 5 worked the night shift (20.8%). The median number of years working in the unit was 12.5 years (13 years day shift vs. 7 years night shift).
All respondents (100%) answered knowing the registration sheet, and 95% used it very frequently, with 87.5% of the respondents considering it useful–very useful. In relation to their assessment, 83% of the respondents considered that it is an adequate for m of communication (100% day shift vs. 60% night shift); 79.2% considered that it favors patient safety (95% day shift vs. 60% night shift); and 75% (89% vs. 40%) considered that it facilitates follow-ups. As improvements, it was recommended to add information on blood bank bracelets, the medication received, PCR results, the type of anesthesia, time of administration, and not being included in the usual computer records, which were considered limitations.
**Conclusions**: This standardized for m is well-known and implemented in intensive care units, although there are differences according to work shift in relation to its assessment, being better valued by workers of the day shift. It is necessary to update it, adding information that is considered useful and should be included in the unit’s computer records.

*P.067*

**Burns in the Elderly**

**Hana Fredj, Souheila Ben Massoud, Amel Mokline, Sarra Temani, Manel Ben Saad, Bahija Gasri, Imen Jami and Amen Allah Massadi**
  Burn Intensive Care Unit, Traumatology and Burn Center

**Introduction**: Admissions of elderly patients to intensive care units are increasing, in parallel with the aging of the general population.
This population is characterized by the frequency of comorbidities that aggravate the prognosis. Outcomes following burns in this special group are poorly studied.
The aim of our study was to assess the incidence and prognosis of burns in elderly patients admitted to intensive care burn departments.
**Patients and Methods**: This was a retrospective, descriptive study, conducted in intensive burn care departments in Tunis over a period of 26 months (January 2020–February 2022). Patients aged over 65 years old were included. Demographic, clinical, therapeutic, and evolutionary data were collected and analyzed.
**Results**: during the study period, total burn admissions were 924; 52 were aged over 65 years old. The mean age was 77 years (65–99). The sex ratio was 0.57. All patients presented with comorbidities. Twenty patients were diabetic (38.5%), and twenty-one were hypertensive (40%). Five patients were followed for dementia or Alzheimer’s disease (10%). Thirteen patients depended on a third person (25%). Burns occurred at home in the majority of cases (83%), and were mainly flame burns (80%). The mean total body surface area burned was 26%, and the burns were mostly deep (71%).
The evolution was marked by the occurrence of severe complications such as nosocomial infections in 56% of cases (*n* = 29), and thromboembolic events in four patients. Ventilatory support was required in 58% of cases. The average duration of ventilation was 9 ± 7 days. The mean length of stay was 9 ± 7 days. The mortality rate was 56% (*n* = 29), double the average mortality recorded in our department.
Multivariate analysis revealed predictive factors for mortality: SCB ≥ 20% (*p* = 0.000), the use of mechanical ventilation (*p* = 0.000), and the occurrence of sepsis (*p* = 0.000).
**Conclusions**: Burns in the elderly mainly occur at home and concern people with comorbidities. Regardless of their extent, their management is fraught with severe complications leading to high mortality.

*P.068*

**Efficiency of Enzymatic Debridement (Nexobrid^®^) and the Association with Other Different Surgical Techniques in Deep Burn Wounds—Personal Experience**

**Mihaela Pertea ^1,2^, Ștefana Luca ^1,2^ and Oxana-Mădălina Grosu ^1,3^**
  ^1^  Grigore T Popa, University of Medicine and Pharmacy
  ^2^  Department of Plastic Surgery, Reconstructive Microsurgery and Burn Unit ‘‘Sf. Spiridon’’ Emergency Hospital
  ^3^  Elytis Hospital Hope

**Objectives**: Enzymatic debridement using bromelain-enriched enzyme mixture (NexoBrid™) products is effective in deep burn debridement, keeping the unaffected areas intact. It can be associated with grafting with split-thickness skin grafts or micrografting (Meek technique) in the case of large areas of burns with minimal donor areas.
**Methods**: We studied a group of 23 patients (10 women and 13 men) with burns with an area between 10% and 60% TBSA. In all the cases, we used enzymatic debridement in the first 48 h, on a surface between 5% and 15% TBSA maximal surface, respecting the indications of the product. It was used for different anatomical regions: upper limbs in 8 cases, anterior thorax and abdomen in 10 cases, dorsal face of the legs in 3 cases, and 2 cases in the calves. In all the cases, enzymatic debridement was used on the deep burn area.
**Results**: The use of enzymatic debridement in the thoracic limbs was followed by secondary epithelialization without the need for split-thickness skin grafts. In all cases where it was used on the abdomen and anterior thorax, enzymatic debridement was followed by the application of split-thickness skin grafts on an area between 25% and 80% of the previously enzymatically debrided surface or micrografts harvested with the Meek technique to reduce the donor surface area and blood loss.
**Conclusions**: Enzymatic debridement is an alternative to surgical debridement that provides speed, tissue selectivity, and safety. Bromelain enzymatic debridement appears to be superior to surgical debridement in terms of its ability to preserve healthy tissues (avoiding blood loss during surgical burn excision) and graft donor sites, with associated morbidity, better outcomes, and the early mobilization of patients. The use of NexoBrid™ shows a better aesthetic appearance of scars compared with those obtained after surgical debridement. The associations of these two types of treatment can be considered in extensive burns of variable depths. We also observed that the early use of NexoBrid™ in deep, circular burns of the limbs could prevent the development of compartment syndrome; however, further studies are necessary for the confirmation of this hypothesis.

*P.069*

**Analysis of a Cohort of Patients Affected by Drug-Induced Toxicodermia Admitted to a Burn Unit**

**Blanca Guembe Zabaleta, Ángel Guillermo Arévalo Bernabé, Jacinto Baena Caparrós, Jordi Serracanta Domènech, Pilar Lalueza Broto, Laura Gómez Ganda, Pablo Sánchez Sancho and Joan Pere Barret Negre**
  Hospital Universitari Vall Hebron

**Objectives**: Stevens–Johnson syndrome (SJS) and toxic epidermal necrolysis (TEN) are severe mucocutaneous reactions, frequently occurring due to an iatrogenic cause. These patients are usually admitted to burn units, due to severe epidermal detachment and the risk of experiencing multi-organ failure.
The aim of this study was to analyze the characteristics of a cohort of patients diagnosed with iatrogenic SJS (affected total body surface area, TBSA, <10%), SJS/TEN (TBSA 10–30%), or TEN (TBSA >30%), as well as their prognostic, mortality, and drug attributable causality.
**Material and Methods**: This was a retrospective study conducted in a tertiary hospital between January 2010 and December 2021. Adult patients admitted to the burn unit due to iatrogenic SJS, SJS/TEN, or TEN were selected.
The prognosis was measured by a Score of Toxic Epidermal Necrosis (SCORTEN), which estimates the mortality rate (MR) based on patient parameters. The ALDEN algorithm (AA) was used to study the causality of the potentially implicated drug; a score greater than 4 suggested that the drug is probably the cause.
Biodemographic, analytical, and clinical data were collected, performing a descriptive univariate analysis with medians and interquartile ranges.
**Results**: A total of 37 patients (66.7% men) were included (3 SJS, 4 SJS/TEN, and 27 TEN), with a median age of 62.1 (42.7–73.9) years. In seven patients (19.4%), the glomerular filtration rate at admission was <30 ml/min. As significant pathological history, five had autoimmune diseases and four had solid organ neoplasia.
The average percentage of maximum TBSA affected was 37.5% (23.65%–60%), with mucosal affection in 80.6% of patients (29).
Mortality was 19.4%: six patients with TEN (22.2%) and one with SJS/TEN (20%) died.
Beta-lactam antibiotics were the most frequent cause (11 patients, 30.5%), followed by allopurinol (6 patients, 17%).
In 75% of patients (27), the AA indicated probable (13) or very probable (14) causality.
**Conclusions**: Mortality in our population is lower than expected prognosis according to the SCORTEN scale, and it is also lower than that described in the literature (>30%).
The most frequent causative agents in our cohort (beta-lactams, allopurinol, and antiepileptics) are drugs known to be potentially causative of severe epidermolysis.
According to the AA, in three-quarters of cases, it is to be expected that the drugs studied were responsible (probably or very probably) for the mucocutaneous reactions.

*P.070*

**Ten Years’ Experience with Colistin for MDRGN Strains of Burn Centre of Turin: Its Impact on Survival and CRRT Requirement**

**Anna Pensa ^1,2^, Filippo Mariano ^3,4^, Valeria Malvasio ^5^, Nadia De Petris ^6^, Giacomo Fucale ^7^, Fabrizio Gennari ^4^, Luigi Bianone ^3,4^ and Maurizio Stella ^2^**
  ^1^  Surgery Department, University of Turin, Italy
  ^2^  Burn Center and Plastic Surgery, Department of General and Specialized Surgery, City of Science and Health, CTO Hospital, Torino, Italy
  ^3^  Department of Medical Science, University of Torino
  ^4^  Nephrology, Dialysis and Transplantation U, Department of General and Specialized Medicine, City of Science and Health, CTO Hospital
  ^5^  Department of Pediatric General Surgery, City of Science and Health, Regina Margherita Childrens Hospital
  ^6^  Anesthesia and Intensive Care 3, Department of Anesthesia and Intensive Care, City of Science and Health, CTO Hospital
  ^7^  Laboratory of Microbiology and Virology, City of Science and Health, Molinette Hospital

**Objectives**: Multidrug-resistant Gram-negative (MDRGN) bacterial infections remain the leading cause of mortality in burn patients who are critical ill. Recently, researchers have developed new alternative drugs against MDGRN bacteria, although colistin therapy continues to play a key role as salvage therapy. In this retrospective study, we described correlations between colistin therapy, survival, and renal replacement therapy (CRRT).
**Methods**: From January 2008 to December 2017, we investigated 168 burn patients infected by MDRGN bacteria (A. baumamnni, P. aeruginosa, and K. pneumoniae): 133 patients were treated with colistimethate sodium (loading dose 9.0 × 10^6^ IU, maintenance dose 4.5 × 10^6^ IU BID), and 35 patients were treated with other antibiotics.
In the multivariate analysis, we analyzed the role of demographic characteristics, hospital stay, total body surface area (TBSA), Revised Baux score (RBS), Charlson comorbidity score, mechanical ventilation, need for CRRT, the duration and dose of colistin on in-hospital mortality, and CRRT requirements. To investigate the impact of colistin therapy on renal function, we only considered surviving patients who completed the colistin therapy.
**Results**: In the colistin-treated group the survival and CRRT requirement rates were 62.4% (83/133) and 39% (53/133), respectively, whereas in the other antibiotic-treated group, the survival and CRRT requirement rates were 88.6% (31/35) and 9.7% (3/35), respectively. The degree of burn, the TBSA, the RBS, the mechanical ventilation, the CRRT requirement, and the mortality rates were higher in the colistin-treated group. In the 83 survivors of the colistin-treated group, 64 patients (77.1%) exhibited normal renal function, and 19 required CRRT, without any difference about the colistin dose and the baseline characteristic. In these 64 patients, the median daily dose of colistin was 9.0 × 10^6^ and the median cumulative dose was 99.0 × 10^6^ IU. Referring to the cumulative dose, no differences in creatinine value and baseline features were found between patients treated with a low dose (<99.0 × 10^6^ IU) or high dose (>99.0 × 10^6^ IU).
**Conclusions**: In burn centers, MDRGN bacterial infections are always a challenge which require early diagnosis and a target therapeutic approach. Colistin therapy is still a valid choice especially for severely burn patients with associated higher mortality and renal injury. Short therapy with an appropriate cumulative dosage of colistin was associated with clinical success without any significant damage on renal function.

*P.071*

**Burns in Elderly Patients over 60: Quantitative Analysis of Published Data**

**Christian Smolle ^1^, Fredrik Huss ^2^ and Lars-Peter Kamolz ^1^**
  ^1^  Division of Plastic, Aesthetic and Reconstructive Surgery, Department of Surgery, Medical University of Graz
  ^2^  Burn Center, Department of Plastic and Maxillofacial Surgery, Uppsala University Hospital

**Objectives**: Along with increasing life expectancy in high-income countries, an increase in burn incidence in the elderly population has been noted. Simultaneously, outcomes after burn injuries have significantly improved due to advances in burn treatment. Little is known about the impact of these developments on elderly burn patients. The aim of this review was to shed light on how burn mortality evolved in patients above 60 throughout the past 25 years.
**Methods**: The PubMed and Medline databases, and the journals “Burns” and “Journal of Burn Care and Research” were searched between 27 August and 1 September 2019 using the keywords “burn” and “elderly”. Research articles describing an age group 60 published from 1995 onwards were included into quantitative analysis if the following data were available: number of patients, mean age (MA), mean%TBSA and mortality rate (MR). Studies were grouped according to the minimum age of inclusion (MAI) on the one hand, and by publication date on the other (cutoff 2010) and weighted (w) means for MA,%TBSA, and MR were calculated.
**Results**: of initially 306 studies, 23 were suitable for quantitative analysis. The wMA of the total study population (*n* = 4111) was 75.6 years (y), w%TBSA was 16.4%, and wMR was 27.7%. When categorized by MAI, these values were 71.6y, 17.9 w%TBSA and 27.2% wMR for MAI ≥60 (7 studies, *n* = 1322, 32.2% of total); 75.6 y, 15.2 w% TBSA and 24.8% wMR for MAI ≥65 (11 studies, *n* = 1851, 45% of total); 80.3 y, 14.2 w% TBSA and 33.9% wMR for MAI ≥70 (2 studies, *n* = 644, 15.7% of total); 81 y, 23% TBSA and 47% MR for MIA ≥75 (1 study, *n* = 201, 4.9% of total); 85.5 y, 21 w% TBSA und 22.5% wMR for MIA ≥80 (2 studies, *n* = 71, 1.7% of total) and 94 y, 9% TBSA and 27% MR for MIA ≥90 (study). Weighted MA, w% TBSA, and wMR of studies published before 2010 (11 studies, *n* = 1712) were comparable to those published thereafter (12 studies, *n* = 2399): 75.2 vs. 75.8 years, 18.9 vs. 14.6%TBSA, 29.4% vs. 26.5% MR.
**Conclusions**: With increasing age, MR in the burn population ≥60 also increased, although%TBSA remained fairly stable. MR, along with%TBSA, declined slightly when comparing recent and non-recent studies. The majority of studies (18/23) included patients ≥60 or ≥65 years of age, although only a paucity of studies focused on those aged ≥70. Bearing in mind increasing life expectancy, future burn research should focus increasingly on elderly.

*P.072*

**Laser Doppler Imaging (LDI) Used in Children with Burns**

**Ianthe Thaels, Laura Monten and Tuur Frederickx**
  Uz Leuven

**Background**: for more than 10 years, laser Doppler imaging (LDI) has been used to assess the severity of burns at the university hospital burn centre in Leuven. This technology is not only used in adults, but has also proven its value in children with burns. To achieve a reliable and stable image in children, sedation is often required. Therefore, careful evaluation of the added value of the LDI is in proportion with the possible complications of sedation. In our center, sedation and general anesthesia of a child is performed under the supervision of an anesthesiologist under full ASA monitoring.
**Objectives**: Evaluate whether the results of LDI in children with burns corresponds to the duration of the wound healing, and can predict the need for surgery.
**Methods**: Performing an LDI scan in child with burns. Based on the results of the LDI scan, a wound care plan (e.g., which dressing should be used) and timeline can be defined. It was checked whether the proposed schedule corresponded to the actual schedule.
**Results**: An LDI scan was performed on a 15-month-old baby with scald burns on day 3 post-accident. A less perfused tissue area was visible on the LDI scan. To cover this area, the patient received a full-sheet skin graft on day 23. Another example is a 12-month-old girl with contact burns on both hands. In this case, the LDI scan showed that the wound should be healed within two weeks. The result of the LDI scan corresponded to the actual time the wound had healed.
**Conclusions**: The use of an LDI scan on children certainly has an added value. The results of LDI scans correspond to the final duration of wound healing and/or whether surgery is necessary. This enables a clearer treatment plan to be drawn up with a possible change in treatment, allowing for outpatient follow up. It also helps us to explain the timeline and treatment to the parents.

*P.073*

**Surgical Management of a Paediatric Patient with a 90% SCQ by Electrical and Deflagration Burns with the Support of Cryopreserved Cultured Epidermal Allograft, Epifast^®^ at 5 Years of Evolution**

**Maria Zulema Cantú-Cantú, Oscar J. Ortega-Saucedo and Paloma Vázquez-Balboa**
  Hospital Pediátrico De Tacubaya

**Objective**: Show the management of paediatric clinical case with 90% SCQ treated with cryopreserved cultured epidermal allograft, and its benefits in long-term results.
**Methodology**: Under the principle that the management of burned patients is multidisciplinary, we present the case of a paediatric patient who suffered electrical burns plus deflagration in 90% of SCQ; the patient required four surgical events. In the first surgery, the patient was covered with a cryopreserved cultured epidermal allograft, and the subsequent surgery was performed with a serial tangential excision, harvesting, and application of mesh and insular grafts. The cryopreserved cultured epidermal allografts were applied over mesh and insular grafts, and over donor areas to promote graft integration and epithelialization.
**Results**: The patient was discharged after 61 days (9 w) of hospitalization, and subsequently required outpatient surgery to release the armpit flange. Currently, 5 years later, the patient shows a good evolution and acceptable scars without motion sequelae.
**Conclusions**: The use of cryopreserved cultured epidermal allografts promotes the epithelialization of superficial second-degree burns, and similarly promotes epithelialization in inter-areas and the integration of mesh and insular grafts, reduces the epithelialization time in donor areas, decreases morbidity and costs by shortening the days of hospital stay, and the long-term result is aesthetically and functionally acceptable. The high incidence of burns in developing countries represents a challenge in their management, as well as a public health problem. The use of a cryopreserved cultured epidermal allograft, epifast®, has been implemented in our hospital as a temporary biological dressing for the management of burns resulting in a decreased morbidity and costs, as well as acceptable long-term results.

*P.074*

**Use of Novosorb BTM in Combination with SSG in MEEK Technique for Covering Chronic Burn Wounds at a Child**

**Nicos Marathovouniotis ^1^, Paul Fuchs ^2^, Rebecca Pohle ^1^ and Tobias Klein ^1^**
  ^1^  Ped. Surgery and Ped. Urology, Burn Centre for Children, Children’s Hospital of Cologne, Amsterdamer Str
  ^2^  Department of Plastic Surgery, Hand Surgery, and Burn Center, University of Witten/Herdecke, Cologne-Merheim Medical Center

**Objectives**: Demonstrate the healing process of chronic burn wounds of a 5-year-old girl after a gas explosion injury. The patient was admitted in our hospital 10 months after the accident and exhibited hypergranulation tissue in 20% TBSA involving the breast, abdomen both axilla, both arms, and the neck. A sternomental scar contracture was present from the chin to the chest, impairing the movement of the neck. Scars without functional impairment existed on the face and both legs.
**Methods**: Transverse incision of the skin contracture to release tension in the neck was necessary to make management of the airways possible.
Debridement of the wound with VersaJet and the application of NPWT (negative pressure wound therapy) 100 mm Hg. Antibiotics were given according to the swab tests (day 4 of treatment).
We used a biodegradable temporizing matrix (Novosorb BTM) in combination with a silver wound dressing (Silverlon), as a local antimicrobial wound dressing and NPWT 100 mm Hg to secure the position of Novosorb BTM (day 8 of treatment).
NPWT was changed every 2–3 days because of tightness problems in the OR.
After delamination of Novosorb BTM, autologous SSG without expansion at the neck and axilla, SSG in MEEK technique for the other wounds was applied (day 25 of treatment).
The donor sites were the scalp and the right thigh. The aim was to use non-expanded SSG for critical sites such as the neck and axilla. We chose the Meek technique (1:4) because of the inadequate donor sites and for the better expansion of the SSG.
**Results**: The take-up rate was about 90% (day 39 of treatment).
Hypergranulation tissue was visible at the edges of the grafted wound near the old scars, especially the axilla and elbow.
Complete closure of the wounds was achieved on day 78.
**Conclusions**: Novosorb BTM, as a synthetic scaffold, helps the healing of large burn wounds, even in cases of a chronic state with superinfection (MRSA, pseudomonas, achromombacter).
As a synthetic material, it minimizes the risk of immune reactions or the transmission of diseases.
There is no other scientific article prescribing the treatment of burn wounds with Novosorb BTM in combination with SSG and the Meek technique.

*P.075*

**Use of Enzymatic Debridement in Paediatric Population. Experience in a Spanish Tertiary Center**

**Leire Aparicio Elizalde, Patricia Martin Playa, Elvira Morteruel Arizkuren, Naroa Cabrera Escondrillas, Paula Rodriguez Ruiz, Irene Gonzalez Alaña and Juan Jose Garcia Gutierrez**
  Hospital de Cruces (Baracaldo, Vizcaya, Spain)

**Objectives**: Nexobrid^®^ has proven to be an effective, rapid, and selective debriding agent in the treatment of adult patients with deep partial and full-thickness burns. However, its use in paediatric population is still beyond the manufacturer’s guidelines. We present our experience in the use of Nexobrid^®^ in a paediatric population in a tertiary center.
**Methods**: We performed a retrospective observational review including all paediatric patients treated with Nexobrid^®^ between June 2019 and December 2021, aged 6 months to 16 years old. We included demographic data,%TBSA, burn aetiology, complications, number of surgeries, the type of analgosedation used for debridement, and the length of stay.
**Results**: Enzymatic debridement was carried out in a total of twelve children. Two of them required more than one application due to total burn extension or insufficient total application time. Further surgery was required in 58.3% after debridement for autografting because of the depth of the burns. They all were treated at bedside at the Paediatric Intensive Care Unit under regional (*n* = 4) or general anaesthesia (*n* = 8). The only adverse event was bleeding requiring transfusion after enzymatic debridement in three patients. No other adverse effects were registered.
**Conclusions**: In our experience, enzymatic debridement with Nexobrid^®^ is a safe and effective procedure in burned paediatric patients under the same indications as in adult patients and can be considered as a standard of care in the management of acute burns in children as long as it follows adequate and multidisciplinary protocols for its use off-label, adjusting local and global management and analgosedation protocols to burn extension, anatomic location, and patient age.

*P.076*

**Spray-On Skin^®^ in the Management of Partial-Thickness Burns in Children: Findings of the BRACS Trial**

**Anjana Bairagi ^1,2^, Zephanie Tyack ^1,3,4^, Roy Kimble ^1,2,4^, Dimitrios Vagenas ^5^, Steven M. McPhail ^3,6^ and Bronwyn Griffin ^1,2,7^**
  ^1^  Centre for Children’s Burns and Trauma Research, Centre for Children’s Health Research and Pegg Leditschke Children’s Burns Centre, Queensland Children’s Hospital, Children’s Health Queensland Hospital and Health Service
  ^2^  Burns Trauma Research, Centre for Children’s Health Research, Queensland University of Technology
  ^3^  Australian Centre for Health Service Innovation and Centre for Healthcare Transformation, Queensland University of Technology
  ^4^  The University of Queensland
  ^5^  Research Methods Group, Queensland University of Technology
  ^6^  Metro South Hospital and Health Service, Clinical Informatics Directorate
  ^7^  National Health and Medical Research Council, Centre of Research Excellence—Wiser Wound Care, Menzies Health Institute of Queensland, Griffith University

**Objectives**: Spray-on Skin^®^ is an autologous skin cell suspension comprising keratinocytes, melanocytes, and fibroblasts, and has been in clinical use for almost three decades. However, little is understood of the re-epithelialisation time of small- to medium-sized partial-thickness thermal injuries treated with Spray-on Skin^®^ in children. The Biobrane^®^ RECELL^®^ Autologous skin Cell suspension and Silver dressings (BRACS) Trial evaluated the time to re-epithelialisation of paediatric partial-thickness thermal injuries.
**Methods**: All children aged ≤16years presenting to the study site with ≥5% total body surface area within 48 h of injury were included. Children were randomised to silver dressings, Spray-On Skin^®^ or Biobrane^®^. Measured outcomes included the time to re-epithelialisation (primary), pain, itch, treatment satisfaction, scar-specific health-related quality of life at each dressing application up to the primary endpoint of ≥95% burn wound re-epithelialisation, long-term follow-up to 12 months post-injury, and health resource utilisation.
**Results**: Twenty-two children were assigned silver dressings (*n* = 8), Spray-On Skin^®^ (*n* = 7) and Biobrane^®^ only (*n* = 7). The median re-epithelialisation time was 12 days for silver dressings (IQR 3.7–20.3), 12 days for Spray-On Skin^®^ (IQR 5.6–18.4), and 14 days for Biobrane^®^ (IQR 6.3–21.7). In addition, only one wound infection and no sepsis and zero requirements for split-thickness skin graft were reported in the Spray-On Skin^®^ group when compared with the other intervention groups. Secondary outcomes and health resource utilisation are presented.
**Conclusions**: The time to re-epithelialisation of partial-thickness thermal injuries managed with Spray-On Skin^®^ was the same as the control group (silver dressings) and faster by two days when compared with the Biobrane^®^ group. In addition, paediatric thermal injuries treated with Spray-On Skin^®^ were associated with the fewest infections compared with other groups, no sepsis, no requirements for skin graft, and the least impact on scar-specific health-related quality of life. A larger cohort is required to better understand this evidence.

*P.077*

**Predictors of the Long-Term Quality of Life of Paediatric Patients after Non-Severe Burn Injuries**

**Amira Allahham**
  Fiona Wood Foundation

Paediatric burns account for one-third of burn admissions in Australia; thus, understanding the quality of life outcomes after a non-severe burn in children is important. This retrospective cohort study describes three paediatric cohorts from Western Australia with burns covering less than 20% TBSA and characterises: (1) The quality of life outcomes measured using the Paediatric quality of life survey (PedsQL) at three months post-burn; and (2) The differences in scoring the psychosocial function between patients and parents during early recovery (~six months) and late recovery (>1 year). PedsQLs consist of a parent report and a patient report with a physical function domain (PF) and a psychosocial function domain (PSF). for the first aim, parent-report scores were significantly different between age groups (PF: *p* = 0.002, PSF: *p* = 0.001, respectively), burn cause (PF: *p* = 0.004, PSF: *p* = 0.005, respectively), and socioeconomic status groups for the PSF (patient: *p* = 0.015, parent: *p* = 0.032, respectively), and 16.46% of paediatric burn patients had critically low quality of life scores. The second aim showed that during early recovery, parents reported poorer PSF for younger children (*p* = 0.01), higher socioeconomic status (*p* = 0.05), and significantly different scores for female patients (*p* < 0.01). In the late recovery cohort, only the age at burn had an effect where parents had lower scores for older patients (*p* = 0.03). These data will enable health professionals to accurately assess patients’ quality of life to provide them with services that can aid in their recovery.

*P.078*

**SARS-CoV-2 Infection in Pediatric Burns: Experience of a National Referral Center**

**Raluca Tatar ^1,2^, Dan Mircea Enescu ^1,2^, Iulia Nacea ^2^, Cristina Stoica ^2^, Bogdan Prisecaru ^2^ and Maria Tomita ^2^**
  ^1^  Carol Davila University of Medicine and Pharmacy Bucharest
  ^2^  Grigore Alexandrescu Clinical Emergency Hospital for Children

**Objectives**: The COVID-19 pandemic is a health issue affecting people worldwide. Although SARS-CoV-2 infection has been less frequent and generally less severe in children, the occurrence of burn injuries with the subsequent alteration of the immune system could turn burn patients into a more vulnerable population. The numbers of pediatric burns and COVID-19 associations reported thus far are small. Thus, we consider it important to share the experience of our department in the management of pediatric burns with COVID-19.
**Methods**: We retrospectively reviewed the electronic database of the hospital, searching for patients hospitalized for burn injuries in the Plastic Reconstructive Surgery and Burns Department of the “Grigore Alexandrescu” Clinical Emergency Hospital for Children, Bucharest, who presented with concomitant SARS-CoV-2 infection confirmed by RT-PCR testing. The search was performed using classic ICD-10 codes T2*-T3* for burn injuries and the new U07.1 code (standing for confirmed COVID-19). The investigated period ran from February 2020 (month of the first confirmed COVID-19 case in Romania) to February 2022.
**Results**: during the defined period, we retrieved 21 cases of concomitant presence of burns in our pediatric population and confirmed COVID-19. The occurrence of the cases was in accordance with the national trend regarding the pandemic waves, with most of patients registered in September–November 2021 (Delta variant) and January–February 2022 (Omicron variant), The average TBSA was 23% (range 5–90%), and the major burn etiology was scalds. The patients were isolated in the COVID-19 ward and burn wounds were managed conservatively for 15 patients and required excision and grafting for 6 cases. for the SARS-CoV-2 infection, only one case required specific treatment (remdesivir); the rest of the cases only had the general supportive systemic treatment given for their burn injuries. None of the patients needed mechanical ventilation for the SARS-CoV-2 infection The hospital stay was mostly correlated with the burn etiology and extent and was not significantly affected by the concomitant infectious condition.
**Conclusions**: Concomitant COVID-19 infection might complicate the surgical and medical management of burn patients. Patients need to be isolated and healthcare staff must effectively use all PPE. However, this study has shown that despite burns, children developed mild-to moderate COVID-19 in the majority of cases. At the same time, it did not show an increased hospital stay caused by the association with SARS-CoV-2 infection.

*P.079*

**Gabapentin as a Potential Treatment for Anticipatory Anxiety in Burn Children**

**Yolanda Peña-López ^1,2^, Gemma Español-Martín ^2,3^, Ángel Guillermo Arévalo Bernabé ^4^, David Roca Pascual ^1^, Borja De Paz Vaquero ^1^, Alejandra Monte Soldado ^5^, Joan Balcells Ramírez ^1,2^ and Elena Arana Martín-Bejarano ^2,5^**
  ^1^  Pediatric Critical Care Department and Burn Center, Vall d’Hebron Barcelona Hospital Campus
  ^2^  Vall d’Hebron Research Institute (VHIR)
  ^3^  Department of Psychiatry, Vall d’Hebron Barcelona Hospital Campus
  ^4^  Pharmacy Department. Vall d’Hebron Barcelona Hospital Campus
  ^5^  Plastic Surgery Department and Burn Center, Vall d’Hebron Barcelona Hospital Campus

**Introduction**: Distress and anxiety are common experiences among burn patients, especially in children, as a result of injuries and psychological trauma related to accidents and first aid. Optimal analgesia, including high doses of clonidine (up to 5 mcg/kg/dose) along with non-pharmacological interventions designed to reduce task-specific anticipatory anxiety (i.e., dressing changes), usually help in pain anxiety responses in this population, but sometimes they cannot be completely managed. Additionally, these patients experience neuropathic pain and hyperalgesic elements and itching that can increase anxiety levels which is difficult to manage and significantly contributes to their suffering. This is especially challenging in children, who are at high risk of mood, eating and sleeping disturbances, and post-traumatic stress symptoms. Appropriate pain and anxiety management for children who have experienced an acute burn injury is necessary to improve patient outcomes and reduce potential morbidities. On the other hand, gabapentin has established efficacy in the reduction in burn-induced hyperalgesia and allodynia and also in anxiety disorders.
**Objective**: We report a case series of six pediatric patients who presented with anticipatory anxiety and were successfully managed with gabapentin.
**Methods**: There were four males and two females. The median age was 7 years old (22 months to 13 years). They were prescribed gabapentin in addition to standard analgesia and high doses of clonidine (3–5 mcg/kg/dose, and 10–12 mcg/kg/day). The starting dosage of gabapentin was 5 mg/kg/day, which it was considered adequate to improve symptomatology in three cases (two toddlers, one child) and it was increased up to 10 or 15 mg/kg/day in the other three (children).
**Results**: The use of gabapentin resulted in a rapid (24–48 h) reduction in irritability and the level of anticipatory anxiety before changing dressings. The medication was well-tolerated, with no severe adverse reactions. Clonidine and other analgesic decreases were observable in six of them.
**Conclusions**: This case series introduces the use of gabapentin as a potentially important therapy in the routine management of children following burn injury who presented with anticipatory anxiety. Further research is required to define the use of gabapentin in this specific setting.

*P.080*

**Time-Scheduled Oral Clonidine for Pain and Anxiety Management in Burn Children**

**Peña-López Yolanda ^1,2^, Ángel Guillermo Arévalo Bernabé ^3^, David Roca Pascual ^1^, Lourdes Ausín García ^4^, Rommy Rossich Verdés ^5^, Pilar Lalueza Broto ^3^, Zhan Qiao Lin Wu ^6^ and Elena Arana Martín-Bejarano ^6^**
  ^1^  Pediatric Critical Care Department and Burn Center, Vall d’Hebron Barcelona Hospital Campus
  ^2^  Vall d’Hebron Research Institute (VHIR)
  ^3^  Pharmacy Department, Vall d’Hebron Barcelona Hospital Campus
  ^4^  Pediatric Emergency Transport Service, Vall d’Hebron Barcelona Hospital Campus
  ^5^  Pediatrics Department and Burn Center, Vall d’Hebron Barcelona Hospital Campus
  ^6^  Plastic Surgery Department and Burn Center, Vall d’Hebron Barcelona Hospital Campus

**Introduction**: Lately, an increased use of oral clonidine has been described for pain and anxiety in pediatric patients with a good security profile. It also has been acquired as an analgesic drug in burned children prior to changing dressings or as a rescue therapy. Thus, the use of time-scheduled clonidine in those patients may represent a good balance between anxiety and pain control. Furthermore, its prescription at night can enhance children’s rest, and the benefits of nocturnal sleep can be translated into a reduced risk of mood, eating disturbances, and secondary wound lesions resulting from scratching. However, its use has raised safety concerns regarding haemodynamic instability, and some pediatric protocols even recommend against its introduction within the first 48 h because of it.
**Objective**: We evaluated the safety profile of clonidine as the main analgesic drug in hospitalized second-grade burn children involving <10% total body surface area (TBSA).
**Methods**: A retrospective study of burned children who received a time-scheduled prescription of oral clonidine between January 2018 and December 2021 was performed. This protocol consisted of the daily administration of 2 mcg/kg of oral clonidine twice: at 10 am (1 h before the change in dressings), and a nightly fixed dose at 11 pm. Additionally, an interspersed rescue clonidine (2 mcg/kg) was prescribed on demand within the next 6 h during the day or overnight for pain control and sleep. The morning clonidine dose before the change in dressings was increased the first 24–72 h up to 5 mcg/kg to achieve better control of pain/anticipatory anxiety according to the attending physician criteria.
**Results**: There were 335 children with second grade-burns admitted from 2018 to 2021. of them, 231 had a TBSA < 10% (56.3% males). Their age (mean, IQR) and hospital stay were 1.9 years [IQR 1.35–5.8] and 6 days [3,4,5,6,7,8,9], respectively. Among them, sixty children were initially admitted to the Pediatric Intensive Care Unit for monitoring and the first cure with debridation was performed under intravenous sedation, being transferred to the Burn Unit the next 24–48 h. The other 171 children were directly admitted to the Burn Unit. In both groups, the time-scheduled clonidine protocol was performed after the first aid. No clinically significant hypotension or bradycardia was observed.
**Conclusions**: The time-scheduled use of oral clonidine in second-degree burn children <10% TBSA seems to be safe.

*P.081*

**Heat Burn Injury in a Neonate: A Clinical and Surgical Challenge**

**Valeria Malvasio ^1^, Maria Grazia Cortese ^1^, Patrizia Magro ^1^, Elisa Zambaiti ^1^, Giovanni Montà ^2^, Gabriella Naretto ^3^, Serena Causi ^3^, Carola Marchetti ^4^, Sara Simona Racalbuto ^4^, Paola Imazio ^4^, Maurizio Stella ^5^ and Fabrizio Gennari ^1^**
  ^1^  General Pediatric Surgery, Regina Margherita Children’s Hospital
  ^2^  Pediatric Plastic Surgery, Regina Margherita Children’s Hospital
  ^3^  Rehabilitation Team of Pediatric Orthopedic Surgery, Regina Margherita Children’s Hospital
  ^4^  Department of Pediatrics Psychology, Regina Margherita Children’s Hospital
  ^5^  Burn Unit, CTO Hospital

**Objectives**: Neonatal burn injuries are rare and challenging. Immature immune systems and the fragility of thin skin, paucity of donor sites for wound coverage, and long-term complications make the clinical management and surgical treatment of burned newborns extremely difficult.
**Methods**: Case report: a 23-day-old female neonate presented as abdominal colic. Her father decided to utilize the white noise of a hairdryer to control pain and reduce crying time. Accidentally, the baby remained in contact with the heat of the hairdryer for several minutes, and she sustained a full-thickness burn to the face, left for earm, and hand. She was admitted on the same day to our Center, Regina Margherita Children’s Hospital in Turin, in December 2021.
**Results**: Due to the deep circumferential wrist burn, an escharotomy was performed at the arrival to the Emergency Department. In the acute phase, the baby was promptly managed by fluid resuscitation, analgesics, antibiotics, and initial conservative wound care. Initial surgical procedures, escharectomy to the for earm and hand and debridement to the face, were performed at day 5. At day 10, the face and upper extremity were covered with amniotic membranes and autologous skin grafts, respectively. Due to the depth of the burn, a second application of amniotic membranes to the face was performed on day 22. Complete healing was achieved after thirty-five days from burn injury. Physical therapy and splinting were promptly started. after wound healing, physical therapy was intensified, and at day 42, the first dye–laser therapy to control hypertrophic scars was performed. The baby was discharged after 48 days. Dye–laser therapy to the face was then performed every 30 days. Despite timely scar management, an important scar contracture of the wrist and hand is present. The first surgical intervention for scar release is planned. Psychological support to the parents and their involvement during physical therapy were mandatory throughout hospitalization, and are still ongoing.
**Conclusions**: This case report presents a challenging situation with a female neonate, with a deep burn to the face and upper limb. A multidisciplinary healthcare team of physicians and therapist is imperative to optimize functional, cosmetic, and psychosocial outcomes. A multistep approach consisting of neonatal intensive care, timely surgical indication, and prompt post-operative rehabilitation constitute a key points for the successful treatment in neonatal severe burns.

*P.082*

**Let us Eat out Tonight! A Review of Paediatric Burns Sustained Whilst in a Restaurant—Single Burns Centre Experience over 5 Years**

**Francesca Ghini ^1^, Mehul Thakkar ^2^ and Bartlomiej Bednarz ^1^**
  ^1^  North Bristol Nhs Trust
  ^2^  Glasgow Royal Infirmary

**Objectives**: Going out is a great occasion for parents and kids to enjoy some quality time together. Unfortunately, sometimes the new environment with hot food/plates can be dangerous, especially for younger populations. We wanted to investigate the incidence of paediatric burns sustained in the hospitality setting and their severity.
**Methods**: We searched our local burns centre database for all burn-related injuries sustained in pubs/restaurants (coffee shops were specifically excluded). We collected retrospective data covering a period of five years between 2015 and 2019. The reason behind the exclusion of 2020–2021 was because of the COVID-19 pandemic and its effect on outdoor dining. Data collected included age, mode of the injury, size, depth, location of a burn, and need for surgery/inpatient stay.
**Results**: Our burns centre covers a population of about four million people. We identified 23 patients (9 females and 14 males) with ages ranging from 5 months to 15 y 7 m (average 2 y 10 m, median 1 y). All the burns were superficial and partial-thickness in nature and were treated in outpatient settings (no need for admission or surgery). The average size of burn was 0.74% (min 0.01%, max 3%, and median 0.3%). The areas affected were mainly the fingers and hands (20/31 areas affected); however, the face, torso, feet, and lower limb were also affected. The most common mode of injury was a contact burn followed by scald (12 vs. 11). Two of the accidents in older patients happened whilst they were working at the restaurant. One child was injured by a waiter spilling food accidentally over the child. In the remaining cases, patients touched either a hot plate/food or spilled a hot drink.
**Conclusions**: Burns occurring at hospitality venues such as restaurants are not common, with the majority of patients being 2 years old or younger. The injuries were small and, in our series, superficial partial-thickness in nature, enabling burns to be treated in the outpatient setting. Our study provides a good basis for updating health and safety guidance in restaurants on how to be more vigilant of small children and how to educate/inform parents of the potential dangers. Additionally, it provides some background for medicolegal cases if litigation occurs.

*P.083*

**Analysis of the Management of Deep Second-Degree and Third-Degree Burns in Special Areas in Pediatric Patienst with Mesh Grafts and Placement Epifast**

**Claudia Berenice Hernández Valverde**
  Imssp

**Objective**: We analyzed the early management of pediatric patients with burns in special areas served in the burn unit from the pediatric intensive care unit of the V.F.N. Traumatology Hospital Instituto Mexicano del Seguro Social, Mexico City.
**Method**: All pediatric patients who had deep second-degree and third-degree burns in special areas, plus electrical or airway burns or more than 20% sct, admitted from intensive care units to the burn unit were included, between February 2020 and February 2022. Tangential excision was carried out from 8% to 20%, in addition to the start of the reconstruction. The tangential excision was performed with a Zimmer Dermatome, as well as the taking of thin grafts from 0.2 to 4 mm. Fixation with skin staples or simple nylon 4–0 points, occlusion with fractioned gauzes in the case of the hands or neck, and, if the case allowed, it in the face; however, due to orotracheal intubation and anatomy, the face was frequently left uncovered. Alloynjects (epifast) were from the hospital’s monthly supply. Part of the basic table. Follow-up was performed from 6 to 24 months and evaluated with the Vancouver scale (pigmentation, vascularity, and thickness).
**Results**: Patients grafted early with epifast (cultivated keratinocytes) on the grafts showed minimum functional sequels and reasonably acceptable aesthetic results. This method of immediate reconstruction was chosen because the preservation of life and the function of vital organs as priorities. Frequently, these children have, as part of the treatment, vasoactive amines at doses that affect the skin. We did not have early crops because they were not able to be surgically intervened several times a week, and the extension required early excision in the initial surgeries to improve the general condition and reduce biochemical factors of inflammation and necrosis. In these patients, the donor areas were limited and cultivated keratinocytes have proven to be accelerators in the recovery of burned patients.
**Conclusions**: The comprehensive management of burned patients includes priority attention to special areas; in serious cases, this is an option for reconstructive salvage which offers minimum scar sequelae with acceptable aesthetic results. The approach was reproducible and with low complexity.

*P.084*

**Follow-Up Study of Children Undergoing Autologous Skin Transplantation for Burns**

**Gergo Jozsa, Aba Lorincz, Angyalka Valik and Zsolt Juhasz**
  Department of Paediatrics, Surgical Unit, University of Pécs

**Introduction**: Deep partial and full-thickness burns require surgical treatment, with autologous skin grafting after necrectomy, which is the optimal way to achieve permanent coverage. Cosmetic outcomes can be assessed during long-term follow-up. The aim of this study was to examine the grafted and donor areas of children undergoing autologous skin grafting using different scar assessment scales to determine the severity of scars and the cosmetic outcomes.
**Patient and methods**: At the Surgical Unit of the Department of Paediatrics of the University of Pécs, between 1 January 2015 and 31 December 2019, 20 children were included in the study out of 44 children who underwent autologous skin transplantation. The authors assessed the results obtained using the Patient and Observer Scar Assessment Scale (POSAS) and the Vancouver Scar Assessment Scale. Cosmetic scores were scored by the patients’ parents and the examiner on the POSAS, whereas the Vancouver scarring scale was scored by the examiner.
**Results**: In the POSAS for the transplanted area, parents recorded a score of 5 or below in fourteen cases. In six children, the score exceeded 7, which is a significant deviation from normal skin characteristics. for the POSAS, the examiners scored the transplanted area of eight children as 1, five as 2, three patients as 3, and four more children as higher. for the Vancouver scar scale, the transplanted area was scored 0 in one case, 1 in four children, and 2 in seven children. Based on these results, the scores were good or acceptable in 12 children. for the POSAS, the donor area was described by the parents as 1 in eight children and 2 in five children. for the POSAS, the examiners rated the donor area of fifteen children as 1. On the Vancouver scar scale, the examiners scored the donor area as 0 in the case of 12 children, which means that it was almost identical to healthy skin; they scored it as 1 in three children, 2 in four children, and 6 in one child.
**Discussion**: There is a significant difference in the ways parents and examiners view scars. In the evaluation of the observer scale, the most critical variables for the area of skin grafted were relief and thickness. for the patient scale, in addition to colour, relief was the worst clinical characteristic as well. Better results were obtained when evaluating the donor area.

*P.085*

**A Simple Mnemonic, B.U.R.N.S., for Burns First Aid**

**Janna Joethy, Keith Koh, Kok Chai Tan and Chee Liam Foo**
  Sgh

**Objectives**: Burn injuries remain common worldwide. The authors felt that there is a need for increased public awareness on first aid burn treatment. The acute management of burns can improve eventual patient outcomes.
The authors devised a simple mnemonic that can be used in burns education for first aid treatment, intended to be taught to trained personnel, who will have first contact with these burn patients.
The aim of the study was to assess the viability of implementing this mnemonic, B.U.R.N.S, to facilitate first aid education for burns.
**Methods**: We presented this mnemonic as a poster to 30 full-time burn care medical professionals. Feedback was then obtained from this group of medical professionals and used to revise the mnemonic. The mnemonic was subsequently taught to 400 medical professionals, who were predominantly involved in the pre-hospital management of burns. They were then asked to reiterate the mnemonic to test the ease of remembering. Objective feedback was obtained with a 5-point scoring system.
**Results**: The results indicated a significant improvement in burn first aid knowledge after the implementation of the mnemonic, from a score of 3.67 to 4.77. The content was deemed to be appropriate and easy to understand and recall, and participants were able to reiterate the content, and will recommend this mnemonic to be used for teaching burns first aid.
**Conclusions**: The study results suggest that this B.U.R.N.S. mnemonic and visual aid is simple and easy to apply, especially for uniformed personnel, because these individuals may have the first contact with burns victims, and it is important for them to render the appropriate burns first aid treatment. Overall, burns first aid awareness and education can be improved with the implementation of this mnemonic and poster. We wish to also be able to do so on an international level when courses are conducted.

*P.086*

**Prevention of Fireplace-Related Burns: Based on a 5-Year Review of a Major Burn Center**

**Dmitry Shelepenko ^1^, Beatriz Jardim ^2^, José Miguel Azevedo ^1^, Gonçalo Tomé ^1^, Inês Catalão ^1^ and Luís Cabral ^1^**
  ^1^  Coimbra Hospital and University Centre (CHUC)
  ^2^  Public Health Care Unit—USP—ACES Pinhal Litoral

**Objectives**: Fireplace-related accidents are consistently a major cause of burns around the world. Although electric and gas heating have largely replaced the use of fireplaces in developed countries, they remain an important source of heating, especially in rural areas. The objective of this study was to show the population impact of burns in fireplaces, and to identify possible factors in which we can intervene to prevent these burns.
**Methods**: The authors analyzed the medical records of all patients admitted to the Coimbra Burns Unit, in Portugal, due to fireplace burns, between 1st January 2017 and 31st December 2021. These patient’s statistics were compared with the general population of the burns unit during the same time period. In the context of public health, we then identified factors that could help prevent this type of burns.
**Results**: during the 5-year sampling, out of 648 patients admitted for all causes of burns, 53 (8%) were due to fireplaces, (27 women and 26 men), and 75% of them were 65 or older. The mortality rate in this group was 26% (vs. 9% in general group); moreover, 23% of all deaths during this period were among fireplace burn patients. The average number of days of hospitalization was 31.4 days (vs. 21.4 in the general group).
We identified possible risk factors involved in these accidents:
  - Patient-related: Advanced age/medical comorbidities (epilepsy, cardiovascular diseases, orthopedic pathology, alcoholism, and diabetes) that lead to falls, difficulty in locomotion, and/or loss of pain/thermal sensitivity.
  - Space-related: Poor ventilation, excessive smoke (due to the burning of all types of waste, wet/green wood, and clogged chimneys), lack of place security (fireplaces at the same level as the floor, without grids, near flammable objects).
We suggest some measures that could be taken in prevention:
  - Education of patients about the dangers of fireplaces and inner recognition of physical limitations. When possible, let them be handled by younger and less physically limited people.
  - Periodic ventilation of the airspace, cleaning the chimneys, and only burning dry wood.
  - Removal of objects near the fireplace perimeter, and storing firewood in a separate room.
  - Installation of closed heat exchangers above the fireplace.
  - Use of alternative heating sources.
**Conclusions**: We were able to perceive that burns in fireplaces occur mainly in the elderly population, require longer hospital stay, and have a higher mortality rate. This knowledge must be shared with primary care doctors and the general population, so that we can adapt measures to prevent these accidents.

*P.087*

**Prevention of Burns in Older People; Development of the ‘Prevent Flame Burns’ Doorknob Hanger**

**Janneke Damen-Thissen ^1,2^, Annebeth Meij-de Vries ^1,3,4^ and Anouk Pijpe ^1,3,5,6^**
  ^1^  Burn Center, Red Cross Hospital
  ^2^  Department of Geriatrics, Red Cross Hospital
  ^3^  Association of Dutch Burn Centres (ADBC)
  ^4^  Paediatric Surgical Centre, Emma Children’s Hospital, Amsterdam UMC, Location AMC
  ^5^  Amsterdam UMC Location Vrije Universiteit Amsterdam, Department of Plastic, Reconstructive and Hand Surgery
  ^6^  Amsterdam Movement Sciences (AMS) Institute, Amsterdam UMC

**Objectives**: As a result of an ageing population and the trend for older people to live at home for longer, we suspect that the number of older people suffering from severe burns will increase. This supports the need for the prevention of burns in this rapidly growing and vulnerable group.
**Methods**: All patients aged 70 years or older who were admitted to the Burn Center of the Red Cross Hospital in Beverwijk, The Netherlands, between the end of 2008 and September 2019 were retrospectively included (*n* =2 38) from the Dutch Burns Repository (NBR-R3). This prospective registry includes patient, burn, and clinical characteristics which were analysed by means of descriptive statistics. Based on the retrieved burn injury mechanism information and our clinical geriatric experience, we developed a prevention tool together with communication experts and a patient representative.
**Results**: The most frequent causes of severe burns in older people are flames (*n* = 105/238, 44%), followed by scalds (*n* = 59/238, 25%) (Figure 1: causes of burns in older patients admitted to the Burn Center of the Red Cross Hospital in Beverwijk, The Netherlands). We observed a slight increase in the absolute number of burn patients aged 80 or older. From 2015 onward, for the first time, we observed patients of 90 years and older admitted to our Burn Center. In this group of the oldest elderly, 84% were women (*n* = 16/19) and 89% (*n* = 17/19) were living at home at the time of the accident. The ‘prevent flame burns’ doorknob hanger we developed includes information and images on risk factors and behavioural advice to prevent burns in older people (Figures 2 and 3).
**Conclusions**: The ageing population, as well as the Dutch political decision that supports living at home longer, increases the need for prevention of burns in this rapidly growing and vulnerable group. The ‘prevent flame burns’ doorknob hanger was developed in the context of the prevention of burns in The Netherlands (Figures 2 and 3). This doorknob hanger serves as a best-practice prevention tool example and is freely available for other countries in Europe.

*P.088*

**Home Burns: Prevention and Treatment. Corporate Training Course. The Experience of the Burn Center in Turin**

**Nadia Sandrone ^1^, Maria Giuseppa D’Onise ^1^ and Valeria Malvasio ^2^**
  ^1^  Burn Center, CTO
  ^2^  General Pediatric Surgery, Regina Margherita Children’s Hospital

**Objective**: Healthcare professionals represent a link between the population and the health environment. Corporate training courses on prevention and health promotion are mandatory to raise their awareness. The presented course identifies the major risks of burns present in the home environment, promoting changes in behavior and then in the general population.
**Method**: As Burn Center nurses, we decided to set up a course about the most frequent causes of domestic accidents that lead individuals to access our operating unit, according to the priority training objectives identified by the Regional Health System. The course has been active since November 2017 thanks to the Continuing Education Plan staff. The course, over the years, has been enriched by inorporating the experience of pediatric nurse colleagues from the Regina Margherita Children’s Hospital of Turin. Reporting the subjective perceptions of a burn patient, hospitalized at the burn center, in his own words, there is a fundamental part of the course to sensitize healthcare professionals on the changing identity of burn patients.
**Results**: Since 2017, we have trained 108 caregivers and 350 healthcare professionals (nurses, physical therapists, lab technicians, radiology technicians, and physicians). during end-of-course interviews, it was found that participants had poor behaviors in handling flammable materials and heat sources present at home. Therefore, it appears that there is a lack of attention to the prevention of domestic accidents due to old habits. The course is still ongoing.
**Conclusions**: We can affirm that this course leads each individual to analyze their personal experience, avoiding underestimating the risks that surround them, especially in the place they consider safest: their own home. It also stimulates each professional to take responsibility for themselves and others and to create new behavioral habits. The experience shared by a witness, who wears burn marks on her skin and on her soul, emotionally involves health professionals who claim to have a greater incentive to do prevention.
Let us Keep Our Skin Dear!

*P.089*

**Factors That Influence the Behaviour and Environment Leading to Severe Burns in Young Children: Implications for Burn Prevention**

**Eva Van Zoonen ^1^, Anne Soek ^2^, Anouk Pijpe ^3,4,5^, Margriet van Baar ^5,6^, Marianne Nieuwenhuis ^4,7,8^, Carine van Schie ^1^ and Annebeth Meij-de Vries ^3,5^**
  ^1^  Dutch Burns Foundation
  ^2^  Vu University
  ^3^  Rode Kruis Ziekenhuis
  ^4^  Association of Dutch Burn Centres
  ^5^  Amsterdam UMC
  ^6^  Maasstad Ziekenhuis
  ^7^  Martini Ziekenhuis
  ^8^  Hanzehogeschool

**Objectives**: Children under 5 years of age are admitted to Dutch burn centres more frequently than any other age group. To prevent burn injury in young children, prevention programs can encourage caregivers to take safety measures. The PRECEDE–PROCEED model of Green and Kreuter is a frequently used tool for the stepwise development of effective prevention programs. To be effective, prevention programs are based on a known aetiology. The aim of this study was, therefore, to identify factors that influence the behaviours of persons that have been involved in accidents that caused burn injury in children under 5 years of age and the environment where the accident took place.
**Methods**: In this qualitative study, phase 3 of the PRECEDE–PROCEED model was completed, in which influencing factors on the behaviour and environment are identified. Thus, interviews with caregivers were carried out until a saturation of topics was reached. A total of 30 interview were expected to be needed. Caregivers of children that were treated in a Dutch burn centre at ages of 0–4 years and who were identified as a risk group for sustaining burn injury were eligible. Interviews were transcribed and coded.
**Results**: Outcomes of the study involve influencing factors on the behaviour and environment. According to the PRECEDE–PROCEDE model, influencing factors are distinguished into (1) predisposing factors, including knowledge, attitudes, beliefs or values; (2) reinforcing factors, including the availability or accessibility of resources; and (3) enabling factors, including peer support or social support.
**Conclusions**: The outcomes of this qualitative study expand knowledge on the aetiology of severe burn incidents in children under 5 years of age in The Netherlands. The identified factors that influence the behaviour and environment can be used to determine strategies and policy for prevention programs aimed at risk groups.

*P.090*

**Dangers of Wearing Facial Mask with Facial Burns—Two Case Reports**

**Francesca Ghini, No Mehul Thakkar, Florin Panduru and Bartlomiej Bednarz**
  North Bristol NHS Trust

**Objectives**: The recent COVID-19 pandemic saw governments enforce mask-wearing as a preventive measure. Although Europe is now recovering from the latest wave and new variants seem less virulent, there is a chance that wearing face masks will be reintroduced at some point. Although there are some exceptions, the majority of people are required to wear a face mask. This can present a problem in some subgroups of plastic and burn surgery patients. We present two cases where such masks could potentially create a scar.
**Methods**: This study is case presentation based on medical notes and medical photographs. A literature review was performed by searching Medline with the terms “facial” [and] “cover” [or] “mask” [and] “burn”.
**Results**: We present a case of facial superficial partial-thickness sun burn, in a fit and well 14-year-old who went paddle boarding without any sunscreen for 4 h. This resulted in erythema on his arms and legs and face, but significant blistering around the posterior part of the ears where his mask was worn.
The second case was a 51-year-old female with a background of diabetes and hypertension. She presented with chemical burns to her left cheek following the accidental application of anti-wart cream consisting of 12% salicylic acid and 4% lactic acid. At the point of presentation, her burns were considered superficial, but with areas corresponding to contact/pressure zones with the mask which looked slightly deeper. This can be seen in the photographs. The patient was advised to not to wear a mask, and she was treated conservatively without any significant sequelae. No studies were found describing similar cases.
**Conclusions**: Face masks can cause facial burns to deepen due to both pressure and friction over contact points. We suggest that at the point of referral, patients should be exempted from wearing facial coverings to decrease the chance of potential scarring from deeper burns. The literature review provided no significant reports on facial masks and burns; however, there are some reports regarding other surgical wounds around the face.

*P.091*

**Female Victims of Intimate Partner Violence (IPV) in the Vall d’Hebron Burn Unit**

**Sara Guila Fidel-Kinori, Eva Fernandez-Mondragon, Maria Sonsoles Cepeda-Diez, David Mateu-Sellares, Carlos Cazalla-Soria, Joan Pere Barret-Nerin and Jordi Serracanta-Domenech**
  Hospital Universitario Vall d’Hebron

**Objectives**:
  - To describe the sociodemographic and clinical profiles of female victims of IPV admitted to the Specialized Burn Unit during 2020–2021;
  - To identify specific needs for appropriate actions towards these patients by the health organization.
**Methods**: This was a retrospective and descriptive study, performed using the Burn Unit database, on the admissions of women assaulted by their partners or ex-partners in the last two years. Sociodemographic, clinical characteristics, and evolution data were collected.
**Results**: Four women were admitted to the Burn Unit during the years 2020 and 2021, because they had been assaulted by their ex-partners: three in 2020, and one in 2021. In 2020, two were adults and the third was a minor. In 2021, two adult women were burned, but only one was able to be admitted, before dying. The summaries of data collected from these women include sociodemographic and clinical features, as well as the reason for admission, age, place of birth, years of residence, marital status, employment status, type of aggression, aggressor type of injury, TBS, days of admission, number of chirurgical procedure, and status at discharge.
**Conclusions**: Women admitted to burn units due to being victims of IPV represent a small percentage in the total number of admissions, but they present specific characteristics, due to the type of incident and its sequelae, which increases complexities in the process of recovery and reparation. Spain presents a large epidemiology of this kind of victims, but the uses of fire, scalds, and acid are the most common for ms of aggression. In contrast, injuries caused by knives and firearms are the usual for ms of aggression by partners or ex-partners. Additionally, the sons and daughters of these women can be victims of these people.
Specific measures of social and psychological support, such as linking to specific care resources, are key to ensuring continuity of care and support for integral recovery.
Specific identification of these women at the point of hospitalization should be carried out by the health organization, to ensure patients’ protection and to improve specialized care

*P.092*

**Effectiveness of Static Progressive Splinting in Minimizing Post-Burn Elbow Contracture**

**Mohamed Bayoumi**
  Lecturer at Faculty of Physical Therapy, Cairo University, Egypt

**Introduction**: Contractures are common after burn injuries, representing a major cause of psychological and functional morbidity.
**Objectives**: This study was conducted to investigate the effect of static progressive splints on patients suffering from post-burn elbow contractures.
**Methods**: for ty patients (males and females) suffered from post-burn elbow contractures, with ages ranging from 20 to 50 years old, and were selected randomly and divided into two equal groups.
Group (A) suffered from post-burn elbow contractures and were managed for 8 weeks with static progressive splints in addition to traditional physical therapy interventions (stretching exercise, ROM (range of motion) exercise and deep heating modalities), whereas patients in group (B) only received traditional physical therapy interventions for 8 weeks. All patients in both groups were assessed for ROM and the level of satisfaction at the start, after 4 weeks and after 8 weeks of the study.
**Results**: after 8 weeks of the study, when comparing the two groups (group A and group B), the mean values of ROM ± SD were 96.3 ± 9.5 and 114.85 ± 7.77, respectively, which indicated significant differences (*p* < 0.05) in favor of the static progressive splint group. Regarding the satisfaction level (SL), the mean values ± SD were 2.25 ± 0.639 and 3.55 ± 1.15, respectively, which indicated significant differences (*p* < 0.05) in favor of the study (static progressive splint) group.
**Discussion**: The results of the study showed clinical and statistical improvements in the ROM of elbow joints in patients who managed with static progressive splints. This improvement may attribute to the viscoelastic properties of the scar tissues which may respond to static progressive splints.
**Conclusions**: It was concluded that static progressive splints are an effective modality in the management of post-burn contractures with minimal complications and are less time-consuming; they also represent a valuable contribution to burn rehabilitation programs.

*P.093*

**Respiratory Response during Early In-Bed Cycling for Mechanically Ventilated Burn Patients**

**Elin Sundin ^1,2^, Filip Fredén ^1,3^ and Fredrik Huss ^1,4^**
  ^1^  Burn Center, Department of Plastic and Maxillofacial Surgery, Uppsala University Hospital
  ^2^  Rehabilitation in Cardiopulmonary Medicine, Orthopaedics and Intensive Care, Department for Rehabilitation and Pain Center, Uppsala University Hospital
  ^3^  Department of Surgical Sciences, Anaesthesiology and Intensive Care Medicine, Uppsala University
  ^4^  Department of Surgical Sciences, Plastic Surgery, Uppsala University

**Objectives**: Muscle weakness is a common problem among severely burned intensive care patients. Rehabilitation is recommended to start immediately to minimize the risk of muscle loss; one of the rehabilitation methods that can be considered during the early intensive care phase is motorised in-bed cycling exercise. As part of a larger study, the aim was to assess the feasibility of in-bed cycling, regarding respiratory responses during exercise, in an early phase of intensive care with invasive mechanical ventilation at the Burn Center at Uppsala University Hospital, Sweden.
**Methods**: Ten Burn-ICU patients, >18 years old, with continuous invasive mechanical ventilation, were monitored subsequently before, during, and after 20 min of exercise with a motorised in-bed cycling device. The variables monitored were respiratory rate, saturation of peripheral oxygen (SpO_2_), end-tidal carbon dioxide (ETCO_2_), arterial saturation of peripheral oxygen (SaO_2_), arterial partial pressure of oxygen (PaO_2_), and arterial partial pressure of carbon dioxide (PaCO_2_).
Safety limits, where the intervention would be discontinued if exceeded, were set for vital parameters before intervention.
**Results**: Nine out of ten patients managed to maintain the in-bed cycling exercise for 20 min, as recommended for other ICU patients. In the other patient, a temporary drop in systolic blood pressure led to premature cessation of the intervention. There were no clinically relevant changes in any of the analysed respiratory-related variables, between the subsequent pre-, intra-, and post-intervention phases.
**Conclusions**: In-bed cycling exercise in an early phase of Burn-ICU treatment during invasive mechanical ventilation seems feasible regarding the respiratory response. Tendencies for the respiratory rate to increase were seen; however, this not clinically significant, nor exceeded the pre-set safety limits. No other impact of clinical relevance was seen. However, larger studies need to be conducted to enable generalizable results.

*P.094*

**Swallow Rehabilitation of a 77-Year-Old Female, with Self-Inflicted 25% Burns and Psychiatric Premorbidity: A Case Report**

**Sonia Tsipi ^1^, Argyro Pipinia ^2^, Pavlos Pavlidis ^3^, Loukas Dagdilelis ^4^, Theodora Tsoliaridou ^2^, Anastasios Emmanouilidis ^2^, Sophia Papadopoulou ^2^**
  ^1^  Yakinthos Rehabilitation Center
  ^2^  Plastic Surgery & Burns Unit, “G. Papanikolaou” Hospital
  ^3^  ENT Clinic, Univerity Hospital Mainz
  ^4^  Department of Radiology, ‘‘G. Papanikolaou’’ Hospital

**Objectives**: Swallowing disorders or dysphagia following severe burns can lead to dehydration, malnutrition, aspiration pneumonia, prolonged hospitalization, and associated deterioration in the quality of life. Concomitant psychological factors and burn injuries add a layer of complexity to the rehabilitation program. This case report highlights and emphasizes the role of the speech therapist in the multidisciplinary team approach.
**Methods**: A 77-year-old female, with a history of two strokes, and psychiatric problems including anxiety and depression over the last 30 years, sustained 25% full-thickness head and neck and thorax burns with inhalation injury due to a suicide attempt by pouring and igniting pure spirit on herself. She was admitted on the day of injury and had a tracheotomy on the fifth post-burn day. Initial positive Evan’s Blue Dye Test on the 40th post-admission day showed penetration. A fiberoptic endoscopic evaluation of swallowing (FEES) revealed severe oropharyngeal phase dysphagia characterized by delayed swallow initiation, pharyngeal pooling with residuals, and penetration with silent aspiration of puree and liquid. Based on these results, a percutaneous endoscopic gastrostomy tube was placed, and she was referred to speech therapy for the rehabilitation of dysphagia.
**Results**: Repeat FEES after 26 sessions of therapy (3 times weekly), showed improvement in swallow function. She was able to clear residuals with multiple swallows. Coordination and swallow initiation speed increased enough to initiate oral feeding with the speech therapist. The patient’s dysphagia improved after 26 sessions and continued to improve over the following month. after 39 sessions, she started oral feeding and drinking. after that, a video fluoroscopic swallow study (VFSS) was performed, her gastrostomy tube was removed, and she could be discharged. Although an SLT is not a standard member of our Burns Unit, their contribution in the rehabilitation of this long-hospitalized patient was immense.
**Conclusions**: SLTs are involved in the assessment and treatment of dysphagia for patients with burn injuries. This case report emphasizes that burn management takes a team effort, including the early diagnosis and management of swallowing impairments. SLT have become valuable members in the burn care team, in order to facilitate burn patient rehabilitation through multidisciplinary collaboration.

*P.095*

**Evaluation of ICT Rehabilitation Tools during COVID-19**

**Laura Grossi Garriga, Eva Santacreu Santacreu, Silvia López Lebrato, Ariadna Marcé Rotger, Eugenia Díaz Recarey and Maria Lluïsa Torrent Bertran**
  Hospital Universitari Vall d’Hebron

At the last Burn Congress, we presented an ICT tool for telematic rehabilitation, which could not be used at the beginning of the pandemic due to hospital APP changes.
When our strict lockdown was ordered, our patients were unable to come at the hospital for the rehabilitation treatment; therefore, we asked for their email addresses in order to send them the recorded videos of different exercises and activities. We created an email address to enable rapid communication and to help them to solve all questions.
Once the lockdown eased, we asked those patients about their feelings and valuation of this telematic treatment. A Likert scale was developed to evaluate the ease, applicability, and effectiveness of this emergency communication system we created.
Unanimous conclusions were founded. Some patients with no computer knowledge had difficulties displaying the videos and contacting us. All showed gratitude for being able to contact us and ask their questions, and then for them answered. Most of them strongly showed their preference for on-site rehabilitation.
As a conclusion, these reflections must be considered to help us to develop new procedures to enable telematic rehabilitation—surely an essential tool in the near future—to be as inclusive and effective as possible.

*P.096*

**Scientific Evidence of the Rehabilitation Treatment in Burned Patients**

**Ariadna Marcé Rotger ^1^, Lluïsa Torrent Bertran ^1^, Eugenia Díaz Recarey ^2^, Laura Grossi Garriga ^1^, Sílvia López Lebrato ^1^ and Maria Nicolás Sola ^1^**
  ^1^  Hospital Universitari Vall d’Hebron
  ^2^  Complejo Hospitalario Universitario de A Coruña

**Introduction**: Rehabilitation treatment in burned patients is essential for achieving effective functional recovery. This enables the patient to return to their previous lifestyle as far as possible and minimizes sequelae. The efficacy of this treatment is widely accepted; however, we can find different treatment protocols.
**Objectives**: We aimed to determine the scientific evidence of therapeutic tools that constitute the therapeutic protocols, and to adapt our protocol to these results.
**Methods**: Bibliographic research of therapeutic tools of rehabilitation treatments in burned patients between 2009 and 2021 in different database platforms (PubMed, PedRo, Google Scholar, and Cochrane) was performed.
**Results**: We found that multidisciplinary teamwork, respiratory physiotherapy, kinesitherapy, occupational therapy, and global muscular training are the therapeutic tools exhibiting the strongest scientific evidence (medium level of evidence).
**Conclusions**: Despite the lack of robust evidence for the rest of the therapeutic tools, it is recommended to apply the entire therapeutic arsenal because there no other therapeutic weapons that have shown efficacy in burned patients. Based on these results, our protocol prioritizes early mobilization and exercise in the context of a multidisciplinary team.

*P.097*

**Acute Management of Burned Patients from a Rehabilitation Medicine Perspective**

**Ariadna Marcé Rotger ^1^, Eugenia Díaz Recarey ^2^, Laura Grossi Garriga ^1^, Sílvia López Lebrato ^1^, Maria Nicolás Sola ^1^ and Lluïsa Torrent Bertran ^1^**
  ^1^  Hospital Universitari Vall d’Hebron
  ^2^  Complejo Hospitalario Universitario de A Coruña

**Objective**: We aimed to describe the approach to the rehabilitation doctors’ management of burned patients in the acute phase.
**Methods**: We describe the evaluation methodology, treatment indication, and therapeutic plan for acutely burned patients followed by Physical Medicine and Rehabilitation Specialists of the Vall d’Hebron University Hospital’s Burn Unit (Barcelona).
**Results**: When treating a burned patient, the rehabilitation doctor performs a detailed anamnesis and a directed exploration, with the aim of defining those patients who are suitable for receiving rehabilitation treatment and establishing a directed treatment plan.
In anamnesis, sociodemographic data (age, comorbidities, previous functionality, and patient’s social situation) are collected, and the burn is defined (data, etiology, surface, depth, and possible associated factors).
In physical examinations, the following variables are generally assessed: higher functions (level of consciousness and ability to collaborate), respiratory status (need for tracheal intubation, smoke inhalation, etc.), especially sensitive areas (natural orifices, neck, and hands), and joint balance (of burned and intact limbs).
Specifically, in the event of smoke inhalation or previous lung pathology, the patient is auscultated, and in case of electrical burn or polytrauma, a standardized neurological examination is carried out.
Rehabilitation treatment subsidiary patients are defined as those who present one of the following features: respiratory disorders, burns affecting joints or areas of special sensitivity, trunk burns in growing children, associated injuries (myopathy, neuropathy, amputation), and patients at risk ages (children and elders).
The rehabilitation treatment of burned patients in the acute phase is based on postural control, anti-edema measures, mobilizations, motor enhancement, splinting, re-education in transfers and daily living activities, standing and walking. This treatment is performed two or three times a day in 30 min sessions, according to the patient’s tolerance.
Functional stability, scar maturity, the definition of the sequelae, and the achievement of the established objectives determine the end of the rehabilitation treatment.
**Conclusions**: It is important to enhance the rehabilitation doctor figures in burn units. An early, multidisciplinary, holistic, and individualized rehabilitation treatment minimizes sequelae in burned patients, helping to achieve their maximum possible functionality and social reintegration.

*P.098*

**Use of Xenografting Compared with Synthetic Cellulose Dermal Substitute (Epicite) in Patients with Second-Degree Burns**

**Enrique Chau Ramos**
  Hospital Lima

**Objective**: This study was carried out with the comparison of healing between dermal substitutes: regenerated cellulose (EPICITE) and xenograft, in patients with second-degree burns.
**Methods**: The report of 60 cases evaluated in a hospital in Lima, Peru, is presented; between January 2018 and December 2021, in patients between 1 and 60 years of age, without comorbidities, where the evaluation of healing in patients with second-degree burns with hot liquid is recorded. This was a comparative, interventional, analytical, prospective, and longitudinal study. Where two dermal substitutes were used at the same time in all patients, this study gained the authorization of each patient through informed consent.
**Results**: after 90 days, an evaluation was carried out, showing better healing with a synthetic cellulose dermal substitute (EPICITE) compared with xenograft; having evaluated the healing results with the Vancouver scale (vascularity, pigmentation, flexibility, and height); being the results with synthetic dermal substitute of cellulose with less redness and greater elasticity, which are the most predominant indicators.
**Conclusions**: In this study, it was possible to show that the synthetic dermal cellulose substitute (EPICITE) is an important alternative that favors the quality of healing in burned areas. It was shown to be more efficient than the xenograft, when evaluated and compared in its four parameters with the Vancouver International Healing Scale. This result is important, because it is an efficient alternative in the treatment of second-degree burns, favoring a better healing process.

*P.099*

**The Use of Platelet-Rich Auto-Plasma in the Treatment of Scars**

**Alina Grigorova, Oksana Vladimirovna Vladimirova, Sergey Viktorovich Minaev, Vladimir Ivanovich Vladimirov, Peter Mikhailovich Lavreshin, Vakhtang Vladimirovich Gobedzhishvili and Sofia Sergeevna Korablina**
  Stavropol State Medical University

**Objective**: To improve the results of autodermoplasty, individuals with chronic non-healing wounds, and reduce the risk of pathological car for mation.
**Materials and Methods**: Platelet-rich autoplasma is a product obtained from the two-step centrifugation of patient blood in a specialized vial containing a purified heparin-based separator gel. The composition enables the sustainable separation and concentration of platelet growth factors, cytokines, and bioactive molecules. The intraoperative infusion of platelet-rich plasma combined with the surgical debridement and closure of the injury with an autodermal graft followed by repeated plasma infusion in the early post-operative period was used in the purulent surgery department. A total of 36 patients aged 21 to 59 years old were followed up during a 2-year period with traumatic injuries, third-degree burns of 5% to 20% of the body area, traumatic wounds to between 2% and 5% of the body area with no cover tissues within the wound. The control group included 15 patients who had been treated using a standard approach.
**Results**: Two days after the surgery, 17 (81.0%) patients of the study group and 11 patients (64.7%) of the control group exhibited partial graft retention. The initiation of marginal epithelialization and secure graft fixation was seen on Days 4 to 5 in the study group and Days 7 to 8 in the control group. after 9 days, the graft survival showed no signs of significant inflammation, with the active epithelialization being 90% to 96%. There were no significant complications in the early post-operational period in either group.
Pathological excessive (hypertrophic) scars were seen in 10 patients (66.7%) of the control group and 6 (28.8%) patients of the study group. In 10 (66.7%) patients of the control group, complaints about itching, dragging sensations in healed wound area, severe cosmetic defects due to skin plasty discrepancies in the density and height of circumferential intact skin were registered during the prolonged post-operation period. In the autostimulation group, such complaints were noted in merely 6 (28.8%) patients. Mild hypertrophy, with no significant complaints about dragging, pressure, and limited mobility in the area of the developing scars was seen in 5 (33.3%) patients of the control group and in 5 (23.8%) patients of the autostimulation group.
**Conclusions**: The use of platelet-rich plasma has demonstrated good results when used in combination therapy to treat burn and traumatic wounds, provided more rapid and uneventful graft healing, and improved rehabilitation in patients, facilitating shorter periods of inpatient treatment and reduced expenditures.

*P.100*

**Is Treatment with Platelet-Rich Plasma (PRP) after Burn Injury a Contribution in Scar Management? A Pilot Study**

**Ester Peters, Marscha Heijblom and Helma Hofland**
  Maasstadziekenhuis

**Objectives**: Scar management after a burn injury, such as through laser therapy, micro-needling, and endermology, is widely known and used to benefit patients with scars. A new method uses platelet-rich plasma (PRP), also known as autologous conditioned plasma. after drawing blood, the centrifuged plasma is injected into the scar with a special device. In the plasma, a higher concentration of growth factors is found than typically in blood. The PRP injections support the restoration of injured tissue and inhibit painful inflammatory processes with possible effects of regeneration. Although this treatment is used, with positive results, in several areas such as surgical scars and acne, it has not been researched in patients with scars after burn injury. To identify whether this PRP could be a useful new treatment, a pilot study was conducted.
**Method**: Five patients with different types of mature scars (see table) were asked to participate. All patients received PRP treatment in one part of the scar; another part was not treated with PRP. after a pre-measurement, the scar quality was assessed for three consecutive months (November 2021, December 2021, and January 2022). The POSAS 2.0 patient and observer scale, rated by patients and researchers, and a Cutometer, employed to establish elasticity of the skin, were used. Furthermore, the patients were asked to report whether they experienced sensory and physical changes between the treated and the untreated scar.
**Results**: All patients received treatment as planned, consisting of one dose of injected plasma. The patients did not experience any pain or discomfort during the treatment. No side effects were seen. The Cutometer results did not provide clear evidence on the improvements in elasticity. However, all patients reported a functional and sensible improvement. Furthermore, if patients compared the treated scar with the untreated scar, they also reported improvements. In addition, this was shown in the outcomes of the POSAS by patients and researchers.
**Conclusions**: PRP is a promising adjunct in scar management practice. Further research with long-term follow-ups is warranted. However, there are some disadvantages; this method can only be used if blood can be obtained through a vena puncture, and unfortunately, this can sometimes present difficulties.

*P.101*

**Preliminary Experience with HDR Brachytherapy for the Treatment of Keloid Scars**

**Akshat Wahal ^1^, Laura Cappuyns ^1,2^, Isabel Syndikus ^3^, Christopher Lee ^3^, Dilnath Gurusinghe ^1^ and Kayvan Shokrollahi ^1^**
  ^1^  St. Helens and Knowsley Teaching Hospitals NHS Trust
  ^2^  Manchester Metropolitan Univeristy
  ^3^  Clatterbridge Cancer Centre NHS Foundation Trust

**Introduction**: Keloid scars are a challenging condition to treat, with a high recurrence rate with surgery. Alternatives often require numerous, and prolonged, clinical interventions, such as intralesional injections. External beam radiotherapy is an option with a proven track record of efficacy, but comes at the price of a significant radiation dose which limits its usage. Brachytherapy allows for the surgical excision of keloids and delivery of radiation precisely, in a manner isolated to the scar. It has a track record of success, but has been not widely adopted due to logistical constraints and availability.
**Objectives**: We demonstrate our preliminary experience of high-dose rate (HDR) brachytherapy and outcomes from two cases.
**Methodology**: Two patients were treated with the excision of keloids followed by post-operative HDR brachytherapy undertaken on the same day with the aid of CT imaging at a regional cancer centre. Surgical excision entailed excision with 1 mm margins and subcuticular closure over a 6 French flexible polyethylene catheter. HDR Iridium-192 brachytherapy was administered. In total, 12 Gy in divided doses of 4 Gy in two fractions were administered with a depth of 10mm from the axis of tube.
**Results:** We present two male patients with a long history of recalcitrant keloids affecting the face/neck and the chest, who had previously been unsuccessfully treated with various modalities over many years. Both patients underwent surgery and HDR brachytherapy as a day case. With a follow-up of longer than 12 months, we achieved good aesthetic and symptomatic results without recurrence and high satisfaction from the patients.
**Conclusions**: We describe our preliminary experience with a combination of surgery and HDR brachytherapy for keloid scarring and demonstrate our technique. Our first few cases have demonstrated the potential to expand on this modality, and future studies are likely to be able to demonstrate our ability to discharge patients from clinics and avoid numerous repeat interventions and appointments in favour of a single definitive technique.

*P.102*

**Systematic Review on Working Mechanisms of Signaling Pathways in Fibrosis during Shockwave Therapy**

**Lot Demuynck**
  Uantwerpen

**Objective/background**: To review the underlying effect of mechanical for ces (especially shockwave therapy or SWT) on scar for mation/fibrosis.
Skin injury is a common occurrence. Every year, 40–70% of the over 80 million scars induced in the developed world, either after surgical procedures or caused by traumatic and burn wounds, develop into problematic hypertrophic scars. Mechanical for ces (such as SWT) can have a beneficial role in scar for mation by activating or modulating signaling pathways on cellular and molecular levels. Today, it is still questioned how precisely mechanical for ces promote wound healing and reduce scar for mation. Previous studies suggest that mechanical for ces initiate a process of mechanotransduction. Thus, controlled mechanical for ces can prevent or alter pathological scar for mation. Nevertheless, it is unclear exactly which pathways are involved in the application of ESWT.
Therefore, we want to review the underlying effect of SWT on fibrosis. By reviewing this, we can further understanding of scar treatment and develop a more standardized scar treatment (under the for m of ESWT modalities).
**Methods**: An SR of all studies of the underlying effect of mechanical for ces in fibrosis was conducted. We performed the search in PubMed and Web of Science, finding 1633 articles associated with our research question.
**Results**: In first screening phase, 21 studies were included. Preliminary evidence shows that ESWT activates macrophage activity, fibroblast activity, the amount of collagen and orientation, TLR-3, TGF ß1/Smad, mTOR-FAK, YAP/TAZ, and apoptosis. Depending on the energy levels of ESWT, other proteins and pathways can be activated. Moreover, different frequency levels can influence other proteins and pathways. Final results about the exact energy levels and frequency will be presented at the congress.
**Conclusions**: We can conclude that ESWT has beneficial effects on the prevention of hypertrophic scar for mation through influences on the aforementioned proteins and pathways. Most of the studies are experimental; therefore, we can introduce the treatment of ESWT in scar for mation. Based on these data, which highlight the underlying mechanisms, we can draw preliminary conclusions about the treatment modalities of ESWT in scar for mation. Suggestions can be made about the energy levels and frequencies that are necessary to prevent or alter hypertrophic scar for mation. Final conclusions about the systematic review will be presented at the congress.

*P.103*

**Pros and Cons of Early and Late Skin Grafting in Children with Burns—Evaluation of Common Concepts**

**Islam Abdelrahman, Ingrid Steinvall, Folke Sjöberg, Mohamed Ellaban, Johann Zdolsek and Moustafa Elmasry**
  Linköping University

**Background**: There is no consensus regarding the timing of surgery in children with a smaller burn size, specifically in deep dermal burns. Delayed surgery has risks in terms of infection and delayed wound healing. Early surgery also risks the removal of potentially viable tissue. We wanted to highlight the pros and cons of early and late skin grafting in children with burns. Therefore, our aim was to investigate the effect of the timing of surgical interventions (i.e., early versus late) on the size of the area operated on, time for wound healing, and the requirement of extra operations in children with burns.
**Methods**: A retrospective analysis was performed for all children (<18 years) with burn size <20% body surface area (BSA%) during 2009–2020 who were operated on with a split-thickness skin graft. The patients were grouped by the timing of the first skin graft operation: early, operated on within 14 days of injury; delayed, operated on more than two weeks after injury.
**Results**: A total of 84 patients were included in the study: 43 who had an early operation, and 41 who had a delayed operation. There were no differences between the groups regarding burn size, or whether the burns were superficial or deep. The duration of healing time was seven days longer in the group with delayed operation (*p* = 0.001). The area operated on was somewhat larger (although not significantly so) in the group who underwent early operation. Nine children had two skin graft operations: eight in the early group and one in the delayed group (*p* = 0.03).
**Conclusions**: The patients who were operated on early had the advantage of a shorter healing time, but there was a higher rate of complementary operations and a tendency towards a larger burn excision.

*P.104*

**MATRIDERM^®^ a Collagen–Elastin Matrix—15 Years of Experience and 452 Grafts Later: What Have We Learned?**

**Eric Dantzer**
  Instruction Military Hospital Sainte Anne

**Introduction**: A dermal equivalent composed of a collagen–elastin matrix is available for the treatment of burn wounds with the possibility of an epidermis graft during the same surgical stage. The purpose of this study was to evaluate the initial and long-term final cosmetic and functional outcomes over soft tissue defects.
**Methods**: after skin trauma, surgical excisions were performed to obtain a viable plane without necrotic or infected tissue. after hydration, the matrix was applied and split-skin was grafted on the top of the matrix; then, the two layers were stapled together. Local dressings were changed every 4 days.
**Results**: Since 2007, 408 patients (241 males and 167 females from 15 to 84 years old) were treated, with a total of 452 grafts performed for 173 acute burns, 90 post-burn reconstructive surgeries, 63 traumatic wounds and necrotizing fasciitis, and 27 carcinoma. The surfaces grafted were from 2 to 2596 cm^2^. The mean healing time was 15 days. The complications observed were 9 hematoma, 2 partial loose dermis, 15 local epidermal dislocations, and 12 local infections (treated with local antiseptic). There were 21 new partial epidermal grafts needed; 18 secondary retractions were observed after post-burn reconstructive surgery. The 10-month follow-up showed rapid and good functional results.
**Discussion**: Yannas and Burke hypothesized that the dermal part of the skin could be restore by a dermal substitute; their bilayered matrix had to be recovered in a second surgical stage 3 weeks later by an epidermal graft. Only a one-stage surgical procedure was possible with the collagen–elastin matrix. The final healing time was reduce. The one-step procedure prevented the infection risk that could occur in the delay between the two surgical procedures. for the patients, this technique reduced the number of surgical processes during the instable phase, and for all patients it reduced the hospital stay. The retraction of the graft was insignificant. after one year, the clinical evaluation exhibited very good integration of the graft. Histological results of biopsies taken between 10 and 12 months after grafting showed the regular organization of the dermal layer. By restoring the shearing planes, the collagen–elastin matrix thus avoids deep-rotted adhesion and improves the tegumentary suppleness and the final function.
**Conclusions**: after this series and long-term follow-up, we consider the Matriderm^®^ collagen–elastin matrix to be a promising dermal substitute for good functional and cosmetic long-term results with rapid healing and a shorter hospital stay.

*P.105*

**The Production and Application of CEAs across Borders**

**Saba Tantula, Melina Gigante-Bounid, Andy Lindford and Jyrki Vuola**
  Helsinki Burn Centre

**Introduction**: In Europe, there are only a few institutions that can produce cultured epithelial autografts (CEAs). We herein describe the process in which CEAs were produced in one European country and then transported to another country for wound coverage in patients with major burns in a well-planned time schedule. The purpose of this paper is to describe this process and the preparation of the patient for the procedure.
**Methods**: During 2016–2021, three severely burned patients received treatment with CEAs in the Helsinki Burn Centre. Skin biopsies were sent to Switzerland where they were cultured in the Centre de Production Cellulairé, CHUV, Lausanne. after 3–4 weeks, the cells attached to polyamide sheets were delivered by personal courier in a time window of 8 h straight to the operation theatre and successfully administered to the patients. Prior to this, the patients had undergone several debridements and allograft treatments, and the wounds were confirmed as not having any infection or necrosis.
**Results**: Three patients with a TBSA burn% ranging from 78% to 92% each received 3000 cm^2^ of CEAs. Unfortunately, the first two patients eventually died, but the third patient, with a TBSA of 78%, survived. The CEAs were applied on top of widely meshed autografts to enhance the epithelialization. The exact benefit of the accelerated healing was difficult to measure. Histological staining confirmed that the cells were alive on arrival. The total costs were less than EUR 10/cm^2^.
**Conclusions**: Cultured epidermal autografts have not (yet) achieved large popularity. The high price and strict regulations are clear hindrances to their production and delivery. With good co-operation, autologous cells can be cultured in one non-EU country and delivered to another EU country located far away, by careful planning and with a strict time schedule, with acceptable costs.

*P.106*

**Free Microvascular Tissue Transfers in High-Voltage Electric Burns: A Single Institute Experience**

**Burak Ozkan ^1^, Cagri A. Uysal ^1^ and Mehmet Haberal ^2^**
  ^1^  Department of Plastic, Reconstructive and Aesthetic Surgery, Faculty of Medicine, Baskent University
  ^2^  Department of General Surgery and Burn Center, Faculty of Medicine, Baskent University

**Introduction**: Free microvascular tissue transfer can provide excessive tissue in one stage in extensive defects where loco regional flaps options cannot be performed. Thus, free flaps represent an important reconstructive option in burn reconstruction whenever neurovascular and skeletal structures are exposed. This sophisticated technique needs surgical expertise and understatements of burn physiology rather than non-burn trauma cases. In this study, we share our experience in burn reconstruction with free flaps.
**Material-Methods**: Between 2017 and 2021, we performed 26 free flaps in 20 burn patients. There were 15 flaps performed in 12 patients in the early phase (first 21 days of the injury); 60% of the flaps were skin flaps (anterior lateral thigh, radial for earm, scip, and parascapular), 20% were musculucutanoeus (latissimus dorsi and vastus lateralis), 10% were fascia flaps (temporal fascia, serratus anterior), and 10% were pure muscle flaps (gracilis, latissimus dorsi).
**Results**: Two free flaps in the early phase reconstruction and one free flap in the late-phase were lost. The etiology of the flap loss was venous congestion in three cases and arterial occlusion due to hematoma for mation in one case. Debridement of the necrotic flaps was delayed until demarcation for mation settled and sub-flap granulation for mation was provoked. Skin grafts were performed after debridement of these flaps. Other flaps completely survived, and no recurrence in contractures or defects was encountered.
**Conclusions**: Although free flaps have changed the reconstructive ladder to reconstructive elevators, performing these flaps has unique challenges in burn reconstruction, such as being prone to thrombosis in electric burns, hemodynamic instabilities, and difficulties in patient positioning due to sedation. Meticulous care should be taken, and patients’ general conditions should be well evaluated prior to free flap surgery.

*P.107*

**Escharotomy for the Face: Facial Aesthetic Subunit Principle-Based Approach**

**Burak Ozkan ^1^, Cagri A. Uysal ^1^, Emin Turk ^2^ and Mehmet Haberal ^2^**
  ^1^  Baskent University Faculty of Medicine, Department of Plastic, Reconstructive and Aesthetic Surgery
  ^2^  Baskent University Faculty of Medicine, Department of General Surgery and Burn Center

**Introduction**: Escharotomy is the relaxation of the eschar through longitudinal or horizontal incisions in order to protect the region’s deep perfusion. The pressure that it will create in the peripheral areas such as hard eschar limb, trunk, and neck causes the circulatory disorder in the limb and the risk of limb loss, inadequate thoracic expansion in the thorax and vital perfusion and oxygenation problems in the neck. It is one of the most basic rules of burn surgery to perform the determined escharotomy incisions very quickly and without hesitation to prevent complications. On the other hand, the face is not an area in which eschar for mation commonly seen because of its robust vascular supply and protection reflex of the patients. Although descriptive drawings and guides for facial escharotomy have not been published yet, the relaxation of axial arteries in terms of compression due to eschar for mation may be needed. In this report, a case of facial subunit principles based escharotomy is presented.
**Case Report**: A 42-year-old man fell into hot sand after having a vertigo attack while working close to an iron-processing zinc-leaded high-blast furnace in an iron and steel factory. Sand, which was used for the isolation of the ground, was placed 10 meters down the furnace and the patient stayed approximately half minute in a prone position on the hot sand. The patient transferred to our burn unit for corresponding 35% of the total body surface burns on the face, total scalp, neck, anterior thorax and bilateral upper extremities, although he had worn protective equipment. There was no capillary fill in the facial skin. Doppler ultrasound examination showed bilateral weak facial artery, temporal superficial artery, supraorbital and trochlear artery flow. A decision was made to perform escharotomy to relieve arterial traces at the 10th hour of the injury. Bilateral nasolabial, infraorbital rim, superior glabellar, and temporal incisions were performed from eschar to the subcutaneous fat layer in accordance with aesthetic subunits.
**Conclusions**: Facial escharotomy based on aesthetic subunit principles may affect the outcome by releasing axial arteries of the face. This maneuver may increase the amount of tissue to survive by releasing diminished circulation of the skin. Escharotomy of the face can help rapid edema relief and be a guide for skin grafting without incision-related excessive scarring.

*P.108*

**The Management of Circumferential Electro-Thermal Ring Burns: A Case Report and a Proposed Treatment Algorithm**

**Burak Ozkan ^1^, Cagri A. Uysal ^1^, Emin Turk ^2^ and Mehmet Haberal ^2^**
  ^1^  Baskent University Faculty of Medicine, Department of Plastic, Reconstructive and Aesthetic Surgery
  ^2^  Baskent University Faculty of Medicine, Department of General Surgery and Burn Center

**Introduction**: Ring-associated burns are infrequent, comprising only a small fraction of burn consults. Due to the circular nature of rings, these burns are often circumferential, with an increased risk for compartment syndrome and neurovascular injury. The severity of ring burn is related to the type of material and electrical current which is related to the voltage of the electrical source. Low-voltage injuries are generally due to contact with batteries or household devices, while high-voltage electric injuries occur with occupation-related accidents or natural disasters. Low-voltage ring burn can be managed conservatively with close follow-up. However, high-voltage ring burn may lead to dramatic consequences such as finger amputation. To date, there have been few cases reported in the literature of ring burn. Most of the cases were superficial burns and managed with secondary healing or skin grafting. However, the management of severe ring burn with deteriorated finger circulation has not been reported in the literature. In this case, report, we present a circumferential electro-thermal ring burn case which resulted in the total loss of a finger. Additionally, we propose an algorithmic approach to ring burn injuries.
**Case Report**: A 40-year-old male working as a welder was transferred to our burn care center. While working, he accidentally touched a wire with his left hand. At presentation, the patient had a circumferential 1 cm wide, circumferential, full-thickness burn in his proximal phalanx of the fourth finger and an eschar for mation at the second metacarpal head level on the palmar side. The finger was gradually congested and the circulation was reduced using relaxation incisions, medical leech application and external bleeding. Ray amputation was performed after total demarcation of the finger, and the stump was closed primarily.
**Conclusions**: In conclusion, while superficial ring burn can be successfully managed with conservative treatment with close follow-up, a full-thickness burn with vascular compromise should be intervened with relaxing incisions, escharotomies, locoregional flaps, and external bleeding. Urgent revascularization with flow through venous flaps and vein grafts should be considered if there is any suspicion that finger circulation has been lost.

*P.109*

**Two Cases of an Unusual Presentation of Electrical Burn Injuries**

**Burak Ozkan ^1^, Santiago J. Santelis ^2^, Abbas Albayati ^1^, Ayse Ebru Abali ^2^, Cagri A. Uysal ^1^ and Mehmet Haberal ^2^**
  ^1^  Baskent University Faculty of Medicine, Department of Plastic and Reconstructive Surgery
  ^2^  Baskent University Faculty of Medicine, Department of General Surgery and Burn Center

**Introduction**: Electric injuries have a wide variety of consequences ranging from disfigurements, extremity loss and death. The limbs are the most affected sites because of the high resistance of muscles and tendons to electricity. The most common pathway of entry to exit point is upper limb to lower limb, so the thigh to foot pathway is exceptional. In this case, report, we aimed to present the mechanism of how two construction workers suffered a high-voltage electric burn.
**Case Reports**: Two construction workers, with no relevant medical history, experienced a work-related accident. They were shocked by an electric current while carrying out work on a public road in the morning; the task involved guiding and unloading with the hands a steel plate lifted by a truck-mounted crane parked near overhead high voltage power lines. During the procedure, the crane accidentally contacted a 6300-volt overhead energized line and the patient provided a path to ground being electrocuted with the consequent electrical injuries. On psychical examination, both of them had third-degree electric entry- and exit-point burns limited to their lower extremity. Multiple surgical interventions were performed for the reconstruction of lower-extremity wounds.
**Conclusions**: Every year, an average of 15 electrocutions are caused by contact between cranes overhead power lines, and over 50% of the crane-related electrocutions occurred in the construction industry (20). These two cases remind us of the importance of safety procedures for construction workers; following these guidelines will reduce the chance and severity of these injuries. Electric burn patterns limited to the lower extremities are not usual and wearing unappropriated dressings can aggregate the injury.

*P.110*

**Identification of Independent Risk Factors for the Treatment of -Third-Degree Burns with the NovoSorb Biodegradable Temporizing Matrix (BTM) in a Multifactorial Logistic Regression Analysis**

**Felix H. Vollbach ^1,2^, Benjamin F. Thomas ^1,2^, Björn Bliesener ^1,2^ and Ulrich Kneser ^1,2^**
  ^1^  BG Trauma Center Ludwigshafen-Burn Center
  ^2^  Department of Plastic Surgery, University of Heidelberg

**Objectives**: NovoSorb biodegradable temporizing matrix (BTM) received European approval in November 2019. Since then, it has been successfully used in the treatment of multiple complex wounds including surgical, traumatic and infectious full-thickness defects, besides third-degree thermal wounds. Independent risk factors affecting the ingrowth of BTM and, subsequently, the take of the skin graft have not yet been analyzed. Here, we report on our 2 years’ experience in the treatment of 88 burn patients using multifactorial logistic regression analysis.
**Methods**: Since their introduction in our clinic in March 2020, all burn patients treated with BTM (*n* = 88) were included in the analysis. Only third-degree burns were treated with BTM. Demographic, traumatic, reconstructive, as well as surgical data were compiled, and multifactorial binary logistic regression models were computed to identify independent predictors for its ingrowth and skin graft take.
**Results**: out of 88 patients with third-degree burns (38 female, 50 male), five patients died (5.7%, two females and three males). The mean age and BMI were 55.7 years (± 20.1 years) and 27.3 kg/m^2^ (± 5.7), respectively. The mean percentage of total body surface area burned (% TBSA) was 18% (±19.4, min 0.2%, max 95%) and an average of 4.8% TBSA (±7.9, min 0.2%, max 49%) was treated with BTM. The mean period from trauma to BTM application and subsequent skin transplantation was 13 days (±14.4 days) and 30 days (±6.6 days). The mean BTM and skin graft take was 84.8% (±22.7%) and 86.3% (±23%). Upon multifactorial binary regression analysis, BTM take (*p* = 0.001), BTM infection (*p* = 0.001), prior cadaver skin homograft (*p* = 0.02), exposure of bone (*p* = 0.01), diabetes mellitus (DM) (*p* = 0.04) and peripheral artery disease (PAD) (*p* = 0.01) proved to be independent risk factors for impaired BTM and skin graft take.
**Conclusions**: Good BTM take is the most important factor influencing skin graft take. Therefore, early debridement and instant BTM application should be emphasized to avoid infection. In cases with impaired vasculature of the wound bed, systemic or topical antibacterial treatment should be applied.

*P.111*

**Delayed Surgical Necrectomy of Burn Wound after Initial Special Dressing Treatment**

**Albin Stritar, Luka Emeršič and Klemen Lovšin**
  University Medical Centre Ljubljana

**Objectives**: It sometimes proves difficult to define burn depth when treating mid-dermal burns, flash burns, scalds or chemical lesions. In these undefined cases, it is possible to initially introduce special burn dressings with their own mechanical and pharmacodynamic characteristics. Wound dynamic is monitored when changing the dressings. Should wound healing stop, surgical procedure is required.
**Methods**: during the first three days, burns of an unspecified degree are treated using wet dressings; afterwards, special dressings are applied. Wound dressing diminishes inflammation, infection and prevents the deepening of the wound. It is in this manner that vital dermis and wound healing potential are preserved. Apart from healing by first intention, the granulation phase is prolonged in some areas of the wound, preventing the process of epithelization, which results in delayed wound healing. In those cases, surgical therapy of slough as secondary necrectomy is indicated.
**Results**: Five patients that were initially treated with special dressings only were analyzed. Surgical treatment of some of the unhealed areas was performed. The population comprises three children (10–15% TBSA) and two elderly patients (15–20% TBSA).
**Conclusions**: The case in point is an individual selective yet active approach. Regarding mid-dermal burns, the demarcation of the burn wound is expected so that it would not gradually heal in its entirety were conservative therapy only to be applied. Indeed, delayed surgical necrectomy decreases the size of the surgical area and skin graft needed. This is especially important in certain populations, i.e., children and elderly patients. A thoughtful surgical management results in an optimal ratio between an operative and conservative approach of burn wound treatment.
Finally, multidisciplinary care is required, which includes microbiological diagnostics, targeted antibiotic therapy, glycemic regulation, nutritive support and physical therapy up until complete rehabilitation.

*P.112*

**Unintentional Perioperative Hypothermy in the Burned**

**Olga Kovalenko, Anton Kovalenko, Oleksandra Lynnyk, Natali Belinska and Andrew Grisha**
  Bogomolets National Medical University

**Objectives**: Fluid loss through the burn surface is more than 300 mL/m^2^/h (normal—15 mL/m^2^/h). Heat loss through the burn surface is up to 580 kcal/h. Hypothermia occurs in patients during surgery, even without burns; in patients with burns, it occurs more often due to loss of skin function and the excision of necrosis.
**Aim**: To study the frequency of perioperative hypothermia in patients with burns.
**Methods**: We observed 31 patients aged 18 to 70 years with burns TBSA 25–60%. Eleven patients underwent surgery under intravenous anesthesia, twenty-two combined endotracheal anesthesia. The duration of the operation, blood loss, the excised area, skin T(temperature), basal T (BT) and T of air in the operating room were calculated. BT was measured one hour before surgery, during surgery, and three times during the first day after surgery.
**Results**: The temperature in the operating room was 18–20 °C. The excision of more than 4–5% necrotic tissue led to additional heat loss. The heating of the operating table area was carried out by two infrared heaters, which maintained the air temperature around the patient at 25.6 °C (24.7; 27).
In nine patients, 5% (3.5; 5.5) of necrosis was excised; in seven patients, wound areas were closed with xenografts and in two patients they were closed with autografts. The anesthesia was intravenous. BT before surgery was 37.2 °C (36.8; 38.1). The surgery lasts 48 minutes. after 45 minutes, the BT dropped to 0.5 °C (0.4; 0.9).
In 22 patients, 9.5% (7.7; 12) of necrotic tissues with xenograft closure were excised once. Anesthesia was endotracheal. BT before surgery was 37.0 °C (36.4; 37.8); 45 minutes later, it fell to 0.90C (0.6; 1.2). There was a decrease in BT from baseline by 1.4 °C (1.2; 2.1) if the duration of surgery exceeded 60 minutes. Peripheral vasoconstriction was observed when BT fell below 36.5 °C. The fall in T during surgery is associated with the excision of necrotic tissue, the use of muscle relaxants, vasopressors, and intraoperative blood loss; it manifested as tremors or chills in 44% and 72.7% of procedures, respectively. In the postoperative period, six patients (group size, twenty-two) required additional hemostasis.
**Conclusions**: The drop in body temperature in the perioperative period was observed in all patients with a burn area greater than 25%, with the excision area more than 5% of necrotic tissue. Postoperative blood loss increases with hypothermia. Cold shivering increases oxygen.

*P.113*

**Bromelain-Based Enzymatic Debridement in the Treatment of Burns: A Standardized Post-Treatment Dressing Proposal**

**Tiziana Pagliarini, Carmela La Greca, Giuseppe Spaltro, Simone Moroni, Paolo Palombo, Rosalba De Maria, Alessia Zinni and Antonella Di Romano**
  Uoc A.S. Centro Ustioni E Chirurgia Plastica Ospedale S. Eugenio Roma

In recent years, the complex treatment of medium and deep burns has been supplemented by non-surgical techniques which, in some cases, replace the surgery, traditionally the gold standard.
One of these non-surgical techniques is Nexobrid, a bromelain-based enzyme debridement, introduced to the market in 2012. It is used for full-term, medium and severe burns.
Methods: The study is being conducted at “Sant’Eugenio” Hospital Burns Center in Rome. It began in June 2021 and is ongoing.
The first patient, with burn for an explosion, was subjected to cleaning and removal of the blisters and subsequently treated with Nexobrid at the level of burns of the hands bilaterally.
According to the manufacturer’s instructions, Nexobrid was applied and held in place for 4 h. It was then removed, and moist dressings were applied to the areas affected by the treatment. In this phase, lacking univocal indications on the specific type of dressing to be applied post-treatment, we compared, at the level of the two hands, the effects of two different types of wet dressing: Nexobrid versus Biatain. In this regard, although Biatain is a wet-to-dry dressing, in this circumstance, the bottom of the burns treated with Nexobrid is permanently moist to avoid the absorption of the wounds’ humors and maintain a humid environment, as prescribed.
The burned surfaces treated with Nexobrid were kept under observation to highlight the evolution.
Following the first evidence, illustrated below, we have turned the choice of post-treatment dressing with Nexobrid exclusively to the use of Noruxol, and we are continuing to collect the results related to this option, which looks very promising.
**Results**: The comparative choice was made between Noruxol, a commonly used product, usually available in hospitals, which requires frequent at least daily changes of dressings, and Biatain, an alginate sponge, with a lower average availability, but with the potential advantage of a less frequent replacement of dressings. Both products have known and proven treatment efficacy.
**Conclusions**: To optimize these results, in the light of this experience, at the Burn Center of the Sant’Eugenio Hospital in Rome we stably adopted the Noruxol dressing after treatment with Nexobrid, even when the surgical treatment was planned in the short term, as it keeps the environment adequately moist and enhances the proteolytic effect of bromelain.

*P.114*

**Severe Electrotrauma Treated by Local Fasciocutaneous Flaps**

**Jiri Stetinsky**
  Burn Centre, University Hospital Ostrava

**Objective**: The aim of this work was to demonstrate the possibility of a local transpositional fasciocutaeous flaps to cover deep defects caused by high-voltage electrotrauma of the for earm soft tissues with a loss of many tendons, muscles and even main nerves.
**Methods**: We chose the method of covering the residual for earm flexor and extensor tendons without peritoneum. As the patient was in a critical condition, the treatment method had to be as simple as possible. The radial artery was also thrombotzsed. The residual skin of the upper for earm was only available to cover wrist defects.
**Result**: Transposition flap from the for earm covered the dorsal wrist defect as well as volar wrist defect. The donor site was treated by skin graft.
**Conclusions**: In the case of exposed deep structures such as nerves, tendons and bones, there is a need to protect these ones and to allow further reconstruction of tendons and nerves. A method using local random fasciocutaneous flaps could be the best choice due to its simplicity and possibility for other reconstructive surgery.

*P.115*

**Peroneal Artery Perforator Free Flap on the Palm and Removal of Back for eign Body in High-Voltage Electrical Burn Patient: A Case Report**

**Sung Won Jung**
  Department of Plastic and Reconstructive Surgery, Hanil General Hospital

**Objectives**: The wounds caused by high-tension electrical burns are deep and complex. We performed peroneal artery perforator free flap on the palm (the entry point of electrical burn) with good results. The exit-point wound on the lower back healed and recurred repeatedly after the burn due to a for eign body—a wire. A metallic for eign body may cause serious damage to the tissues adjacent to the metals by electrical burn.
**Methods**: A 38-year-old male patient was transferred due to high voltage electrical burn. The entry point was on the right palm; the exit points were on the left foot and left lower back on the posterior superior iliac spine (PSIS). The palm wound was deep and complex. On postburn day 4th week, the peroneal artery perforator free flap was performed on the right hand. The donor site was midportion of right lower leg. The flap was 15 cm × 6 cm sized, the vascular pedicle length of flap was 4.0 cm, and the arterial diameter was 1.0 mm. The recipient artery was the third common palmar digital artery; recipient veins were two comitant veins. One artery and two veins were anastomosed each. At PSIS, a 1.5 cm-sized wound healed and recurred for 6 months after burn injury. The pelvis AP radiograph showed a 3 cm × 4 cm-sized radiopaque density in the left pelvic area. Pelvic CT scan revealed radiopaque for eign body within the left gluteus maximus. The history showed that the patient had suffered a buttock injury in his childhood. The wound was explored. Within the gluteus maximus muscle, 2.5 × 3.0 cm-sized multi-braid wires were found.
**Results**: The flap was well and the ROM of the hand was good. The wound on the PSIS healed well after the removal of wires. The peroneal artery perforator flap was thin and anatomical variation was low. Primary closure of the donor site was possible, and the length of the vascular pedicle fit well in the recipient vessels of the palm. We suspected that the wire for eign body in the gluteus maximus caused a chronic wound by electrical burn. Many metallic medical devices within the body, such as metal plates in orthopedics, cardiac pacemakers, and stents in the vessels or ducts, may cause thermal damage to the adjacent tissues by electrical burn.
**Conclusions**: The peroneal artery perforator free flap is useful in palm resurfacing in high-voltage electrical. Additionally, burns. Patients with metal in their body should be careful to avoid electrical burn.

*P.116*

**Biological Dressing of Cultured Live Human Keratinocytes Epifast^®^ to Care for Skin Graft Donor Site**

**Pablo Rodriguez-Ferreyra and Omar Gayosso**
  Instituto de Salud del Estado de Mexico

**Introduction**: Various methods have been advocated for to care for a skin graft donor site. They include open-air exposure to an application of a thin autologous skin graft. An application of microporous paper dressings with radiant local heat has been the approach commonly used in Mexico. Spontaneous separation of the dressing from the wound bed occurs after 12 to 15 days as the healing of graft donor sites takes place. A biological dressing, composed of cultured live human keratinocytes (special gauze with three to five layers of keratinocytes), was assessed for its usefulness in caring for a graft donor site. The findings for med this report.
**Material and Methods**: A total 2600 files from patients who once received autografts from us and in which Epifast was used to cover the donor site were reviewed.
**Results**: From total of more than 4000 patients from 2006 to 2022, in this review we included 2600 files of patients in who once received autografts from us and in which Epifast was used to cover the donor site. The sample was composed of 55% boys and 45% girls, the age of patients ranged from the youngest of 1 year to the oldest of 17 years, with a mean age of 7.6 years. They had sustained burn injuries with a body surface involvement ranging from 2 to 45% and an average BSA of 29%. The hospital stay was 1 to 32 days with an average of 22 days. The wound consequential to skin graft harvesting was covered with the biological dressing of human keratinocytes; the healing of the skin graft donor sites was uneventful. The time required to complete the re-epithelialization processes for the area covered the biological dressing of human keratinocytes was between 5 and 7 days.
**Conclusions**: The healing time noted in a wound consequential to skin graft harvest was short if it was covered with the biological dressing of human keratinocytes, Epifast. In previous comparative work, we demonstrated that the time required to complete the re-epithelialization processes for the area covered with microporous paper dressing varied between 12 and 15 days. Then, with this patient review, we supported the use of biological dressings on the donor site.

*P.117*

**Combined Use of Versajet^®^ and Epifast^®^ in Treatment of Partial Thickness Burns**

**Pablo Rodriguez-Ferreyra and Omar Gayosso**
  Instituto de Salud del Estado de Mexico

**Introduction**: Various regimens to manage superficial burn injuries have been advocated for. They varied from covering the burned areas with antimicrobials to the early debridement of burned wounds and skin grafting. Although death caused by superficial burns is uncommon, the morbidities attributable to a lengthy hospital stay and the cost of treatment can be extensive. A surgical regimen to manage burned wounds that comprises debridement with Versajet^®^ and covering the resultant wounds with Epifast^®^, a cultured live human keratinocytes imbedded in a allogenic collagen matrix, was reviewed for this report.
**Materials and Methods**: A total of 256 files of patients who sustained superficial burns between October 2006 and February 2020 were included in this review.
**Results**: of the files of patients included, 56% were boys and 44% were girls. The age ranged from 1 to 15 years old, with a mean age of 3.4 years. The body surface area burned varied between 16 and 50%. The average area of involvement was 24.30%. The etiology was scald in 100% of cases; they were resuscitated and taken to the operating room within 36 h of the injury. There were no deaths in this group of patients. The length of hospital stays varied between 9 and 14 days, with an average of 11.2 days. Most of the patients healing with one surgery, Versajet+Epifast; the more extensive burns were treated with 2nd surgery, “tangential scarectomy+versajet+epifast”, and a small number of patients received a third surgery with autograft. The frequency of burn wound debridement was between 4 and 6 days. The healing of the burned sites was uneventful in the patients that required only one surgery; furthermore, scarring was noted to be minimal and there was no clinical evidence of infections. To date, this patient is only skin hypochromic, characteristic of a burn caused by a scald.
**Conclusions**: The hydroscalpel, Versajet^®^, was a useful tool in removing the devitalized epidermis in burn patients. The resultant wounds were readily covered with Epifast^®^. Recovery from the injury and the surgical treatment was uneventful; the hospital stay averaged slightly less than 11 days and the cost of care for this regimen of burn treatment was one-half of a conventional approach in patient management in Mexico, because the average hospital stay in Mexico per patient with 25% of TBSA is 20 days as a maximum, transferred to the operating room three times and with a risk for 20% of patients getting hypertrophic scars.

*P.118*

**“All In” Interdisciplinary Cooperations and Individualized Innovative Care for Patients with Severe Burn Injuries**

**Tamas Püski, Maresa Berns and Bert Reichert**
  Klinikum Nürnberg

**Introduction**: The advantage provided by dedicated centers for patients with severe burn injuries is that an experienced. Interdisciplinary team is able to handle the severe complications that may arise together with these types of injuries. The significance of a functioning interdepartmental collaboration among the plastic surgeon and the anesthesiologist It is widely recognized for achieving the best possible patient outcome. The patient in our case also benefited profoundly from the efficient cooperation of four separate departments.
**Overview**: We present a 50-year-old man with severe burn injuries and serious complications. Only by applying personalized innovative methods tailored to the actual state of the patient were we able to achieve a desirable outcome.
**Case Review**: Together with the hydrotherapy team at admission, an acute enzymatic prophylaxis against compartment syndrome on both hands and both calves was applied. after enzymatic debridement, the wounds were tended to with skin grafts, Kerecis, Epicite und Suprathel. The patient suffered an acute STEMI on the third postoperative day, by which a recanalization of the coronary artery with interventional methods was performed, followed by a subsequent implantation of an ECLS device in the cardiac surgery unit. We were able to continue the individualized plastic surgical care while the patient was treated for other conditions by the interdisciplinary team.
**Summary**: The optimal care for patients with severe burn injuries requires interdepartmental collaboration. Without the emergency procedures accompanied by our plastic surgical care, the patient would not have been able to leave the hospital on his own feet with his wounds almost completely healed.

*P.119*

**Chronic Osteomyelitis of Cranial Bones in a Patient with High-Voltage Electrical Burn on the Scalp: A Case Report**

**Sung Won Jung**
  Department of Plastic and Reconstructive Surgery, Hanil General Hospital

**Objectives**: High-voltage electrical burn injuries on the scalp often result in scalp and cranial bone necrosis, requiring rich, vascularized flap coverage. Despite successful flap coverage, chronic osteomyelitis of cranial bones may occur. Treatment of chronic osteomyelitis is surgical debridement and re-coverage by a well-vascularized flap.
**Methods**: A 37-year-old man sustained a 22,000-volt electrical burn injury to his scalp. The entry point of the electrical current was on the right side of the parietal scalp. The exit points were on right side of the face and neck, shoulder and chest, for earm and hand. At first, a 4 × 3 cm-sized discolored scalp wound was noted with visible charring at the parietal scalp. It had enlarged to 12 × 12 cm with cranial bone exposure. Debridement of the cortical bone and coverage by the latissimus dorsi (LD) myocutaneous (MC) free were performed on PBD 45. The inner cortex of the bone was intact and preserved. after the operation, the LD MC flap survived well. Thirty-seven days after the successful LD free flap surgery, a small opening was noted on the posterior site of the scalp wound, accompanied by a small amount of discharge. The brain MRI and skull CT and whole-body bone scan showed signs of cranial bone osteomyelitis at the previous burn injury site. Surgical exploration of the scalp wound was performed on POD 12 months. A sinus tract, approximately 11 cm long, was found in the posterior to anterior direction. A 2 × 3 cm undermined area was noted at the mid-portion of the sinus tract. The scalp flap (previous LD flap) was incised along the sinus tract. A definite discolored and necrotic bone segment (sequestrum), 2 × 3 cm in size, and another 2 × 3 cm-sized discolored area on the cortical bone were noted, which were debrided. The inner cortical bone was viable and preserved. The elevated overlying muscle flap was replaced and closed.
**Results**: The wound healed well without recurrence for 20 months.
**Conclusions**: High-voltage electrical burn on the scalp is treated by repetitive debridements and coverage by a rich vascularized flap. Despite successful flap coverage, chronic OM of the skull may occur. The treatment of chronic OM is surgical debridement and well-vascularized flap coverage. The LD muscle free flap is suitable not only for coverage of the skull, but also for the treatment of chronic OM of the skull.

*P.120*

**Reconstruction of Post-Burn Neck Contractures in Children: Our Experiences and Challenges in Three Case Reports**

**Thibault Trevidic and Ruzsena Bene**
  Bethesda Children Hospital

**Introduction**: Post-burn neck contractures are not only an aesthetical problem, but also a functional one. An excellent long-term solution is flap reconstruction. Our three cases will show the challenges and results obtained after the use of different local transpositional flaps following expander implant augmentation.
**Methods**: Our patients had multiple post-burn neck contractures with multiple types of reconstruction (full-thickness skin graft transplant, integra+ graft or sometimes expander implant reject).
We rejected patients with poor hygiene and patients with non-cooperative parents.
**Results**: In all cases, we had positive outcomes with no tissue necrosis. However, not all cases were straightforward; one patient needed repositioning of their expander due to wound dehiscence and one developed hypertrophic scars following primary wound healing.
**Conclusions**: Transpositional flap reconstruction with prior expander implant augmentation is an acceptable treatment for post-burn neck contractures with satisfactory outcomes. Furthermore, free tissue transfer flaps are also an acceptable solution. However, in children, a tissue expander is more commonly used for primary closure of the tissue donor site.

*P.121*

**Bilayered Skin Reconstruction Using Glycerolised Acellular Dermis (Glyaderm) as a Dermal Replacement Simultaneously with Split-Thickness Skin Grafts Leads to a Long-Term Improvement in Patient Satisfaction**

**Ignace De Decker, Henk Hoeksema, Jozef Verbelen, Petra De Coninck, Els Vanlerberghe, Sofie De Schepper, Ali Pirayesh, Phillip Blondeel, Stan Monstrey and Karel Claes**
  Ghent University Hospital Burn Center—Department of Plastic, Aesthetic and Reconstructive Surgery

**Objectives**: The absence of almost the entire dermis is inherent to the use of autologous split-thickness skin grafting (STSG), which often results in contractures and/or rigid and thick hypertrophic scarring. Numerous dermal substitutes have been developed, but many of them, unfortunately, with variable results in terms of improvement compared with the use of STSG alone. Additionally, the high costs involved have been acknowledged as an important drawback and have been reported to for m one of the main reasons for the reluctant attitude of surgeons concerning its use. Bilayered skin reconstruction using the human-derived glycerolized acellular dermis (Glyaderm^®^) has already been reported to result in significantly improved scar elasticity, but this is the case when making use of a two-step procedure. In the current clinical trial, we investigated the use of Glyaderm in a single-stage setting.
**Methods**: A prospective, controlled, randomized, intra-individual, single-blinded study was performed, investigating the simultaneous application of Glyaderm^®^ and STSG versus STSG alone in full-thickness burns or comparable skin defects in 82 wound comparisons (66 patients). during the acute phase bacterial load, graft take (primary outcome) and time to wound closure were assessed. Scar quality (secondary outcomes) was evaluated at 3-, 6-, 9- and 12-month follow-ups using subjective and objective scar measurement tools. Biopsies for histological analysis were taken at 3- and 12-month follow-ups.
**Results**: Graft take (>95%), pain management and wound healing time were optimal and comparable in both groups. No Glyaderm or autografts were lost in the immediate post-operative phase. At 1 year follow-up, the patients subjectively preferred the sites that received dermal substitution. The majority of patients attributed the difference to an improved skin sensation. Histological analysis of Glyaderm-treated sites showed the presence of a well-formed neodermis, with donor elastin present up to 12 months.
**Conclusions**: Simultaneous bilayered reconstruction with Glyaderm^®^ for the restoration of full-thickness skin defects in a single-stage procedure results in optimal graft take, does not lead to loss of dermal substitutes nor the covering autografts due to infection, and provides improved patient satisfaction—the presence of a highly durable collagen-elastin based matrix has been demonstrated 1 year post-application.

*P.122*

**The Effect of Post-Injury Duration on Perforator Flap Success**

**Cagri A. Uysal ^1^, Burak Ozkan ^1^ and Mehmet Haberal ^2^**
  ^1^  Baskent University Faculty of Medicine, Department of Plastic, Reconstructive and Aesthetic Surgery
  ^2^  Baskent University Faculty of Medicine, Department of General Surgery and Burn Center

**Introduction**: Perforator arteries raised from muscles and the intermuscular septum are generally protected in burn injuries. Thus, pedicled perforator flaps are considered more reliable than conventional flaps in burn reconstruction. The role of pedicled perforator flaps changes according to injury timing. In the acute phase of injury (first 21 days), pedicled perforator flaps are used to cover exposed neurovascular and musculoskeletal systems. In the late phase of burn injuries, they are used to correct burn-related complications such as contractures and persistent defects. In this study, we present our experience with pedicled perforator flaps in both the acute and chronic phases of burn injury.
**Material and Methods**: Between 2017 and 2020, our center performed 25 pedicled perforator flap procedures in 20 patients (17 men/3 women) for burn injury reconstruction. of the 25 total pedicled perforator flaps, 4 were utilized for acute defects and 21 were performed during the late phase of injury. In the acute phase, the main indication was to cover exposed vessels. In the late phase, 16 of the 21 flaps were used to cover defects (burn scar contracture release in axilla, neck, popliteal, and antecubital region), 2 were used to cover pressure ulcers due to prolonged hospitalization, and 2 were used for persistent defects in the extremities.
**Results**: Average age of patients was 34 years (range, 28–62 years). Our success rate with pedicled perforator flaps was 88%, with two total losses (one supraclavicular artery perforator flap, one superior epigastric artery perforator flap) and one partial necrosis (superior gluteal artery perforator flap) encountered in the late phase in flap with 180-degree rotation arc.
**Conclusions**: The role of pedicled perforator flaps varies depending on the timing of burn injuries. We found that pedicled perforator flaps were a reliable reconstruction option in both acute and late phases of burn injury.

*P.123*

**Reconstruction of Burn Contractures with Free Anterolateral Thigh Flap in Various Anatomic Sites**

**Burak Ozkan ^1^, Cagri A. Uysal ^1^, Ulas Z. Bali ^2^ and Mehmet Haberal ^3^**
  ^1^  Baskent University Faculty of Medicine, Department of Plastic, Reconstructive and Aesthetic Surgery
  ^2^  Celal Bayar University Faculty of Medicine, Department of Plastic and Reconstructive Surgery
  ^3^  Baskent University Faculty of Medicine, Department of General Surgery and Burn Center

**Background**: Burn contractures that cause a restriction in extremity movements have to be reconstructed. Free microvascular flaps are generally needed in cases of severe contractures. The ideal free flap for severe contracture defects has to have a large skin island without bulk and a long pedicle for preventing recurrence and tension-free adaptation. The anterolateral thigh flap (ALT flap), which possesses these features, has widely been used for several indications in reconstructive surgery. The usage of an ALT flap in burn contracture was described for burn and axillary contractures in the literature. In this study, the usage of free ALT flaps in various anatomic contracture sites was reported.
**Methods**: Fifteen free ALT flaps were performed in 14 (12 male, 2 female) patients with a mean age of 36.6. Burn contracture defects in neck, axilla, popliteal, cubital region, plantar foot and hand were reconstructed with ALT flap.
**Results**: No total flap loss was encountered. Distal flap necrosis was observed in one case. All patients had significant improvement in a range of motions. Recurrence in contracture was seen in one patient with hand flexor contracture due to a lack of physical treatment.
**Conclusions**: ALT flap can safely be used in various anatomic contracture sites. Suprafascial elevation of the flap can be preferred for better adaptation in the neck, hand and foot and prevention of bulky appearance.

*P.124*

**Reconstruction of Finger Contracture with an Expanded First Dorsal Metacarpal Artery Perforator Flap**

**Burak Ozkan ^1^, Cagri A. Uysal ^1^, Ayse Ebru Abali ^2^ and Mehmet Haberal ^2^**
  ^1^  Baskent University Faculty of Medicine, Department of Plastic and Reconstructive Surgery
  ^2^  Baskent University Faculty of Medicine, Department of General Surgery and Burn Center

**Objectives**: The first dorsal metacarpal artery perforator (DMCAP) flap is frequently used to cover bones, tendons, and neurovascular structures in the hands and fingers after trauma and burns. Free flaps are used when soft tissue defects in the finger cannot be closed with locoregional flaps. However, the thickness of the free flaps may impair the function and aesthetics of the hand. This study will present a case in which finger contracture reconstruction was performed with an expanded first DMCAP flap after an electrical burn.
**Case Report**: A 9-year-old male patient applied to our clinic with the complaint of inability to open the second finger of the left hand after an electrical burn. Physical examination revealed distal interphalangeal (DIP) proximal interphalangeal (PIP) joint contractures. Session expanded first DMCAP flap. In the first session, under general anesthesia, a tissue expander area of 5 × 3 cm, reaching the second metacarpal level from the dorsum of the hand, was marked. A 16 mL 5 × 3 cm tissue expander was placed in the prepared area from the vertical incision. The tissue expander was inflated with 4 mL of isotonic solution. This was enlarged six weeks later by giving 22 mL of isotonic solution. In the second session, the contractures were released. Fibers came from the fascia. after the pedicle dissection was completed, the 9 × 3 cm DMCAP flap was elevated. With 180 degrees of rotation, the left hand second finger was adapted to the 6 × 2 cm defect.
**Results**: This is the first case in the literature where finger contracture reconstruction was performed with an expanded DMCAP flap. Unfortunately, free flap surgery becomes difficult due to damage to the arterial and venous system in the acute period after electrical burns. Therefore, safer locoregional flaps should be preferred. However, in cases where the tissue defect cannot be closed with locoregional flaps, extra tissue can be provided using tissue expanders. In addition, primary closure of the donor area, appropriate flap thickness for the finger, and aesthetically pleasing results are the advantages of the expanded DMKA flap. However, the patient should be followed closely in tissue expander applications in the upper extremity, and pain and finger circulation should be constantly questioned.
**Conclusions**: Expanded DMCAP flap can safely close volar large defects in the fingers.

*P.125*

**Reconstructive Surgery of Upper Extremity after Thermal Burns—Guidelines or Experience**

**Albin Stritar, Luka Emeršič and Klemen Lovšin**
  Department of Plastic, Reconstructive, Aesthetic Surgery and Burns, University Medical Centre Ljubljana

**Objectives**: Hand burns hold a special place in the field of burn care, even though the surface area of the hand comprises approximately 1% TBSA. This is due to their anatomical, functional and aesthetic characteristics. Surgical treatment is a priority in these types of burns, especially on the dorsum of the hand and fingers.
**Methods**: Early tangential excision is the method of choice for treating deep dermal and subdermal hand burns. In these cases, surgery yields considerably better functional and aesthetic outcomes, as opposed to conservative therapy. Consequently, hands are given priority over other parts of the body in surgical interventions.
Physiotherapy and rehabilitation play a very important role in the treatment of hand burns. Functionality of the hand is of paramount importance for the quality of life of burn patients. Some general rules apply for the treatment of hand burns, i.e., an individualized approach, careful patient selection and operative therapy based on the principles of the reconstructive ladder–skin graft, local flap, distal flap, dermal matrix substitute, free flap, and skin expansion.
**Results**: A patient’s psychophysical state and participation are undoubtedly important; they have to be monitored in addition to providing the patient with objective information regarding their treatment. The treatment should be opportune and carefully planned regarding the expected timeline as well as the prediction of the end result. Postoperative follow-up is lengthy, several operations may be required, and the rehabilitation program must complete the whole treatment.
**Conclusions**: Guidelines and experience occasionally differ. The for mer primarily protect logistic and for ensic facts, while the latter expresses operative, surgical and patient’s outcomes. Nevertheless, hand reconstruction, be it primary or late, should abide by the protocol without sudden changes or improvisation, while being in accordance with the expected timeline and burn scar maturation.

*P.126*

**Superficial Temporal Fascia Flap in Burn Patients: Old However, Savior—A Case Report**

**Abbas Albayati ^1^, Burak Ozkan ^1^, Cagri A. Uysal ^1^ and Mehmet Haberal ^2^**
  ^1^  Baskent University Faculty of Medicine, Department of Plastic and Reconstructive Surgery
  ^2^  Baskent University, Faculty of Medicine, Burn and Fire Disasters Institute

**Introduction**: Deep burn wounds over hands and foots need thin and pliable flaps to cover underlying structures and to provide a free range of motion. In this case, we present the use of free superficial temporal fascia flap for coverage of third-degree burn on the lateral side of left foot and ankle joint.
**Methodology**: A twenty-eight-year-old male patient presented to our burn care unit with 30% total surface area of second-, third- and fourth-degree burns due to an electrical burn. after primary survey and patient stabilization, extensive debridement was conducted to remove the eschar tissues of third- and fourth-burneddegree-burnt areas. after debridement, there was a tissue defect measuring 16x11 cm over the lateral aspect of left ankle and foot with an exposure of the head of fifth metatarsal bone and the tendons of both peroneus longus and brevis muscles (Figure 1). A plan was devised to cover the wound with a free superficial temporal fascia (STF) flap. First, the dorsalis pedis artery and accompanying vein were prepared as recipient vessels for free tissue transfer. Then, a T-shaped incision was made over the left temporal region. after skin incision, a sharp and meticulous dissection was carried out over STF. A flap of 12 × 10 cm in size was harvested based on the superficial temporal artery and vein. The flap was adapted over the wound of the foot. The donor vessels were anastomosed dorsalis pedis artery and accompanying vein. Then, a split-thickness skin graft taken from the right leg was applied over the fascia flap (Figure 2). A posterior short leg splint and bandage was applied. Postoperatively, the leg was elevated and kept warm with external warming device.
**Results**: The flap was monitored regularly every 2 h for the first 72 h followed by less frequent monitoring in the following days. There was no anastomotic problem in the postoperative period and the donor site healed well.
**Conclusions**: The superficial temporal fascia flap is an ultrathin pliable flap based on superficial temporal vessels; it is used mainly for the coverage of tissue defects over the hand and foot. The flap is ideal for the reconstruction of burn wounds over the dorsum of the foot and ankle joint, since it is the thinnest flap in the human body. The smooth areolar surface of the fascia flap makes it particularly important in providing a gliding surface in coverage of exposed tendons.

*P.127*

**Return to Short-Term Burn Reconstruction Missions to Ukraine: COVID-19-Prompted Protocol Changes**

**Robert Dabek ^1^, Alexey Vlasov ^2^, Maxim Savenko ^3^, Artem Pasunko ^2^, Daniel Driscoll ^4^, Justin Knittel ^5^ and Gennadiy Fuzaylov ^4^**
  ^1^  Ascension Saint Agnes Hospital
  ^2^  Regional Children’s Hospital
  ^3^  Dnipro State Medical School
  ^4^  Harvard Medical School
  ^5^  Washington University School of Medicine

**Objectives**: The COVID-19 pandemic resulted in tremendous challenges for healthcare systems globally. However, the increased distribution and availability of a vaccine is allowing a return to near-normal function in many regions. Within the medical community, individuals engaged in global outreach missions are assessing the risks of returning to these activities abroad. Here, we describe our return to an annual burn reconstruction mission in Ukraine.
**Methods**: Protective strategies and alterations to standard mission protocol were applied to limit exposure and transmission risk and promote education and training. We increased utilization of telemedicine and removed in person clinic visits for triage. Perioperative safety and follow-up protocols were implemented. Operative complexity was increased to maximize benefit while limiting patient exposure.
**Results**: We performed 76 reconstructive procedures on 25 pediatric patients in four days.
**Conclusions**: Through an increased use of telemedicine, elimination of in person triage, implementation of rigorous safety and follow-up protocols, and emphasis on education we improved efficiency, safety, and accountability of our outreach program. The applied protocol changes provide guidance on responsibly resuming surgical outreach programs, with alterations that are expected to remain beyond the COVID-19 pandemic.

*P.128*

**Up-to-Date Burned Hand Management Using New Technologies**

**Claudia Corrao, Giulio Maggio, Pasquale Tedeschi, Simone Magistri and Giuseppe Giudice**
  Plastic Surgery

Hand burns are a challenge for plastic surgeons since they cause functional and aesthetic problems related to invalidating scars.
  Approximately 80% of burned patients present hand burn wounds which are treated, according to guidelines, with a surgery procedure, i.e., escharectomy and partial thickness dermo-epidermal graft (standard of care).
However, there are new methods such as Nexobrid and SVF (stromal vascular fraction), which represent minimal-invasive modalities (M.I.MOs).
Nexobrid is a medicine of concentrate of proteolytic enzymes enriched in bromelain, which is applied within 48 h, coating the burned region with a 1.5 mm to 3 mm layer and removed after 4 h obtaining enzymatic selective escariolysis of the necrotic tissues.
Otherwise, SVF is obtained through physical filtration and sedimentation of lipoaspirated material set on a skin substitute (hyalomatrix) and applied to wound areas.
Our goal is to treat mid-deep hand burns with Nexobrid and SVF to obtain the best functional and morphological outcomes and to compared with SOC (M.I.MO).
From January 2021 to January 2022, we enrolled nine patients (five males and four females) with mid-deep hand burns, aged 18–65 years old, with TBSA of 20–40%, and we divided them in three randomized groups—A, B, and C—each including five burned hands (the same patient has been enrolled in different groups).
The “A group” was treated exclusively with Nexobrid, the “B group” was treated by combining Nexobrid with SVF (stromal vascular factor), and the “C group” was treated with surgical escharotomy and a partial-thickness dermo-epidermal graft.
The results show that, in the A group, patient healing was achieved in a month, avoiding a surgery procedure with a good functional recovery.
In the B group, patients achieved faster healing and a better morpho-functional outcome compared with A group’s patients.
C group’s patients obtained complete healing in 15 days with a good functional and aesthetic outcome.
To conclude, a better aesthetic and functional result in patients treated with Nexobrid and SVF (group B) was observed compared with those treated exclusively with Nexobrid (group A), and very similar to those treated with skin-grafts (group C).

*P.129*

**Upper Extremities Reconstruction in a 4-Year-Old Child with Complex Post-Burn Scars**

**Maria Grazia Cortese ^1^, Valeria Malvasio ^1^, Patrizia Magro ^1^, Elisa Zambaiti ^1^, Gabriella Naretto ^2^, Elisa Rolfo ^2^, Serena Causi ^2^, Andrea Tisone ^2^, Paola Imazio ^2^, Maurizio Stella ^3^ and Fabrizio Gennari ^1^**
  ^1^  General Pediatric Surgery, Regina Margherita Children’s Hospital
  ^2^  Rehabilitation Team of Pediatric Orthopedics Surgery, Ospedale Infantile Regina Margherita, Children Hospital
  ^3^  Burn Center, CTO Hospital

**Objectives**: The upper extremity is involved in the majority of severe burns. In developing countries, where treatment is often delayed or inadequate, burns sequelae remain a major public health problem. Follow-up is problematic, rehabilitation service is non-existent and, therefore, upper extremity burns cause important scar contractures with severe disability. Moreover, surgical reconstruction after contractures release is challenging.
**Methods**: Case report: a 4-year-old child sustained an extensive burn at upper extremities and trunk when she was 1. She was treated in a developing country. Burns sequelae resulted in severe scar contractures. In 2019, she was referred to our Center, for scar release and surgical reconstruction. She presented a complete loss of right axilla, elbow, wrist and hand function with fingers fused together.
The left upper extremity presented a severe elbow flexion and wrist extension with a claw deformity of the hand. Surgical planning included scar incisions, tenolysis, amputation of affected phalanx and resurfacing of soft tissue defects with local flaps, dermal substitutes and skin grafts. To maintain and gradually increase the range of motion, intensive physiotherapy rehabilitation started immediately and continued at home, providing education to the patient and her caregiver. The patient was hosted by a local voluntary association for the entire duration of treatments.
**Results**: We performed 12 surgical procedures from March 2019 to October 2021 for a total of seven hospitalizations. We restored a complete elbow and wrist function at left upper extremity and we obtained a good functional pollicis–digital grip, key pinch and fingers flexion–extension range of motion. for the right upper extremity, we obtained a complete shoulder abduction and elbow flexion–extension range of motion. for the hand, we obtained a limited but gradually improving functional pollicis–digital grip.
**Conclusions**: reconstructive surgeons may follow different algorithms to achieve an efficient release of burn contractures. However, the final choice must always be personalized for the patient. Due to the age of our patient, we decided not to use free flaps to preserve the donor site in a growing patient. With a complex reconstructive program, we obtained our target to return joints to a functional range of motion. We allowed the right hand, at least, to grasp some objects. Open critical point in the treatment of these patients, coming from developing countries, despite a local reference, remains the possibility to maintain a remote and continue follow-up.

*P.130*

**Soft Tissue and Nerve Reconstruction in High-Voltage Electrical Injuries: A Case Report and Review of the Literature**

**Carlos Pueyo-Morillo, Alejandra Monte, Jordi Serracanta and Joan-Pere Barret**
  Vall D’Hebrón University Hospital

**Objectives**: High-voltage electrical injuries (HVEI) can be devastating injuries with great soft tissue loss and peripheral nerve injury, which can result in the amputation of the limb, especially in the upper extremity. Since the pathophysiology is highly complex, reconstructing these burns still represents a difficult issue for the burn surgeon.
We describe the case of an HVEI in the hand with associated nerve injury and review the literature on this type of injury and its soft tissue and nerve reconstruction options.
**Methods**: A 50-year-old male who suffered an HVEI in his right hand presented at our center with a third-degree burn on the palmar side that exposed tendons. We evidenced a paralysis of the abductor pollicis brevis and the opposition of the thumb, together with anesthesia of the 1st to 4th fingers. The electromyography (EMG) indicated complete axonotmesis of the median nerve at the wrist. We performed debridement of all the devitalized tissue and covered the wound with Biobrane^®^. Ten days later, nerve repair surgery was performed using the left sural nerve divided into four grafts and sutured from the viable stump of the median nerve to the radial and ulnar digital nerves of the thumb, second and third common interdigital nerves. Additionally, the motor branch of the thenar eminence was identified and sutured. A fasciocutaneous anterolateral thigh flap was used to cover the soft-tissue defect, anastomosed to the radial artery outside the damaged area.
**Results**: The postoperative period was uneventful. A year post-surgery, the EMG showed a reappearance of motor potential in the second lumbrical and an increase in reinnervation in APB. The patient presents a progressive improvement in the global function of the hand, with a recovery of sensation until the first interphalangeal joint.
**Conclusions**: The literature shows an elevated risk of failure in microsurgical free flap reconstruction and controversy over the ideal time for definitive coverage. The most common technique for reconstructing peripheral nerves is the use of autologous nerve grafts. The success of the graft is influenced by several factors, but especially the quality of the wound bed. Free tissue transfer creates a vascularized bed for the non-vascularized nerve grafts and allows early mobilization of the limb. A multidisciplinary treatment in a specialized burn center allows for adequate surgical management of HVEI, including nerve repair and soft tissue coverage.

*P.131*

**Toxic Epidermal Necrolysis and Epidermolysis Bullosa Successfully Treated with Cultured Skin Grafts (Epifast)**

**Alfonso Masse Sanchez**
  Imss

Toxic Epidermal Necrolysis and Epidermolysis Bullosa successfully treated with cultured skin grafts EPIFAST
**Introduction**: Most cases of toxic epidermal necrolysis (TEN) are drug-induced. Immunological reactions are implicated (keratinocytes apoptosis). Epidermolysis bullosa (EB) is a disease of the skin transmitted genetically is classified (genodermatosis).
**Objective**: To find an appropriate treatment for patients with toxic epidermal necrolysis (TEN) and epidermolysis bullosa (EB) since there are no guidelines for it, and the patients behave as burned ones. Therefore, cultured skin grafts, “EPIFAST”, were used.
**Methodology**: 1,2—A Three- and eleven-year-old females developed an adverse reaction to medication; after 48 h, toxic epidermal necrolysis (TEN) had developed in 80% of the body surface (skin). 3—A 6-year-old male with a convulsive crisis, which creates an allergy to Lamotrigina at 7 days; TEN was developed on 75% of the body surface, meriting intensive care management.
4, 5—Two females, 3 and 12 years old, with Epidermolysis Bullosa on different parts of the body and infection.
**Results**: On the 1st day of treatment, Epifast was applied to five patients (50% of the lesions to patients with NET and 100% to NB) and was removed one week later, and Epifast was applied in the other 50% on the lesion (NET), the patient with EB healed with a single application of Epifast.
**Disscussion**: There are no previous toxic epidermal necrolysis or epidermolysis bullosa treatments reported in the medical literature following these procedures; in cultured skin grafts, Epifast was shown to be an ideal proven treatment for these cases, obtaining a good-quality epithelium within 5 days after application.
**Conclusions**: The surgical technique applied with cultured skin grafts, EPIFAST, demonstrated better results than expected, since adequate skin cover throughout the treated surface was achieved. It stimulates the healing and shortens the recovery period of the patient.

*P.132*

**Eyelash Reconstruction Utilizing a Composite Strip Graft for Burn-Related Madarosis: Case Reports and Operative Technique**

**Robert Dabek ^1^, Fawaz Araim ^1^ and Daniel Driscoll ^2^**
  ^1^  Ascension Saint Agnes Hospital
  ^2^  Harvard Medical School

**Objectives**: Reconstruction of the periocular region after burn injury is technically difficult. Restoring the functional status and ensuring preservation of vision is essential. Recreating the eyelid margin and eyelashes can prove to be challenging. Although there are several options for eyelash grafting, there is no established gold standard, and there is little-to-no literature related to procedures involving burn madarosis. **Methods**: Here, we describe two cases of eyelash grafting, utilizing a composite strip graft in severe facial burn injury with madarosis. **Results**: The presented results show the efficacy of this technique at restoring function and providing a good aesthetic result. **Conclusions**: The utilization of a composite strip graft to recreate the eyelid margin is a viable choice for reconstruction of burned upper eyelids in the appropriate patient. Further explorations regarding procedure timing and choice of donor site are warranted.

*P.133*

**Experience in Using Biobrane™ in the Treatment of Severe Burns in Children at the Bulgarian National Burn Center**

**Maya Argirova, Anastasiya Viktorova, Yolanda Zayakova and Dario Carlo Premuselli**
  Department of Burns and Plastic Surgery, UMHATEM “N. I. Pirogov”

**Objectives**: The excision of burn eschar in large burns is a key component of treatment as well as prevention of infection. Early excision and autografting of burns patients is the gold standard for acute burn care. Children suffering from large burns undergo serial stages of operations depending on the extent of injury and often require temporary coverage with an allograft or skin substitutes. Allograft serves as an effective option for temporary burn wound covering. Due to its shortcomings, we started using a biosynthetic dressing, Biobrane™. The aim of this study was to evaluate the therapeutic effect of Biobrane™ in the treatment of excised deep dermal and full-thickness burns as temporary biosynthetic wound coverage. **Methods**: Between January 2017 and December 2021, a retrospective review of children with extensive deep dermal and full-thickness burns was performed. A total of 54 patients, aged 1–14 years, were included in this study. Demographic data were collected from the hospital records. The early excision (tangential, fascial) was carried out in stages, each procedure being limited to 10–30% TBSA. after excision and meticulous hemostasis, all wounds were covered with Biobrane™ as a temporary wound coverage. Biobrane™ was placed firmly in contact with the excised surface and held in place with surgical staples. In the cases with subsequent skin autografting, Biobrane™ was applied on autograft areas, donor sites and superficial burns as well. The clinical efficiency of the skin substitute was analyzed according to the following parameters: adherence, presence of fluid collection, rejection, infection and follow-up wound evaluation. **Results**: The scalds were the most common mechanism of injury (84.8%). The percentage of TBSA was ranged from 20% to 68%. Biobrane™ reduced pain, decreased exudative protein loss, limited bacterial growth, protected the underlying tissues and enhanced the healing of partial-thickness wounds and donor sites. Upon removal of Biobrane™, the granulation tissue was appropriate for skin autografting. Hematoma for mation was observed in seven cases without the need to change Biobrane™. Infected exudate underneath Biobrane™ was reported in three cases 10 days after the placement. Biobrane™ was not removed until the wounds were successfully autografted. **Conclusions**: Biobrane™ is effective and easy to use as a temporary wound coverage after early excision in extensive burns. The permeable silicone outer layer allows the use of antimicrobial agents and the drainage of fluids. Biobrane™ protects excised burn wounds from bacterial contamination and dehydration, at the same time preparing the wound for autografting.

*P.134*

**Biocompatibility and Cytotoxicity of Alternative Antimicrobials for Burn Wound Regeneration with Biological Bandages**

**Philippe Abdel-Sayed, Sandra Jaccoud, Lee Applegate**
  Chuv

**Objectives**: Severe burns destroy the skin, the primary body barrier against pathogens, thus implicating severe pathophysiology conditions. Nosocomial infections remain the major persistent challenge in burns, replacing the traumatic injury as the major cause of morbidity and mortality. This issue is exacerbated by the arrival of antibiotic resistant strains. Biological bandages made of progenitor skin cells seeded on collagen scaffold have offered a treatment option for severely burned patients. However, despite promoting natural regeneration, these band-aids lack antimicrobial properties. Our objective was to investigate the use of alternative antimicrobials with biological bandages to positively impact the wound healing process due to a simultaneous release of growth factors and antimicrobials.
**Methods**: Local Swiss black Propolis, three for mulations of cannabidiol (CBD) and methyglyoxal (MGO), the major antimicrobial component in manuka honey, were tested for their cytotoxicity and biocompatibility. Thus, growth curves of cultured human progenitor fibroblasts up to 14 days in presence of each antimicrobial were used to determine cytotoxic concentrations. Likewise, in the ovo, a chorioallantoic membrane (CAM) assay was used to assess the angiogenesis and the biocompatibility of the different antimicrobials.
**Results**: Propolis at 1 mg/mL and 10 mg/mL were cytotoxic, but at 100 ug/mL, the viability of fibroblasts was comparable to the control. for MGO, we determined that concentrations of above 1 ug/mL were cytotoxic. Additionally, only one of the three CBD for mulations was determined cytocompatible for concentrations between 25 and 100 ug/mL. It was previously reported that propolis MIC is around 80 ugL/mL, 1–5 μg/mL for CBD and 512 μg/mL for MGO, respectively. Thus, cytocompatible concentrations for Propolis and CBD should still be potent against bacterial infection, but not for MGO. Interestingly, qualitative CAM assay results showed that CBD at 50 ug/mL involved an enhanced neovascularization after 48 h incubation, while incubation with MGO implied damaged blood vessels. Similar destructive CAM results were observed with propolis, but most probably because of the high concentration of ethanol in the solvent, since a lack of biocompatibility was also observed in the control.
**Conclusions**: As neovascularization is pivotal in the healing of deep burn wounds, the use of CBD could potentially improve healing processes with an enhanced angiogenesis, while suppressing bacterial growth. Further investigations are planned in order to determine synergistic effect of the above-mentioned antimicrobials with the biological bandages, and subsequently, an in vivo study to confirm the effects of antimicrobials on wound healing and bacterial infection.

*P.135*

**Standardized Cytotherapeutic Chimeras: Optimizing Cultured Allodermal–Autoepithelial Grafts for Post-Burn Regenerative Medicine**

**Nathalie Hirt-Burri ^1,2,3^, Pharm Alexis Laurent ^1,2^, Wassim Raffoul ^1,3^, Katya Rémi ^2^ and Lee Ann Applegate ^1,2,3^**
  ^1^  Plastic, Reconstructive and Hand Surgery Service, University Hospital (CHUV)
  ^2^  Regenerative Therapy Unit (UTR), University Hospital (CHUV)
  ^3^  Lausanne Burn Center, University Hospital (CHUV)

**Objectives**: Three decades of clinical practice and hindsight have been gathered in the Lausanne Burn Center around the cytotherapeutic use of cultured dermal-epidermal autografts (CDEA) in severe burn cases (i.e., deep partial- and full-thickness burns >70% TBSA). Treatments with an autologous bilayer CDEAs (i.e., fibroblasts and keratinocytes) may result in minimal skin retraction in vivo following application with dermal substitutes (e.g., Matriderm^®^, Integra^®^). Despite superior qualitative outcomes as compared with cultured epithelial autografts (CEA), CDEAs require longer manufacturing delays (i.e., 6–8 weeks versus 3–4 weeks for CEAs), restricting the scope of their potential clinical uses. Specifically, autologous fibroblasts require in vitro expansion and subsequent vitamin C stimulation for optimal generation of collagen, eventually adopting a structural role in the complex graft. Therefore, the objective was to evaluate the performance of clinically implemented allogeneic progenitor fibroblasts (e.g., FE002-SK2 primary cell type) for potential substitution of autologous fibroblasts in CDEAs, for the optimization of product standardization and shortening of clinical availability delays.
**Methods**: FE002-SK2 progenitor fibroblasts were compared with several burn patient dermal fibroblast cell types in terms of collagen synthesis (i.e., revealed by sirius red staining) under vitamin C stimulation in vitro. Structural evaluations and primary functional assessments (i.e., ability to for m an adherent cutaneous barrier) were then comparatively performed in vitro and ex vivo on CDEAs (i.e., manufactured with the various investigated sources of fibroblasts) using several cell staining solutions (e.g., HE, MTT) and de-epidermalized skin (DED) scaffolds.
**Results**: Overall, manufacturing periods of progenitor-fibroblast-containing CDEAs fell within 3–4 weeks. Furthermore, FE002-SK2 cells outperformed burn patient fibroblasts in vitro in terms of collagen synthesis capacities under vitamin C stimulations, and all the investigated bi-layer cytotherapies were able to adhere to DED.
**Conclusions**: The use of safe and standardized primary progenitor fibroblasts bares great potential for substitution of autologous fibroblasts in CDEAs. In addition to improved structural properties and equivalent behaviors ex vivo, optimized cultured allodermal–autoepithelial grafts may be manufactured with half the delay of a fully autologous CDEA. This complex skin bioengineering approach, based on historically validated regenerative medicine best practices, may tangibly contribute to the limitation of morbidity and mortality in severe burn patients.

*P.136*

**Lyophilized Dermal Progenitor Fibroblasts for Off-the-Shelf Progenitor Biological Bandages in Post-Burn Regenerative Medicine**

**Alexis Laurent ^1,2^, Nathalie Hirt-burri ^1,2,3^, Corinne Scaletta ^1,2,3^, Murielle Michetti ^1,2,3^, Wassim Raffoul ^1,3^ and Lee Ann Applegate ^1,2,3^**
  ^1^  Plastic, Reconstructive and Hand Surgery Service, University Hospital (CHUV)
  ^2^  Regenerative Therapy Unit (UTR), University Hospital (CHUV)
  ^3^  Lausanne Burn Center, University Hospital (CHUV)

**Objectives**: Cultured progenitor dermal fibroblasts (e.g., FE002-SK2 cell type) have been studied in Swiss translational regenerative medicine for over three decades, wherein thorough allogeneic cytotherapeutic clinical experience was gathered for safely managing burns and refractory cutaneous ulcers. Inherent technical advantages of primary progenitor cells include high robustness, optimal adaptability to industrial GMP manufacture, and great potential for effective repair stimulation of wounded tissues. By contrast, major technical bottlenecks in current cell therapy development comprise sustainability, stability, and logistics of biological material sources. The objective of the study was to further optimize and up-scale the processing (i.e., primary cell biobanking workflows and cell stabilization by lyophilization) of FE002-SK2 cells, with the objective of addressing potential cell source sustainability and product stability issues in cutaneous cell-based regenerative medicine.
**Methods**: Firstly, multi-tiered in vitro progenitor cell banking was optimized in terms of overall process quality and efficiency by benchmarking of key reagents (e.g., cell proliferation medium supplement source, cell dissociation reagent), consumables (e.g., cell culture vessels), and ad hoc technical specifications. Secondly, primary progenitor cell stabilization by classical lyophilization was undertaken with the objective to maintain the key or critical functions of the devitalized cells, potentially enabling high logistical gains.
**Results**: Fetal bovine serum batch identity and cell culture vessel surface were confirmed, among other parameters, to largely impact the endpoint harvest cell yields of FE002-SK2 progenitor cells. The proposed ad hoc cell banking workflows, currently implemented in the Lausanne Burn Center for authorized investigational cytotherapeutic application, potentially enable the consistent GMP manufacture of several billion therapeutic products for allogeneic homologous clinical use. The use of appropriate lyoprotective sugar-based solutions (e.g., sucrose, dextran) enabled effective FE002-SK2 progenitor cell stabilization by lyophilization, resulting in the maintenance of important qualitative and functional attributes of the processed biological materials, as compared with equivalent fresh cell preparations. Specifically, protein contents (i.e., clusters of differentiation, cytokines, growth factors) and in vitro stimulatory potentials (i.e., proliferation and migration promotion of target primary fibroblasts and keratinocytes) of selected progenitor cell lyophilizates were shown to be of interest for cutaneous wound closure and tissular repair.
**Conclusions**: This study provided a tangible and systematic technical basis for the elaboration of next-generation off-the-shelf topical cytotherapeutic products for wound healing and post-burn applications. Critical aspects such as primary cytotherapeutic cell source sustainability and stability were addressed, further enabling the implementation of safe and standardized cell-based solutions in regenerative medicine for burn patient clinical care.

*P.137*

**Novel Method in Surgical Treatment of Hidradenitis Suppurativa: Co-Graft of ADM and STSG**

**Marcin Gierek, Wojciech Łabuś, Karolina Ziółkowska and Artur Wielgórecki**
  Dr Stanisław Sakiel Centre for Burns Treatment

**Objectives**: Hidradenitis suppurativa (Acne inversa) is a chronic disease characterized by recurrent, painful, deep-seated, nodules and abscesses of apocrine gland-bearing skin. HS causes painful discomfort as well as social embarrassment, which has a negative influence on quality of life. A mean disease incidence of 6.0 per 100,000 person years and an average prevalence of 1% has been reported in Europe. Acne inversa (HS) also known as Hidradenitis Suppurativa or Verneuli Disease, is an inflammatory chronic disease of the hair follicle that presents different lesions in the apocrine gland-bearing areas of the human body. Most common areas are axillae, inguinal and anogenital regions. The presentation of the disease involves a classic distribution of painful lesions in the skin creases with associated local complications of abscesses and sinuses. The most efficient treatment of HS is surgical excision. The extended excision needs to be primary reconstructed (rotatory flaps or split-thickness skin grafts). The Centre Burns Treatment in Siemianowice Slaskie, Poland, is one of the leading hospitals in Poland in the field of hidradenitis suppurativa surgery.
**Methods**: Co-graft of ADM and STSG is still a new method. This method was used in healing of burn wounds or chronic wounds. In March 2022, we performed first co-graft of ADM+STSG in the world during HS reconstructive surgery. We would like to present the novel method of co-grafting of ADM and STSG as a new technique in large skin defects after local excicisions of axillae and buttocks in HS surgery treatment. We would like to present the method and surgical technique with ADM and STSG and 3 months follow-up after surgeries. We used laser speckle analysis (LASCA) to observe wound healing.
**Results**: Our first results are very satisfactory. We found that wound healing and scar for mation is better in ADM+STSG method vs. normal split-thickness skin grafts. ADM and STSG is a reconstructive technique in hidradenitis suppurativa surgery of buttocks and axillae, which can be widely used in HS surgery centers as Centre for Burns Treatment in Siemianowice Slaskie
**Conclusions**: To achieve very good aesthetic results in the field of HS reconstructive surgery, we recommend the co-graft of ADM and STSG.

*P.138*

**Comparison of Wound Healing and Patient Comfort in Partial-Thickness Burn Wounds Treated with SUPRATHEL^®^ and Epictehydro^®^ Wound Dressings**

**Jennifer Schiefer and Paul Fuchs**
  Cologne Merheim Medical Center, Clinic of Plastic and Burn Surgery, University of Witten/Herdecke

**Introduction**: Among the available dressings for partial-thickness burn wound treatment, SUPRATHEL^®^ has shown good usability and effectiveness for wound healing and patient comfort and has been used in many burn centers in the last decade. Recently, bacterial nanocellulose has become popular for the treatment of wounds, and many studies have demonstrated its efficacy. Epicitehydro^®^, consisting of bacterial nanocellulose and 95% water, is a promising product and has recently been introduced in numerous burn centers. To date, no studies including direct comparisons to existing products like SUPRATHEL^®^ have been conducted. Therefore, we aimed to compare epicitehydro^®^ to SUPRATHEL^®^ in the treatment of partial-thickness burns.
**Methods**: Twenty patients with partial-thickness burns affecting more than 0.5% of their total body surface area were enrolled in this prospective, unicentric, open, comparative, intra-individual clinical study. after debridement, the wounds were divided into two areas: one was treated with SUPRATHEL^®^ and the other with epicitehydro^®^. Wound healing, infection, bleeding, exudation, dressing changes, and pain were documented. The quality of the scar tissue was assessed subjectively using the Patient and Observer Scar Scale.
**Results**: Wound healing in patients with a mean total body surface area of 9.2%, took 15–16 days for both treatments without dressing changes. All wounds showed minimal exudation and patients reported decreased pain with the only significant difference between the two dressings on day 1. No infection or bleeding occurred in any of the wounds. Regarding scar evaluation, SUPRATHEL^®^ and epicitehydro^®^ did not differ significantly.
**Conclusions**: Both wound dressings were easy to use, were highly flexible, created a safe healing environment, had similar effects on pain reduction, and showed good cosmetic and functional results without necessitating dressing changes. Therefore, epicitehydro^®^ can be used as an alternative to SUPRATHEL^®^ for the treatment of partial-thickness burn wounds.

*P.139*

**Enzymatic Debridement and Stromal Vascular Fraction in Burn Wound Healing—Pilot Study and the Evolution of Minimal Invasive Modality**

**Michelangelo Vestita ^1^, Giuseppe Minunni ^1^, Giulio Maggio ^2^ and Giuseppe Giudice ^2^**
  ^1^  Perrino Hospital, Burn Center
  ^2^  Policlinico di Bari, Unit of Plastic Surgery and Burn Center

**Purpose**: Given the stromal vascular fraction (SVF) documented benefits in other fields of plastic surgery and the lack of studies on acute burns, we wanted to investigate the clinical efficacy and safety of the SVF in treating intermediate–deep burns after selective enzymatic debridement with Nexobrid^®^.
**Materials**: We enrolled 10 consecutive patients affected with thermal intermediate–deep burns in symmetrical body sites and comparable TBSA involved. for each patient, the two areas were defined as study and control following randomization. The SVF was applied over the study area after enzymatic debridement with Nexobrid^®^ and then covered by a hyaluronic acid scaffold. The control area was covered with the scaffold. Both areas were left untouched for 15 days, at which time point they were first blindly assessed by histogram planimetry wound area tracing. Further assessments were made at 20, 25 and 30 and 180 days. If any of the study or control areas had not shown signs of significant healing by the 30 days follow up, they were covered with a split-thickness autologous skin graft. At 15days, biopsies were taken from both the study and control areas to assess for CD31 expression by immunohistochemistry. The Vancouver Scar Scale and patient satisfaction VAS were also recorded at the 180 days follow up. Adverse events and complications were monitored at each visit.
**Results**: Each treated area showed presence of islands of re-epithelization and subtotal healing at 15 days, and complete healing at 30 days in all cases. Each control area failed to heal spontaneously and underwent coverage with a split-thickness autologous skin graft in all cases.
CD31 immunohistochemistry examination to assess for angiogenesis showed an increased mean vascularity in the case areas when compared with the control ones. No adverse events were recorded.
**Conclusions**: Our experience indicates a significant and early wound healing with the SVF in intermediate-deep burns after selective enzymatic debridement with Nexobrid^®^, which provides a clean and viable wound bed. Such “spontaneous” healing saves the patient further surgery, which would instead be needed to cover the defect with a skin graft, and leads to better long-term cosmetic outcomes. Therefore, an advantage in terms of patient morbidity (less surgery, less blood transfusion needed), quality of life and healthcare costs is easily for eseeable when applying this technique, which we called the “minimally invasive modality”.
This is a pilot study with a limited cohort. Further observations will be needed to confirm our results.

*P.140*

**The Effect of Remote Ischemic Post Conditioning on Stasis Zone in Acute Burn: Experimental Study**

**Cagri A. Uysal ^1^, Kadri Akinci ^1^, Abbas Albayati ^1^, Burak Ozkan ^1^, Ebru Sebnem Ayva ^2^, Tugce Sencelikel ^3^, Deniz Ilhan Topcu ^4^ and Mehmet Haberal ^5^**
  ^1^  Department of Plastic, Reconstructive and Aesthetic Surgery, Baskent University Faculty of Medicine
  ^2^  Department of Pathology, Baskent University Faculty of Medicine
  ^3^  Department of Biostatistics, Baskent University Faculty of Medicine
  ^4^  Department of Biochemistry, Baskent University Faculty of Medicine
  ^5^  Department of General Surgery and Burn Center, Baskent University Faculty of Medicine

**Introduction**: The stasis zone is the encircling area of the coagulation zone which is a critical area determining the depth and width of the necrosis in burn patients. We performed an experimental study to find out the effect of remote ischemic postconditioning, systemically and locally, on the stasis zone in burn model.
**Methodology**: Sixty Sprague Dawley rats were included into the study. Twelve rats were used to gather rat serum (4 mL each) and were divided into two groups as Group X (*n* = 6 rats) (no intervention conducted) and Group Y (*n* = 6 rats) (right lower extremity was exposed to 60 min ischemia by tourniquet and then 60min reperfusion). The serum from Group X was normal (NS) and from Group Y was remote ischemic post-conditioned (RIpS).
Thermal injury was applied on dorsum of rats (*n* = 48) according to the previously described “comb burn” model. The rats were divided into 4 groups and injections were done: Group 1: Local injection of NS.Group 2: Systemic injection of NS.Group 3: Local injection of RIpS. Group 4: Systemic injection of RIpS. All 4 groups were sub-grouped as a (*n* = 6 rats) (specimens were taken after 24 h) and b (*n* = 6 rats) (specimens were taken and macroscopic analysis were carried out).
Biochemical evaluation was done with NS and RIpS to calculate the levels of endothelial nitric oxide synthase(eNOS) inducible nitric oxide synthase(iNOS) and heme oxygenase-1(HO-1) by ELISA.
Immunohistochemical evaluation was performed to the specimens taken from the sub-group a of all groups. Histological scoring system (HSCORE) was used to calculate the immune reaction of anti-nuclear factor-like 2 (anti-Nrf-2) to nuclear factor-like 2 (Nrf-2).
Macroscopic evaluation of the viable and necrotic areas was performed to rats in sub-Group B in all groups and areas were calculated by Adobe Photoshop. Microscopic evaluation was done to the specimens taken from sub-group B in all groups to find out the quantitative amount of capillary count, inflammatory cell count, fibrosis gradient and epithelial thickness.
**Results**: There was a statistically significant difference between NS and RIpS among eNOS, iNOS and HO-1 levels (*p* < 0.01). HSCORE for Nrf-2 was statistically higher in Groups 3 and 4 compared with Groups 1 and 2. Macroscopic stasis zone tissue survival percentage was statistically high on Group 4 (46%) (*p* < 0.05). There was a statistically significant difference between Groups 1–2 and Groups 3–4 among capillary count, inflammatory cell count, fibrosis gradient and epithelial thickness (*p* < 0.05).
**Conclusions**: RIpC has been indicated to be helpful in salvaging stasis zone on acute burn injuries with the control of the ischemia–reperfusion pathways.

*P.141*

**Biological Debridement and Mucrochirurgical Coverage in Facial Electrical Burn**

**Jonathan Varela Elena, Diego Mate Martín, Alba González Rodríguez, Nerea Comellas Melero, Juan Javier García Barreiro and María Eugenia López Suso**
  Complejo Hospitalario Universitario de a Coruña

Burns represent a pathology with a high prevalence and complexity; they are injuries that require special care. Preserving as much healthy tissue as possible and creating a progranulative environment is essential. Thanks to its mechanism of action and the characteristics of the histological lesions resulting from electrical burns, larva therapy could be used as a tool in the treatment of said burns. Very promising results have been observed in patients treated by larval therapy with a protocol of local cures applied directly on the injured area, replacing the larva every 3 days for a period of 10–14 days. We report an increase in the granulation tissue, a local antiseptic effect, a selective debridement of the injured tissue and a decrease in the bloody area. The use of biological debridement combined with microsurgical techniques such as the antero lateral thigh (ALT) flap, allows for a decisive approach to be carried out in complex patients that present injuries on areas of great aesthetic and functional importance such as the facial region

*P.142*

**The Effect of Adipose-Derived Stromal Vascular Fraction on Stasis Zone in an Experimental Burn Model on Streptozocin—Induced Diabetic Rats**

**Cagri A. Uysal ^1^, Kadri Akinci ^1^, Burak Ozkan ^1^, Abbas Albayati ^1^, Gonca Ozgun ^2^, Ugur Toprak ^3^ and Mehmet Haberal ^4^**
  ^1^  Department of Plastic, Reconstructive and Aesthetic Surgery, Baskent University Faculty of Medicine
  ^2^  Department of Pathology, Baskent University Faculty of Medicine
  ^3^  Department of Biostatistics, Baskent University Faculty of Medicine
  ^4^  Department of General Surgery and Burn Center, Baskent University Faculty of Medicine

**Introduction**: Stasis zone is the encircling area of the coagulation zone which is a critical area determining the depth and width of the necrosis in burn patients. In our study, we aim to salvage the stasis zone by injecting adipose-derived stromal vascular fraction (ADSVF).
**Methods**: Intraperitoneal streptozotocin was administered for the induction of diabetes mellitus (DM) and the development of DM was confirmed by the measurement of blood glucose levels in the blood samples with blood glucometer weekly 48 h after injection. Rats with blood glucose levels above 200 mg/dL were accepted as diabetic. The diabetic animals were followed for 4 weeks before the intervention.
Thermal injury was applied on dorsum of diabetic Sprague Dawley rats (*n* = 20) according to the previously described “comb burn” model. after the burn injury (30 min) on Sprague Dawley rats; rat dorsum was separated into two equal parts consisting of four burn zones (three stasis zones) on each pair. ADSVF cells harvested from inguinal fat pads of diabetic Sprague Dawley rats (*n* = 5) were injected on the right side, while the same amount of phosphate-buffered saline (PBS) was injected on the left side of the same animal.
One week later, the average vital tissue on the statis zone was determined by macroscopy, angiography and microscopy. Vascular density, inflammatory cell density and gradient of fibrosis were determined via immunohistochemical assay.
**Results**: Macroscopic stasis zone tissue survivability percentage (32 ± 3.28%, 57 ± 4.28%), average number of vessels (10.28 ± 1.28, 19.43 ± 1.72), capillary count (15.67 ± 1.97, 25.35 ± 2.15) and vascular density (1.55 ± 0.38, 2.14 ± 0.45) were higher on ADSVF side. Fibrosis gradient (1.87 ± 0.51, 1.50 ± 0.43) and inflammatory cell density (1.33 ± 0.40, 1.20 ± 0.32) were higher on the PBS side.
**Conclusions**: Macroscopic and microscopic findings determined that ADSVF has a statistically significant benefit for salvaging stasis zone on acute burn injuries in DM.

*P.143*

**The Effect of Lactate on Pain, Oxygenation, and Wound Healing**

**Herbert Haller ^1^, Matthias Rapp ^2^, Frank Sander ^3^, Mehmet Demircan ^4^ and Lars Kamolz ^5^**
  ^1^  Ukh Linz of Auva/retired; HLMedConsult
  ^2^  Clinic for Orthopedics, Trauma Surgery and Sports Traumatology, Burn Center, Marienhospital
  ^3^  Burn Center, Plastic Surgery of Trauma Hospital Berlin, Warener Strasse 7
  ^4^  Pediatric Intensive Burn Care Unit, Department of Pediatric Surgery, Faculty of Medicine, Inönü University
  ^5^  Department of Surgery, Plastic, Aesthetic and Reconstructive Surgery, Medical University Graz, Auenbruggerplatz 29, 8036 Graz

**Objectives**: Burns and Wound treatment with polylactide membranes has a long history dating back to 1998. over the years, it has shown many significant positive effects. Undisturbed wound healing and effective pain relief, dressing changes, and a reduced number of skin grafts in the case of partial-deep burns together with rapid stabilization of the patient come to the for e. In addition, recent results combined with previous results updated the understanding of the mechanism of action.
**Methods**: David Julius and Ardem Papapotian received the 2021 Nobel Prize in Medicine for work on TRPV and TRPM ion channels and their impact on pain perception. The influence of these channels on pain and activation of the burn cascade is presented. Lactate can block these channels and influence pain perception and wound healing. Mechanisms and methods of topically applied lactate and the influence on wound healing are presented.
**Results**: The thermally activated TRPV channels initiate pain and influence cell death through Ca++ accumulation and increased sodium influx. Cell changes release DAMPs, and via TLRs, P2X, and AIM2 receptors, the entire burn cascade is affected, and the itching [1].
However, TRPV channels can be successfully blocked by lactate, which is not only manifested in the pain-relieving effect [2]. Lactate is an essential co-factor in wound healing, without which adequate angiogenesis cannot occur [3], although it can also be applied topically. In addition, it contributes to the normalization of the wound pH and thus to the reduction in excessive matrix metalloproteinase activity. Lactate adjusts wound pH to optimal growth and proliferation for keratinocytes and fibroblasts [4]. The improved vascularization increases local oxygen tension, which increases the ability of leukocytes to kill bacteria oxidatively and reduces the risk of infection [5].
**Conclusions**: Lactate and oxygen are essential to wound healing through TRPV channel blockage, pH adjustment, and cytokine induction. Lactate can be used systemically or topically. The release of lactate can be controlled in terms of amount and duration by the type of membranes used.

**Keywords**: lactate; synthetic polylactide membranes; TRPV channel; SupraSDRM^®^ CW; Suprathel^®^ angiogenesis; wound healing; pain reduction; tissue neogenesis

**References**
  1. Morgan, M.; Depuis, J.R.; Frøsig-Jørgensen, M.; Lewis, R.J.; Cabot, P.J.; Gray, P.D.; Vetter, I. Burn Pain: A Systematic and Critical Review of Epidemiology, Pathophysiology, and Treatment. *Pain Med.* **2018**, *19*, 708–734. https://doi.org/10.1093/pm/pnx228.
  2. De la Roche, J.; Walther, I.; Leonow, W.; Hage, A.; Eberhardt, M.; Fischer, M.Reeh, P.W.; Sauer, S.; Leffler, A. Lactate is a potent inhibitor of the capsaicin receptor TRPV1. *Sci. Rep.* **2016**, *6*, 36740. https://doi.org/10.1038/srep36740.
  3. Hunt, T.K.; Aslam, R.; Hussain, Z.; Beckert, Z. Lactate, with Oxygen, Incites Angiogenesis. In *Oxygen Transport to Tissue XXIX*; Springer: Berlin/Heidelberg, Germany, 2016; pp. 73–80. https://doi.org/10.1007/978-0-387-74911-2_9.
  4. Strohal, R.; Mittlböck, M.; Hämmerle, G. The Management of Critically Colonized and Locally Infected Leg Ulcers with an Acid-Oxidizing Solution: A Pilot Study. *Adv. Ski. Wound Care* **2018**, *13*, 163–171. https://doi.org/10.1097/01.ASW.0000530687.23867.bd
  5. Allen, D.B.; Maguire, J.J.; Mahdavian, M.; Wicke, C.; Marcocci, L.; Scheuenstuhl, H.; Chang, M.; Le, A.X.; Hopf, H.W.; Hunt, T.K. Wound Hypoxia and Acidosis Limit Neutrophil Bacterial Killing Mechanisms. *Arch. Surg.* **1997**, *132*, 991–996 https://doi.org/10.1001/archsurg.1997.01430330057009.

*P.144*

**Use of an Apertured Silicone Dressing as a Primary or Secondary Wound Contact Layer**

**Michael Serghiou and Jonathan Niszczak**
  Bio Med Sciences Inc.

**Introduction**: The management of wounds requires the clinician to utilize a wide variety of wound dressings in managing a variety of wound etiologies. A versatile wound dressing should have the ability to manage exudates, adhere to the wound site and surrounding skin without disturbing the healing process, allow for wound visual observation and provide a layer of protection. A new wound contact layer dressing comprising a uniformed tricot material coated with a proprietary silicone technology was evaluated in cases involving burns, decubiti, abrasions, and other wound types. The dressing comprises a woven matrix partially coated with a silicone polymer on one side, such that the apertures of the matrix remain open.
**Methods**: A series of clinical cases provides examples of how this new dressing is utilized. The dressing was applied directly on wounds (primary wound contact layer), or over a biosynthetic/synthetic skin substitute (secondary dressing). In both scenarios, an absorbent dressing was applied over the silicone dressing and was changed as needed. Wound size and re-epithelialization rates were assessed. Wounds were evaluated and photographed biweekly. Pain was assessed via a visual analogue scale and the amount of patient-required pain medication.
**Results**: The dressing remained intact as a primary dressing for 7 days. When used as a secondary dressing over a poly-lactic membrane and spray cell technologies, it remained intact for up to 14 days allowing for undisturbed and complete re-epithelization. Data on donor site coverage, as a standalone option or in conjunction with a sheet biosynthetic membrane, demonstrated faster healing, minimal pain and required fewer dressing changes as compared with other standard-of-care options.
**Conclusions**: This new wound-contact-layer-managed wound exudates well while allowing for uninterrupted re-epithelialization. When used over a sheet biosynthetic skin substitute, a spray-on biosynthetic skin substitute or as a standalone option, complications were reduced as the dressing provided the necessary protection to allow for the biosynthetic skin substitutes to serve their intended purpose. Dressing changes were well tolerated and, in most cases, pain was minimized as compared with other standard of care options. This dressing warrants further study on a broader spectrum of wounds including deep partial and full-thickness burns with or without biosynthetic skin substitutes. This dressing demonstrates a simple and effective option to manage wounds in a cost-effective manner.

*P.145*

**Biochemical Profiling of Burn Wound Healing following Treatment with Acellular Skin Grafts**

**Aristotelis Kotronoulas ^1^, Marieke Heijink ^2^, Giorgios Stamatakis ^3^, Martin Giera ^2^, Martina Smiotaki ^3^, Randolph Stone II ^4^, Hilmar Kjartansson ^5^ and Óttar Rolfsson ^1^**
  ^1^  University of Iceland
  ^2^  Leiden University Medical Center
  ^3^  Biomedical Sciences Research Center “Alexander Fleming”
  ^4^  US Army Institute for Surgical Research
  ^5^  Kerecis

**Objectives**: How skin grafts modulate the biochemical profiles of burn wound healing is not understood, but it is of importance for understanding their mechanism of action and advancing wound care. We are working towards understanding how skin grafts influence the lipid, metabolite and protein profiles of burn wound healing using a mass-spectrometry-based data-driven approach.
**Methods**: Partial (PTBW)- and full (FTBW)-thickness burn wounds (*n* = 4 for each type) were created on Yorkshire pigs (*n* = 4). The PTBW were treated either with fish skin graft (FSG) or with fetal bovine dermis grafts. FTBW were treated either with FSG or cadaver skin initially and followed by a split-thickness skin graft. Punch biopsies were collected over time (day 7, 14, 21, 28 and 60) and extracted in order to measure approximately 50 derivatives of EPA, DHA, arachidonic acid (AA), linoleic acid (LA) by targeted UPLC-MS/MS. Untargeted metabolomics analysis using UPLC/Q-TOF-MS, and label free quantitative shotgun proteomics by UPLC/Orbitrap-MS of tissue biopsies were also performed.
**Results**: In the partial thickness burn wounds, EPA and DHA derivatives, including 18-HEPE and 17-HDHA, were significantly increased at day 7 in the FSG treated wounds. A similar but non-significant trend was observed in full-thickness wounds. Prostaglandin F2a and its 15-keto derivative from the AA pathway along with 13-HODE and 13-HOTrE from the LA pathway increased significantly at day 7 independent of treatment. Statistical analyses of proteomics data are currently pending. Untargeted metabolomics of tissue biopsies yielded 1367 features common to all analyzed biopsies. Irrespective of wound type, the changes in metabolite profiles fell into defined temporal trajectories during healing with changes observed to selected nucleotides, amino acids and lipids.
**Conclusions**: Our results demonstrate that the type of skin graft used to treat burn wounds can alter the for mation of lipid mediators involved the resolution of inflammation and pain, and influences metabolic biomarkers of wound healing.

*P.146*

**Retrospective Evaluation of Progenitor Biological Bandage Use: A Complementary and Safe Therapeutic Management Option for Prevention of Hypertrophic Scarring in Pediatric Burn Care**

**Karim Al-Dourobi, Alexis Laurent, Lina Deghayli, Marjorie Flahaut, Philippe Abdel-Sayed, Corinne Scaletta, Murielle Michetti, Laurent Waselle, Jeanne-Pascale Simon, Oumama El Ezzi, Wassim Raffoul, Lee Ann Applegate, Nathalie Hirt-Burri and Anthony de Buys Roessingh**
  Plastic, Reconstructive and Hand Surgery Service, Lausanne University Hospital

Progenitor biological bandages (PBB) have been continuously applied clinically in the Lausanne Burn Center for over two decades. Vast translational experience and hindsight have been gathered, specifically for cutaneous healing promotion of donor-site grafts and second-degree pediatric burns. PBBs constitute combined advanced therapy medicinal products, containing viable cultured allogeneic fetal dermal progenitor fibroblasts. Such constructs may partly favor repair and regeneration of functional cutaneous tissues by releasing cytokines and growth factors, potentially negating the need for subsequent skin grafting, while reducing the for mation of hypertrophic scar tissues.
This retrospective case–control study (2010–2018) of pediatric second-degree burn patients comprehensively compared two initial wound treatment options (i.e., PBBs versus Aquacel^®^ Ag) applied ten to twelve days post-trauma.
The results confirmed the clinical safety of PBBs with regard to morbidity, mortality, and overall complications. No difference was detected between groups for length of hospitalization or initial relative burn surface decreasing rates. Nevertheless, a trend was observed in younger patients treated with PBBs, requiring fewer corrective interventions or subsequent skin grafting. Importantly, significant improvements were observed in the PBB group regarding hypertrophic scarring (i.e., reduced number of scar complications and related corrective interventions).
Such results establish evidence of clinical benefits yielded by the Swiss fetal progenitor cell transplantation program and favor further implementation of specific cell therapies in highly specialized regenerative medicine.

*P.147*

**Use of Polylactic Membranes as a Dressing for Sprayed Autologous Skin Cells in Deep Dermal Burn Wounds**

**Bernd Hartmann, Simon Kuepper and Claudia Belfekroun**
  Department of Plastic Surgery/Burn Unit, Unfallkrankenhaus Berlin

**Objective**: Polylactic membranes have demonstrated usefulness in the treatment of superficial and partial thickness burns because of pain reduction, ease of application, reduced workload and good clinical results. This paper presents the results of 103 patients treated with sprayed autologous skin cells and covered with polylactic membranes.
**Methods**: Retrospective quality control in 103 patients from 2003 to 2018, in which partial-thickness burns were treated with sprayed keratinocytes and covered with polylactic membranes. From 2003 to 2015, evaluation of burn depth was conducted from a clinical aspect; from 2016 to 2018, laser doppler imaging (LDI) was used. Patients were included when the time of healing was expected to be longer than 15 days.
**Results**: There were 67 male and 36 female patients with an average TBSA of 17.8% ± 14.8 median: 14. Average second-degree burn was 8.52% (±9.8), average 3rd° burn was 9.47% plus ± 12.2. Age was, on average, 39 years (median 42). No significant difference could be found between male and female in age and TBSA distribution. ABSI was 5.97. Nearly all patients had significant comorbidities. Source of injury was explosion (18), flame (55), scald burn (17), contact burn (2), electricity (2), suicide attempt (6) and others (2). Operation was started on average 4 days (±3) after the injury (median 3.0). Sprayed keratinocytes were applied in average 12 days after the first operation (±7.6). Using LDI, the time for keratinocytes application was reduced to 7.6 days. Three different cell types were applied to twelve different regions. Healing time (time to 99% healing) was on average 8. 33 days (median: 7). The total treatment time to healing was shorter in the non-cultured cell suspension treated patients due to the lacking necessity of preculturing cells. In six patients, a second transplantation had to be carried out. The last control was performed on average 96 days (±368) after spraying. A scar was visible in four patients, pigment changes happened in nine patients, hypertrophic scarring occurred in five patients, and infections were suspected or confirmed in six patients.
**Conclusions**: Polylactic membranes proved to be an adequate dressing for sprayed keratinocytes in deep 2nd ° burns. There was no need for frequent dressing changes. Infections in other areas of the body did not spread on the wounds covered with polylactic membranes. Other than predicted, 91% of the wounds were healed within 14 days.

*P.148*

**First Experiences with an Polyurethan Biodegradable Temporising Matrix in Wounds with Complications**

**Ina Nietzschmann**
  BG-Clinic Bergmannstrost, Clinic for Plastic and Hand Surgery/BVZ

**Objectives**: The biodegradable dermis substitute is a purely synthetic polyurethane polymer consisting of a matrix, an adhesive layer and a sealing membrane.
It is used for temporary wound closure and conditioning of the wound bed. We have been using the biodegradable matrix for approx. 2 years in complicated, protracted treatment courses for wound conditioning. The affected areas are covered with split-skin at different times. We aim to present our indications for the biodegradable matrix and describe our experience.
**Methods**: In our department, we have treated a total of 15 patients with the novel biodegradable matrix since May 2020. We primarily treated prolonged treatment courses after decollement injuries and secondary after-burns.
We present three cases as examples.
**Results**: We present three cases with decollement injuries und burns. In all three cases, we obtained a complete wound closure with good cosmetical and functional results without any infection.
**Summary**: Our initial experiences with the new biodegradable polyurethane dermal substitute were positive. The biogradable temporising matrix is applicable in different indications. The rate of infections is lower and there is less exposure to other materials. The quality of the scar is better than with split-skin only.
We will continue to use this material for certain indications.

*P.149*

**Treatment of Superficial and Partial Thickness Hand Burns Using Cultured Human Keratinocytes Epifast^®^**

**Jose Joel Casas Beltran**
  Instituto Mexicano Del Seguro Social

**Introduction**: The hands represent one of the most highly affected areas of the body as a result of burn injuries. In addition to this, it is difficult to use dressings to cover the area, due to the normal irregular anatomy of the hand skin. New treatments for burns aim to reduce pain, inflammation and prevent infections with the aim of reducing re-epithelialization time and obtaining better aesthetic results. Dressing with cultured human keratinocytes stimulates migration of cells and promotes growth factors, playing an important role in the proliferative phase of restoration of tissue structure and function; however, there is no information on clinical hand burn studies published.
**Objective**: To evaluate pain, infection rates, re-epithelialization time, and long-term scarring using cultured human keratinocytes as a treatment for superficial and partial-thickness hand burns.
**Materials and Methods**: Records of patients attended for hand burns treated with cultured human keratinocytes Epifast^®^, 6 months prior to this study, analyzing age, sex, cause of burn, depth, and percentage affected. Measures of pain scale (Verbal Rating Scale 1–10), time of re-epithelialization and complications recorded. POSAS (Patient and Observer Scar Scale) and VSS (Vancouver Scar Scale) applied at 6 months, recording data. Exclusion criteria: Infection prior to application and full-thickness burns.
**Results**: From October 2017 to October 2021, 50 files of patients with hand burns treated with cultured human keratinocytes Epifast^®^ were collected. The cohort comprised 86% males (43) and 14% females (7) with a mean age of 29 years (range 1 to 87 years), mean percentage 15% (range 1% to 50%), 94% 2nd-degree burn (47 cases), 6% 3rd-degree burn (three cases that were excluded). Cause of burns: 88% fire, 12% scald, 6% chemical and 4% contact with hot material. The pain scale mean was 2 (range 1–4), mean re-epithelialization was 8 days (range 5–14 days). No complications were registered including infections or allergies. POSAS and VSS 6.5/<1
**Conclusions**: Dressing with cultured human keratinocytes, Epifast^®^, demonstrated effectiveness in reducing pain, reducing inflammation and preventing infections. It also reduced re-epithelialization time and prevented hypertrophic scarring. It can be safely used on skin surfaces where the epithelium is missing, helping improve quality of life by functional and aesthetic outcomes, making it a great option for burn treatment.

*P.150*

**Challenging Donor Site Problems: Corticosteroid Cream—Hazard or Opportunity?**

**Pia Tiimo**
  HUS, Helsinki Burn Centre

Donor site problems are common after skin graft operations and can arise from heavy bleeding, dressing detachment or slippage, infection, selection of the wrong product and inadequate patient guidance.
The donor site should heal within two weeks with the primary dressing. Suprathel^®^ or Aquacel foam^®^ dressings are usually used at the Helsinki Burn Centre. When Suprathel^®^ is used, the wound is initially evaluated daily with changing of the upper absorbent dressings and, thereafter, only when necessary as wound discharge decreases. Aquacel foam^®^ is kept in place for two weeks unless the dressing becomes full of exudate. In the latter case, a new dressing is applied within 1–2 days and is left in place until epithelialization is complete.
**Objectives**: The aim was to analyze the use of corticosteroid cream in the treatment of donor sites with delayed (>2 weeks) healing. Another aim was to reduce or find an alternative to the use of silver products.
**Methods**: The study period was January 2020–March 2022 and included 15 patients with an average age of 50 years. The most common donor site was a lower limb. Follow-up was daily during inpatient care or weekly in the outpatient clinic/image consultation.
The nurses always consult the doctor regarding the choice of wound product and treatment with corticosteroid cream was only commenced on the doctors’ orders. Possible donor site infections requiring antibiotic treatment were excluded from the use of the cream.
Corticosteroid treatment was daily and involved cleansing and application of an overlying non-stick dressing. Outpatient-directed treatment included oral and written guidance for the patients.
**Results**: Corticosteroid therapy was administered for an average of 1–2 weeks and treatment progress monitored using regular photographs. Corticosteroid cream provided an alternative to silver dressings and reduced costs and treatment time in some patients.
**Conclusions**: Corticosteroid cream treatment initiated at the appropriate time can reduce the use of silver products. It may also reduce the need for antibiotics. The functionality of the overlying dressing is important as well as the effect of the dressing on pain experienced by the patient. In further studies, it would be interesting to look at the effect of corticosteroid ointment on the pain experience and the use and quantity of analgesics.

*P.151*

**Suprathel^®^—How to Establish an Appropriate and Efficient Application Algorithm for Surgeons Working in Burn Centres: An Attempt to Identify Risk Factors for Burn Wound Conversion Phenomenon**

**Artur Wielgórecki, Wojciech Łabuś, Marcin Gierek, Karolina Mikuś-Zagórska and Karolina Ziółkowska**
  Dr. Stanisław Sakiel Centre for Burns Treatment

**Objectives**: The main challenge for surgeons working in burn centres is to keep burn wounds from becoming infected and to stimulate healing as quickly as possible. Proper patient qualification for a synthetic skin substitute can help avoid further surgery, which may otherwise be necessary in the event of burn wound conversion, when superficial partial-thickness burns develop into deeper burns. Fewer surgical interventions mean better cost-effectiveness of treatment.
**Methods**: A clinical observational study was conducted. Such efficacy studies are often viewed with scepticism due to the fact that patients are not randomized to treatment, which means that results are more prone to error. However, we believe that in such a specific and small group of patients as burn patients qualified for the application of a synthetic skin substitute, the observations made will initiate the creation of a treatment algorithm that will allow for the proper qualification of patients for a specific treatment procedure—in this study, it is the application of Suprathel^®^. Our study evaluated the incidence of burn wound conversion in a group of participants undergoing burn wound treatment through the application of Suprathel^®^. At the Dr Stanislaw Sakiel Centre for Burn Treatment, Suprathel^®^ has been used in over 250 patients from 2018 to 2021. Hospitalization time and surgical procedures used were reviewed. The International Statistical Classification of Diseases and Related Health Problems (IDC) codes of the initial disease diagnosis were compared with the final diagnosis. A review of the current literature was conducted to identify recent studies on the pathogenesis of burn wound conversion.
**Results**: Wound healing was observed in most cases after Suprathel^®^ implementation. There have been more than a dozen cases of burn wound conversion among patients undergoing this type of treatment. It seems that the main categories of risk factors for this phenomenon have been identified.
**Conclusions**: A preliminary review has led to the identification of a set of potential factors driving the burn wound conversion phenomenon. Understanding the pathophysiologic substrate contributing to deeper burn progression is an essential prerequisite for planning any intervention. We need to improve our knowledge in this area in order to effectively qualify patients for appropriate procedures that are likely to be successful.

*P.152*

**Selective Enzymatic Debridement and Modified MEEK Technique in the Treatment of Extensive Burns**

**Jasminka Minic, Enrico Vigato, Martina Hubova, Edoardo Dalla Pozza and Maurizio Govern**
  Plastic Surgery and Burn Unit, A.O.U:I. of Verona

**Background**: Selective enzymatic debridement has emerged as a valid alternative for the treatment of extensive burns, especially for partial-, mixed- or full-thickness burns. The lack of the donor sites, regardless, remains the main reconstructive challenge. Autologous skin grafting represents the procedure of choice when available, and modified MEEK technique of grafting maybe suitable in this regard to optimize the final outcome.
**Methods**: We performed a single-cohort retrospective analysis of nine consecutive burn patients who underwent both enzymatic debridement and subsequently modified MEEK technique from 2018 to 2021 at the Verona Burn Unit. Demographic and clinical data, including mechanism of injury, surgical treatment, complications, necessity for re-grafting, further surgery and aesthetic outcome, were collected by means of electronic medical record reviews.
**Results**: All patients were affected by large burns of mixed and deep dermal thickness. The average age was 52 years old and the mean TBSA was 40.7%. Local infection sustained by poor general conditions was the main complication for all patients after surgery, which led to the loss of the micro-graft and the need for re-intervention in two cases. All but one patient survived. Selectiveness of enzymatic debridement seemed to improve the quality of scars and the final functional and aesthetic result of micro grafting.
**Conclusions**: Combined selective enzymatic debridement and MEEK techniques are a powerful synergic combination for the treatment of extensive burns, both for short-term and long-term outcomes.

*P.153*

**Evaluation of the Safety and Effectiveness of Progenitor Biological Bandages in Burn Care (Bru_PBB Clinical Study)**

**Marjorie Flahaut ^1,2,3^, Philippe Abdel-Sayed ^1,2,3^, Nathalie Hirt-Burri ^1,2,3^, Corinne Scaletta ^1,2,3^, Professor Wassim Raffoul ^1,3^, Professor Lee Ann Applegate ^1,2,3^ and Anthony De Buys Roessingh ^3,4^**
  ^1^  Plastic, Reconstructive and Hand Surgery Service (CPR), University Hospital (CHUV)
  ^2^  Unit of Regenerative Therapy (UTR), University Hospital (CHUV)
  ^3^  Lausanne Burn Center, University Hospital (CHUV)
  ^4^  Children and Adolescent Surgery Service (CHPD), University Hospital (CHUV)

**Objectives**: The current gold-standard treatment for third-degree or deep-second-degree burn wounds is skin autografting, which creates a donor site wound (DSW). The Burn Center of the CHUV (Lausanne, Switzerland), with more than 20 years of clinical experience in the therapy of severe burns, has developed progenitor biological bandages (PBB), composed of human skin progenitor cells from GMP clinical cell banks (i.e., FE002-SK2 cell type) seeded on a biodegradable collagen scaffold (Resorba^®^). A clinical study aiming to confirm the safety and to demonstrate the efficacy of PBBs in the allogeneic cytotherapeutic treatment of the DSW and 2nd-degree burn wounds is currently under preparation in the CHUV.
**Methods**: Seventy-six patients among pediatric and adult severe burn patients will be enrolled in this randomized prospective trial. Patients (i.e., candidates for a skin autograft) will be randomly allocated for the treatment of their DSW in two groups. DSWs of Group A will be treated with PBBs and DSWs of Group B will be treated with standard bandages (Jelonet^®^). Bandages will be placed on the DSW and maintained in place for a maximum of 15 days with two dressing’s changes at Days 5 and 10. Wound status (i.e.,% of re-epithelialisation), as well as complications, will be evaluated during this acute cytotherapeutic treatment phase. All patients will then be followed during five years for a long-term evaluation of outcomes, with measures of skin quality.
**Results**: In order to verify the hypothesis of high therapeutic performance of PBBs as compared with the standard treatment, short-term safety and efficacy will be evaluated during the acute treatment phase, as well as for the long-term maintenance treatment in order to measure skin quality of the repaired wound. The study was approved by the respective and competent Swiss health authorities (i.e., cantonal ethics commission and Swissmedic) and is ready to include the first burn patients.
**Conclusions**: By confirming the safety and the effectiveness of the use of PBBs for treating patients with 2nd-degree burns, we will regularly implement this historically documented safe and standardized allogeneic cell therapy for acute wounds in the multidisciplinary therapeutic approach of severely burn patients admitted in our Burn Center.

*P.154*

**Simplified Easy-to-Use Hydro Active Dressing for Burns on Hard-to-Dress Areas Relieves Pain, Increases Healing, Prevents Complications and Saves Resources in Terms of Time and Cost**

**Janni Mulvad Nielsen**
  Rigshospitalet, Copenhagen University Hospital

**Objectives**: The Burns Unit at Copenhagen University Hospital, Rigshospitalet, admits approx. 250 patients per year. This includes burn injuries from Denmark, the Faroe Islands and Greenland.
In 2019 we started a project on facial burns as we had challenges treating this hard-to-dress area.
The objective was to explore whether use of Epicite Hydro (EH) could simplify and optimize bandaging of facial burns, improve healing time, reduce time of hospitalization, save time for the nurses as well as coverage patient experience.
Facial burns are expected to heal within 14 days. We saw a complete epithelization on av. Day 9.
Of the 40 patients included, 33 patients had complete data collection. Due to satisfying experiences, positive results, and cost-savings with EH, we changed the Treatment Guideline on facial burns and have now initiated further trial on another hard-to-dress areas, genital burns and scalds.
**Methods: for** 9 months 2019/2020, patients admitted with facial burns had EH applied within 48 h after the accident. A quantitative questionnaire was completed, including patients and nurses’ experiences of application, treatment, duration of treatment, observation, and removal.
The ongoing trials are documented in a quantitative questionnaire focusing on pain, edema, itching, signs of infection, wearing comfort, healing, as well as ease-to-use, time consumption plus clinical photos.
We expect to collect data on 5–8 genital burns before EBA.
The facial project and ongoing trial are not funded.
**Results**: TBSA involvement of the facial burns varied between 0.5–4.5%, three patients with an av. facial TBSA of 73.4%. Burns were superficial and deep 2nd- as well as 3rd-degree burns.
Age: 2–84 years, 6 pediatric, av. age for adults: 27 years.
All 33 facial burns healed on average of 8,9 days.
22 patients found the application of EH “very comfortable”, 18 experienced “no pain”, 9 described “some pain”.
Nurses finds EH easy to apply even on hard-to-dress areas, the majority spends less than 10 min applying it.
**Conclusions**: EH seems to relieve pain on facial burns, shortens hospitalization, and improve healing time. The application is easy and less time consuming. According to patients’ experiences, it has proven to be a comfortable treatment.
The Burns Unit changed the Danish Treatment Guideline for facial burns, and we are looking for ward presenting our experiences and results from our on-going trial on genital burns at EBA.
The positive feedback from nursing staff treating these hard-to-dress areas promotes the implementation of changes and new routines.

*P.155*

**The Use of Polylactic Acid in Acute Burns**

**Giorgia Fara, Vanessa Canu and Alma Posadinu**
  University Hospital

Polylactic acid is one of the most reliable skin substitutes in the management of burn injuries. It preserves residual dermal tissue, thus allowing for primary healing with a single treatment. for this reason, the polylactic acid is used at our center in split-thickness skin graft donor sites, intermediate- and deep-second-degree pediatric skin burns, and after enzymatic debridement for eschar removal in deep burns to stimulate and support the healing of cutaneous and epidermal structures.
**Objectives**: In our experience, the polylactic acid has shown remarkable effectiveness for patient comfort and management of acute burn injury pain, which allows for undisturbed dressing changes while improving healing time and reducing need for hospitalization. We observed that the use of the polylactic acid together with proteolytic enzymes and bromelain may further improve healing times, particularly in burns with full or even partial dermal sparing.
**Methods**: We treated 10 patients with deep second-degree burns with the polylactic following debridement with proteolytic enzymes and bromelain. We then evaluated the following outcomes:
  1. Pain assessment with visual analog scale (VAS);
  2. Accelerated cutaneous re-epithelialization;
  3. Reduced risk of infection.
**Results**: We found that 70% of patients showed no evidence of bacterial or fungal wound contamination. The average time for re-epithelialization was 14 days with good pain control since the first application, as demonstrated by the reduced need for pain relief drugs.
**Conclusions**: Even if limited by the small sample size and the absence of a control group, our study suggests that the association of the polylactic acid and proteolytic enzymes and bromelain is effective for pain control in severe burn injuries and clearly accelerates cutaneous re-epithelialization with low risk of infection. These results encourage us to extend our research and recruit more patients to the study.

*P.156*

**Influencing the Ratio of Dermal to Epidermal Tissue during Wound Healing by Controlling Moisture Content**

**Alexandru-Cristian Tuca ^1^, Ives Bernardelli de Mattos ^2^, Martin Funk ^3^, Raimund Winter ^1^, Alen Palackic ^1^, Florian Groeber-Becker ^2,4^, Daniel Kruse ^2^, Fabian Kukla ^5^, Thomas Lemarchand ^5^ and Lars-Peter Kamolz ^1,6^**
  ^1^  Division of Plastic, Aesthetic and Reconstructive Surgery, Department of Surgery, Medical University of Graz
  ^2^  Department Tissue Engineering & Regenerative Medicine (TERM), University Hospital Würzburg
  ^3^  EVOMEDIS GmbH
  ^4^  Fraunhofer Institute for Silicate Research ISC, Translational Center Regenerative Therapies
  ^5^  TPL Path Labs GmbH
  ^6^  Joanneum Research for schungsgesellschaft mbH, COREMED

**Objectives**: A balanced moist wound environment promotes cell division and cell migration. Furthermore, the effect of different cytokines, chemokines and growth factors is promoted and thus wound healing is supported. Depending on the type of wound, the amount of dermis and epidermis to be regenerated may vary. Accordingly, the wound dressing should be selected.
The aim of the present study was to investigate whether it is possible to influence the ratio of dermis to epidermis through specific regulation of moisture.
**Methods**: Commercially available dressing materials used in daily hospital routine were used to conduct the study. The biotechnologically produced cellulose wound dressing epicitehydro with a high water content of at least 95% as isotonic saline solution was used as the primary wound dressing. To control moisture in the primary dressing, either cotton gauze (Gazin), fatty gauze (Jellonet), Hydro Fibre (Aquacell Extra) or an occlusive dressing (Opsite Flexifix) were used as secondary dressings.
In previously conducted in vitro experiments, the evaporation kinetics from epicitehydro alone and in combination with the secondary wound dressings were presented. Furthermore, in a standardized dermatome wound model in a pig, the effects of different evaporation behaviors and thus varying wound moisture were investigated in vivo. Afterwards, the wounds were processed histologically and immunohistochemically.
**Results**: The histological results showed that the wounds treated with epicitehydro had varying amounts of new dermis and epidermis, depending on the secondary dressing. The combinations with cotton gauze or the hydrofibre had very low residual moisture, a low amount of new dermis, but the highest re-epithelialization rates. Treatment with fatty gauze as a secondary dressing showed moderate residual moisture and equally moderate amount of new dermis and epidermis. In comparison, the occlusive dressing had the highest residual moisture, hardly any new epidermis, but up to three times as much new vital dermal tissue.
**Conclusions**: The results of the present study show that the wound environment, its moisture and evaporation from the wound can be specifically influenced by the use of different secondary dressings. The present findings may be of great benefit to wound treatment and clinical wound management.

*P.157*

**The Use of Primatrix in Deep Burns following Enzymatic Debridement: A Case Series**

**Laura Bonifazio and Giuseppe Perniciaro**
  Villa Scassi Hospital—Burn Center

**Objective**: Modern burn care requires assets and resources. The focus is on developing novel non-invasive treatment options that will benefit patients in the long run while also saving hospitals money. The goal of this study is to see how well our minimally invasive burn wound treatment technique works in patients who do not need intensive care.
**Methods**: A review of the patients’ medical records was conducted retrospectively. Between January 2020 and January 2021, 52 patients of both sexes (>17 years old) with deep partial-thickness (DPT) and full-thickness (FT) burns, including burns of the extremities, were admitted. Within 24 h of the injury, all of the patients received early enzymatic debridement (ED) (Nexobrid), which can also be used for escharotomy. All of the wounds were covered with Primatrix (PTX), a repair scaffold, after 48 h. Both treatments were carried out at the patient’s bedside, with prior analgesia sedation. Patients were discharged to their homes as they awaited wound closure, with one weekly follow-up appointment planned.
**Results**: There were 52 patients in total, 35 of whom were males. The mean age was 55.4 (17–81). Total body surface area (TBSA) was 13% (5–25). Burn wounds were first diagnosed as 89% deep partial thickness (DPT) and 11% full-thickness (FT) burns, including extremity burns. In comparison to the initial diagnosis, the post-ED did not indicate any FT burns area. for all patients, the demand for blood transfusions and the necessity for an operating room was none. after PTX application, all of the patients reported reduced pain. The average time until discharge was 13 (5–28) days, and the average follow-up in the outpatient clinic was 7 (5–8) days. As for the long-term outcomes, the average period for complete wound closure was 21 (18–25) days. None of the patients died, and none of them had any adverse reactions or complications.
**Conclusions**: The combined treatment strategy NXB + PTX can offer a minimally invasive modality of burn wound treatment, accelerate wound healing, and reduce hospitalization time in patients with non-severe comorbidities, providing greater comfort to patients and benefits to the healthcare system, according to our observation.

*P.158*

**The Use of Fish Skin Grafts for the Management of Burn Wounds**

**Ariel Aballay, Jason Bregg, Ariana Langholz, Brianna Bell, Linda Leonard, Susan Sommers, Mariah Sturges, Eric Tolchin and Kristen George**
  Allegheny Health Network

**Objectives**: Current advances in technology have made possible the modification in live tissues structures with the purpose of improving its intrinsic, innate abilities to support the healing of burns and wounds. This case series examines how fish-skin grafts assist in the healing cascade and describes wound healing progression over time after application.
**Methods**: In this case, series, burn patients of varying age and ethnicity with various burn and/or wound types and sizes, received fish-skin grafts applied post debridement and/or excision to the wound bed. The fish-skin graft effects on wound healing were evaluated regarding time to wound closure, restoration of barrier function, skin pigmentation, complications, and pain severity.
**Results**: Overall, the cohort of patients treated with fish-skin grafts had an observed decrease in healing time, complications, and patient-reported pain. Additional observational findings of skin pigmentation and barrier function were also noted. Clinical challenges encountered are outlined. Evidence of product assimilation is presented.
**Conclusions**: In this case, series, the fish-skin graft demonstrated similar or better outcomes to our previous standard of care treatment. Certain characteristics observed during and after wound closure indicates relatively high speed of product assimilation compared with standard of care products. Further studies are needed to assess factors that may be contributing to faster healing and outcomes.

